# Enhancing cap-independent translation of linear mRNA

**DOI:** 10.1038/s41467-025-64257-6

**Published:** 2025-10-16

**Authors:** Sebastian Golojuch, Brendan Largey, Afaf H. El-Sagheer, Tom Brown

**Affiliations:** 1 Department of Chemistry, Chemistry Research Laboratory, University of Oxford, Oxford, UK; 2 School of Chemistry, University of Southampton, B30 East Highfield Campus, Southampton, UK

**Keywords:** Nucleic-acid therapeutics, RNA, Fluorescence imaging, Transcription, Nucleic acids

## Abstract

While cap-dependent translation remains the primary focus in mRNA-based therapeutics, cap-independent translation holds promise for targeting diseases ranging from cancer to neurodegeneration. However, cap-independently translated mRNAs are unstable, produce less protein than capped mRNAs, and current methods for their improvement are imperfect. Here, we propose the use of in vitro transcription priming with azido-modified dinucleotide primer and post-transcriptional modification utilising click chemistry to improve the properties of cap-independently translated mRNAs. Our results demonstrate a significant enhancement in mRNA stability and protein output without eliciting immunogenicity. Moreover, we show how the mRNA 5′-end modification strategy can be used to investigate transfection and cap-independent translation processes in cells overcoming burdens associated with previous methods. Together, our findings support cap-independent translation as a viable alternative to the established cap-dependent process and provide tools for further exploration and enhancement of this modality.

## Abstract (web summary)

Cap-independently translated mRNAs could expand the scope of mRNA therapies, but yield limited protein, and existing enhancement strategies are imperfect. Here, the authors install a 5′-end modification that both boosts efficiency and facilitates in-depth characterisation of these mRNAs.

## Introduction

**Fig. 1:**
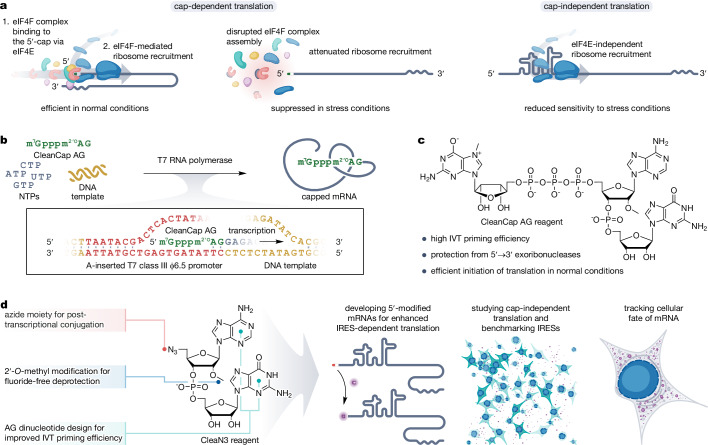
Inspiration and design of the study. **a** Cap-dependent translation is the predominant mode of eukaryotic gene expression and the main focus in therapeutic mRNA development. In this mode of translation, ribosomes are recruited into the mRNA through the eukaryotic translation initiation factor 4F (eIF4F) complex, which is initiated by the 5′-cap binding to eukaryotic translation initiation factor 4E (eIF4E). Although very efficient in normal conditions, cap-dependent translation can be suppressed in stress conditions, e.g., by inhibition of the eIF4F complex formation. A solution to this problem can offer cap-independent translation in which ribosomes can be recruited without the participation of the eIF4F, e.g., through internal ribosome entry sites (IRESs). Nevertheless, in normal conditions, cap-independent translation is not as efficient as its cap-dependent counterpart. **b** 5′-cap can be incorporated into the mRNA during in vitro transcription (IVT). Co-transcriptional capping is carried out by adding a cap analogue (e.g., CleanCap AG reagent) to the IVT reaction mixture. **c** CleanCap AG reagent enables highly efficient 5′-capping of the mRNA. The introduced 5′-cap protects the mRNA from 5′ → 3′ exoribonucleases and efficiently promotes cap-dependent translation in normal conditions. **d** We investigated if a similar IVT priming strategy as for co-transcriptional capping can be used to modify the 5′-end of cap-independently translated mRNAs in order to improve their properties and consequently make cap-independent translation more efficient. To this end, we designed and synthesised CleaN3 – an azido-modified dinucleotide primer. Next, we studied its incorporation into transcripts and their further post-transcriptional modification with a model molecule – AF647. We comprehensively evaluated the properties of the modified mRNAs with particular emphasis on translation efficiency, stability, and immunogenicity. Finally, we explored how the modified transcripts can be utilised to study cap-independent translation and track the cellular fate of mRNAs.

Synthetic mRNAs have great utility as vaccines and hold promise for tackling currently undruggable diseases^1,2^. Most mRNA-based therapeutics currently under development or already approved, i.e., COVID-19 vaccines: Spikevax and Comirnaty, utilise cap-dependent translation (Fig. 1a)^3,4^. This choice is dictated by the stabilising role of the 5′-cap and its efficient promotion of translation^5^.

The 5′-cap can be introduced to mRNA by in vitro transcription (IVT) priming (Fig. 1b). However, early implementation of this approach had limitations such as incomplete/faulty capping and reduced IVT yields^6,7^. The development of trinucleotide cap analogues^8^, notably CleanCap AG (Fig. 1c), has addressed these issues by introducing a dinucleotide sequence involved in base-pairing with A-inserted T7 class III φ6.5 promoter during transcription initiation^9,10^. The simplicity of co-transcriptional capping made it attractive not only for manufacturing mRNA vaccines but also for developing modified transcripts for improved stability^11^, enhanced or spatiotemporally controlled translation^12–15^, facile purification^16^, and investigation of RNA-related processes^17–21^.

While cap-dependent translation is the predominant mode of eukaryotic mRNA expression, certain endogenous and viral mRNAs utilise cap-independent mechanisms^22^. These involve specific mRNA features such as internal ribosome entry sites (IRESs) and cap-independent translational enhancers (CITEs), facilitating ribosome loading and translation without the 5′-cap (Fig. 1a)^23^. These elements have been identified in ~10% of human mRNA^24^. Emerging evidence suggests that certain cell phenotypes exhibit impairment of cap-dependent translation, leading to relative upregulation of cap-independent translation (Fig. 1a)^25,26^. This shift can drive differential gene expression which is crucial for cell survival under stress^22,26–28^. Furthermore, enhanced cap-independent translation has been implicated in various human diseases^22,28^, including cancers^29–31^, diabetes^32,33^, and neurodegenerative disorders^34^.

As synthetic mRNAs advance from vaccines to more robust therapeutics, it will be critical to understand how IRESs and CITEs contribute to mRNA translation and whether they can be leveraged for tissue-specific vector optimisation. As an alternative to 5′-cap, IRESs may improve protein yields while targeting tissues with altered regulation of translation. However, because linear cap-independently translated (CIT) mRNAs lack a protective 5′-cap, their lifespan in cells is significantly reduced, drastically lowering their translational output^35^. Additionally, current methods of characterisation and validation of IRESs and CITEs are labour-intense and prone to artefacts leading to misinterpretation of the results^36–38^. In order to investigate and exploit components promoting cap-independent translation to improve mRNA design, novel molecular tools are required.

Here we demonstrate that IVT priming can be used to significantly enhance properties of CIT mRNA and facilitate its characterisation (Fig. 1d). We prime the transcript with an azido-functionalised dinucleotide (CleaN3), enabling efficient post-transcriptional 5′-modification via strain-promoted azide-alkyne cycloaddition (SPAAC). Using AF647 as a model modification, we show enhancement of the mRNA properties, particularly translation efficiency, stability, and immunogenicity. Finally, we demonstrate the applicability of the 5′-modification strategy to study IRES-driven translation and CIT mRNA cellular fate, overcoming burdens associated with previous methods.

## Results

### CleaN3 can be efficiently synthesised on a solid support

**Fig. 2:**
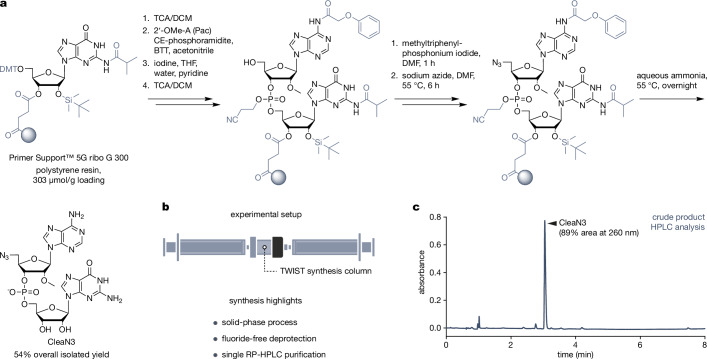
Synthesis of CleaN3. **a** We carried out the synthesis of CleaN3 on high-loading polystyrene support with RNA G attached as the first nucleoside. We added the second nucleoside (2′-*O*-methyl A) using a standard oligonucleotide synthesis cycle. We introduced the azide moiety via 5′-hydroxyl-to-iodide and 5′-iodide-to-azide conversion, after which we deprotected and cleaved CleaN3 from the solid support. **b** We carried out the synthesis in a TWIST synthesis column and used syringes to manually pass reagents through it. The experimental setup and synthesis design allowed all the steps to be carried out on the solid support, as well as enabled fluoride-free deprotection and single RP-HPLC purification. **c** HPLC chromatogram (absorbance at 260 nm) of the crude synthesis product. Source data are provided as a Source Data file.

Building on the highly efficient co-transcriptional capping with CleanCap, we designed CleaN3 to achieve a similar mode of action by employing AG dinucleotide sequence base-pairing with A-inserted φ6.5 T7 promoter (Fig. 1d). To synthesise it, we employed a solid-phase process on a high-loading (303 µmol/g) polystyrene resin (Fig. 2a, b). Current methods for synthesising cap analogues or other IVT primers involve multistep deprotection employing fluorides (tetra-n-butylammonium fluoride or triethylamine trihydrofluoride) and/or multistep purification (ion-exchange, and reversed-phase chromatography)^9,13,16,39–41^. The multistep deprotection is dictated by the need to protect 2′-hydroxyl group(s) neighbouring the phosphodiester linkage(s), with tert-butyldimethylsilyl (TBDMS) or other silicon-based protecting groups to prevent 3′-to-2′ phosphoryl migration or phosphodiester bond cleavage. However, the complex processing leads to reduced isolated yields, and the fluorides used in the process exhibit toxicity. We anticipated that the 2′-hydroxyl group of the first transcribed nucleotide would not be engaged in translation-related interactions, so in our design, we introduced the 2′-*O*-methyl modification to permanently block it. We achieved this by using *O*^5′^-dimethoxytrityl-*N*^6^-phenoxyacetyl-protected 2′-*O*-methyl-A phosphoramidite for coupling with support-bound *O*^5′^-dimethoxytrityl-*N*^2^-isobutyryl-*O*^2′^-TBDMS-protected RNA G in a standard oligonucleotide synthesis cycle. To modify the 5′-hydroxyl group, we employed a 5′-hydroxyl-to-iodide and 5′-iodide-to-azide conversion procedure^42,43^. Finally, we carried out deprotection and cleavage in aqueous 35% ammonia overnight at 55 °C, which was sufficient to fully remove the TBDMS group without causing degradation (Fig. 2c). These decisions enabled us to simplify the process, including minimisation of the purification to one-step reversed-phase chromatography, which gave 54% isolated yield of the CleaN3 on a 75 µmol reaction scale.

### IVT priming with CleaN3 provides homogeneous 5′-azido-modified transcripts

**Fig. 3:**
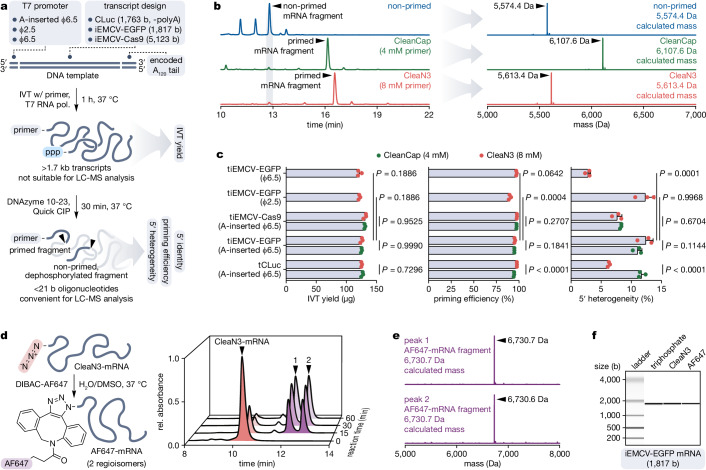
Priming in vitro transcription with CleaN3 and post-transcriptional modification of the transcripts. **a** DNA template design and workflow for studying in vitro transcription (IVT) priming. We carried out the IVT reactions using T7 RNA polymerase and CleaN3 or CleanCap as the IVT primer. To analyse priming efficiency, 5′-heterogeneity, and 5′-identity of the transcripts via LC-MS, we trimmed the RNAs using DNAzyme 10–23. We additionally used calf intestinal alkaline phosphatase (Quick CIP) that removes 5′-triphosphates to improve separation between primed and non-primed fragments. **b** Left, representative HPLC chromatograms (absorbance at 260 nm, traces normalised to maximum) of the fragments of tiEMCV-EGFP (with A-inserted φ6.5 promoter)-templated transcripts primed with CleaN3 or CleanCap and non-primed. Right, representative mass spectra (deconvolved, normalised to maximum) of fragments of the main (by area of absorbance peak) products of IVTs, together with masses calculated for anticipated product fragments. **c** IVT yields (in the amount of produced mRNA), priming efficiencies, and transcript 5′-heterogeneities from IVTs primed with CleaN3 or CleanCap. The bar graphs show individual data points and the means ± s.d. of three experimental replicates. Statistical significance was calculated using two-way ANOVA followed by Šídák’s multiple comparisons test (comparisons of IVT primers for different DNA templates with A-inserted φ6.5 promoter) and one-way ANOVA followed by Dunnett’s multiple comparisons test (comparisons of T7 promoters for IVTs primed with CleaN3). **d** To assess CleaN3-primed mRNA susceptibility to post-transcriptional modification, we reacted it with AF647 dye functionalised with dibenzoazacyclooctyne (DIBAC). The HPLC chromatograms stack (relative absorbance at 260 nm, corrected by normalising to total trace area and scaled to maximum absorbance at reaction time 0) shows the progress of post-transcriptional modification of CleaN3-primed iEMCV-EGFP mRNA with DIBAC-AF647. **e** Mass spectra (deconvolved, normalised to maximum) of two fractions marked in panel (**d**) (trimmed with DNAzyme 10–23), together with masses calculated for anticipated reaction product fragments. **f** Pseudo-gel image of 5′-triphosphate, 5′-CleaN3, and 5′-AF647 iEMCV-EGFP mRNAs. Data of one replicate. Source data are provided as a Source Data file.

To evaluate the performance of CleaN3 in IVT priming mediated by T7 RNA polymerase, we prepared a series of DNA templates with various lengths of encoded transcripts (1.8–5.1 kb), T7 promoters (A-inserted φ6.5, φ2.5, φ6.5), and optionally encoded poly(A) tail (Fig. 3a, Supplementary Fig. 1). While the standard approach for benchmarking IVT primers involves DNAzyme 10–23 cleavage of transcripts and subsequent denaturing PAGE analysis^13,16,20,39,41,44^, it did not yield sufficient separation of fragments to assess the CleaN3 performance (Supplementary Fig. 2a). Therefore, we introduced an additional 5′-triphosphate removal alongside the DNAzyme 10–23 cleavage and performed the subsequent analysis using LC-MS (Fig. 3a). This demonstrated superior resolution and enabled confirmation of the RNA fragment identities (Fig. 3b). IVTs with varying CleaN3 concentrations (2–10 mM) showed its successful incorporation into transcripts even at 2 mM, with 88.9% priming efficiency, which improved to 93.2% at 4 mM and up to 97.2% at 10 mM (Supplementary Fig. 2b). Across all tested concentrations the transcript yield remained comparable (119.3–121.7 µg; 20 µL reaction). When used at 8 mM, CleaN3 matched the performance of CleanCap (4 mM, per the manufacturer’s optimised protocol) in IVTs with short (1.8 kbp) and long (5.1 kbp) templates with A-inserted φ6.5 promoter (Fig. 3c). Compared to non-primed transcripts, their CleaN3-primed counterparts showed greatly reduced 5′-heterogeneity (Supplementary Fig. 2c,d). Finally, CleaN3 showed an excellent performance with φ6.5 promoter (comparable yield and priming efficiency, reduced 5′-heterogeneity) and fair compatibility with φ2.5 promoter (comparable yield and 5′-heterogeneity, slightly reduced priming efficiency) – both compared to A-inserted φ6.5 promoter (Fig. 3c). Together, these results demonstrate that IVT priming with CleaN3 enables highly efficient and specific synthesis of 5′-azido-modified transcripts across a broad range of primer concentrations, DNA template lengths, and T7 promoters.

### CleaN3-primed transcripts can be efficiently modified post-transcriptionally

Next, we assessed the susceptibility of CleaN3-primed iEMCV-EGFP mRNA to post-transcriptional modification utilising SPAAC by reacting it with AF647 dye functionalised with dibenzoazacyclooctyne (DIBAC) (Fig. 3d). To maximise the reaction rate, we used a high mRNA concentration (29.3 µg/µL), a 100-fold molar excess of DIBAC-AF647, and mild heating (37 °C). Time-course ion-pair reversed-phase (IPRP) HPLC analysis revealed excellent separation of the product from the starting material and fast reaction progress (70% conversion in 15 min, full conversion in 1 h). Interestingly, the product gave two, well-separated peaks, which we suspected to be two regioisomers as SPAAC is not regiospecific. To verify this and to confirm that the additional peak does not correspond to an unexpected by-product, we collected the two fractions, trimmed them with DNAzyme 10–23 and analysed the fragments using MS (Fig. 3e). The analysis showed similar masses for both fragments, which agree with expected product mass. Further, as DIBAC-AF647 is a mixture of two diastereomers, we reacted 5′‑CleaN3 mRNA with the isolated dye diastereomers and confirmed that the two conjugate peaks do not arise from dye isomerism (Supplementary Fig. 3). Finally, we assessed if the modification affects the integrity of the transcripts by comparing 5′-AF647 iEMCV-EGFP mRNA to its 5′-triphosphate and 5′-CleaN3 counterparts using on-chip electrophoresis (Fig. 3f). The analysis indicated no significant difference in integrity between the 5′-variants. Overall, CleaN3-primed transcripts can be readily and efficiently post-transcriptionally modified via SPAAC without affecting their integrity and the 5′-modified products can be easily separated from non-primed and non-reacted material.

### Post-transcriptional 5′-modification of CIT mRNA can significantly enhance its properties

Although 5′-triphosphate protects linear CIT mRNAs from direct degradation by 5′ → 3′ exoribonucleases like XRN-1, it can be hydrolysed to monophosphate by NUDT2 to trigger the decay^45^. Moreover, 5′-triphosphate is immunogenic, inducing cytokine production and suppressing translation^46^. It can be removed post-transcriptionally but at the cost of mRNA stability^47^. These obstacles hamper linear CIT mRNAs from advancing to clinical applications. Therefore, we wondered whether installing a potentially non-removable blocker like AF647 at the 5′-end would overcome these obstacles and consequently improve the properties of CIT mRNAs.

**Fig. 4:**
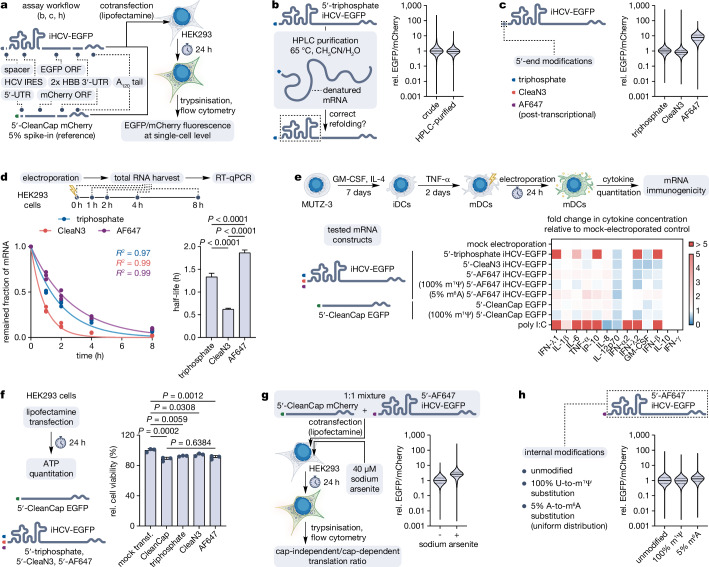
Evaluation of properties of modified CIT mRNAs. **a** Workflow for evaluating CIT mRNA properties. **b** Influence of the HPLC purification on the 5′-triphosphate iHCV-EGFP mRNA translation efficiency (relative to crude). *n* = 28,821 (crude) and *n* = 23,365 (HPLC-purified) pooled single cells from three experimental replicates. **c** Influence of 5′-modifications on the iHCV-EGFP mRNA translation efficiency (relative to triphosphate). *n* = 31,789 (triphosphate), *n* = 31,446 (CleaN3), and *n* = 31,094 (AF647) pooled single cells from three experimental replicates. **d** Stability of 5′-triphosphate, 5′-CleaN3, and 5′-AF647 iHCV-EGFP mRNAs in electroporated HEK293 cells. Left, remaining fractions of the mRNAs over time as individual data points of three experimental replicates and curves obtained by fitting a one-phase decay model to the data using the least squares method. Right, calculated half-lives based on the one-phase decay fit as means ± s.e. Statistical significance was calculated using one-way ANOVA followed by Tukey’s multiple comparisons test. **e** Immunogenicity of mRNAs in MUTZ-3-derived mature dendritic cells. The heatmap shows fold changes in cytokine concentration in the medium relative to mock-electroporated control as means of three experimental replicates. Additional data, including statistical analysis, are shown in Supplementary Fig. 5. **f** Viability of HEK293 cells transfected with 5′-triphosphate, 5′-CleaN3, 5′-AF647 iHCV-EGFP, or 5′-CleanCap EGFP mRNA, 24 h after transfection (relative to mock-transfected control). The graph shows individual data points and the means ± s.d. of three experimental replicates. Statistical significance was calculated using one-way ANOVA followed by Šídák’s multiple comparisons test. **g** Influence of the arsenite stress on the cap-independent/cap-dependent translation ratio (relative to - arsenite). *n* = 29,866 (- arsenite) and *n* = 17,560 ( + arsenite) pooled single cells from three experimental replicates. **h** Influence of internal modifications on the 5′-AF647 iHCV-EGFP mRNA translation efficiency (relative to unmodified). *n* = 29,508 (unmodified), *n* = 28,383 (100% m^1^Ψ), and *n* = 28,987 (5% m^6^A) pooled single cells from three experimental replicates. **b, c, g, h** All violin plots show data distribution, median (solid line), and first and third quartile (dashed lines). Gating strategies and dot plots for all flow cytometry data are shown in Supplementary Fig. 4. Source data are provided as a Source Data file.

To answer this, we established a translation efficiency assessment workflow with iHCV-EGFP (containing Hepatitis C virus IRES) as a model CIT mRNA design (Fig. 4a, Supplementary Fig. 1). As the IRES-mediated ribosome recruitment relies on the mRNA secondary structure, we first confirmed that the HPLC purification in denaturing conditions that is integral to our post-transcriptional 5′-modification protocol does not affect the CIT mRNA translational properties (Fig. 4b, Supplementary Fig. 4a, b). Next, we tested the influence of the 5′-AF647 modification on the translational properties of the CIT mRNA (Fig. 4c, Supplementary Fig. 4a, c). The 5′-AF647 construct demonstrated a 7.6-fold greater translation efficiency compared to the 5′-triphosphate version. The 5′-CleaN3 mRNA showed, on the other hand, a 1.2-fold lower performance than its 5′-triphosphate counterpart. These results demonstrate that post-transcriptional 5′-modification of CIT mRNA can enhance its translational properties.

To determine whether the improved translation of the 5′-AF647 mRNA could arise from its stabilisation, we assessed the stability of all the 5′-variants in electroporated HEK293 cells via RT-qPCR (Fig. 4d). While 5′-CleaN3 mRNA was 2.2-fold less stable than the 5′-triphosphate version, 5′-AF647 modification increased the stability 1.4-fold over 5′-triphosphate and 3.0-fold over 5′-CleaN3. These data suggest that the augmented translation efficiency of the 5′-AF647 mRNA can be conferred by its improved stability.

Next, we asked whether the 5′-modifications can address the immunogenicity issue of linear CIT mRNAs. To this end, we screened all the 5′-variants for inducing secretion of viral infection-related cytokines in transfected MUTZ-3-derived mature dendritic cells (Fig. 4e, Supplementary Fig. 5). As controls we used poly I:C and 5′-CleanCap EGFP mRNA without IRES (without and with U-to-m^1^Ψ substitutions known to prevent activation of toll-like and RIG-I-like receptors^48,49^). For 5′-triphosphate iHCV-EGFP mRNA we observed strong induction of IFN-λ1, IL-6, IP-10, IFN- λ2, and IFN-β (linked to RIG-I activation). In contrast, cytokine levels for the modified versions – 5′-CleaN3, 5′-AF647, as well as 5′-AF647 with 100% m^1^Ψ or 5% m^6^A modifications were comparable to 5′-CleanCap EGFP and its 100% m^1^Ψ counterpart. These results indicate that introducing modifications to the 5′-end of transcripts, either by IVT priming with CleaN3 or subsequent post-transcriptional modification with DIBAC-AF647 reduces the immunogenicity of CIT mRNA.

As modifications like 5′-CleaN3 or 5′-AF647 do not exist naturally in mRNAs, we tested whether they interfere with cellular processes by assessing the viability of the transfected HEK293 cells (Fig. 4f). All the tested mRNAs caused a slight reduction of cell viability compared to the mock-transfection (e.g., 91 ± 2% for 5′-AF647 mRNA). The difference between the 5′-AF647 iHCV-EGFP and 5′-CleanCap EGFP mRNAs was, however, not significant. This suggests that the slightly decreased viability is due to overexpression of EGFP and indicates no influence of IVT priming with CleaN3 or post-transcriptional 5′-AF647 modification on cell viability.

We next asked if the 5′-AF647 CIT mRNA is more resistant to suppression of translation under cellular stress than capped mRNA. To this end we cotransfected HEK293 cells with 1:1 mixture of 5′-AF647 iHCV-EGFP and 5′-CleanCap mCherry mRNAs and measured the EGFP/mCherry fluorescence ratio under normal and arsenite stress conditions (Fig. 4g, Supplementary Fig. 4 d). In cells exposed to stress conditions the EGFP/mCherry ratio increased 2.6-fold compared to normal conditions. This indicates that 5′-AF647 CIT mRNA is more resistant to the suppression of translation under cellular stress than the cap-dependently translated mRNA.

Finally, we investigated whether internal modifications (100% m^1^Ψ and 5% m^6^A), shown to improve translation of capped mRNAs^50^, are compatible with the 5′-AF647 CIT mRNA and if they can further enhance its translational properties (Fig. 4h, Supplementary Fig. 4e). While the 100% m^1^Ψ version showed comparable translation efficiency to the unmodified version, the 5% m^6^A modification resulted in its slight (1.3-fold) improvement. These data indicate that both m^1^Ψ and m^6^A internal modifications are compatible with CIT mRNA and that m^6^A can slightly augment its translational properties.

### Post-transcriptional 5′-modification can serve as a tool for benchmarking CIT mRNAs

Due to differences in cell-specific proteomes, mRNA uptake, processing, and translation efficiency can substantially vary among different cell types. Thus, to accurately compare the translational properties of CIT mRNAs between cell lines, a data normalisation strategy is essential. Currently, a prevalent approach for that involves employing dual-reporter, bicistronic mRNAs, wherein the initial cap-dependently translated cistron acts as a reference for IRES-mediated translation of the subsequent cistron^51–55^. However, as the normalisation reference in this system is sensitive to variability in translation factors^56^, using it to compare translational properties of mRNAs in different cell types can lead to inaccuracies. The fluorescence of a fluorophore, on the other hand, is independent of cell-specific factors. Given this, CleaN3-priming and post-transcriptional fluorescent labelling of mRNAs could be an attractive alternative enabling normalisation of the translation to the total mRNA uptake.

**Fig. 5:**
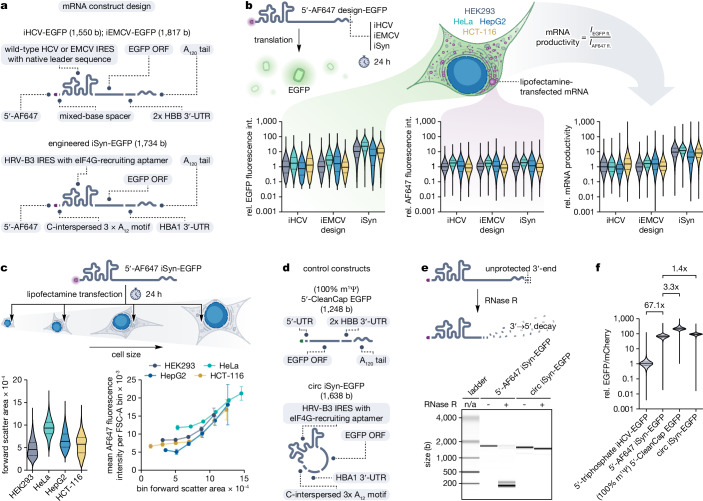
Benchmarking 5′-modified CIT mRNAs using flow cytometry. **a** Design of 5′-AF647 CIT mRNAs used in the benchmark. **b** CIT mRNA benchmark: EGFP expression (left), mRNA uptake (AF647 fluorescence intensity; middle), and mRNA productivity (EGFP/AF647 fluorescence intensity ratio; right). All values are relative to 5′-AF647 iHCV-EGFP mRNA in HEK293 cells. For all three plots: *n* = 17,310 (iHCV, HEK293), *n* = 32,494 (iHCV, HeLa), *n* = 8,290 (iHCV, HepG2), *n* = 23,673 (iHCV, HCT-116), *n* = 20,385 (iEMCV, HEK293), *n* = 33,026 (iEMCV, HeLa), *n* = 8,975 (iEMCV, HepG2), *n* = 26,190 (iEMCV, HCT-116), *n* = 17,708 (iSyn, HEK293), *n* = 33,516 (iSyn, HeLa), *n* = 9,084 (iSyn, HepG2), and *n* = 24,566 (iSyn, HCT-116) pooled single cells from three experimental replicates. **c** Dependence of the mRNA uptake on cell size within the individual cell lines transfected with 5′-AF647 iSyn-EGFP mRNA. Left, violin plots of cell forward scatter areas (FSC-A). *n* = 29,476 (HEK293), *n* = 33,601 (HeLa), *n* = 18,930 (HepG2), and *n* = 32,728 (HCT-116) pooled single cells from three experimental replicates. Right, relation between AF647 fluorescence intensities (mRNA uptake) and FSC-A of the cells binned according to increasing FSC-A as bin means ± s.d. of three experimental replicates. FSC-A binning strategies are shown in Supplementary Fig. 8b. **d** Design of 5′-CleanCap EGFP and circ iSyn-EGFP control mRNAs. **e** RNase R digest of 5′-AF647 iSyn-EGFP and circ iSyn-EGFP mRNAs. Data of one replicate. **f** Translational properties of the highest-performing CIT mRNA (5′-AF647 iSyn-EGFP) compared to the unmodified CIT mRNA (5′-triphosphate iHCV-EGFP), capped mRNA (100% m^1^Ψ, 5′-CleanCap EGFP), and circular RNA (circ iSyn-EGFP) in HEK293 cells. Reported values are relative to 5′-triphosphate iHCV-EGFP. *n* = 31,118 (5′-triphosphate iHCV-EGFP), *n* = 30,957 (5′-AF647 iSyn-EGFP), *n* = 29,302 (5′-CleanCap EGFP), and *n* = 30,728 (circ iSyn-EGFP) pooled single cells from three experimental replicates. The assay workflow is shown in Fig. 4a. **b, c, f** All violin plots show data distribution, median (solid line), and first and third quartile (dashed lines). Gating strategies and dot plots for all flow cytometry data are shown in Supplementary Figs. 6 and 4a,f. Source data are provided as a Source Data file.

To explore this approach, we prepared three linear CIT mRNAs with 5′-AF647 modification (Fig. 5a, Supplementary Fig. 1) and benchmarked them in four human cell lines (Fig. 5b, Supplementary Fig. 6). Two of these mRNAs contained wild-type viral IRESs, whereas the third was an HRV-B3 IRES-based synthetic version derived from circRNA engineered for enhanced protein production^57^, incorporating two poly(A) tails (5′ 3 × C-interspersed A_12_ and 3′ A_120_) in its linear form. Analysis of the sole EGFP expression showed the highest fluorescence intensity for HeLa cells across all mRNA designs, which could suggest that in this cell line, all the CIT mRNAs undergo translation the most efficiently. However, the mRNA uptake indicated by the AF647 fluorescence was also, in all cases, the highest for HeLa. Performing data normalisation and comparing the mRNA productivity (defined as the EGFP/AF647 ratio) revealed that only the iSyn mRNA was the most efficiently translated in HeLa. The iHCV construct demonstrated peak translation efficiency in HCT-116 cells, while the iEMCV construct performed similarly well in both HeLa and HepG2 cells. To further verify that the observed differences are not affected by saturating the translational machinery of the cells, we analysed dose-response relationships (Supplementary Figs. 7 and 8a). For all the analysed cell lines, the mRNA concentrations were within the cells’ translational capacities. Collectively, these results shed light on cell line-specific differences in CIT mRNA productivity and underline the 5′-modification strategy as an attractive solution for the normalisation of the translation data.

Next, as the post-transcriptionally modified 5′-AF647 mRNAs, in contrast to bicistronic constructs, allow for determining their quantity in cells, we wanted to gain insights into the factors behind observed differences in uptake and productivity. To this end, we analysed the cell sizes and their relationship with the amount of transfected mRNA (Fig. 5c, Supplementary Fig. 8b). HeLa cells were significantly larger than the cells belonging to the other three cell lines. This could suggest that the highest EGFP expression and mRNA uptake observed for this cell line is related to its size; however, there was no similar correlation for the other three cell lines. As other cell line-specific factors, besides the cell size, can contribute to the mRNA uptake, to rule them out, we binned the cells within all the individual cell lines according to increasing size. This showed that the transfected mRNA amount increases together with the cell size. To test if the inverse relationship occurs, we compared cell sizes for untreated and mRNA-transfected cells (Supplementary Fig. 9). For HEK293, we observed a slight (15.8%) increment in size related to transfection, but for the other cell lines, the forward scatter areas of untreated and mRNA-transfected cells were comparable. These data show broader applicability of the 5′-modification strategy and suggest that the cell size influences mRNA uptake and translation, but other cell type-specific factors have a stronger effect.

Finally, as the benchmark (Fig. 5b) revealed the iSyn-EGFP mRNA as the highest performing design across all cell lines, we wondered how efficient its translation is compared to a standard non-modified version and translation-optimised capped and circular RNAs (Fig. 5d-f, Supplementary Figs. 1 and 4f). The design of the used circRNA, originally reported by Chen et al. ^57^., was similar to the linear 5′-AF647 version (Fig. 5d). However, thanks to its circular topology, it conferred resistance to not only 5′ → 3′, but also 3′ → 5′ exoribonucleolytic decay, in contrast to its linear counterpart (Fig. 5e). In the transfected HEK293 cells, the 5′-AF647 iSyn-EGFP mRNA showed 67.1-fold improved translation efficiency over the standard, non-modified 5′-triphosphate iHCV-EGFP transcript (Fig. 5f). Its translational performance was close to the circ iSyn-EGFP RNA (1.4-fold difference) and 5′-CleanCap, 100% m^1^Ψ EGFP mRNA (3.3-fold difference). These results show that the post-transcriptionally modified 5′-AF647 CIT mRNA with optimised sequence design can confer comparable translational performance to capped and circular RNAs.

### Post-transcriptional 5′-modification facilitates cellular fate tracking of CIT mRNA

Encouraged by the applicability of the 5′-modification strategy in benchmarking CIT transcripts, we sought to explore its suitability for spatiotemporal characterisation of CIT mRNA cellular fate from transfection to IRES-mediated translation.

**Fig. 6:**
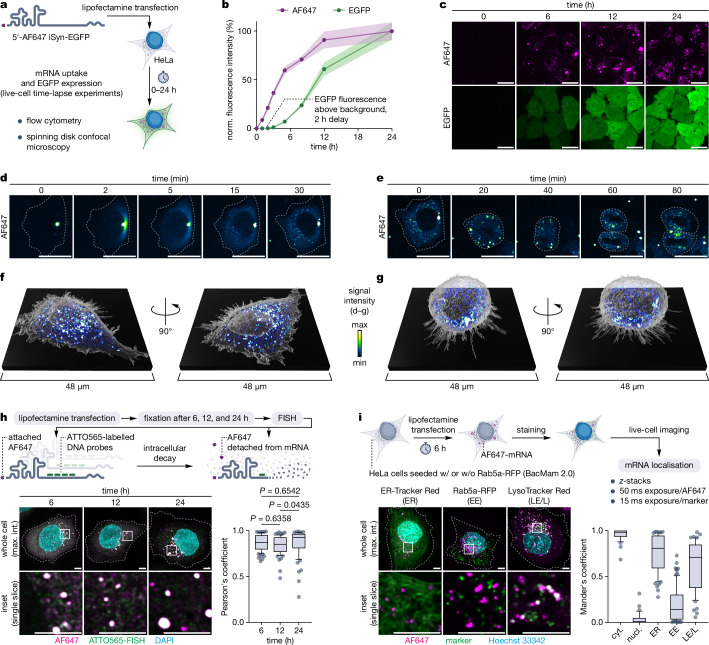
Transfection and cellular fate tracking of 5′-AF647 iSyn-EGFP mRNA. **a** mRNA uptake and EGFP expression time-lapse experiments workflow. **b** Tracking lipofectamine-mediated mRNA delivery and EGFP expression using flow cytometry. The graph shows changes in average cellular AF647 and EGFP fluorescence intensities (normalised to maximum) over time as means ± 95% c.i. of three experimental replicates. Gating strategies and dot plots are shown in Supplementary Figs. 6 and 10a, respectively. **c** Representative images of HeLa cells incubated with lipofectamine-formulated mRNA showing changes in AF647 and EGFP fluorescence intensities over time. Scale bars, 25 μm. **d** Representative images showing the fate of a single lipoplex during lipofectamine-mediated mRNA delivery to HeLa cell over time. Scale bars, 25 μm. **e** Representative images showing the fate of the delivered mRNA during the division of HeLa cell over time. Scale bars, 25 μm. **f** Live-cell 3D image of transfected HeLa cell. **g** Live-cell 3D image of transfected, dividing HeLa cell. **h** colocalisation of the AF647 dye and iSyn-EGFP mRNA in fixed HeLa cells. Left, Representative images showing colocalisation of the AF647 with FISH probes over time. Control experiments for the FISH probes specificity are shown in Supplementary Fig. 10b. Right, Pearson’s correlation coefficients of AF647 and ATTO565-FISH. *n* = 71 (6 h), *n* = 65 (12 h), and *n* = 69 (24 h) pooled *z* slices spanning the whole cell volumes from three distinct cells per time point. Statistical significance was calculated using Kruskal-Wallis test followed by Dunn’s multiple comparisons test. **i** mRNA cooccurrence with cytoplasm, nucleus, endoplasmic reticulum (ER), early endosomes (EE), and late endosomes/lysosomes (LE/L). Left, Representative cooccurrence images. Right, Mander’s overlap coefficients. *n* = 39 (cyt.), *n* = 39 (nucl.), *n* = 61 (ER), *n* = 86 (EE), and *n* = 55 (LE/L) pooled *z* slices spanning the whole cell component volumes from three distinct cells per component. Representative thresholded masks are shown in Supplementary Fig. 10c. **h, i** Cell segmentation based on the edge of the EGFP fluorescence. Scale bars, 5 μm. Box plots show median (line), first and third quartile (box), 10–90 percentile (whiskers), and outliers (points). Source data are provided as a Source Data file.

First, we tracked the lipofectamine-mediated transfection of the 5′-AF647 iSyn-EGFP mRNA and its expression in HeLa cells over time using flow cytometry and spinning disk confocal microscopy (Fig. 6a). The flow cytometry (Fig. 6b, Supplementary Fig. 10a) consistently with the microscopy (Fig. 6c, Supplementary Movie 1) data showed rapid mRNA accumulation in cells immediately upon addition of the lipoplex solution and its slowdown between 6 and 24 h. The EGFP was expressed with 2 h delay, localising in the entire cell volume. For both AF647 and EGFP, the fluorescence was intense enough to allow for image acquisition at low laser power, which prevented cell death and noticeable photobleaching even during 24 h time-lapse imaging with 5-minute temporal resolution. Overall, the post-transcriptional 5′-AF647 modification of the mRNA enables the characterisation of its uptake and expression using flow cytometry and microscopy.

To further explore the potential of the 5′-modification in super-resolution live-cell imaging, we studied the mRNA uptake in more detail. First, we captured a single transfection event, collecting a new set of images with a 1 min temporal resolution (Fig. 6d, Supplementary Movie 2). The images revealed a multi-stage characteristic of the process. First, the observed lipoplex (diameter 1.3 ± 0.3 μm, *n* = 3) started slowly fusing with the cell membrane, causing diffusion of the mRNA into the cytoplasm (but not the nucleus). After 5.7 ± 0.5 min (*n* = 3) from the cell entry, the diffused mRNA started concentrating in multiple foci of diameters below 600 nm. Another set of images, collected at increased magnification (tile size 69.3 × 69.3 μm) with 100-millisecond temporal resolution, revealed their rapid movement (but slower than this of the diffused, non-concentrated in foci mRNA molecules) in the cytoplasm (Supplementary Movie 3). During cell division, the mRNA was equally split between the two daughter cells (Fig. 6e, Supplementary Movie 4). We further performed super-resolution 3D live-cell imaging of single, transfected, non-dividing (Fig. 6f, Supplementary Movie 5) and dividing (Fig. 6g, Supplementary Movie 6) cells that was enabled by rapid (80 and 70 ms exposure for AF647 and EGFP, respectively) collection of *z*-stacks. This revealed an increased concentration of the mRNA foci close to the nucleus in the non-dividing cell and their more even distribution in the dividing cell. In all the experiments, the 5′-AF647 iSyn-EGFP mRNA displayed excellent fluorescence intensities of AF647 and expressed EGFP that enabled image acquisition with low background, minimal photobleaching, no cellular damage, and with very short exposure times – essential for the 3D live-cell and real-time time-lapse imaging. Collectively, these data indicate excellent performance of the post-transcriptionally 5′-modified transcript in super-resolution live-cell imaging and suitability of the fluorescent labelling method for spatiotemporal characterisation of the mRNA cellular fate.

Because the fluorescent dye detaches upon the intracellular decay of the modified transcript, we next asked how well its fluorescence signal correlates with the actual localisation of the mRNA over time. To assess it, we performed fluorescence in situ hybridisation (FISH) with EGFP sequence-specific (Supplementary Fig. 10b) probes in HeLa cells fixed 6, 12, and 24 h post-transfection (Fig. 6h). Pearson’s coefficients indicated an excellent correlation between the intracellular localisation of AF647 and mRNA at all time points. However, while at 24 h, accumulation of the intact mRNA in large foci (above 300 nm) with high fluorescence intensity of both AF647 and FISH probes manifested in slightly elevated Pearson’s coefficient, small foci (below 300 nm) with low fluorescence intensity were mostly enriched with detached AF647. These results indicate that AF647 fluorescence of the 5′-modified mRNA accurately reflects mRNA localisation for both small and large mRNA-containing bodies, particularly within short times (6–12 h) post-transfection.

Finally, we explored the applicability of the 5′-AF647 iSyn-EGFP mRNA in more detailed localisation studies by analysing its cooccurrence with cellular components in live cells (Fig. 6i, Supplementary Fig. 10c). To ensure no relocation of the stained organelles and mRNA during acquiring their images, we used short (50 ms for AF647 and 15 ms for markers) exposure times. Yet, the 5′-AF647-modified mRNA exhibited excellent signal intensity that enabled precise thresholding. 6 h from transfection, the mRNA was localised in the cytoplasm. The cytoplasmic bodies that contained accumulated mRNA predominantly associated with the endoplasmic reticulum were mostly late endosomes and lysosomes, with a smaller fraction being early endosomes. These results demonstrate good compatibility of the post-transcriptionally 5′-modified mRNA with localisation studies involving live-cell imaging and shed light on the identity of the cytoplasmic bodies that are the destination of the transfected transcripts.

## Discussion

The mRNA platform achieved extraordinary success during the COVID-19 pandemic; however, expanding its scope beyond vaccines requires new tools enabling the improvement of transcript properties^1,2^. Here, we have shown that IVT priming, a strategy typically used for 5′-capping, can be harnessed to enhance cap-independent translation of mRNA by improving CIT transcript properties and facilitating refinement of elements promoting cap-independent translation.

Our approach utilises CleaN3 – an azido-functionalised dinucleotide IVT primer, which we have efficiently synthesised, overcoming burdens related to previous methods, like the use of fluorides or multistep purification^9,13,16,39,40^. The simplicity and efficiency of the synthesis make it accessible to virtually any laboratory and allow it to be automated for industrial production. Thanks to the CleaN3 design relying on dinucleotide base-pairing with T7 promoters, it gives superior IVT yields, priming efficiency, and primed transcript homogeneity, which were not attainable with previous IVT primers enabling post-transcriptional modifications^17–21^. The 5′-azide, present in CleaN3-primed transcripts, provides access to rapid and efficient post-transcriptional modification via SPAAC, making it possible within one working day to synthesise 5′-modified transcripts starting from nucleoside triphosphates. The CIT mRNA, which we have conjugated with AF647, has improved translational properties due to its 5′-stabilisation and reduced immunogenicity. As this improvement arises from a chemical modification of the mRNA, our strategy is orthogonal to sequence-based engineering approaches^35^. Although we showcased the 5′-modification strategy with only one model molecule (AF647), our approach creates myriad possibilities for conjugation with different commercially available or easy-to-prepare alkynes, which can be utilised in the future to fine-tune CIT mRNA properties.

Despite rising concerns regarding the use of bicistronic constructs in the discovery and characterisation of elements promoting cap-independent translation^36–38^, little has been done to find a better alternative, and the method is still being commonly used, even for comparing translation between different cell types^51–55^. Our approach addresses these concerns by enabling a normalisation strategy independent of cell line-specific factors. Moreover, it provides access to detailed spatiotemporal characterisation of CIT mRNA uptake and cellular fate, which is not possible with the bicistronic constructs. These advancements will enable recharacterisation of known elements promoting cap-independent translation and will facilitate the discovery of new ones.

Although displaying advantages such as improved resistance to translation suppression under cellular stress (compared to capped mRNAs) and high synthesis yield (compared to circRNAs), linear CIT constructs have generally been overlooked in therapeutic mRNA design. This neglect stems from their poor translational performance tied to the conservative design that has lacked innovation over the years. Our results demonstrated that combining the post-transcriptional 5′-modification, enabled by CleaN3, with sequence optimisation can significantly improve the properties of the linear CIT mRNAs. As a result, these modifications render them a viable alternative to circRNAs and capped mRNAs. Moreover, since our modified CIT mRNA was inspired by a design originally optimised for circRNA, we envision that developing sequence variants better suited to the linear version and potentially conferring even higher translational performance is feasible. By leveraging our 5′‑modification method for construct benchmarking, we believe such sequence-optimised CIT mRNA variants can be systematically studied in the future.

Overall, thanks to its simplicity and immediate applicability, the approach we have developed here will stimulate exploration and expedite advances in cap-independent translation and the characterisation of elements promoting cap-independent translation. These strides can propel CIT mRNAs closer to clinical applications.

## Methods

Supplementary Figs., list of manufacturer’s protocols, nucleic acid sequences, and NMR and MS spectra are provided in Supplementary Information. *pRSET-IRES-EGFP* (plasmid #216151, https://www.addgene.org/216151/), *pmRNA-EGFP-A120* (plasmid #225903, https://www.addgene.org/225903/), *pmRNA-mCherry-A120* (plasmid #225904, https://www.addgene.org/225904/), *pmRNA-iEMCV-EGFP-A120* (plasmid #225905, https://www.addgene.org/225905/), *pmRNA-iHCV-EGFP-A120* (plasmid #225906, https://www.addgene.org/225906/), and *pmRNA-iSyn-EGFP* (plasmid #225907, https://www.addgene.org/225907/) plasmids have been deposited with Addgene.

### Statistics & reproducibility

Information on study design, sample sizes, and statistical tests used is provided in figure legends and/or protocols listed in the Methods. No statistical method was used to predetermine sample size. No data were excluded from the analyses. The experiments were not randomised. The Investigators were not blinded to allocation during experiments and outcome assessment.

### General information

All reagents were purchased from Sigma-Aldrich, Merck, Fluorochem, VWR, Applied Biosystems, Link Technologies, Serva, ATTO-TEC, Jena Bioscience, Thermo Fisher Scientific or LGC Biosearch Technologies and, if not stated otherwise, used without any pre-treatment. All enzymes and kits were purchased from New England Biolabs, Thermo Fisher, Promega, Lonza, BioLegend, Omega Bio-tek, LGC Biosearch Technologies, and Agilent Technologies. Catalogue numbers of used reagents and kits are provided in the experimental procedures. *pRSET-B mCherry* plasmid was a gift from Kalina Hristova (Addgene plasmid #108857; http://n2t.net/addgene:108857; RRID: Addgene_108857)^58^. *pCAG-EOMES-EGFP-IRES-Puro* plasmid was a gift from Elizabeth J. Robertson (Sir William Dunn School of Pathology, University of Oxford). *pRNA2-(A)128* plasmid was a gift from Stephen Ikeda (Addgene plasmid #174006; http://n2t.net/addgene:174006; RRID: Addgene_174006)^59^. *pmCherry-N1 GBP (GBP-mCherry)* plasmid was a gift from Frederic Meunier (Addgene plasmid #162879; http://n2t.net/addgene:162879; RRID: Addgene_162879)^60^. *pFR_HCV_xb* plasmid was a gift from Phil Sharp (Addgene plasmid #11510; http://n2t.net/addgene:11510; RRID: Addgene_11510)^61^. *pMCS-rybozyme-IRES-CAS9* plasmid was a gift from Wataru Fujii (Addgene plasmid #64668; http://n2t.net/addgene:64668; RRID: Addgene_64668)^62^. *circRNA-synIRES-R25-mNeonGreen* plasmid was a gift from Howard Chang (Addgene plasmid #188115; http://n2t.net/addgene:188115; RRID: Addgene_188115)^57^. HepG2 cells (ATCC #HB-8065) were purchased from ATCC. MUTZ-3 cells (DSMZ #ACC 295) were purchased from DSMZ. HEK293 cells (ATCC #CRL-1573) were a gift from Elizabeth J. Robertson (Sir William Dunn School of Pathology, University of Oxford). HeLa (ATCC #CCL-2) and HCT-116 (ATCC #CCL-247) cells were a gift from Katherine A. Vallis (Oxford Institute for Radiation Oncology, Department of Oncology, University of Oxford). All cell lines used in this study were authenticated by ECACC. Type I water used in all experiments was produced using Synergy® coupled to RiOs™ 3 Water Purification System (Merck).

### HPLC

Automated high-performance liquid chromatography separations were carried out on an Agilent 1260 Infinity instrument equipped with an autosampler, a column oven, a diode array detector and a fraction collector. For instrument control, data acquisition and data processing, OpenLab CDS 2.7 (Agilent Technologies) software was used. Reversed-phase separations of CleaN3 were carried out on Phenomenex Kinetex® EVO C18, 5 µm, 100 Å, 250 × 10.0 mm column (mobile phase A: 50 mM ammonium acetate, pH 5.9 in water, mobile phase B: acetonitrile, linear gradient: 0–80% B over 12 min, 80–100% B over 1 min, then 100% B for 2 min, temperature 25 °C, flow rate 5 mL/min, detection at 260 nm). Reversed-phase separations of DIBAC-AF647 were carried out on Phenomenex Gemini NX-C18, 3 μm, 110 Å, 150 × 4.6 mm column (mobile phase: 50 mM ammonium acetate, pH 5.9 in water/acetonitrile (82:18), isocratic elution, 20 min, temperature 20 °C, flow rate 2 mL/min, detection at 650 nm). Ion-pairing reversed-phase separations of oligonucleotides were carried out on Phenomenex Kinetex® EVO C18, 5 µm, 100 Å, 250 × 10.0 mm column (mobile phase A: 100 mM triethylammonium acetate, pH 7.0 in water, mobile phase B: 100 mM triethylammonium acetate, pH 7.0 in water/acetonitrile 1:1 (v/v), linear gradient: 0–15% B over 1 min, 15-25% B over 19 min, 25–100% B over 0.1 min, then 100% B for 1.9 min, temperature 55 °C, flow rate 5 mL/min, detection at 260 nm). Ion-pairing reversed-phase separations of plasmids, DNA templates, and mRNAs were carried out on Thermo Fisher DNAPac™ RP, poly(styrene-divinylbenzene), 4 µm, 150 × 10 mm column (mobile phase A: 100 mM triethylammonium acetate, pH 7.0 in water, mobile phase B: 100 mM triethylammonium acetate, pH 7.0 in water/acetonitrile 3:1 (v/v), linear gradient: 46–66% B over 20 min, 66–100% B over 0.1 min, then 100% B for 4.9 min, temperature 65 °C (time-course SPAAC analysis), linear gradient: 0–46% B over 1 min, 46–66% B over 20 min, 66–100% B over 0.1 min, then 100% B for 3.9 min, temperature 65 °C (all preparative mRNA purifications), or linear gradient: 45–75% B over 30 min, 75–90% B over 1 min, then 90% B for 4 min, temperature 50 °C (all preparative plasmid and DNA template purifications), flow rate 2 mL/min, detection at 260 nm).

### NMR

NMR spectra were recorded at 25 °C on a Bruker AVIII HD 500 instrument at 500.30 MHz (^1^H), 125.81 MHz (^13^C) and 202.53 MHz (^31^P). Raw NMR files were processed using MestReNova 14.3 (Mestrelab Research) software. For referencing ^1^H, ^1^H-^1^H COSY, and ^1^H-^13^C HSQC spectra, the residual solvent signal was used (D_2_O, δ 4.7901 ppm). NMR peak assignments were made/ensured based on 2D experiments.

### HRMS

High-resolution mass spectra of small molecules were recorded on a Thermo Scientific Exactive High-Resolution Orbitrap FTMS instrument at negative ion mode (ESI (-)).

### LC-MS

LC-MS analyses of nucleic acids were carried out on a Waters Acquity UHPLC instrument coupled with Waters Xevo G2 QTof mass spectrometer. For instrument control, data acquisition and data processing (including deconvolution of the raw spectra), MassLynx 4.1 (Waters) software was used. Ion-pairing reversed-phase separations of oligonucleotides were carried out on Waters ACQUITY Premier Oligonucleotide C18, 1.7 µm, 130 Å, 2.1 ×50 mm column (routine analyses, i.e., assessment of CleaN3 and oligonucleotides purity) or Waters ACQUITY Premier Oligonucleotide C18, 1.7 µm, 130 Å, 2.1 × 150 mm column (high-resolution analyses, i.e., determination of IVT priming efficiency). Mobile phase A: 200 mM 1,1,1,3,3,3-hexafluoro-2-propanol, 8.15 mM triethylamine, in water/methanol 95:5 (v/v), mobile phase B: mobile phase A/methanol 2:8 (v/v), mobile phase C: 200 mM 1,1,1,3,3,3-hexafluoro-2-propanol, 8.15 mM triethylamine, in water, linear gradient: 0–100% B in C over 8 min, then 0% B for 2 min (assessment of CleaN3 purity), linear gradient: 0–16% B in A over 0.1 min, 16–26% B in A over 7.4 min, then 100% B for 0.5 min, and 100% A for 2 min (assessment of oligonucleotides purity), linear gradient: 0-12% B in A over 2.5 min, 12-20% B in A over 27.5 min, then 100% B for 5 min, and 100% A for 5 min (determination of IVT priming efficiencies), temperature 55 °C, flow rate 0.2 mL/min, PDA detection at 260 nm. The following tuning parameters were used for the MS data acquisition: negative polarity, capillary voltage 1.8 kV, sampling cone voltage 40 V, extraction cone voltage 2.1 V, source temperature 80 °C, cone gas flow 30 L/h, desolvation gas flow 600 L/h. The MS data were acquired in resolution mode and normal dynamic range, from 600 Da to 1800 Da, with 0.5 s scan time. Perfluorotetradecanoic acid was used as a LockSpray reference. The raw mass spectra were deconvolved using the maximum entropy method to produce the true molecular mass spectra.

### Chemical synthesis of CleaN3

The synthesis was carried out manually, using a TWIST synthesis column with syringe fittings (#20-0040-00, Glen Research) and plastic syringes (see Supplementary Fig. 11 for the experimental setup).

First, Primer Support™ 5 G ribo G 300 (#28996442, Cytiva, 303 µmol/g, 248.3 mg, 75.2 µmol, 1.0 equiv.) was loaded into an empty column. Subsequently, a trichloroacetic acid solution (3% v/v in DCM, 10 mL) was passed through the solid support over 1 min, followed by acetonitrile wash (10 mL). The support was then dried using argon. Next, 2′-*O*-Me-A (Pac) CE-phosphoramidite solution (0.2 M in acetonitrile, 0.8 mL, 151 µmol, 2.0 equiv.) was mixed with 5-benzylthio-1*H*-tetrazole solution (0.3 M in acetonitrile, 0.8 mL, 226 µmol, 3.0 equiv.) and the resulting mixture was repetitively passed back and forth through the solid support over 12 min, after which the solid support was washed with acetonitrile (10 mL) and dried with argon. Next, oxidizer solution (0.1 M iodine in THF/water/pyridine 77:2:21 (v/v/v), 3.0 mL, 301 µmol, 4.0 equiv.) was passed through the solid support over 2 min followed by acetonitrile wash (10 mL). The support was then dried using argon. Next, a trichloroacetic acid solution (3% v/v in DCM, 10 mL) was passed through the solid support over 1 min, followed by acetonitrile wash (10 mL). The support was then dried using argon. Next, methyltriphenylphosphonium iodide solution (0.5 M in DMF, 1.5 mL, 752 µmol, 10 equiv.) was repetitively passed back and forth through the solid support over 1 h, after which the solid support was washed with acetonitrile (10 mL) and dried with argon. Subsequently, the column was placed in an oven heated at 55 °C, and the saturated solution of sodium azide in DMF was pushed through the solid support in 1.0 mL portions every 1 h (6.0 mL in total). One hour after adding the last portion of sodium azide solution, the solid support was washed with DMF (10 mL), acetonitrile (10 mL), and dried with argon. The solid support was transferred to a glass vial, and ammonia solution (35% in water, 3 mL) was added. The vial was tightly sealed and incubated at 55 °C overnight. Next, the mixture was filtered through a 0.45 µm syringe filter, evaporated under reduced pressure to dryness, redissolved in water (1 mL) and purified using HPLC (Phenomenex Kinetex® EVO C18, 5 µm, 100 Å, 250 × 10.0 mm column, mobile phase A: 50 mM ammonium acetate, pH 5.9 in water, mobile phase B: acetonitrile, linear gradient: 0–80% B over 12 min, 80–100% B over 1 min, then 100% B for 2 min, temperature 25 °C, flow rate 5 mL/min, detection at 260 nm). Collected fractions containing the desired compound were combined and freeze-dried, giving CleaN3 as a white powder (27.2 mg, 40.7 µmol, 54% isolated yield). **HRMS** ESI (-) calculated m/z for C_21_H_25_N_13_O_10_P^-^ [M-H]^-^ 650.1590, found 650.1589. ^**1**^**H NMR** (500 MHz, D_2_O) δ 8.23 (s, 1H, A8), 8.12 (s, 1H, A2), 7.91 (s, 1H, G8), 5.97 (d, *J* = 4.6 Hz, 1H, A1′), 5.78 (d, *J* = 5.0 Hz, 1H, G1′), 4.76 (dd, *J* = 8.9, 4.6 Hz, 1H, A3′), 4.73 (t, *J* = 5.0 Hz, 1H, G2′), 4.49 (t, *J* = 5.0 Hz, 1H, G3′), 4.43 (t, *J* = 4.6 Hz, 1H, A2′), 4.32 – 4.28 (m, 1H, G4′), 4.27 (q, *J* = 4.6 Hz, 1H, A4′), 4.21 – 4.13 (m, 2H, G5′), 3.62 – 3.51 (m, 2H, A5′), 3.44 (s, 3H, A2′OMe). ^**13**^**C{**^**1**^**H} NMR** (126 MHz, D_2_O) δ 158.16 (G6), 153.47 (G2), 152.91 (A6), 151.13 (G4), 149.44 (A2), 148.10 (A4), 140.58 (A8), 137.29 (G8), 118.46 (A5), 115.91 (G5), 87.63 (G1′), 86.08 (A1′), 83.17 (d, J = 9.5 Hz, G4′), 81.91 (d, J = 4.1 Hz, A4′), 81.28 (d, J = 4.5 Hz, A2′), 73.31 (G2′), 72.43 (d, J = 5.4 Hz, A3′), 69.97 (G3′), 65.19 (d, J = 5.5 Hz, G5′), 57.94 (A2′OMe), 50.97 (A5′). ^**31**^**P{**^**1**^**H} NMR** (203 MHz, D_2_O) δ -0.93.

### Automated on-chip electrophoresis

Automated on-chip electrophoresis of nucleic acids was carried out on the Agilent 2100 Bioanalyzer instrument. For instrument control, data acquisition and data processing, 2100 Expert B.02.11 (Agilent Technologies) software was used. Electrophoretic separations of mRNAs were carried out using Agilent RNA 6000 Nano kit (#5067-1511, Agilent Technologies) using the manufacturer’s protocol.

### Automated solid-phase synthesis of oligonucleotides

Automated solid-phase synthesis of oligonucleotides was performed on an Applied Biosystems 394 automated DNA/RNA synthesiser under the control of OligoNet 1.0.1 (Applied Biosystems) software. Syntheses of oligonucleotides were carried out on a 1 µmol (short and medium length sequences) or 0.2 µmol (long sequences) scale using a standard phosphoramidite cycle of detritylation, coupling, capping, and oxidation. For the synthesis of short sequences (short PCR primers), 500 Å CPG supports: dG (iBu), dA (Bz), dC (Ac), and dT (#LK2262-P008, #LK2263-P008, #LK2357-P008, #LK2261-P008, Link Technologies) were used. For the synthesis of medium-length sequences (medium-length PCR primers, DNAzymes 10–23), 1000 Å CPG supports: dG (iBu), dA (Bz), dC (Ac), and dT (#LK2272-P008, #LK2273-P008, #LK2275-P008, #LK2271-P008, Link Technologies) were used. For the synthesis of long sequences (long PCR primers, inserts for cloning), 3000 Å CPG supports: dG (iBu), dA (Bz), dC (Ac), and dT (#LK2384-P002, #LK2381-P002, #LK2383-P002, #LK2386-P002, Link Technologies) were used. For all DNA syntheses, DMT-dG(ib), DMT-dA(bz), DMT-dC(ac), and DMT-dT (#G111030-12X2G, #A111030-12X2G, #C113030-12X2G, #T111030-12X2G, Sigma-Aldrich) phosphoramidites were used. 5′-phosphate was introduced to oligonucleotides using Chemical Phosphorylating Reagent (CPR) (#LK2101-F100, Link Technologies). 5′-amine for subsequent NHS ester labelling was introduced to oligonucleotides using 5′-MMT-Amino Modifier C6 CE-Phosphoramidite (#LK2123-F100, Link Technologies). All phosphoramidites were dissolved in anhydrous acetonitrile (#L010000-6X100ML, Sigma-Aldrich) to a concentration of 0.1 M immediately prior to use. For detritylation, coupling, capping, oxidation, and washes during the synthesis cycle, the following reagents were used (respectively): TCA Deblock (#L020000-4X4L, Sigma-Aldrich), BTT Activator (#LK3160-D450, LGC Biosearch Technologies), Cap A (#L040030-6X450ML, Sigma-Aldrich)/Cap B (#L050030-6X450ML, Sigma-Aldrich), oxidizer (#L060020-6X450ML, Sigma-Aldrich), and acetonitrile (#L010000-4X4L, Sigma-Aldrich). The coupling time was 45 s for standard DNA monomers and 10 min for modifier amidites. Prior to deprotection of oligonucleotides, the cyanoethyl groups were removed by exposing the solid support to the DEA wash solution (20% diethylamine in acetonitrile, #LK4028-L001, LGC Biosearch Technologies) for 10 min at room temperature. Cleavage from the solid support and deprotection of DNA oligonucleotides were achieved by exposure to AMA (35% aqueous ammonia (#10305220, Fisher Scientific)/40% aqueous methylamine (#426466-4X100ML, Sigma-Aldrich) 1:1 v/v) for 15 min at 65 °C. All obtained oligonucleotides were purified using HPLC (details in the *HPLC* section) and/or desalted (details in the *nucleic acid desalting* section).

### Flow cytometry

Flow cytometry analyses were carried out using an LSRFortessa™ X-20 (BD Biosciences) instrument equipped with BD™ High Throughput Sampler (used in all experiments) and 5 spatially separated lasers: UV – 355 nm, violet – 405 nm, blue – 488 nm (used for EGFP), yellow-green – 561 nm (used for mCherry and LEGENDplex™ assay reporter), and red – 640 nm (used for AF647 and LEGENDplex™ assay beads classification). The following bandpass filters were used: 530/30 (used for EGFP), 610/20 (used for mCherry), 586/15 (used for LEGENDplex™ assay reporter), and 670/30 (used for AF647 and LEGENDplex™ assay beads classification). For quantitation of mRNA uptake and protein expression, fluorescence pulse areas were recorded. The PMT gains were adjusted to achieve a clear separation of populations transfected with mRNA, expressing fluorescent proteins, from control, mock-transfected populations and to ensure the signals were within the linear response range of the cytometer. For instrument control and data acquisition, FACSDiva 9.2 (BD) software was used.

### Microscopy

Spinning disk confocal microscopy was carried out on an Olympus IXplore SpinSR Super Resolution Microscope System consisting of the IX-83 inverted microscope frame equipped with a Yokogawa CSU-W1 SoRa confocal scanner unit, Teledyne Photometrics Prime BSI (used in all experiments) and Prime 95B sCMOS cameras, SD-MGCA motorised magnification changer with 1x magnification (used for all time-lapse experiments) and 3.2x magnification (used for FISH, cooccurrence, transfected cell video, and live cell 3D imaging), SoRa 50 μm (used in all experiments) and 50 μm pinhole disks, IX3 DIC optics, IX3-ZDC2 TruFocus Z-Drift compensation module (used for all time-lapse experiments), Coherent OBIS LX 405 nm (used for Hoescht 33342 and DAPI), 488 nm (used for EGFP), 561 nm (used for ATTO 565, LysoTracker™ Red, ER-Tracker™ Red, and CellLight™ Early Endosomes-RFP, BacMam 2.0), 640 nm (used for AF647) lasers, B447/60 (used for Hoescht 33342 and DAPI), B525/50 (used for EGFP), B617/73 (used for ATTO 565, LysoTracker™ Red, ER-Tracker™ Red, and CellLight™ Early Endosomes-RFP, BacMam 2.0) and B685/40 (used for AF647) bandpass filters, and Olympus UPLAPO60XOHR 60x/1.50 (used for all experiments) oil immersion objective. In all experiments, the disk speed was set to 4000 rpm. For live cell imaging, slides were maintained at 37 °C, 5% CO_2_. Fixed cell imaging was carried out at room temperature and atmospheric CO_2_ level. For instrument control and data acquisition, cellSens Dimension 3.2 (Olympus) software was used.

Raw spinning disk confocal 3D images were deconvolved and corrected for chromatic aberration using Huygens 24.04 (Scientific Volume Imaging). For deconvolution, Classic MLE algorithm was used with 30 iterations, slice by slice brick mode, optimized iteration mode, and acuity set to 0. For correcting chromatic aberration, XYZ shift method was used.

Microscopy images were processed (cropping, adjusting levels, applying LUTs, adding scale bars, exporting) and analysed (colocalization/cooccurrence) using Fiji 2.14.0 (National Institutes of Health, USA). For transfected cell video, bleach correction was applied using Bleach Correction 2.1.0 (Fiji plugin) with histogram matching mode. For colocalization/cooccurrence analysis, JACoP 1.1.10 (Fiji plugin) was used with no thresholds set for Pearson’s coefficients calculation and manual thresholds set based on the background levels from control images for Mander’s coefficients calculation. Cells were segmented based on intensity in the EGFP channel or cell contours in the DIC channel. 3D images were rendered using Imaris 10.2 (Bitplane).

Videos were edited and rendered using DaVinci Resolve Studio 18.5 (Blackmagic Design) based on level-adjusted 8-bit RGB tiff frames exported from Fiji or videos exported from Imaris. For colour management, sRGB colour space and gamma were used.

### Determination of concentrations of nucleic acids

Nucleic acid concentrations were calculated using NanoDrop 2000/2000c 1.6 (Thermo Scientific) software (IVT yield determination) or Oligonucleotide Properties Calculator^63^ (all other experiments) based on absorbance measured using NanoDrop 2000 spectrophotometer (Thermo Scientific).

### Nucleic acid desalting

Collected fractions of nucleic acids after HPLC purification were desalted on Amicon^®^ Ultra-4 30 kDa MWCO (#UFC803096, Merck) centrifugal filters (DNA templates, mRNAs) or Amicon^®^ Ultra-4 3 kDa MWCO (#UFC800396, Merck) centrifugal filters (oligonucleotides) following the manufacturer’s recommendations. After the first round of centrifugation (concentration of samples), samples were repeatedly diluted with 1 mM sodium citrate buffer, pH 6.4 (RNA) or 10 mM Tris buffer, pH 8.0 (DNA) and concentrated (three times in total).

### Mammalian cell culture

HEK293, HeLa, HCT-116, and HepG2 cells were cultured in DMEM, high glucose, GlutaMAX™ Supplement, pyruvate (#31966021, Thermo Fisher Scientific) medium containing glucose (4.5 g/L), GlutaMAX™ Supplement (862 mg/L), sodium pyruvate (110 mg/L), sodium bicarbonate (3.7 g/L), and phenol red (15 mg/L), supplemented with 10% (v/v) fetal bovine serum (#A5209402, Thermo Fisher Scientific) at 37 °C and 5% CO_2_. For light microscopy-based experiments with HeLa cells, the medium was exchanged with FluoroBrite™ DMEM (#A1896701, Thermo Fisher Scientific) containing glucose (4.5 g/L) and sodium bicarbonate (3.7 g/L), supplemented with 10% (v/v) fetal bovine serum (#A5209402, Thermo Fisher Scientific), sodium pyruvate (#11360070, Thermo Fisher Scientific, 1X, 110 mg/L), and GlutaMAX™ Supplement (#35050061, Thermo Fisher Scientific, 1X, 434 mg/L). MUTZ-3 cells were cultured in MEM α, nucleosides (#12571063, Thermo Fisher Scientific) medium containing L-glutamine (292 mg/L), ribonucleosides (10 mg/L each), deoxyribonucleosides (10 mg/L each), sodium bicarbonate (2.2 g/L), and phenol red (10 mg/L), supplemented with 20% (v/v) heat-inactivated fetal bovine serum (#A5209402, Thermo Fisher Scientific), and GM-CSF (#G5035-5UG, Sigma-Aldrich, 40 ng/mL) at 37 °C and 5% CO_2_. In regular subcultures of the adherent cells, TrypLE™ Express Enzyme (1X), no phenol red (#12604013, Thermo Fisher Scientific) was used for cell dissociation. Regular mycoplasma testing was performed using a MycoAlert® PLUS Mycoplasma Detection Kit (#LT07-701, Lonza).

### Preparation of plasmids

*pRSET-IRES-EGFP* plasmid was prepared using Gibson assembly. The vector was obtained by digesting *pRSET-B mCherry* plasmid with EcoRI (#R0101S, New England Biolabs) and BamHI (#R0136S, New England Biolabs) restriction enzymes following the manufacturer’s protocol. Gibson-assembly-compatible inserts were prepared by PCR amplifying *pCAG-EOMES-EGFP-IRES-Puro* with IRES- *(IRES_GA_F, IRES_GA_R)* and EGFP- *(EGFP_GA_F, EGFP_GA_R)* specific primers. Vector and inserts were separated on a 0.8% agarose (1x TBE) gel, excised, and extracted using a Monarch® DNA Gel Extraction Kit (#T1020L, New England Biolabs) following the manufacturer’s recommendations. Gibson assembly reactions were carried out using Gibson Assembly® Master Mix (#E2611S, New England Biolabs). For a 10 μL reaction, *pRSET* vector (25 ng) was mixed with 3 equiv. of both inserts and water (5.0 μL final volume), to which Gibson Assembly® Master Mix (5.0 μL) was added and mixed by vortexing. Gibson assembly reaction was incubated at 50 °C for 15 min and was then transferred to ice. Next, NEB® 5-alpha Competent *E. coli* (#C2987H, New England Biolabs, 50 μL) were transformed with an aliquot of the reaction mixture (5.0 μL) according to the manufacturer’s high efficiency transformation protocol (C2987H/C2987I). Transformed bacteria were plated onto ampicillin (#A9518-5G, Sigma-Aldrich, 100 μg/mL) selection plates and incubated at 37 °C for 18 h. Colonies were picked and grown in LB broth (Miller) (#L3522-1KG, Sigma-Aldrich, 25 g/L, 5 mL) supplemented with ampicillin (#A9518-5G, Sigma-Aldrich, 100 μg/mL) at 37 °C, 1000 rpm, for 18 h. Plasmid was isolated from liquid culture using a QIAprep® Spin Miniprep Kit (#27104, Qiagen) according to the manufacturer’s protocol. The size and purity of the obtained plasmid were verified using electrophoresis on a 0.8% agarose (1x TBE) gel. For the size assessment, the plasmid was linearised by NheI-HF (#R3131S, New England Biolabs) digest. The sequence of isolated plasmid was verified using Oxford Nanopore sequencing.

*pmRNA-EGFP-A120* plasmid was prepared through traditional cloning in two steps. In the first step, the T7 class III φ6.5 promoter was substituted with the A-inserted T7 class III φ6.5 promoter, and an additional AarI restriction site was added upstream of the promoter. The vector was obtained by digesting *pRNA2-(A)128* plasmid with ScaI-HF (#R3122S, New England Biolabs) and MluI-HF (#R3198S, New England Biolabs) restriction enzymes following the manufacturer’s protocol and subsequently purified using HPLC (details in the *HPLC* section). Then, the vector was ligated using T4 DNA ligase (#M0202M, New England Biolabs) with an insert prepared by annealing *A_ins_*φ*6.5-1* and *A_ins_*φ*6.5-2* DNA oligonucleotides, following the manufacturer’s protocol. For a 20 μL reaction, solutions containing the vector (50 ng) and insert (3 equiv.) were mixed with T4 DNA ligase buffer (2 μL), T4 DNA ligase (1 μL), and water (to a final volume of 20 μL), and incubated at room temperature for 2 h. Subsequently, the reaction mixture was heated to 65 °C for 10 min and chilled on ice. Next, NEB® 5-alpha Competent *E. coli* (#C2987H, New England Biolabs, 50 μL) were transformed with an aliquot of the reaction mixture (5.0 μL) according to the manufacturer’s high efficiency transformation protocol (C2987H/C2987I). Transformed bacteria were plated onto kanamycin (#26897.02, Serva, 50 μg/mL) selection plates and incubated at 37 °C for 14 h. Colonies were picked and grown in LB broth (Miller) (#L3522-1KG, Sigma-Aldrich, 25 g/L, 5 mL) supplemented with kanamycin (#26897.02, Serva, 50 μg/mL) at 37 °C, 300 rpm, for 16 h. Plasmid was isolated from liquid culture using a QIAprep® Spin Miniprep Kit (#27104, Qiagen) according to the manufacturer’s protocol. The size and purity of the obtained plasmid were verified using electrophoresis on a 1.2% agarose (1x TBE) gel. For the size assessment, the plasmid was cleaved in two sites using AarI (#ER1582, Thermo Scientific). The sequence of isolated plasmid was verified using Oxford Nanopore sequencing. In the second step, due to the instability of the original poly(A) sequence in plasmid maintained in NEB® 5-alpha Competent *E. coli*, it was substituted with a new fragment encoding A_120_ tail upstream of the AarI restriction site, and the plasmid was cloned in NEB® Stable Competent *E. coli*. The vector was obtained by digesting the plasmid from the first step with PmeI (#R0560S, New England Biolabs) and BaeI (#R0613S, New England Biolabs) restriction enzymes following the manufacturer’s protocol and subsequently purified on a 1.0% agarose (1x TBE) gel, excised, and extracted using a Monarch® DNA Gel Extraction Kit (#T1020L, New England Biolabs) following the manufacturer’s recommendations. Then, the vector was ligated using T4 DNA ligase (#M0202M, New England Biolabs) with an insert prepared by annealing *A120-AarI-1* and *A120-AarI-2* DNA oligonucleotides, following the manufacturer’s protocol. For a 20 μL reaction, solutions containing the vector (50 ng) and insert (3 equiv.) were mixed with T4 DNA ligase buffer (2 μL), T4 DNA ligase (1 μL), and water (to a final volume of 20 μL), and incubated at room temperature for 2 h. Subsequently, the reaction mixture was heated to 65 °C for 10 min and chilled on ice. Next, NEB® Stable Competent *E. coli* (High Efficiency) (#C3040H, New England Biolabs, 50 μL) were transformed with an aliquot of the reaction mixture (2.0 μL) according to the manufacturer’s protocol for cloning DNA containing repeat elements (C3040). Transformed bacteria were plated onto kanamycin (#26897.02, Serva, 30 μg/mL) selection plates and incubated at 30 °C for 24 h. Colonies were picked and grown in LB broth (Miller) (#L3522-1KG, Sigma-Aldrich, 25 g/L, 15 mL) supplemented with kanamycin (#26897.02, Serva, 30 μg/mL) at 30 °C, 300 rpm, for 16 h. For sequence verification using Oxford Nanopore sequencing and poly(A) tail length assessment through AarI (#ER1582, Thermo Scientific) and PmeI (#R0560S, New England Biolabs) restriction digest in rCutSmart™ Buffer (#B6004S, New England Biolabs) followed by a 3.0% agarose (1x TBE) gel analysis, the plasmid was isolated from liquid culture using an E.Z.N.A.® Plasmid DNA Mini Kit II (#D6945-01, Omega Bio-tek) according to the manufacturer’s protocol. For the large-scale plasmid preparation for downstream DNA template generation, the starter liquid culture was diluted (1:500) in LB broth (Miller) (#L3522-1KG, Sigma-Aldrich, 25 g/L, 2.5 L) supplemented with kanamycin (#26897.02, Serva, 30 μg/mL) and incubated at 30 °C, 160 rpm, for 16 h. Plasmid was isolated from liquid culture using a Plasmid Giga Kit (5) (#12191, Qiagen) according to the manufacturer’s protocol. The size and purity of the obtained plasmid were verified using electrophoresis on a 1.0% agarose (1x TBE) gel. For the size assessment, the plasmid was cleaved in two sites using AarI (#ER1582, Thermo Scientific). The sequence of isolated plasmid was verified using Oxford Nanopore sequencing.

*pmRNA-mCherry-A120* plasmid was prepared using NEBuilder® HiFi DNA assembly. The vector was obtained by digesting *pmRNA-EGFP-A120* plasmid with AgeI-HF (#R3552S, New England Biolabs) and NotI-HF (#R3189S, New England Biolabs) restriction enzymes following the manufacturer’s protocol and subsequently separated on a 1.0% agarose (1x TBE) gel, excised, and extracted using a Monarch® DNA Gel Extraction Kit (#T1020L, New England Biolabs) following the manufacturer’s recommendations. Insert was prepared by PCR amplifying *pmCherry-N1 GBP (GBP-mCherry)* with mCherry-specific primers *(mCherry_F, mCherry_R)*. NEBuilder® HiFi DNA assembly reactions were carried out using NEBuilder® HiFi DNA Assembly Master Mix (#E2621S, New England Biolabs). For a 20 μL reaction, *pmRNA-A120* vector (100 ng) was mixed with 2 equiv. of insert, and water (10.0 μL final volume), to which NEBuilder® HiFi DNA Assembly Master Mix (10.0 μL) was added. The reaction mixture was incubated at 50 °C for 15 min and was then transferred to ice. Next, NEB® Stable Competent *E. coli* (High Efficiency) (#C3040H, New England Biolabs, 50 μL) were transformed with an aliquot of the reaction mixture (2.0 μL) according to the manufacturer’s protocol for cloning DNA containing repeat elements (C3040). Transformed bacteria were plated onto kanamycin (#26897.02, Serva, 30 μg/mL) selection plates and incubated at 30 °C for 24 h. Colonies were picked and grown in LB broth (Miller) (#L3522-1KG, Sigma-Aldrich, 25 g/L, 15 mL) supplemented with kanamycin (#26897.02, Serva, 30 μg/mL) at 30 °C, 300 rpm, for 16 h. For sequence verification using Oxford Nanopore sequencing and poly(A) tail length assessment through AarI (#ER1582, Thermo Scientific) and PmeI (#R0560S, New England Biolabs) restriction digest in rCutSmart™ Buffer (#B6004S, New England Biolabs) followed by a 3.0% agarose (1x TBE) gel analysis, the plasmid was isolated from liquid culture using an E.Z.N.A.® Plasmid DNA Mini Kit II (#D6945-01, Omega Bio-tek) according to the manufacturer’s protocol. For the large-scale plasmid preparation for downstream DNA template generation, the starter liquid culture was diluted (1:500) in LB broth (Miller) (#L3522-1KG, Sigma-Aldrich, 25 g/L, 2.5 L) supplemented with kanamycin (#26897.02, Serva, 30 μg/mL) and incubated at 30 °C, 160 rpm, for 16 h. Plasmid was isolated from liquid culture using a Plasmid Giga Kit (5) (#12191, Qiagen) according to the manufacturer’s protocol. The size and purity of the obtained plasmid were verified using electrophoresis on a 1.0% agarose (1x TBE) gel. For the size assessment, the plasmid was cleaved in two sites using AarI (#ER1582, Thermo Scientific). The sequence of isolated plasmid was verified using Oxford Nanopore sequencing.

*pmRNA-iEMCV-EGFP-A120* plasmid was prepared through NEBuilder® HiFi DNA assembly and traditional cloning in two steps. In the first step, the iEMCV fragment was inserted upstream of the EGFP in the *pmRNA-EGFP-A120* plasmid. The vector was prepared by PCR amplifying *pmRNA-EGFP-A120* with *pmRNA-EGFP-A120-v1_F* and *pmRNA-EGFP-A120-v1_R* primers. The insert was prepared by PCR amplifying *pRSET-IRES-EGFP* with iEMCV-specific primers *(iEMCV_F, iEMCV_R)*. NEBuilder® HiFi DNA assembly reactions were carried out using NEBuilder® HiFi DNA Assembly Master Mix (#E2621S, New England Biolabs). For a 20 μL reaction, *pmRNA-A120* vector (100 ng) was mixed with 2 equiv. of insert, and water (10.0 μL final volume), to which NEBuilder® HiFi DNA Assembly Master Mix (10.0 μL) was added. The reaction mixture was incubated at 50 °C for 15 min and was then transferred to ice. Next, NEB® Stable Competent *E. coli* (High Efficiency) (#C3040H, New England Biolabs, 50 μL) were transformed with an aliquot of the reaction mixture (2.0 μL) according to the manufacturer’s protocol for cloning DNA containing repeat elements (C3040). Transformed bacteria were plated onto kanamycin (#26897.02, Serva, 30 μg/mL) selection plates and incubated at 30 °C for 24 h. Colonies were picked and grown in LB broth (Miller) (#L3522-1KG, Sigma-Aldrich, 25 g/L, 15 mL) supplemented with kanamycin (#26897.02, Serva, 30 μg/mL) at 30 °C, 300 rpm, for 16 h. Plasmid was isolated from liquid culture using an E.Z.N.A.® Plasmid DNA Mini Kit II (#D6945-01, Omega Bio-tek) according to the manufacturer’s protocol. The sequence of isolated plasmid was verified using Oxford Nanopore sequencing. In the second step, the AarI restriction site downstream of the poly(A) stretch was replaced with the BsaI restriction site. The vector was obtained by digesting the plasmid from the first step with PmeI (#R0560S, New England Biolabs) and BaeI (#R0613S, New England Biolabs) restriction enzymes following the manufacturer’s protocol and subsequently purified on a 1.0% agarose (1x TBE) gel, excised, and extracted using a Monarch® DNA Gel Extraction Kit (#T1020L, New England Biolabs) following the manufacturer’s recommendations. Then, the vector was ligated using T4 DNA ligase (#M0202M, New England Biolabs) with an insert prepared by annealing *A120-BsaI-1* and *A120-BsaI-2* DNA oligonucleotides, following the manufacturer’s protocol. For a 20 μL reaction, solutions containing the vector (50 ng) and insert (3 equiv.) were mixed with T4 DNA ligase buffer (2 μL), T4 DNA ligase (1 μL), and water (to a final volume of 20 μL), and incubated at room temperature for 2 h. Subsequently, the reaction mixture was heated to 65 °C for 10 min and chilled on ice. Next, NEB® Stable Competent *E. coli* (High Efficiency) (#C3040H, New England Biolabs, 50 μL) were transformed with an aliquot of the reaction mixture (2.0 μL) according to the manufacturer’s protocol for cloning DNA containing repeat elements (C3040). Transformed bacteria were plated onto kanamycin (#26897.02, Serva, 30 μg/mL) selection plates and incubated at 30 °C for 24 h. Colonies were picked and grown in LB broth (Miller) (#L3522-1KG, Sigma-Aldrich, 25 g/L, 15 mL) supplemented with kanamycin (#26897.02, Serva, 30 μg/mL) at 30 °C, 300 rpm, for 16 h. For sequence verification using Oxford Nanopore sequencing and poly(A) tail length assessment through BsaI-HF®v2 (#R3733S, New England Biolabs) and PmeI (#R0560S, New England Biolabs) restriction digest in rCutSmart™ Buffer (#B6004S, New England Biolabs) followed by a 3.0% agarose (1x TBE) gel analysis, plasmid was isolated from liquid culture using an E.Z.N.A.® Plasmid DNA Mini Kit II (#D6945-01, Omega Bio-tek) according to the manufacturer’s protocol. For the large-scale plasmid preparation for downstream DNA template generation, the starter liquid culture was diluted (1:500) in LB broth (Miller) (#L3522-1KG, Sigma-Aldrich, 25 g/L, 2.5 L) supplemented with kanamycin (#26897.02, Serva, 30 μg/mL) and incubated at 30 °C, 160 rpm, for 16 h. Plasmid was isolated from liquid culture using a Plasmid Giga Kit (5) (#12191, Qiagen) according to the manufacturer’s protocol. The size and purity of the obtained plasmid were verified using electrophoresis on a 1.0% agarose (1x TBE) gel. For the size assessment, the plasmid was cleaved in two sites using BsaI-HF®v2 (#R3733S, New England Biolabs). The sequence of isolated plasmid was verified using Oxford Nanopore sequencing.

*pmRNA-iHCV-EGFP-A120* plasmid was prepared through NEBuilder® HiFi DNA assembly. The vector was prepared by PCR amplifying *pmRNA-EGFP-A120* with *pmRNA-EGFP-A120-v2_F* and *pmRNA-EGFP-A120-v2_R* primers. The insert was prepared by PCR amplifying *pFR_HCV_xb* with iHCV-specific primers *(iHCV_F, iHCV_R)*. NEBuilder® HiFi DNA assembly reactions were carried out using NEBuilder® HiFi DNA Assembly Master Mix (#E2621S, New England Biolabs). For a 20 μL reaction, *pmRNA-A120* vector (100 ng) was mixed with 2 equiv. of insert, and water (10.0 μL final volume), to which NEBuilder® HiFi DNA Assembly Master Mix (10.0 μL) was added. The reaction mixture was incubated at 50 °C for 15 min and was then transferred to ice. Next, NEB® Stable Competent *E. coli* (High Efficiency) (#C3040H, New England Biolabs, 50 μL) were transformed with an aliquot of the reaction mixture (2.0 μL) according to the manufacturer’s protocol for cloning DNA containing repeat elements (C3040). Transformed bacteria were plated onto kanamycin (#26897.02, Serva, 30 μg/mL) selection plates and incubated at 30 °C for 24 h. Colonies were picked and grown in LB broth (Miller) (#L3522-1KG, Sigma-Aldrich, 25 g/L, 15 mL) supplemented with kanamycin (#26897.02, Serva, 30 μg/mL) at 30 °C, 300 rpm, for 16 h. For sequence verification using Oxford Nanopore sequencing and poly(A) tail length assessment through AarI (#ER1582, Thermo Scientific) and PmeI (#R0560S, New England Biolabs) restriction digest in rCutSmart™ Buffer (#B6004S, New England Biolabs) followed by a 3.0% agarose (1x TBE) gel analysis, the plasmid was isolated from liquid culture using an E.Z.N.A.® Plasmid DNA Mini Kit II (#D6945-01, Omega Bio-tek) according to the manufacturer’s protocol. For the large-scale plasmid preparation for downstream DNA template generation, the starter liquid culture was diluted (1:500) in LB broth (Miller) (#L3522-1KG, Sigma-Aldrich, 25 g/L, 2.5 L) supplemented with kanamycin (#26897.02, Serva, 30 μg/mL) and incubated at 30 °C, 160 rpm, for 16 h. Plasmid was isolated from liquid culture using a Plasmid Giga Kit (5) (#12191, Qiagen) according to the manufacturer’s protocol. The size and purity of the obtained plasmid were verified using electrophoresis on a 1.0% agarose (1x TBE) gel. For the size assessment, the plasmid was cleaved in two sites using AarI (#ER1582, Thermo Scientific). The sequence of isolated plasmid was verified using Oxford Nanopore sequencing.

*pmRNA-iSyn-EGFP* plasmid was prepared through NEBuilder® HiFi DNA assembly. The vector was obtained by digesting *circRNA-synIRES-R25-mNeonGreen* plasmid with BamHI-HF (#R3136S, New England Biolabs) and MfeI-HF (#R3589S, New England Biolabs) restriction enzymes following the manufacturer’s protocol and subsequently separated on a 1.0% agarose (1x TBE) gel, excised, and extracted using a Monarch® DNA Gel Extraction Kit (#T1020L, New England Biolabs) following the manufacturer’s recommendations. The insert was prepared by PCR amplifying *pmRNA-EGFP-A120* with EGFP-specific primers *(oEGFP_F, oEGFP_R)*. NEBuilder® HiFi DNA assembly reactions were carried out using NEBuilder® HiFi DNA Assembly Master Mix (#E2621S, New England Biolabs). For a 20 μL reaction, the *synIRES* vector (100 ng) was mixed with 2 equiv. of the insert and water (10.0 μL final volume), to which NEBuilder® HiFi DNA Assembly Master Mix (10.0 μL) was added. The reaction mixture was incubated at 50 °C for 15 min and was then transferred to ice. Next, NEB® Stable Competent *E. coli* (High Efficiency) (#C3040H, New England Biolabs, 50 μL) were transformed with an aliquot of the reaction mixture (2.0 μL) according to the manufacturer’s protocol for cloning DNA containing repeat elements (C3040). Transformed bacteria were plated onto kanamycin (#26897.02, Serva, 30 μg/mL) selection plates and incubated at 30 °C for 24 h. Colonies were picked and grown in LB broth (Miller) (#L3522-1KG, Sigma-Aldrich, 25 g/L, 15 mL) supplemented with kanamycin (#26897.02, Serva, 30 μg/mL) at 30 °C, 300 rpm, for 16 h. Plasmid was isolated from liquid culture using an E.Z.N.A.® Plasmid DNA Mini Kit II (#D6945-01, Omega Bio-tek) according to the manufacturer’s protocol. The size and purity of the obtained plasmid were verified using electrophoresis on a 1.0% agarose (1x TBE) gel. For the size assessment, the plasmid was cleaved using ScaI-HF (#R3122S, New England Biolabs). The sequence of isolated plasmid was verified using Oxford Nanopore sequencing.

### Preparation of DNA templates for IVT

#### Restriction digest

*tiEMCV-EGFP*, *tiHCV-EGFP*, *tmCherry*, and *tEGFP* DNA templates for IVT were prepared using restriction digest of *pmRNA-iEMCV-EGFP-A120*, *pmRNA-iHCV-EGFP-A120*, *pmRNA-mCherry-A120*, and *pmRNA-EGFP-A120*, respectively. For *tiHCV-EGFP*, *tmCherry*, and *tEGFP*, plasmid (1.3 μg/μL, 615 μL), AarI (#ER1582, Thermo Scientific, 25 μL), 10X AarI buffer (75 μL), and water (35 μL) were mixed and the reaction mix was incubated at 37 °C for 14 h followed by 65 °C for 20 min. For *tiEMCV-EGFP*, plasmid (1.3 μg/μL, 615 μL), BsaI-HF®v2 (#R3733S, New England Biolabs, 20 μL), rCutSmart™ Buffer (#B6004S, New England Biolabs, 75 μL), and water (40 μL) were mixed and the reaction mix was incubated at 37 °C for 14 h followed by 65 °C for 20 min. The digest products were purified using a Monarch® RNA Cleanup Kit (500 μg) (#T2050L, New England Biolabs), following the manufacturer’s recommendations and ion-pairing reversed-phase HPLC (details in the *HPLC* section). The quality of the DNA templates was assessed through electrophoresis on a 1.0% agarose (1x TBE) gel. Poly(A) tail lengths were assessed by PmeI (#R0560S, New England Biolabs) restriction digest in rCutSmart™ Buffer (#B6004S, New England Biolabs) followed by a 3.0% agarose (1x TBE) gel analysis.

#### PCR and restriction digest

*tiEMCV-EGFP* (φ6.5 and φ2.5) DNA templates for IVT were prepared using PCR on *pmRNA-iEMCV-EGFP-A120* with Q5® High-Fidelity 2X Master Mix (#M0492S, New England Biolabs) and subsequent restriction digest with BsaI-HF®v2 (#R3733S, New England Biolabs). For 2 mL reactions, *IVT_*φ*6.5_iEMCV-EGFP-A120_F*, or *IVT_*φ*2.5_iEMCV-EGFP-A120_F* primer (10 μM, 100 μL), *IVT_iEMCV-EGFP-A120_R* primer (10 μM, 100 μL), Q5® High-Fidelity 2X Master Mix (1 mL), *pmRNA-iEMCV-EGFP-A120* template (100 ng/μL, 4 μL), and water (796 μL) were mixed. The reaction mixtures were initially denatured at 98 °C for 30 s, followed by 30 cycles of denaturation (98 °C, 10 s), annealing (71 °C, 30 s), and extension (72 °C, 70 s). After cycling, a final extension step was performed at 72 °C for 2 min. The resulting PCR products were purified using a Monarch® RNA Cleanup Kit (500 μg) (#T2050L, New England Biolabs), following the manufacturer’s recommendations. Subsequently, the PCR products were digested with BsaI-HF®v2. For 200 μL reactions, PCR products (0.7 μg/μL, 140 μL), BsaI-HF®v2 (6.0 μL), rCutSmart™ Buffer (20 μL), and water (34 μL) were mixed and the reaction mixtures were incubated at 37 °C for 14 h followed by 65 °C for 20 min. The digest products were purified using a Monarch® RNA Cleanup Kit (500 μg) (#T2050L, New England Biolabs), following the manufacturer’s recommendations and ion-pairing reversed-phase HPLC (details in the *HPLC* section). The quality of the DNA templates was assessed through electrophoresis on a 1.0% agarose (1x TBE) gel.

#### Tail overhang PCR

*tiEMCV-Cas9* and *tiSyn-EGFP* DNA templates for IVT were prepared using PCR on *pMCS-rybozyme-IRES-CAS9* and *pmRNA-iSyn-EGFP-A120*, respectively, with Q5® High-Fidelity 2X Master Mix (#M0492S, New England Biolabs). For *tiEMCV-Cas9*, *IVT_iEMCV-Cas9-A120_F* primer (10 μM, 100 μL), *IVT_iEMCV-Cas9-A120_R* primer (10 μM, 100 μL), Q5® High-Fidelity 2X Master Mix (1 mL), *pMCS-rybozyme-IRES-CAS9* template (100 ng/μL, 4 μL), and water (796 μL) were mixed (total volume 2 mL). The reaction mixtures were initially denatured at 98 °C for 30 s, followed by 30 cycles of denaturation (98 °C, 10 s), annealing (65 °C, 30 s), and extension (72 °C, 140 s). After cycling, a final extension step was performed at 72 °C for 2 min. For *tiSyn-EGFP*, *IVT_iSyn-EGFP-A120_F* primer (10 μM, 100 μL), *IVT_iSyn-EGFP-A120_R* primer (10 μM, 100 μL), Q5® High-Fidelity 2X Master Mix (1 mL), *pmRNA-iSyn-EGFP-A120* template (100 ng/μL, 4 μL), and water (796 μL) were mixed (total volume 2 mL). The reaction mixtures were initially denatured at 98 °C for 30 s, followed by 30 cycles of denaturation (98 °C, 10 s), annealing (59 °C, 30 s), and extension (72 °C, 45 s). After cycling, a final extension step was performed at 72 °C for 2 min. The resulting PCR products were purified using a Monarch® RNA Cleanup Kit (500 μg) (#T2050L, New England Biolabs), following the manufacturer’s recommendations and ion-pairing reversed-phase HPLC (details in the *HPLC* section). The quality of the DNA templates was assessed through electrophoresis on a 1.0% agarose (1x TBE) gel.

#### PCR

*tcirc-iSyn-EGFP* DNA template for IVT was prepared similarly as described before (ref. ^57^), using PCR on *pmRNA-iSyn-EGFP-A120* with Q5® High-Fidelity 2X Master Mix (#M0492S, New England Biolabs). For 2 mL reaction, *IVT_circ-iSyn-EGFP_F* primer (10 μM, 100 μL), *IVT_circ-iSyn-EGFP_R* primer (10 μM, 100 μL), Q5® High-Fidelity 2X Master Mix (1 mL), *pmRNA-iSyn-EGFP-A120* template (100 ng/μL, 4 μL), and water (796 μL) were mixed. The reaction mixture was initially denatured at 98 °C for 30 s, followed by 30 cycles of denaturation (98 °C, 10 s), annealing (72 °C, 30 s), and extension (72 °C, 45 s). After cycling, a final extension step was performed at 72 °C for 2 min. The resulting PCR product was purified using a Monarch® RNA Cleanup Kit (500 μg) (#T2050L, New England Biolabs), following the manufacturer’s recommendations and ion-pairing reversed-phase HPLC (details in the *HPLC* section). The quality of the DNA template was assessed through electrophoresis on a 1.0% agarose (1x TBE) gel.

### In vitro transcription (non-primed)

Standard in vitro transcription reactions yielding 5′-triphosphate transcripts were carried out using HiScribe® T7 High Yield RNA Synthesis Kit (#E2040S, New England Biolabs). For 20 μL reaction, water (6.0 μL), 10X reaction buffer (2.0 μL), ATP (100 mM, 2.0 μL), GTP (100 mM, 2.0 μL), UTP (100 mM, 2.0 μL), CTP (100 mM, 2.0 μL), DNA template (500 ng/μL, 2.0 μL), and T7 RNA polymerase mix (2.0 μL) were mixed. The reaction mix was incubated in a thermocycler at 37 °C for 2 h. Afterwards, water (30 μL) and DNase I (2.0 μL) were added, and the reaction mix was incubated at 37 °C for 15 min. Transcripts were purified using a Monarch® RNA Cleanup Kit (500 μg) (#T2050L, New England Biolabs), following the manufacturer’s recommendations, and ion-pairing reversed-phase HPLC (details in the *HPLC* section).

### In vitro transcription priming

Primed in vitro transcription reactions yielding 5′-CleanCap or 5′-CleaN3 transcripts were carried out using HiScribe® T7 High Yield RNA Synthesis Kit (#E2040S, New England Biolabs). For 20 μL reaction, water (5.8 μL for CleaN3 or 7.8 μL for CleanCap), 10X reaction buffer (2.0 μL), ATP or 95% ATP:5% m^6^ATP (#NU-1101S, Jena Bioscience) (100 mM, 1.2 μL), GTP (100 mM, 1.0 μL), UTP or m^1^ΨTP (#NU-890L, Jena Bioscience) (100 mM, 1.0 μL), CTP (100 mM, 1.0 μL), IVT primer (CleanCap® Reagent AG, #N-7113-1, TriLink Biotechnologies, 40 mM, 2.0 μL, or CleaN3, 40 mM, 4.0 μL), template DNA (500 ng/μL, 2.0 μL), and T7 RNA polymerase mix (2.0 μL) were mixed. The reaction mix was incubated in a thermocycler at 37 °C for 2 h. Afterwards, water (30 μL) and DNase I (2 μL) were added, and the reaction mix was incubated at 37 °C for 15 min. Primed transcripts were purified using a Monarch® RNA Cleanup Kit (500 μg) (#T2050L, New England Biolabs), following the manufacturer’s recommendations, and ion-pairing reversed-phase HPLC (details in the *HPLC* section). Labelling of 5′-CleaN3 transcripts through SPAAC was performed without HPLC purification prior to setting up the reaction.

### Preparation of circRNA

One-pot in vitro transcription and splicing reactions yielding circular transcripts were carried out similarly as described before^57^, using HiScribe® T7 High Yield RNA Synthesis Kit (#E2040S, New England Biolabs). For 20 μL reaction, water (6.0 μL), 10X reaction buffer (2.0 μL), ATP (100 mM, 2.0 μL), GTP (100 mM, 2.0 μL), UTP (100 mM, 2.0 μL), CTP (100 mM, 2.0 μL), DNA template (500 ng/μL, 2.0 μL), and T7 RNA polymerase mix (2.0 μL) were mixed. The reaction mix was incubated in a thermocycler at 37 °C for 16 h. Afterwards, water (30 μL) and DNase I (2.0 μL) were added, and the reaction mix was incubated at 37 °C for 15 min. Subsequently, the reaction mixtures were cleaned up using a Monarch® RNA Cleanup Kit (500 μg) (#T2050L, New England Biolabs), following the manufacturer’s recommendations. Next, to the eluate (50 μL), water (2.0 μL), 10X RNase R Reaction Buffer (6.5 μL), and RNase R (#RNR07250, LGC Biosearch Technologies, 20 U/μL, 6.5 μL) were added and the reaction mix was incubated in a thermocycler at 37 °C for 1 h. Products were purified using a Monarch® RNA Cleanup Kit (500 μg) (#T2050L, New England Biolabs), following the manufacturer’s recommendations, and ion-pairing reversed-phase HPLC (details in the *HPLC* section). The circularity of the RNA was confirmed by RNase R digest as follows. To the RNA (*circ-iSyn-EGFP* or 5′-AF647 *iSyn-EGFP* – linear control, 1 μM, 2 μL), water (1.5 μL), 10X RNase R Reaction Buffer (0.5 μL), and RNase R (#RNR07250, LGC Biosearch Technologies, 20 U/μL, 1.0 μL) were added and the reaction mix was incubated in a thermocycler at 37 °C for 30 min. Next, Na_2_EDTA (0.1 M solution in water, pH 7.5, 1.0 μL) and water (2.0 μL) were added, and the mixture was analysed using on-chip electrophoresis (details in the *Automated on-chip electrophoresis* section).

### Determination of IVT yields, priming efficiencies and transcript heterogeneities

IVT yields and priming efficiencies were determined based on three independently replicated primed in vitro transcription reactions, each in a 20 μL scale (carried out as described in In vitro *transcription priming* and In vitro *transcription (non-primed)* sections, but without downstream HPLC purification and for the titration experiment with varying concentration of CleaN3 – 2, 4, 6, 8, and 10 mM). For the titration experiment, the *tiEMCV-EGFP* template (with A-inserted φ6.5 promoter) was used. For determining IVT yields and priming efficiencies, 5 DNA templates (*tCLuc* – CLuc AG Control Template, #N2078AVIAL, New England Biolabs, *tiEMCV-EGFP* (with A-inserted φ6.5 promoter), *tiEMCV-EGFP (φ6.5)*, *tiEMCV-EGFP (φ2.5)*, and *tiEMCV-Cas9* (with A-inserted φ6.5 promoter)) and 2 IVT primers (CleanCap® Reagent AG, #N-7113-1, TriLink Biotechnologies and CleaN3) were used. The IVT yields were determined based on the total volume and absorbance measured using a NanoDrop 2000 spectrophotometer (Thermo Scientific) of the eluate after the cleanup step. After the cleanup, transcripts were cleaved using DNAzyme 10–23 as follows. Eluate (100 μL), *DNAzyme Dz_10–23_1* (for *iEMCV-EGFP* transcripts), *Dz_10–23_2* (for *iEMCV-Cas9* transcripts), or *Dz_10–23_3* (for *CLuc-control* transcripts) (50 μM, 18 μL), and Tris-HCl buffer, pH 7.5 (250 μM in water, 36 μL) were mixed. The mixture was incubated at 95 °C for 1 min and cooled down to 37 °C (0.1 °C/s). Next, rCutSmart™ Buffer (#B6004S, New England Biolabs, 18 μL) and Quick CIP (#M0525S, New England Biolabs, 4.5 μL) were added, and the mixture was incubated at 37 °C for 30 min. Afterwards, Na_2_EDTA, pH 7.5 (0.5 M in water, 20 μL) was added, and the mixture was cleaned up using a Monarch® RNA Cleanup Kit (500 μg) (#T2050L, New England Biolabs), following the manufacturer’s recommendations. Eluates collected after the cleanup step were freeze-dried, redissolved in 12 μL of buffer containing 50 mM TEA_2_EDTA, 90 mM triethylammonium acetate, pH 7.0 in water and analysed using LC-MS (details in the *LC-MS* section). The IVT priming efficiencies and transcript heterogeneities were determined based on the integrals of *A*_260_ peaks corresponding to fragments of primed and non-primed transcripts or IVT side products and their extinction coefficients at 260 nm. The experiment was carried out in three experimental replicates. Data were processed using GraphPad Prism 10.0.1 (GraphPad Software). Statistical significance was calculated using two-way ANOVA followed by Šídák’s multiple comparisons test (comparisons of IVT primers for different DNA templates with A-inserted φ6.5 promoter), one-way ANOVA followed by Dunnett’s multiple comparisons test (comparisons of T7 promoters for IVTs primed with CleaN3), and one-way ANOVA followed by Tukey’s multiple comparisons test (comparisons of CleaN3 concentrations in the titration experiment for IVT priming optimisation).

### 5′ labelling of CleaN3-primed mRNA

5′-CleaN3 *iEMCV-EGFP* mRNA was labelled at the 5′-end with AF647 dye through SPAAC. To freeze-dried 5′-CleaN3 *iEMCV-EGFP* mRNA (585 μg, 1.0 nmol, 1 equiv.), water (15 μL), and DBCO-AF647 solution (#CLK-1302 A-1, Jena Bioscience, 20 mM in DMSO, 5.0 μL, 100 equiv.) were added. The reaction mixture was incubated at 37 °C for 1 h. The labelled mRNA was purified using a Monarch® RNA Cleanup Kit (500 μg) (#T2050L, New England Biolabs), following the manufacturer’s recommendations, and ion-pairing reversed-phase HPLC (details in the *HPLC* section), followed by desalting (details in the *nucleic acid desalting* section) to isolate labelled product (363 μg, 0.62 nmol, 62% yield). For the time-course study, 2 μL aliquots were taken from the reaction mixture after 15 min, 30 min, and 60 min, diluted with water (48 μL), cleaned up using a Monarch® RNA Cleanup Kit (50 μg) (#T2040L, New England Biolabs), following the manufacturer’s recommendations, and analysed using HPLC (details in the *HPLC* section). Timepoint 0 min corresponds to a similarly prepared sample but without the addition of DBCO-AF647 solution. The same protocol was used for labelling *iHCV-EGFP* and *iSyn-EGFP* mRNAs bearing 5′-CleaN3 modification.

### Influence of modifications and stress conditions on mRNA translation in HEK293 cells

For comparison of translation efficiency in HEK293 cells, 5′-triphosphate, 5′-CleaN3, and 5′-AF647 *iHCV-EGFP*, 5′-AF647 *iHCV-EGFP* (100% m^1^Ψ and 5% m^6^A variants), 5′-CleanCap *EGFP* (100% m^1^Ψ variant), 5′-AF647 *iSyn-EGFP*, *circ-iSyn-EGFP*, and 5′-CleanCap *mCherry* (transfection control and cap-dependent translation reference for stress experiment) mRNAs and 1 mM sodium citrate buffer, pH 6.4 (mock transfection) were used. All the mRNAs were HPLC-purified, except the 5′-triphosphate *iHCV-EGFP* sample for comparing the influence of HPLC purification on translation efficiency that was only cleaned up using a Monarch® RNA Cleanup Kit (500 μg) (#T2050L, New England Biolabs). One day prior to transfection, 2 × 10^4^ HEK293 cells per well were seeded in a 96-well transparent polystyrene flat bottom plate (#3595, Corning). To prepare each transfection mixture, the mRNA with 5% 5′-CleanCap *mCherry* spike-in (or 50% 5′-AF647 *iHCV-EGFP*:50% 5′-CleanCap *mCherry* for stress experiment) (1 μM, 0.8 μL) or the citrate buffer (0.8 μL) were diluted in Opti-MEM (#31985062, Thermo Fisher Scientific, 9.2 μL). Separately, Lipofectamine™ MessengerMAX™ Transfection Reagent (#LMRNA001, Thermo Fisher Scientific, 1.1 μL) was diluted in Opti-MEM (#31985062, Thermo Fisher Scientific, 8.9 μL) and incubated for 10 min at room temperature. Then, to the diluted mRNA/buffer solutions, the diluted lipofectamine solution (10.0 μL) was added and incubated for 5 min at room temperature. The transfection mixtures (15 μL) were then each added to 135 μL of DMEM, high glucose, GlutaMAX™ Supplement, pyruvate (#31966021, Thermo Fisher Scientific) medium containing glucose (4.5 g/L), GlutaMAX™ Supplement (862 mg/L), sodium pyruvate (110 mg/L), sodium bicarbonate (3.7 g/L), and phenol red (15 mg/L), supplemented with 10% (v/v) fetal bovine serum (#A5209402, Thermo Fisher Scientific). Then, the cell culture medium was exchanged with the prepared transfection solution in medium (100 μL/well) to initiate the transfection. For the stress experiment, sodium arsenite (#S7400-100G, Sigma-Aldrich) solution was added to the medium to the final concentration of 40 μM in the transfection mixture. After 24 h of incubation, cells were detached by incubating with TrypLE™ Express Enzyme (1X), no phenol red (#12604013, Thermo Fisher Scientific, 50 μL) for 10 min at 37 °C and vigorously pipetted with PBS (#10010023, Thermo Fisher Scientific) containing 2% (v/v) fetal bovine serum (#A5209402, Thermo Fisher Scientific) and 2 mM EDTA (#E4884-100G, Sigma-Aldrich) (100 μL). Harvested cells were analysed using flow cytometry as described in the *Flow cytometry* section. The experiments were carried out in three experimental replicates. Raw data were processed using FlowJo 10.8 Software (BD Life Sciences) and GraphPad Prism 10.0.1 (GraphPad Software).

### Assessment of the mRNA stability in HEK293 cells

To assess the influence of the 5′-modifications on the mRNA stability, HEK293 cells were electroporated with 5′-triphosphate, 5′-CleaN3, and 5′-AF647 *iHCV-EGFP* mRNAs using the Neon™ Transfection System (Thermo Fisher Scientific) fitted with 100 μL tips (#MPK10096, Thermo Fisher Scientific). The electroporation was performed according to the manufacturer’s protocol with slight modifications. Briefly, each mRNA solution (1 μg/μL, 2.5 μL) was mixed with buffer R (60 μL). Next, 9 × 10^6^ HEK293 cells were suspended in buffer R (225 μL). Then, the cell suspension (62.5 μL) was added to each of the diluted mRNAs (resulting in a total volume of 125 μL, 2 × 10^4^ cells/μL, 20 ng/μL mRNA). After mixing, cell suspension (100 μL) was immediately electroporated (1100 V pulse voltage, 20 ms pulse width, 2 pulses) and diluted in DMEM, high glucose, HEPES, no phenol red (#21063029, Thermo Fisher Scientific) medium containing glucose (4.5 g/L), L-glutamine (584 mg/mL), sodium bicarbonate (3.7 g/L), and HEPES (25 mM), supplemented with 10% (v/v) fetal bovine serum (#A5209402, Thermo Fisher Scientific) (400 μL). Electroporated cells were split into aliquots (5 × 100 μL) and incubated at 37 °C. At time points 0, 1, 2, 4, and 8 h, the cells were pelleted (500 × g, 2 min), washed with PBS (#10010023, Thermo Fisher Scientific, 100 μL), pelleted (500 × g, 2 min), and snap frozen in liquid nitrogen. Total mRNA from frozen cell pellets was extracted using a Monarch^®^ Total RNA Miniprep Kit (#T2010S, New England Biolabs), following the manufacturer’s protocol for cultured mammalian cells. The experiment was carried out in three experimental replicates. To quantify the 5′-modified *iHCV-EGFP* mRNAs, RT-qPCR was performed using Luna® Universal One-Step RT-qPCR Kit (#E3005X, New England Biolabs). For a single 20 μL reaction, water (5.4 μL), 2X Luna Universal One-Step Reaction Mix (10 μL), 20X Luna WarmStart® RT Enzyme Mix (1.0 μL), *EGFP_F* primer (10 μM, 0.8 μL), *EGFP_R* primer (10 μM, 0.8 μL), and extracted total mRNA (2 μL, 20 ng/μL) were mixed. The reaction mix was cycled in a Bio-Rad CFX96 C1000 Real-Time PCR Detection System under the control of CFX Manager 3.1 (Bio-Rad) software, using SYBR scan mode, and the following protocol: 55 °C, 10 min; 95 °C, 1 min; 35 cycles of 95 °C, 10 s, 60 °C, 30 s. For data normalisation, GAPDH mRNA was also analysed with *GAPDH_F and GAPDH_R* primers using the same conditions. RT-qPCR for all samples was carried out in three technical replicates. The threshold fluorescence intensity to determine the quantification cycle (*Cq*) was set at 800 RFU. The fold change in mRNA amount at a given timepoint (*tx*) relative to timepoint 0 (*t0*) was calculated as 2^-ΔΔCq^, where ΔΔ*Cq* represents GAPDH-corrected difference in quantification cycle values between *tx* and *t0*, i.e., ΔΔ*Cq* = (*Cq*_EGFP, tx_ − *Cq*_GAPDH, tx_) − (*Cq*_EGFP, t0_ − *Cq*_GAPDH, t0_). The data were then plotted, the one-phase decay model was fitted to it, and the half-lives were calculated using GraphPad Prism 10.0.1 (GraphPad Software). Statistical significance was calculated using one-way ANOVA followed by Tukey’s multiple comparisons test.

### Assessment of mRNA immunogenicity

To assess the immunogenicity of 5′-triphosphate, 5′-CleaN3, and 5′-AF647 (non-body-modified, or 100% m^1^Ψ, and 5% m^6^A versions) *iHCV-EGFP* mRNAs, 5′-CleanCap *EGFP* (100% U and 100% m^1^Ψ versions) mRNAs as well as poly I:C (#P1530-25MG, Sigma-Aldrich) control in mature dendritic cells (mDCs) derived from MUTZ-3 cell line, LEGENDplex™ Hu Anti-Virus Response Panel 1 (13-plex) w/VbP V02 (#741270, BioLegend) was used. First, to generate dendritic cells, 1 × 10^6^ MUTZ-3 cells per well were seeded in 6-Well CytoOne® Plate, TC-Treated (#CC7682-7506, Starlab) in 2 mL/well of 1:3 (v/v) conditioned to fresh MEM α, nucleosides (#12571063, Thermo Fisher Scientific) medium containing L-glutamine (292 mg/L), ribonucleosides (10 mg/L each), deoxyribonucleosides (10 mg/L each), sodium bicarbonate (2.2 g/L), and phenol red (10 mg/L), supplemented with 20% (v/v) heat-inactivated fetal bovine serum (#A5209402, Thermo Fisher Scientific), GM-CSF (#G5035-5UG, Sigma-Aldrich, 50 ng/mL), and IL-4 (#I4269-5UG, Sigma-Aldrich, 20 ng/mL). The cells were incubated at 37 °C and 5% CO_2_ for 7 days. Half of the medium was replaced with freshly prepared (containing the same concentration of the supplements) after 2, 4, and 6 days. After 7 days, to induce the maturation, half of the medium was replaced with MEM α, nucleosides (#12571063, Thermo Fisher Scientific) medium containing L-glutamine (292 mg/L), ribonucleosides (10 mg/L each), deoxyribonucleosides (10 mg/L each), sodium bicarbonate (2.2 g/L), and phenol red (10 mg/L), supplemented with 20% (v/v) heat-inactivated fetal bovine serum (#A5209402, Thermo Fisher Scientific), and TNFα (#H8916-10UG, Sigma-Aldrich, 24 ng/mL, final concentration in the 1:1 mixture 12 ng/mL) and the cells were incubated at 37 °C and 5% CO_2_ for 2 days. Next, to assess the immunogenicity of mRNAs, the generated mDCs were electroporated using the Neon™ Transfection System (Thermo Fisher Scientific) fitted with 10 μL tips (#MPK1096, Thermo Fisher Scientific). The electroporation was performed according to the manufacturer’s protocol with slight modifications. Briefly, 4 × 10^6^ mDCs were suspended in buffer R (200 μL). Next, 2.0 μL of each mRNA solution (10 μM), or 1 mM sodium citrate buffer, pH 6.4 (mock electroporation), or poly I:C (#P1530-25MG, Sigma-Aldrich, 5 μg/μL), were mixed with buffer R (18 μL). Then, the cell suspension (20 μL) was added to each of the diluted samples (resulting in a total volume of 40 μL, 1 × 10^4^ cells/μL). After mixing, cell suspension (10 μL) was immediately electroporated (1400 V pulse voltage, 20 ms pulse width, 1 pulse) and diluted in MEM α, nucleosides (#12571063, Thermo Fisher Scientific) medium containing L-glutamine (292 mg/L), ribonucleosides (10 mg/L each), deoxyribonucleosides (10 mg/L each), sodium bicarbonate (2.2 g/L), and phenol red (10 mg/L), supplemented with 20% (v/v) heat-inactivated fetal bovine serum (#A5209402, Thermo Fisher Scientific) (90 μL). The electroporated cells were incubated in a 96-well transparent polystyrene flat bottom plate (#3595, Corning) placed in a humidified incubator maintained at 37 °C and 5% CO_2_. After 24 h of incubation, the plate was centrifuged (500 × *g*, 5 min), and the supernatant (25 μL) was collected for the assay. The cytokine concentrations were determined using bead-based LEGENDplex™ Hu Anti-Virus Response Panel 1 (13-plex) w/VbP V02 (#741270, BioLegend) kit and flow cytometry (details in the *Flow cytometry* section), following the manufacturer’s protocol. All beads were recorded, resulting in >30000 gated bead events per well. The experiment was carried out in three experimental replicates. Raw data were analysed using Biolegend LEGENDplex™ 2024-06-15 software (Qognit) and GraphPad Prism 10.0.1 (GraphPad Software). Statistical significance was calculated using one-way ANOVA followed by Tukey’s multiple comparisons test.

### Effect of mRNA 5′ modifications on HEK293 cell viability

To assess the impact of the mRNA 5′ modifications on cell viability using CellTiter-Glo® Luminescent Cell Viability Kit (#G7572, Promega), HEK293 cells were transfected with *iHCV-EGFP* mRNAs bearing 5′-triphosphate, 5′-CleaN3, and 5′-AF647, as well as *EGFP* mRNA bearing 5′-CleanCap and 1 mM sodium citrate buffer, pH 6.4 (mock). One day prior to transfection, 2 × 10^4^ HEK293 cells per well were seeded in a 96-well flat clear bottom white polystyrene plate (#3610, Corning). To prepare the transfection mixtures, the mRNAs (1 μM, 0.8 μL) and the citrate buffer (0.8 μL) were diluted in Opti-MEM (#31985062, Thermo Fisher Scientific, 9.2 μL). Separately, Lipofectamine™ MessengerMAX™ Transfection Reagent (#LMRNA001, Thermo Fisher Scientific, 6.0 μL) was diluted in Opti-MEM (#31985062, Thermo Fisher Scientific, 50.0 μL) and incubated for 10 min at room temperature. Then, to each of the diluted mRNA/buffer solutions, the diluted lipofectamine solution (10.0 μL) was added and incubated for 5 min at room temperature. The transfection mixtures (15 μL) were then each added to 135 μL of DMEM, high glucose, GlutaMAX™ Supplement, pyruvate (#31966021, Thermo Fisher Scientific) medium containing glucose (4.5 g/L), GlutaMAX™ Supplement (862 mg/L), sodium pyruvate (110 mg/L), sodium bicarbonate (3.7 g/L), and phenol red (15 mg/L), supplemented with 10% (v/v) fetal bovine serum (#A5209402, Thermo Fisher Scientific). Then, the cell culture medium was exchanged with the prepared transfection solution in medium (100 μL/well), and the cells were incubated for 24 h. To assess cell viability, the CellTiter-Glo® Luminescent Cell Viability Kit (#G7572, Promega) was used following the manufacturer’s protocol. The plate was read using a FLUOstar Omega (BMG LABTECH) microplate reader under the control of Omega 5.10 R2 (BMG LABTECH) software. For data acquisition, luminescence mode was used with the following parameters: “lens” filter, 1 s integration. The experiment was carried out in three experimental replicates. Data were processed using GraphPad Prism 10.0.1 (GraphPad Software). Statistical significance was calculated using one-way ANOVA followed by Šídák’s multiple comparisons test.

### Comparison of IRES-driven translation in human cell lines

To compare the efficiency of IRES-driven translation across human cell lines, HEK293, HeLa, HepG2, and HCT-116 cells were transfected with 5′-AF647 *iSyn-EGFP*, 5′-AF647 *iHCV-EGFP*, and 5′-AF647 *iEMCV-EGFP* mRNAs and analysed by flow cytometry. 24 h before transfection, 2 × 10^4^ HEK293, HeLa, HepG2, or HCT-116 cells per well were seeded in a 96-well transparent polystyrene flat bottom plate (#3595, Corning). To prepare each transfection mixture, the mRNA (1 μM, 0.8 μL) was diluted in Opti-MEM (#31985062, Thermo Fisher Scientific, 9.2 μL). Separately, Lipofectamine™ MessengerMAX™ Transfection Reagent (#LMRNA001, Thermo Fisher Scientific, 1.1 μL) was diluted in Opti-MEM (#31985062, Thermo Fisher Scientific, 8.9 μL) and incubated for 10 min at room temperature. Then, to the diluted mRNA, the diluted lipofectamine solution (10.0 μL) was added and incubated for 5 min at room temperature. The transfection mixtures (15 μL) were then each added to 135 μL of DMEM, high glucose, GlutaMAX™ Supplement, pyruvate (#31966021, Thermo Fisher Scientific) medium containing glucose (4.5 g/L), GlutaMAX™ Supplement (862 mg/L), sodium pyruvate (110 mg/L), sodium bicarbonate (3.7 g/L), and phenol red (15 mg/L), supplemented with 10% (v/v) fetal bovine serum (#A5209402, Thermo Fisher Scientific). Then, the cell culture medium was exchanged with the prepared transfection solution in medium (100 μL/well) to initiate the transfection. After 24 h of incubation, cells were harvested and analysed using flow cytometry (as described in the *Influence of modifications and stress conditions on mRNA translation in HEK293 cells* section). The experiment was carried out in three experimental replicates. Raw data were processed using FlowJo 10.8 (BD Life Sciences) and GraphPad Prism 10.0.1 (GraphPad Software). The mRNA productivity was calculated as the ratio of the expressed EGFP fluorescence to AF647 fluorescence (reflecting the total mRNA uptake). The same data were used for the assessment of mRNA dose – EGFP expression relationships and cell size analysis.

### Flow cytometry analysis of the mRNA transfection and expression over time

To monitor the transfection and translation of 5′-AF647 *iSyn-EGFP* mRNA over time, HeLa cells were transfected and harvested for flow cytometry in a time-course assay. 24 h before the transfection, 2 × 10^4^ HeLa cells per well were seeded in a 96-well transparent polystyrene flat bottom plate (#3595, Corning). The transfections (at each timepoint before harvesting – 24, 12, 8, 5, 3, 2, 1 h) were performed as described in the *Comparison of IRES-driven translation in human cell lines* section. 24, 12, 8, 5, 3, 2, and 1 h after transfection, transfected as well as non-transfected (corresponding to the timepoint 0 h) cells were harvested and analysed using flow cytometry (as described in *Influence of modifications and stress conditions on mRNA translation in HEK293 cells* and *Flow cytometry* sections). The experiment was carried out in three experimental replicates. Raw data were processed using FlowJo 10.8 Software (BD Life Sciences) and GraphPad Prism 10.0.1 (GraphPad Software).

### 24 h transfection and expression time-lapse

To investigate the transfection and translation over time using microscopy, time-lapse imaging of HeLa cells incubated with the transfection solution was performed. One day prior to transfection, 2 × 10^4^ HeLa cells per well were seeded in a µ-Slide 8 Well high ibiTreat (#80806, Ibidi). Directly before the transfection, the μ-Slide was placed on the microscope stage inside a preconditioned (37 °C, 5% CO_2_), humidified incubator chamber. To prepare the transfection mixture, 5′-AF647 *iSyn-EGFP* mRNA (1 μM, 0.8 μL) was diluted in Opti-MEM (#31985062, Thermo Fisher Scientific, 9.2 μL). Separately, Lipofectamine™ MessengerMAX™ Transfection Reagent (#LMRNA001, Thermo Fisher Scientific, 1.2 μL) was diluted in Opti-MEM (#31985062, Thermo Fisher Scientific, 10.0 μL) and incubated for 10 min at room temperature. Then, to the diluted mRNA solution, the diluted lipofectamine solution (10.0 μL) was added and incubated for 5 min at room temperature. The transfection mixture (20.0 μL) was then mixed with 380 μL of FluoroBrite™ DMEM (#A1896701, Thermo Fisher Scientific) containing glucose (4.5 g/L) and sodium bicarbonate (3.7 g/L), supplemented with 10% (v/v) fetal bovine serum (#A5209402, Thermo Fisher Scientific), sodium pyruvate (#11360070, Thermo Fisher Scientific, 1X, 110 mg/L), and GlutaMAX™ Supplement (#35050061, Thermo Fisher Scientific, 1X, 434 mg/L). Then, the cell culture medium in the imaged well was exchanged with the prepared transfection solution in medium (300 μL), and immediately after that, the image acquisition was started. Each 220.88 μm × 220.88 μm frame was captured every 5 min (289 frames in total), and the collected dataset was subsequently processed (details in the *Microscopy* section).

### Single transfection event and cell division time-lapse

To investigate the single transfection events and the transfected mRNA distribution during cell division, time-lapse imaging of HeLa cells incubated with the transfection solution was performed. One day prior to transfection, 2 × 10^4^ HeLa cells per well were seeded in a µ-Slide 8 Well high ibiTreat (#80806, Ibidi). Directly before the transfection, the μ-Slide was placed on the microscope stage inside a preconditioned (37 °C, 5% CO_2_), humidified incubator chamber. The transfection mixture was prepared as described in the *24 h transfection and expression time-lapse* section. The cell culture medium in the imaged well was exchanged with the prepared transfection solution in medium (300 μL), and immediately after that, the image acquisition was started. Each 220.88 μm × 220.88 μm frame was captured every 1 min for 8 h (481 frames in total), and the collected dataset was subsequently processed (details in the *Microscopy* section).

### Transfected cell video

To acquire the real-time video of transfected cell, HeLa cells were seeded and transfected with 5′-AF647 *iSyn-EGFP* mRNA as described in the *Live cell 3D imaging* section. The image acquisition was started 6 h post-transfection, capturing 69.33 μm × 69.33 μm frames every 100 ms for 10 s (100 frames in total). The video was rendered from the acquired frames after applying bleach correction as described in the *Microscopy* section.

### Live cell 3D imaging and colocalisation

To investigate the mRNA localisation in live HeLa cells, 3D imaging of the cells transfected with 5′-AF647 *iSyn-EGFP* mRNA, 5′-CleanCap *EGFP mRNA*, and untransfected was performed. One day prior to transfection, 1 × 10^4^ HeLa cells per well were seeded in a poly-D-lysine (#A3890401, Thermo Fisher Scientific) treated µ-Slide 8 Well high Glass Bottom (#80807, Ibidi). To stain early endosomes, the CellLight™ Early Endosomes-RFP, BacMam 2.0 (#C10587, Thermo Fisher Scientific) reagent was added at a ratio of 1:10 to the medium during seeding. To prepare the transfection mixture, mRNA (1 μM, 1.5 μL) was diluted in Opti-MEM (#31985062, Thermo Fisher Scientific, 17.3 μL). Separately, Lipofectamine™ MessengerMAX™ Transfection Reagent (#LMRNA001, Thermo Fisher Scientific, 1.05 μL) was diluted in Opti-MEM (#31985062, Thermo Fisher Scientific, 18.0 μL) and incubated for 10 min at room temperature. Then, to the diluted mRNA solution, the diluted lipofectamine solution (18.8 μL) was added and incubated for 5 min at room temperature. The transfection mixture (37.6 μL) was then mixed with 712 μL of FluoroBrite™ DMEM (#A1896701, Thermo Fisher Scientific) containing glucose (4.5 g/L) and sodium bicarbonate (3.7 g/L), supplemented with 10% (v/v) fetal bovine serum (#A5209402, Thermo Fisher Scientific), sodium pyruvate (#11360070, Thermo Fisher Scientific, 1X, 110 mg/L), and GlutaMAX™ Supplement (#35050061, Thermo Fisher Scientific, 1X, 434 mg/L). Then, the cell culture medium was exchanged with the prepared transfection solution in medium (300 μL), and the cells were incubated at 37 °C, 5% CO_2_. 6 h after transfection initiation, the cell culture medium was replaced with staining solutions. For nucleus and late endosome/lysosome staining, cells were incubated in 300 μL of FluoroBrite™ DMEM (#A1896701, Thermo Fisher Scientific) containing glucose (4.5 g/L) and sodium bicarbonate (3.7 g/L), supplemented with 10% (v/v) fetal bovine serum (#A5209402, Thermo Fisher Scientific), sodium pyruvate (#11360070, Thermo Fisher Scientific, 1X, 110 mg/L), and GlutaMAX™ Supplement (#35050061, Thermo Fisher Scientific, 1X, 434 mg/L), mixed with Hoescht 33342 (#H3570, Thermo Fisher Scientific, 1 μg/mL) and LysoTracker™ Red (#L7528, Thermo Fisher Scientific, 75 nM) for 30 min at 37 °C. For nucleus and ER staining, cells were incubated in 300 μL of HBSS, calcium, magnesium, no phenol red (#14025092, Thermo Fisher Scientific) mixed with Hoescht 33342 (#H3570, Thermo Fisher Scientific, 1 μg/mL) and ER-Tracker™ Red (#34250, Thermo Fisher Scientific, 1 μM) for 30 min at 37 °C. For nucleus only staining (including cells prestained with the CellLight™ Early Endosomes-RFP, BacMam 2.0 reagent), cells were incubated in 300 μL of FluoroBrite™ DMEM (#A1896701, Thermo Fisher Scientific) containing glucose (4.5 g/L) and sodium bicarbonate (3.7 g/L), supplemented with 10% (v/v) fetal bovine serum (#A5209402, Thermo Fisher Scientific), sodium pyruvate (#11360070, Thermo Fisher Scientific, 1X, 110 mg/L), and GlutaMAX™ Supplement (#35050061, Thermo Fisher Scientific, 1X, 434 mg/L), mixed with Hoescht 33342 (#H3570, Thermo Fisher Scientific, 1 μg/mL) for 30 min at 37 °C. After incubation, the cells were immediately imaged (without exchanging the medium or washing). Imaging was performed as described in the *Microscopy* section, collecting 12 μm *z*-stacks of 69.33 μm × 69.33 μm tiles with 250 nm spacing. For the image rendering and quantitative analysis of colocalisation, the 3D datasets were cropped in *x*, *y*, *z*, deconvolved, corrected for chromatic aberration, and segmented, as described in the *Microscopy* section.

### FISH

Fluorescence in situ hybridisation was carried out in fixed HeLa cells using pooled 20 nt oligonucleotide DNA probes targeting EGFP mRNA sequence (*FISH_EGFP_1-4*). For fluorescent labelling, the solid-phase synthesised 5′-amino modifier C6 oligonucleotides (40 mM, 2.5 μL each) were pooled. Next, sodium bicarbonate buffer (400 mM, pH 8.3, 10 μL) and ATTO 565 NHS ester (#AD 565-35, ATTO-TEC, 100 mM in DMSO, 20 μL, 5 equiv.) were added and the reaction mixture was incubated for 2 h at room temperature. Afterwards, the labelled oligonucleotides were doubly purified using a Monarch® RNA Cleanup Kit (500 μg) (#T2050L, New England Biolabs), following the manufacturer’s recommendations. The completion of the labelling was confirmed via LC-MS analysis. For investigating the colocalization of the AF647 with the 5′-modified mRNAs coding for EGFP, 1 × 10^4^ HeLa cells per well were seeded in a poly-D-lysine (#A3890401, Thermo Fisher Scientific) treated µ-Slide 8 Well high Glass Bottom (#80807, Ibidi). 6 h after seeding, the cells were transfected with 5′-AF647 *iSyn-EGFP*, 5′-CleanCap *EGFP* mRNA, or left untransfected. To prepare the transfection mixture, mRNA (1 μM, 1.5 μL) was diluted in Opti-MEM (#31985062, Thermo Fisher Scientific, 17.3 μL). Separately, Lipofectamine™ MessengerMAX™ Transfection Reagent (#LMRNA001, Thermo Fisher Scientific, 1.05 μL) was diluted in Opti-MEM (#31985062, Thermo Fisher Scientific, 18.0 μL) and incubated for 10 min at room temperature. Then, to the diluted mRNA solution, the diluted lipofectamine solution (18.8 μL) was added and incubated for 5 min at room temperature. The transfection mixture (37.6 μL) was then mixed with 712 μL of FluoroBrite™ DMEM (#A1896701, Thermo Fisher Scientific) containing glucose (4.5 g/L) and sodium bicarbonate (3.7 g/L), supplemented with 10% (v/v) fetal bovine serum (#A5209402, Thermo Fisher Scientific), sodium pyruvate (#11360070, Thermo Fisher Scientific, 1X, 110 mg/L), and GlutaMAX™ Supplement (#35050061, Thermo Fisher Scientific, 1X, 434 mg/L). Then, the cell culture medium in the imaged well was exchanged with the prepared transfection solution in medium (300 μL) and the cells were incubated at 37 °C, 5% CO_2_. 6 h (for 5′-AF647 *iSyn-EGFP* and 5′-CleanCap *EGFP* mRNA), 12 h (for 5′-AF647 *iSyn-EGFP* mRNA), and 24 h (for 5′-AF647 *iSyn-EGFP* mRNA) post-transfection, transfected and untransfected (control) cells were washed with PBS (#10010023, Thermo Fisher Scientific, 300 μL), fixed by incubating with 4% (w/v) formaldehyde (#10630813, Fisher Scientific) solution in PBS (300 μL) for 15 min at room temperature, rinsed 3 times with PBS (300 μL) and permeabilised by incubating with 0.5% (v/v) Triton X-100 (#T8787-50ML, Sigma-Aldrich) solution in PBS containing ribonucleoside vanadyl complex (#S1402S, New England Biolabs, 10 mM) for 15 min at room temperature. Afterwards, the cells were rinsed 3 times with PBS (300 μL), 1 time with SSC (#15557044, Thermo Fisher Scientific, 2X, 300 μL), and 1 time with 10% (v/v) formamide (#344206-100 ML, Merck) in 2X SSC. The hybridisation was carried out by incubating the cells with solution of the pooled, ATTO 565-labelled, oligo DNA probes (4 probes, 10 nM/probe) in 2X SSC containing 10% (w/v) dextran sulphate (#42867-5 G, Sigma-Aldrich), yeast tRNA (#AM7119, Thermo Fisher Scientific, 1 mg/mL), BSA (#B4287-5G, Sigma-Aldrich, 0.2 mg/mL), ribonucleoside vanadyl complex (10 mM), and 10% (v/v) formamide (160 μL), in a humidified incubator, for 16 h at 37 °C. Afterwards, the cells were incubated 2 times with 10% (v/v) formamide in 2X SSC (300 μL) for 30 min each time and rinsed with 2X SSC (300 μL). To stain the nuclei, the cells were incubated with DAPI (#D8417-5MG, Sigma-Aldrich, 300 nM) solution in 2X SSC for 5 min at room temperature and subsequently rinsed 3 times with 2X SSC (300 μL). Prior to imaging, the cells were covered with SlowFade™ Diamond Antifade Mountant (#S36967, Thermo Fisher Scientific). The imaging was performed as described in the *Microscopy* section, collecting 12 μm *z*-stacks of 69.33 μm x 69.33 μm tiles with 250 nm spacing. In total, data for 6 conditions (6 h, 12 h, and 24 h post-transfection, 5′-AF647 *iSyn-EGFP*, hybridised; 3 controls for evaluating FISH specificity and spectral bleed-through, i.e., 6 h post-transfection, 5′-CleanCap *EGFP* mRNA, hybridised, 6 h post-transfection, 5′-AF647 *iSyn-EGFP*, non-hybridised, and non-transfected, hybridised) and 3 fields of view per condition were collected. The quantitative analysis of colocalisation was performed on the 3D datasets after cropping in *x*, *y*, *z*, deconvolving, correcting for chromatic aberration, and segmenting, as described in the *Microscopy* section. Statistical significance was calculated using Kruskal-Wallis test followed by Dunn’s multiple comparisons test.

### Reporting summary

Further information on research design is available in the Nature Portfolio Reporting Summary linked to this article.

## Supplementary information

Supplementary Information

Description of Additional Supplementary Information

Supplementary Movie 1

Supplementary Movie 2

Supplementary Movie 3

Supplementary Movie 4

Supplementary Movie 5

Supplementary Movie 6

Reporting Summary

Transparent Peer Review file

## Source data

Source Data

## Data Availability

Raw microscopy data generated in this study have been submitted to Zenodo (10.5281/zenodo.14589630)^64^. Source data are provided with this paper.
