# Supplementary material for: Enhancing cap-independent translation of linear mRNA
Source: Nat Commun. 2025 Oct 16;16:9205. doi: 10.1038/s41467-025-64257-6 (PMC12532787; doi:10.1038/s41467-025-64257-6)

Table of Contents

Manufacturer's protocols **2**

Supplementary figures **3**

Supplementary Fig. 1 3

Supplementary Fig. 2 5

Supplementary Fig. 3 6

Supplementary Fig. 4 7

Supplementary Fig. 5 8

Supplementary Fig. 6 9

Supplementary Fig. 7 10

Supplementary Fig. 8 11

Supplementary Fig. 9 12

Supplementary Fig. 10 13

Supplementary Fig. 11 14

Nucleic acid sequences **15**

PCR primer sequences 15

DNAzyme sequences 16

FISH probe sequences 17

Other oligonucleotide sequences 17

Plasmid sequences 18

DNA template sequences 31

mRNA sequences 39

NMR and MS spectra **45**

Manufacturer's protocols

Kit/device/reagent name	Supplier	Catalogue number	Manufacturer's protocol
Agilent RNA 6000 Nano Kit	Agilent Technologies	5067-1511	link
Amicon® Ultra-4 30 kDa MWCO<br>Amicon® Ultra-4 3 kDa MWCO	Merck	UFC803096<br>UFC800396	link
Monarch® DNA Gel Extraction Kit	New England Biolabs	T1020L	link
NEB® 5-alpha Competent E. coli	New England Biolabs	C2987H	link
NEB® Stable Competent E. coli (High Efficiency)	New England Biolabs	C3040H	link
QIAprep® Spin Miniprep Kit	Qiagen	27104	link
Plasmid Giga Kit (5)	Qiagen	12191	link
E.Z.N.A.® Plasmid DNA Mini Kit II	Omega Bio-tek	D6945-01	link
Monarch® RNA Cleanup Kit	New England Biolabs	T2050L	link
Neon™ Transfection System 100 µL Kit<br>Neon™ Transfection System 10 µL Kit	Thermo Fisher Scientific	MPK10096<br>MPK1096	link
Monarch® Total RNA Miniprep Kit	New England Biolabs	T2010S	link
CellTiter-Glo® Luminescent Cell Viability Kit	Promega	G7572	link
LEGENDplex™ Hu Anti-Virus Response Panel 1 (13-plex) w/VbP V02	BioLegend	741270	link

Supplementary figures

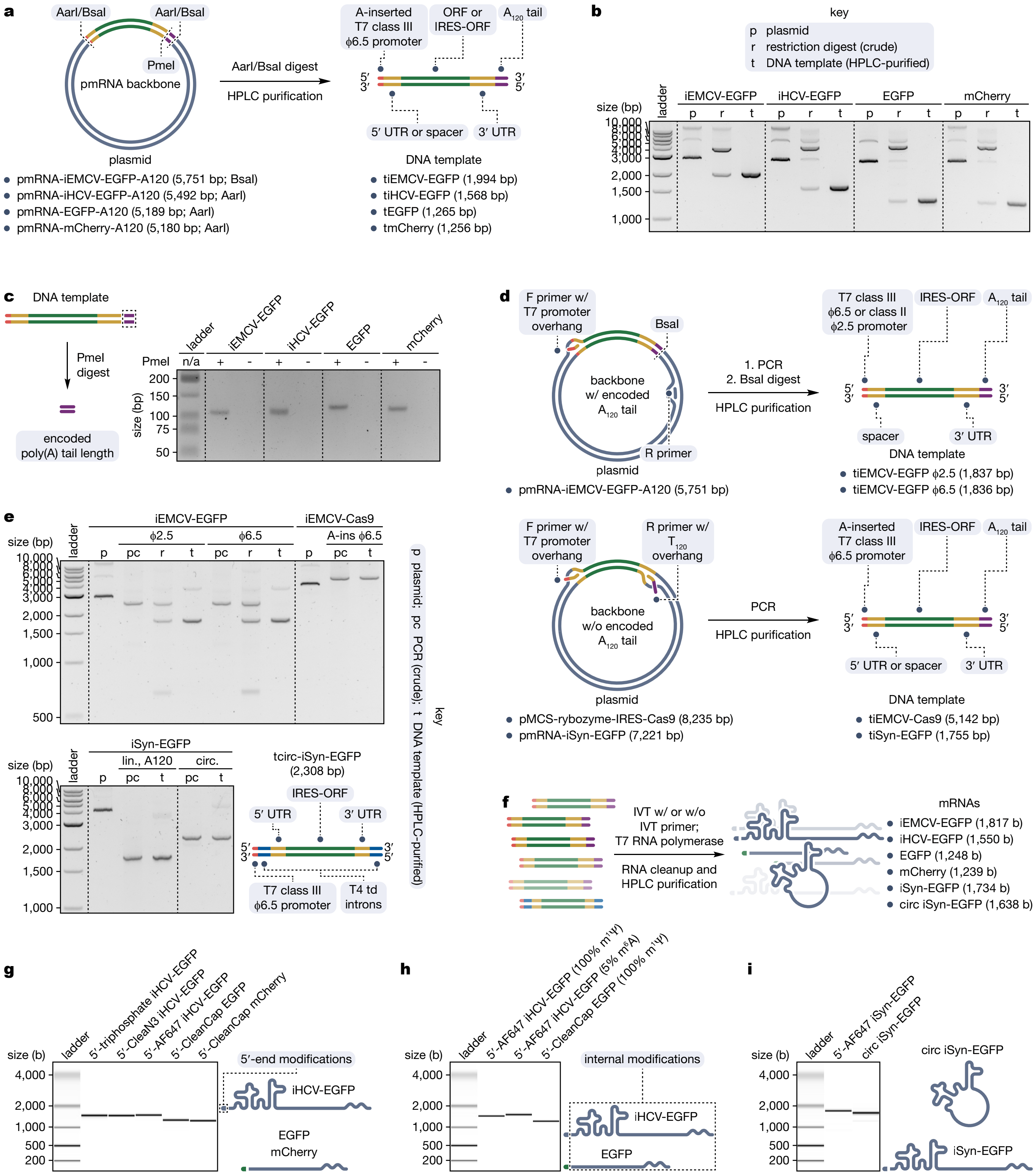

Supplementary Fig. 1 | Preparation of DNA templates and mRNAs. a, Preparation of tiEMCV-EGFP, tiHCV-EGFP, tEGFP, and tmCherry (containing A-inserted T7 class III φ6.5 promoter) DNA templates via restriction digest. **b,** Agarose gel electrophoresis of the plasmids, crude restriction digests, and HPLC-purified DNA templates prepared according to workflow shown in panel a. **c,** Long homopolymeric sequences in plasmids can undergo shortening in *E. Coli*. To verify the lengths of the plasmid-encoded poly(A) tails in DNA templates prepared via restriction digests, we performed PmeI digests and analysed the fragments using agarose gel electrophoresis. The results indicated no shortening of the encoded poly(A) tails in the analysed DNA templates. **d,** Preparation of tiEMCV-EGFP (containing T7 class II φ2.5 or T7 class III φ6.5 promoter) as well as tiEMCV-Cas9 and tiSyn-EGFP (both containing A-inserted T7 class III φ6.5 promoter) DNA templates via PCR and restriction digest or tail overhang PCR. **e,** Agarose gel electrophoresis of the plasmids, crude PCR

products, crude restriction digests, and HPLC-purified DNA templates prepared according to workflow shown in panel d. **f,** General workflow for preparation of mRNAs. **g,** Pseudo-gel image of 5′-triphosphate, 5′-CleaN3, and 5′-AF647 iHCV-EGFP, 5′-CleanCap EGFP, and 5′-CleanCap mCherry mRNAs. **h,** Pseudo-gel image of 5′-AF647 iHCV-EGFP and 5′-CleanCap EGFP mRNAs containing internal modifications (100% $m^1\Psi$ and 5% m^6A). **i,** Pseudo-gel image of 5′-AF647 iSyn-EGFP and circ iSyn-EGFP mRNAs. **b,c,e,g,h,i,** Data of one replicate. Source data are provided as a Source Data file.

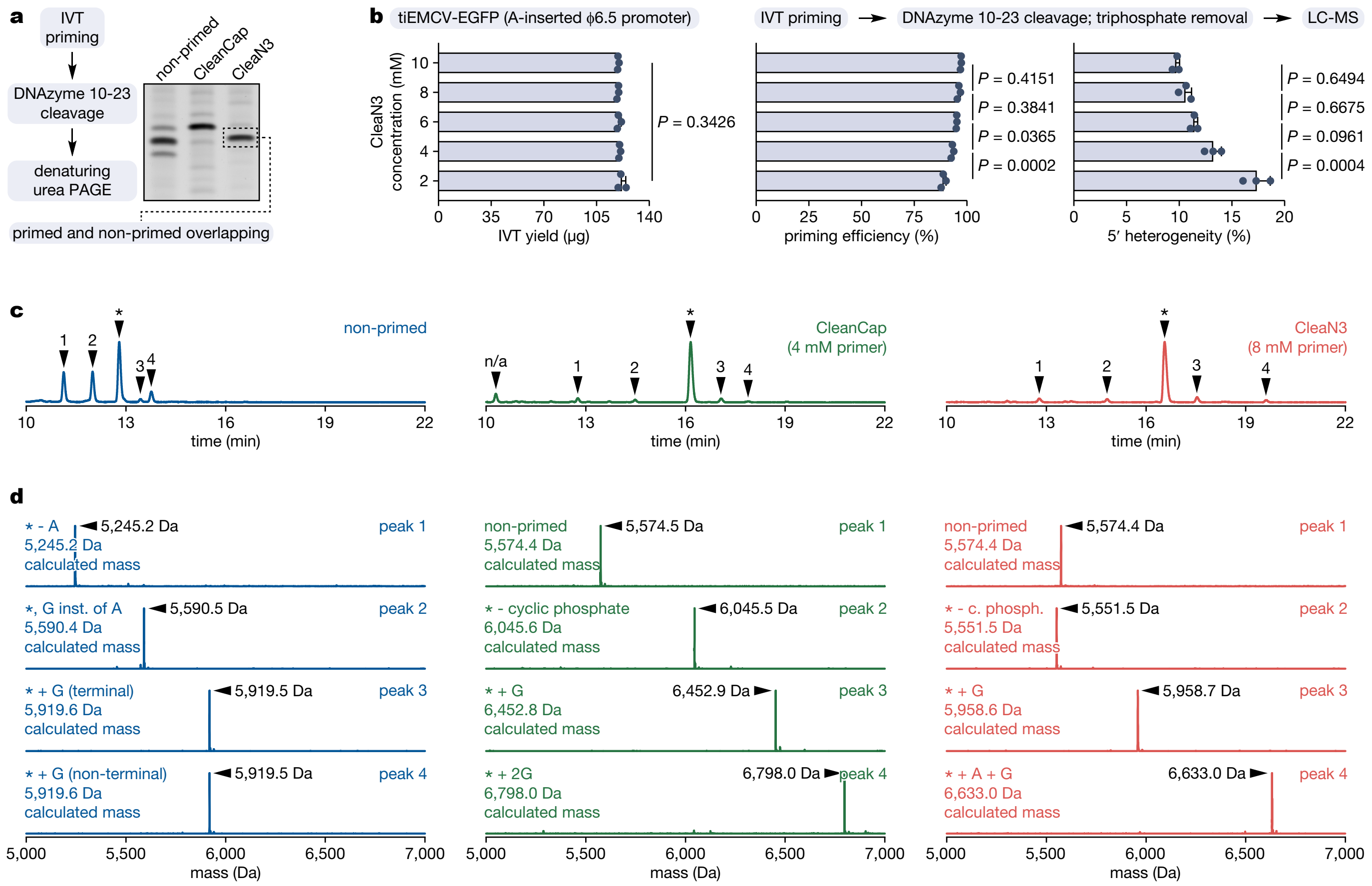

Supplementary Fig. 2 | IVT priming optimisation and transcript fragment analysis. a, To assess the priming efficiency and 5′-heterogeneity of transcripts primed with CleanCap and CleaN3, we trimmed the RNAs using DNAzyme 10-23 and analysed the obtained fragments using denaturing (7 M urea) 20% PAGE. For the CleanCap-primed IVT, the primed fragment separated well from its non-primed counterpart; however, for the CleaN3-primed reaction, both the primed and non-primed fragments migrated at the same height. This indicated that the analysis of the CleaN3-primed transcript fragments using denaturing PAGE can be unsuitable for the assessment of priming efficiency and transcript 5′-heterogeneity. **b,** To optimise the CleaN3-mediated IVT priming, we ran IVTs with varying concentrations of CleaN3 (2–10 mM) and analysed the reaction mixtures according to the workflow shown in Fig. 3a. The bar graphs show IVT yields (in amount of produced mRNA), priming efficiencies, and transcript 5′-heterogeneities as individual data points and the means ± s.d. of three experimental replicates. Statistical significance was calculated using one-way ANOVA followed by Tukey's multiple comparisons test. **c,** Representative HPLC chromatograms (absorbance at 260 nm, traces normalised to maximum) of the fragments of tiEMCV-EGFP (with A-inserted φ6.5 promoter)-templated transcripts primed with CleaN3 or CleanCap and non-primed. The numbers and asterisks mark peaks taken into account to calculate priming efficiencies and transcript 5′-heterogeneities. Mass spectra of the peaks marked with the numbers are shown in panel d. Mass spectra of the fragments of the main products of the *in vitro* transcriptions, marked with asterisks, are shown in Fig. 3b. **d,** Representative mass spectra (deconvolved, normalised to maximum) of fragments of the by-products of *in vitro* transcriptions together with masses calculated for anticipated by-product fragments. The fragments captioned "* **-** cyclic phosphate" arise from hydrolysis of the 2′,3′-cyclic phosphates at the ends of the fragments of the main products of the IVTs after DNAzyme 10-23 cleavage. Thus, in the calculations of the heterogeneities and priming efficiencies in Fig. 3c, we treated these peaks as fragments of the main products of the IVTs. **a,c,d** Representative data from three experimental replicates. Source data are provided as a Source Data file.

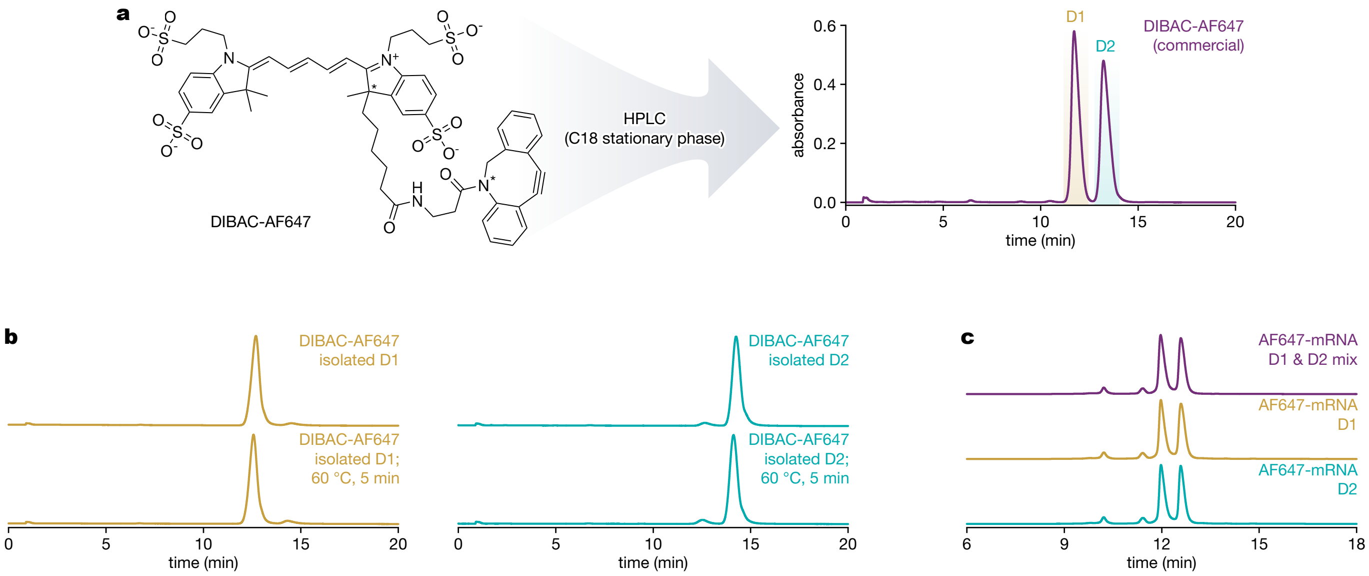

Supplementary Fig. 3 | Influence of DIBAC-AF647 isomerism on the HPLC separation of labelled mRNA. a, Commercial DIBAC-AF647 is a mixture of diastereomers that separate on a C18 HPLC stationary phase upon slow isocratic elution. **b,** We separated the two diastereomers of DIBAC-AF647 and confirmed their stability by heating them for 5 min at 60 °C. **c,** We conjugated 5′-CleaN3 iEMCV-EGFP mRNA with DIBAC-AF647 in the form of mixture of the two diastereomers (D1 & D2 mix), isolated D1, and isolated D2. Next, we analysed the reaction mixtures using HPLC on a PS-DVB stationary phase. The results showed no influence of the DIBAC-AF647 isomerism on the HPLC separation of the labelled mRNA. **a,b,c,** HPLC chromatograms (absorbance at 260 nm; for b and c, traces normalised to maximum). Experimental details are shown in the HPLC section of the Supplementary Methods. Data of one replicate. Source data are provided as a Source Data file.

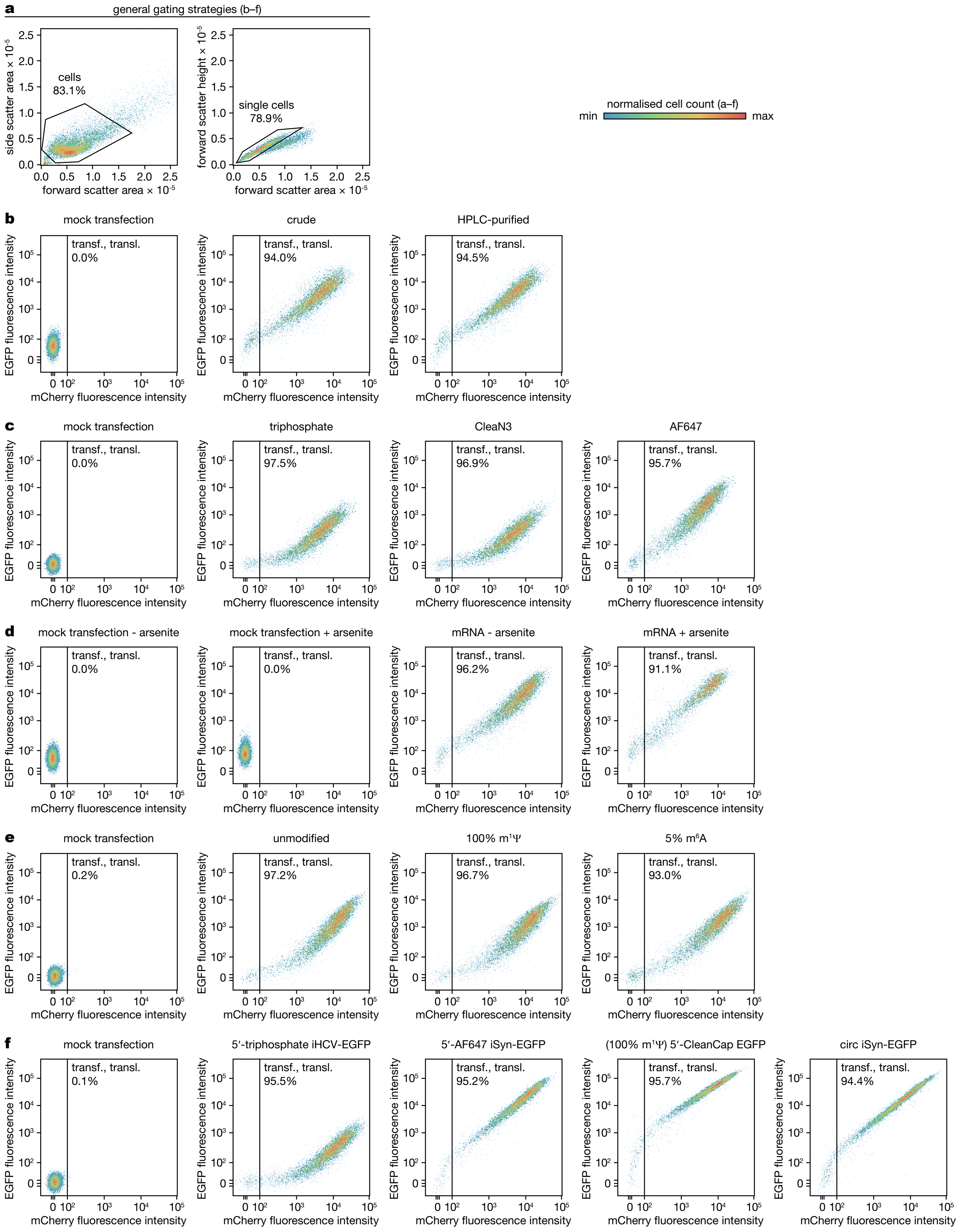

Supplementary Fig. 4 | Representative dot plots and gating strategies used for assessing translational properties of mRNAs. a, General gating strategies applied for analyses involving HEK293 cells. **b,** dot plots and gating strategy for data shown in Fig. 4b. **c,** dot plots and gating strategy for data shown in Fig. 4c. **d,** dot plots and gating strategy for data shown in Fig. 4g. **e,** dot plots and gating strategy for data shown in Fig. 4h. **f,** dot plots and gating strategy for data shown in Fig. 5f. Source data are provided as a Source Data file.

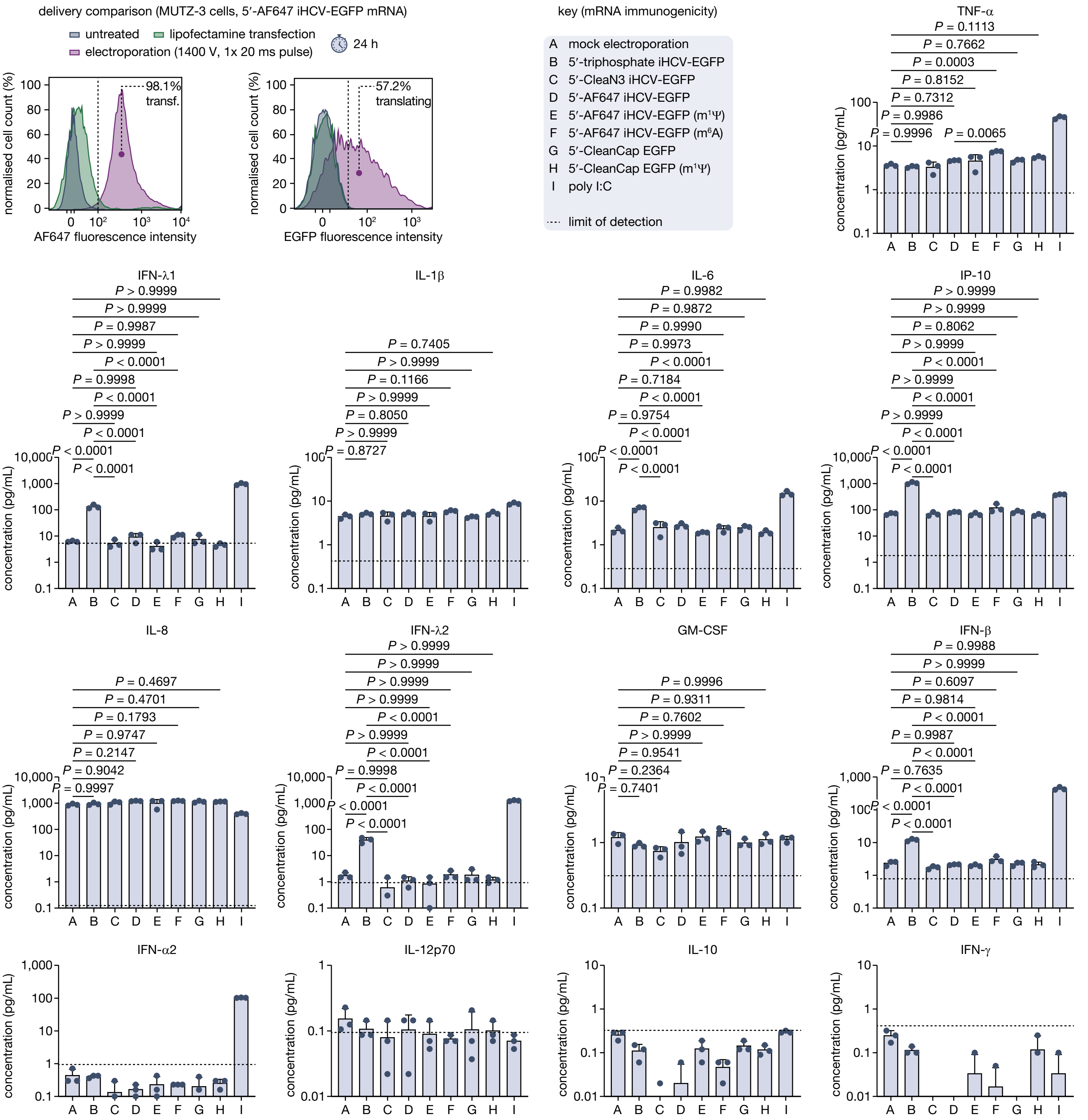

Supplementary Fig. 5 | Immunogenicity of mRNAs in MUTZ-3-derived mature dendritic cells. Top left, influence of mRNA delivery method (lipofectamine transfection and electroporation) on 5′-AF647 iHCV-EGFP mRNA uptake and EGFP expression in MUTZ-3 cells. Flow cytometry data acquired 24 h post-treatment. The histograms are normalised to mode and smoothed. Data of one replicate. Top right and below, concentrations of cytokines secreted to medium by mRNA- or control (1 mM sodium citrate buffer, pH 6.4 – mock or poly I:C)-treated MUTZ-3-derived mature dendritic cells, assessed 24 h post-electroporation. The graphs show individual data points and the means ± s.d. of three experimental replicates. Statistical significance was calculated using one-way ANOVA followed by Tukey's multiple comparisons test. Source data are provided as a Source Data file.

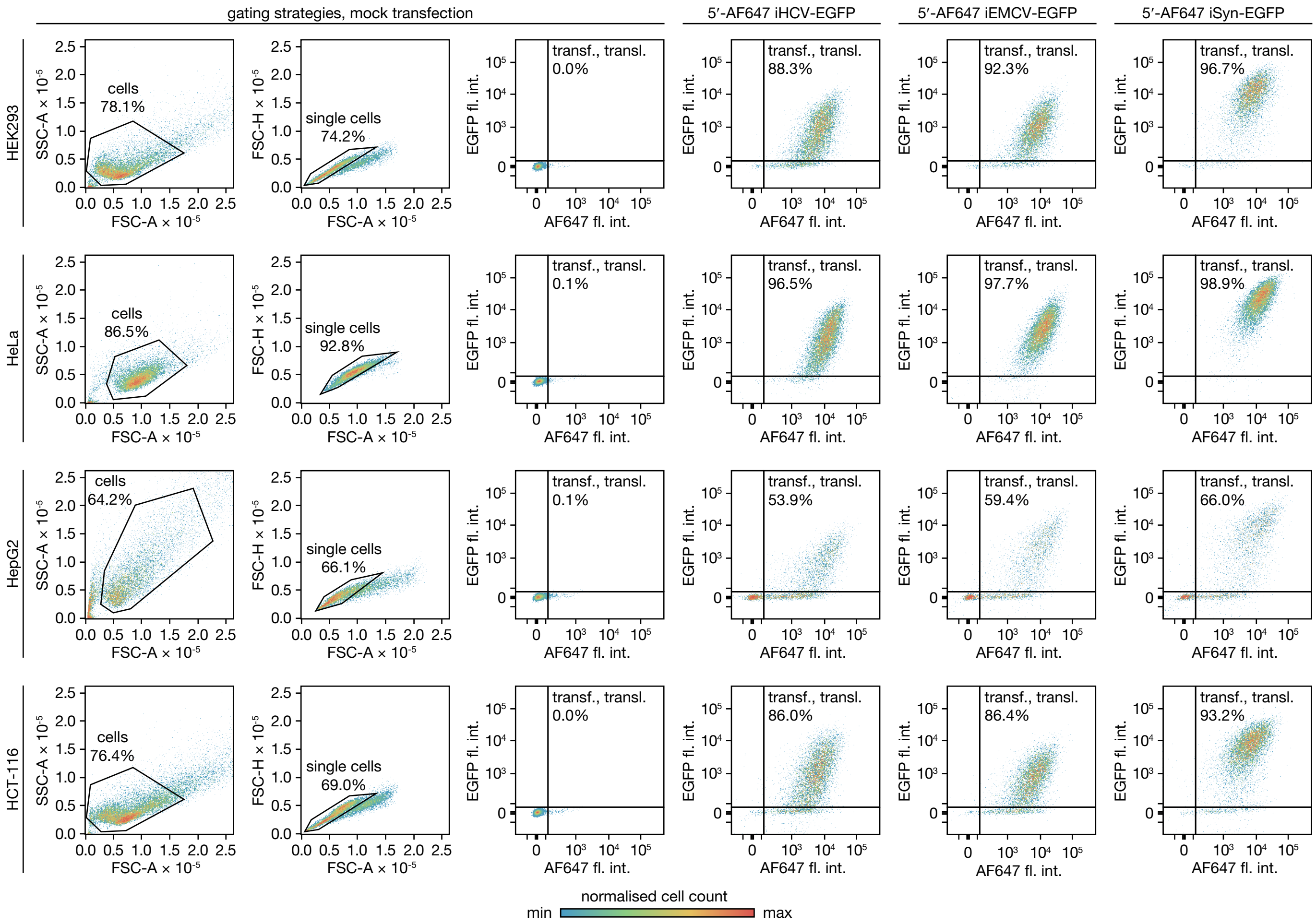

Supplementary Fig. 6 | Representative dot plots and gating strategies used for mRNA productivity studies. Dot plots and gating strategies for data shown in Fig. 5b,c. Source data are provided as a Source Data file.

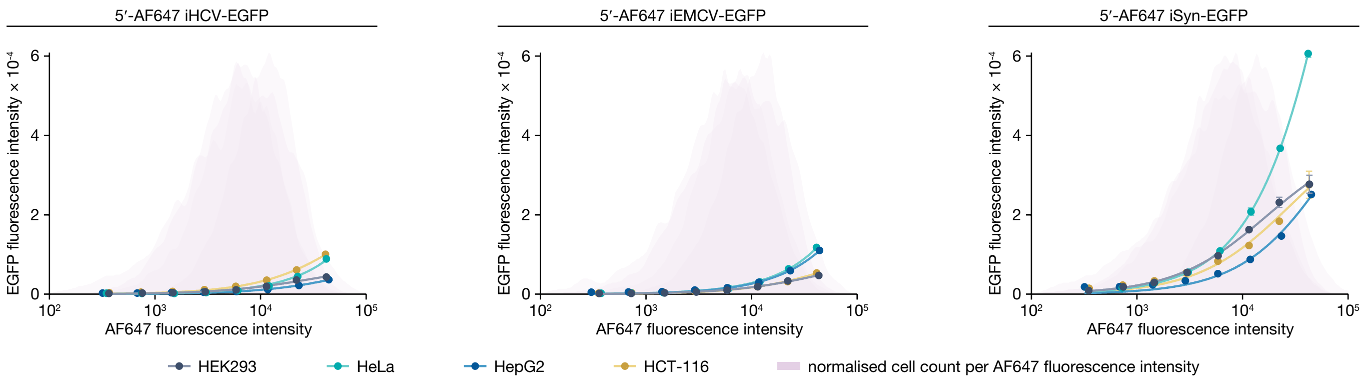

Supplementary Fig. 7 | mRNA dose – EGFP expression relationships. Dose-response profiles representing the relationships between the mRNA (5′-AF647 iHCV-EGFP, 5′-AF647 iEMCV-EGFP, and 5′-AF647 iSyn-EGFP) dose (AF647 fluorescence intensity) and the EGFP expression (EGFP fluorescence intensity) 24 h post-transfection in HEK293, HeLa, HepG2, and HCT-116 cells binned according to increasing AF647 fluorescence intensity as bin means ± s.d. of three experimental replicates. Dose-response curves were obtained by fitting the three-parameter dose-response model to the data using the least squares method. AF647 fluorescence intensity binning strategies are shown in Supplementary Fig. 8a. Representative histograms in purple (normalised to mode and smoothed) show the distribution of the collected cellular AF647 fluorescence intensity data. Source data are provided as a Source Data file.

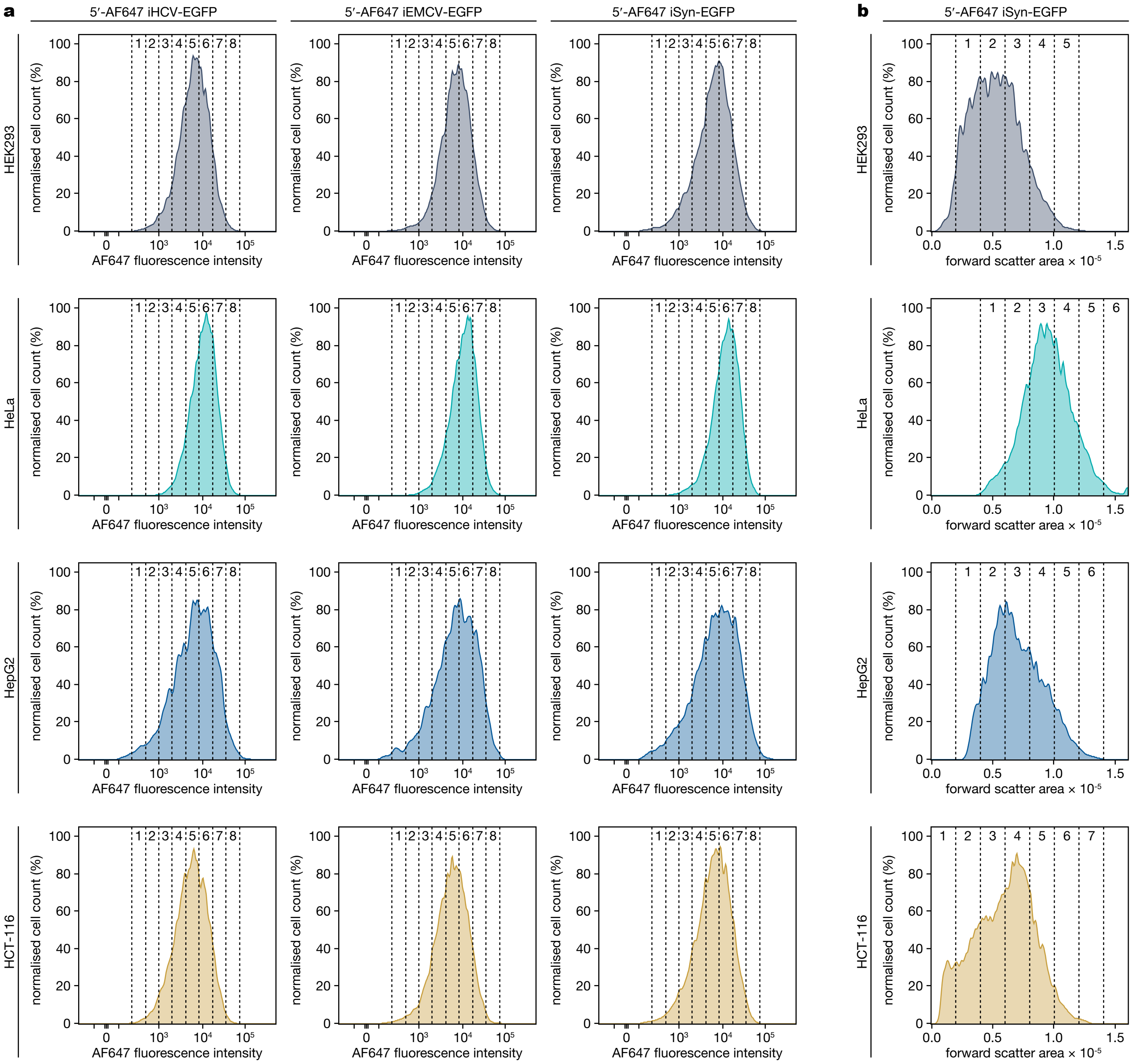

Supplementary Fig. 8 | Binning strategies. a, representative histograms and binning strategies for data shown in Supplementary Fig. 7. **b,** representative histograms and binning strategies for data shown in Fig. 5c. **a,b,** The histograms are normalised to mode and smoothed. Source data are provided as a Source Data file.

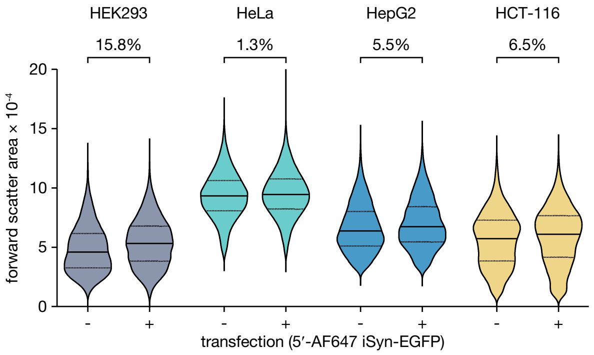

Supplementary Fig. 9 | Cell size comparison for 5′-AF647 iSyn-EGFP mRNA-transfected and non-transfected cells. To answer whether the mRNA transfection influences the cell size, we compared HEK293, HeLa, HepG2, and HCT-116 forward scatter areas (FSC-A) before and after lipofectamine transfection with 5′-AF647 iSyn-EGFP mRNA. The violin plot of FSC-A of mRNA-transfected and non-transfected cells shows data distribution, median (solid line), and first and third quartile (dashed lines). n = 29,476 (HEK293, non-transfected), n = 18,383 (HEK293, transfected), n = 33,601 (HeLa, non-transfected), n = 33,873 (HeLa, transfected), n = 18,930 (HepG2, non-transfected), n = 14,935 (HepG2, transfected), n = 32,728 (HCT-116, non-transfected), and n = 26,277 (HCT-116, transfected) pooled single cells from three experimental replicates. Gating strategies are shown in Supplementary Fig. 6. Source data are provided as a Source Data file.

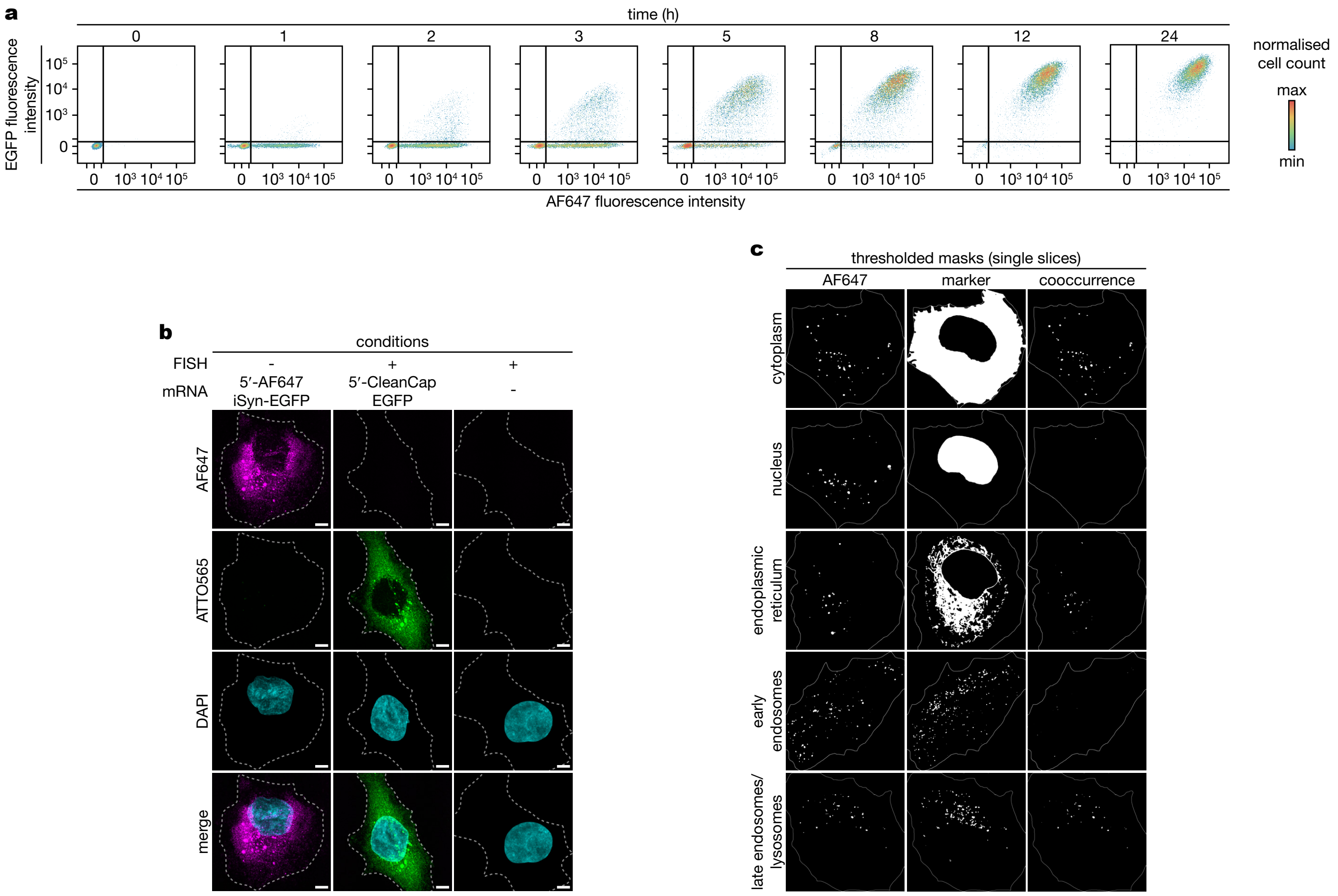

Supplementary Fig. 10 | Assessment of mRNA uptake, cellular localisation and expression. a, Representative dot plots for data shown in Fig. 6b. General gating strategy is shown in Supplementary Fig. 6. **b,** Assessment of the specificity of the FISH probes used in experiment shown in Fig. 6h. Cell segmentation based on the edge of the EGFP fluorescence or cell contours in the DIC channel. Maximum intensity projections from slices spanning the whole cell volumes. Scale bars, 5 µm. **c,** Representative thresholded masks used for calculating Mander's overlap coefficients shown in Fig. 6i. Source data are provided as a Source Data file.

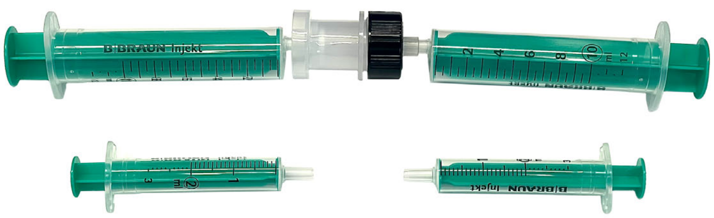

Supplementary Fig. 11 | Experimental setup for the manual synthesis on a solid support.

Nucleic acid sequences

All sequences are listed in the $5'\rightarrow3'$ direction. 'p' denotes a phosphate. 'm^{7}G' denotes N^7-methylguanosine. m$^{2'O}$A denotes $O^{2'}$-methyladenosine. All nucleoside monomers, if not stated otherwise, are linked through phosphodiester bonds.

PCR primer sequences

IRES_GA_F

TGTACGACGATGACGATAAGCTGGAGGCCAAGGTGTGAT

IRES_GA_R

ACCATGGTGGCGAATTCTTTGCCAAGGTTGTGGCCATATTATCATCGTG

EGFP_GA_F

ACACGATGATAATATGGCCACAACCTTGGCAAAGAATTCGCCACC

EGFP_GA_R

CCGGATCAAGCTTCGAATTTTACTTGTACAGCTCGTCCATGC

mCherry_F

GCGGGCCCGGGATCCACCGGTCGCCACCATGGTGAGCAAGGG

mCherry_R

GAAATTGGACAGCAAGAAAGCGAGCTGCGGCCGCTTTACTTGTACAG

pmRNA-EGFP-A120-v1_F

CGATGATAATATGACTTCGAAAGTTTATGATCCAGAACAAATGGTGAGCAAGGGCGAGGAG

pmRNA-EGFP-A120-v1_R

ATTCGATATCAAGCTTATCGAGGCTAGCTCTAGAACGCGTGATATCT

iEMCV_F

GAGCTAGCCTCGATAAGCTTGATATCGAATTCCGCCCCC

iEMCV_R

ATCATAAACTTTCGAAGTCATATTATCATCGTCATATTATCATCGTGTTTTTCAAAGGAAAACCACGTCC
CCGT

pmRNA-EGFP-A120-v2_F

ATGGTGAGCAAGGGCGAG

pmRNA-EGFP-A120-v2_R

GCTAGCTCTAGAACGCGTGATATCT

iHCV_F

TATAAGGAGAGATATCACGCGTTCTAGAGCTAGCTAGTGTACGTAGCCAGCCC

iHCV_R

GGTGAACAGCTCCTCGCCCTTGCTCACCATGGTTTTTCTTTGAGGTTTAGGATTCGTG

oEGFP_F

GCATATATATAACATATACTGTGATCATGGTGAGCAAGGGCGAGGAG

oEGFP_R

CACCGAGGCTCCAGCCAATTGTTATTACTTGTACAGCTCGTCCATGCC

IVT_φ6.5_iEMCV-EGFP-A120_F

ACTTAATACGACTCACTATAGGAGAGATATCACGCGTTCTAG

IVT_φ2.5_iEMCV-EGFP-A120_F

ACTTAATACGACTCACTATTAGGAGAGATATCACGCGTTCTAG

IVT_iEMCV-EGFP-A120_R

GTGTTGTTCCAGTTTGGAACAAGAGTCC

IVT_iEMCV-Cas9-A120_F

GACTTAATACGACTCACTATAAGGAGAGATATCACGCGTCACAAATACCACTGAGATCCG

IVT_iEMCV-Cas9-A120_R

TTTTTTTTTTTTTTTTTTTTTTTTTTTTTTTTTTTTTTTTTTTTTTTTTTTTTTTTTTTTTTTTTTTTTT
TTTTTTTTTTTTTTTTTTTTTTTTTTTTTTTTTTTTTTTTTTTTTTTTTTCCCATATGTCCTTCCGAGTG

IVT_iSyn-EGFP-A120_F

GACTTAATACGACTCACTATAAGGCGTCATATTCGGCG

IVT_iSyn-EGFP-A120_R

TTTTTTTTTTTTTTTTTTTTTTTTTTTTTTTTTTTTTTTTTTTTTTTTTTTTTTTTTTTTTTTTTTTTTT
TTTTTTTTTTTTTTTTTTTTTTTTTTTTTTTTTTTTTTTTTTTTTTTTTTCATCTACTGAGTCGGC

IVT_circ-iSyn-EGFP_F

AAAAAAAAAAAAAAAAAAAAAAAAAAAGGCCAGTGAATTGTAATACGACTCACTATAGGG

IVT_circ-iSyn-EGFP_R

TTTTTTTTTTTTTTTTTTTTTTTTTTTTTTCAAAAAACCCCTCAAGACCCGTTTAGAGGC

EGFP_F

AGATCCGCCACAACATCGAG

EGFP_R

AACTCCAGCAGGACCATGTG

GAPDH_F

TCAAGGCTGAGAACGGGAAG

GAPDH_R

CGCCCCACTTGATTTTGGAG

DNAzyme sequences

Dz_10-23_1

GCTAGCTCTAGAAGGCTAGCTACAACGAGCGTGATATCTCTCC

Dz_10-23_2

CAGTGGTATTTGTGAGGCTAGCTACAACGAGCGTGATATCTCTCC

Dz_10-23_3

GATCCGAGCTCGGTAGGCTAGCTACAACGACAAGCTTGGGTCTCC

FISH probe sequences

X = ATTO 565

FISH_EGFP_1

XTGAACTTGTGGCCGTTTACG

FISH_EGFP_2

XTGGCGGACTTGAAGAAGTCG

FISH_EGFP_3

XTTGAAGTCGATGCCCTTCAG

FISH_EGFP_4

XTGCTCAGGTAGTGGTTGTCG

Other oligonucleotide sequences

A_ins_φ6.5-1

pCACCTGCAACATGACTTAATACGACTCACTATAAGGAGAGATATCA

A_ins_φ6.5-2

pCGCGTGATATCTCTCCTTATAGTGAGTCGTATTAAGTCATGTTGCAGGTG

A120-AarI-1

pAAACAAAAAAAAAAAAAAAAAAAAAAAAAAAAAAAAAAAAAAAAAAAAAAAAAAAAAAAAAAAAAAAAA
AAAAAAAAAAAAAAAAAAAAAAAAAAAAAAAAAAAAAAAAAAAAAAAAAAAAAAAAAAAGCAGGTGGGAG
GGTACGTTCGTGATAAACAAACGGCC

A120-AarI-2

pTTTGTTTATCACGAACGTACCCTCCCACCTGCTTTTTTTTTTTTTTTTTTTTTTTTTTTTTTTTTTTTT
TTTTTTTTTTTTTTTTTTTTTTTTTTTTTTTTTTTTTTTTTTTTTTTTTTTTTTTTTTTTTTTTTTTTTT
TTTTTTTTTTTTTTTTTGTTT

A120-BsaI-1

pAAACAAAAAAAAAAAAAAAAAAAAAAAAAAAAAAAAAAAAAAAAAAAAAAAAAAAAAAAAAAAAAAAAA
AAAAAAAAAAAAAAAAAAAAAAAAAAAAAAAAAAAAAAAAAAAAAAAAAAAAAAAAGAGACCGGTACGTT
CGTGATAAACAAACGGCC

A120-BsaI-2

pTTTGTTTATCACGAACGTACCGGTCTCTTTTTTTTTTTTTTTTTTTTTTTTTTTTTTTTTTTTTTTTTT
TTTTTTTTTTTTTTTTTTTTTTTTTTTTTTTTTTTTTTTTTTTTTTTTTTTTTTTTTTTTTTTTTTTTTT
TTTTTTTTTGTTT

Plasmid sequences

pRSET-IRES-EGFP

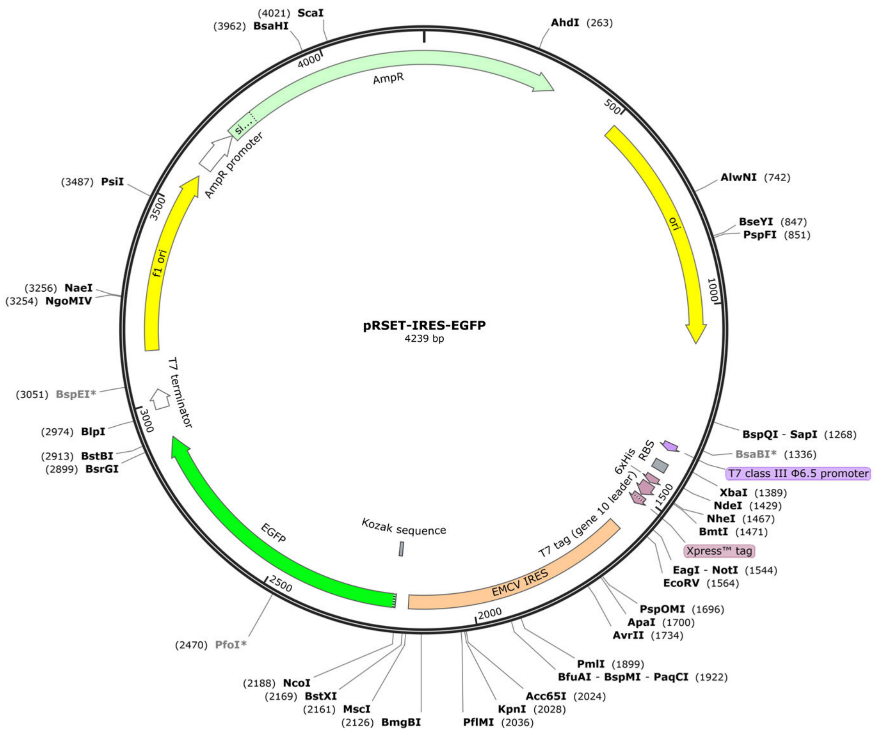

GGATCTTCTTGAGATCCTTTTTTTCTGCGCGTAATCTGCTGCTTGCAAACAAAAAAACCACCGCTACCAG
CGGTGGTTTGTTTGCCGGATCAAGAGCTACCAACTCTTTTTCCGAAGGTAACTGGCTTCAGCAGAGCGCA
GATACCAAATACTGTTCTTCTAGTGTAGCCGTAGTTAGGCCACCACTTCAAGAACTCTGTAGCACCGCCT
ACATACCTCGCTCTGCTAATCCTGTTACCAGTGGCTGCTGCCAGTGGCGATAAGTCGTGTCTTACCGGGT
TGGACTCAAGACGATAGTTACCGGATAAGGCGCAGCGGTCGGGCTGAACGGGGGGTTCGTGCACACAGCC
CAGCTTGGAGCGAACGACCTACACCGAACTGAGATACCTACAGCGTGAGCTATGAGAAAGCGCCACGCTT
CCCGAAGGGAGAAAGGCGGACAGGTATCCGGTAAGCGGCAGGGTCGGAACAGGAGAGCGCACGAGGGAGC
TTCCAGGGGGAAACGCCTGGTATCTTTATAGTCCTGTCGGGTTTCGCCACCTCTGACTTGAGCGTCGATT
TTTGTGATGCTCGTCAGGGGGGCGGAGCCTATGGAAAAACGCCAGCAACGCGGCCTTTTTACGGTTCCTG
GCCTTTTGCTGGCCTTTTGCTCACATGTTCTTTCCTGCGTTATCCCCTGATTCTGTGGATAACCGTATTA
CCGCCTTTGAGTGAGCTGATACCGCTCGCCGCAGCCGAACGACCGAGCGCAGCGAGTCAGTGAGCGAGGA
AGCGGAAGAGCGCCCAATACGCAAACCGCCTCTCCCCGCGCGTTGGCCGATTCATTAATGCAGGATCTCG
ATCCCGCGAAATTAATACGACTCACTATAGGGAGACCACAACGGTTTCCCTCTAGAAATAATTTTGTTTA
ACTTTAAGAAGGAGATATACATATGCGGGGTTCTCATCATCATCATCATCATGGTATGGCTAGCATGACT
GGTGGACAGCAAATGGGTCGGGATCTGTACGACGATGACGATAAGCTGGAGGCCAAGGTGTGATGCGGCC
GCTCGATAAGCTTGATATCGAATTCCGCCCCCCCCCCCTCTCCCTCCCCCCCCCCTAACGTTACTGGCCG
AAGCCGCTTGGAATAAGGCCGGTGTGCGTTTGTCTATATGTTATTTTCCACCATATTGCCGTCTTTTGGC
AATGTGAGGGCCCGGAAACCTGGCCCTGTCTTCTTGACGAGCATTCCTAGGGGTCTTTCCCCTCTCGCCA
AAGGAATGCAAGGTCTGTTGAATGTCGTGAAGGAAGCAGTTCCTCTGGAAGCTTCTTGAAGACAAACAAC
GTCTGTAGCGACCCTTTGCAGGCAGCGGAACCCCCCACCTGGCGACAGGTGCCTCTGCGGCCAAAAGCCA
CGTGTATAAGATACACCTGCAAAGGCGGCACAACCCCAGTGCCACGTTGTGAGTTGGATAGTTGTGGAAA
GAGTCAAATGGCTCTCCTCAAGCGTATTCAACAAGGGGCTGAAGGATGCCCAGAAGGTACCCCATTGTAT
GGGATCTGATCTGGGGCCTCGGTGCACATGCTTTACATGTGTTTAGTCGAGGTTAAAAAAACGTCTAGGC
CCCCCGAACCACGGGGACGTGGTTTTCCTTTGAAAAACACGATGATAATATGGCCACAACCTTGGCAAAG
AATTCGCCACCATGGTGAGCAAGGGCGAGGAGCTGTTCACCGGGGTGGTGCCCATCCTGGTCGAGCTGGA
CGGCGACGTAAACGGCCACAAGTTCAGCGTGTCCGGCGAGGGCGAGGGCGATGCCACCTACGGCAAGCTG

ACCCTGAAGTTCATCTGCACCACCGGCAAGCTGCCCGTGCCCTGGCCCACCCTCGTGACCACCCTGACCT
ACGGCGTGCAGTGCTTCAGCCGCTACCCCGACCACATGAAGCAGCACGACTTCTTCAAGTCCGCCATGCC
CGAAGGCTACGTCCAGGAGCGCACCATCTTCTTCAAGGACGACGGCAACTACAAGACCCGCGCCGAGGTG
AAGTTCGAGGGCGACACCCTGGTGAACCGCATCGAGCTGAAGGGCATCGACTTCAAGGAGGACGGCAACA
TCCTGGGGCACAAGCTGGAGTACAACTACAACAGCCACAACGTCTATATCATGGCCGACAAGCAGAAGAA
CGGCATCAAGGTGAACTTCAAGATCCGCCACAACATCGAGGACGGCAGCGTGCAGCTCGCCGACCACTAC
CAGCAGAACACCCCCATCGGCGACGGCCCCGTGCTGCTGCCCGACAACCACTACCTGAGCACCCAGTCCG
CCCTGAGCAAAGACCCCAACGAGAAGCGCGATCACATGGTCCTGCTGGAGTTCGTGACCGCCGCCGGGAT
CACTCTCGGCATGGACGAGCTGTACAAGTAAAATTCGAAGCTTGATCCGGCTGCTAACAAAGCCCGAAAG
GAAGCTGAGTTGGCTGCTGCCACCGCTGAGCAATAACTAGCATAACCCCTTGGGGCCTCTAAACGGGTCT
TGAGGGGTTTTTTGCTGAAAGGAGGAACTATATCCGGATCTGGCGTAATAGCGAAGAGGCCCGCACCGAT
CGCCCTTCCCAACAGTTGCGCAGCCTGAATGGCGAATGGGACGCGCCCTGTAGCGGCGCATTAAGCGCGG
CGGGTGTGGTGGTTACGCGCAGCGTGACCGCTACACTTGCCAGCGCCCTAGCGCCCGCTCCTTTCGCTTT
CTTCCCTTCCTTTCTCGCCACGTTCGCCGGCTTTCCCCGTCAAGCTCTAAATCGGGGGCTCCCTTTAGGG
TTCCGATTTAGTGCTTTACGGCACCTCGACCCCAAAAAACTTGATTAGGGTGATGGTTCACGTAGTGGGC
CATCGCCCTGATAGACGGTTTTTCGCCCTTTGACGTTGGAGTCCACGTTCTTTAATAGTGGACTCTTGTT
CCAAACTGGAACAACACTCAACCCTATCTCGGTCTATTCTTTTGATTTATAAGGGATTTTGCCGATTTCG
GCCTATTGGTTAAAAAATGAGCTGATTTAACAAAAATTTAACGCGAATTTTAACAAAATATTAACGCTTA
CAATTTAGGTGGCACTTTTCGGGGAAATGTGCGCGGAACCCCTATTTGTTTATTTTTCTAAATACATTCA
AATATGTATCCGCTCATGAGACAATAACCCTGATAAATGCTTCAATAATATTGAAAAAGGAAGAGTATGA
GTATTCAACATTTCCGTGTCGCCCTTATTCCCTTTTTTGCGGCATTTTGCCTTCCTGTTTTTGCTCACCC
AGAAACGCTGGTGAAAGTAAAAGATGCTGAAGATCAGTTGGGTGCACGAGTGGGTTACATCGAACTGGAT
CTCAACAGCGGTAAGATCCTTGAGAGTTTTCGCCCCGAAGAACGTTTTCCAATGATGAGCACTTTTAAAG
TTCTGCTATGTGGCGCGGTATTATCCCGTATTGACGCCGGGCAAGAGCAACTCGGTCGCCGCATACACTA
TTCTCAGAATGACTTGGTTGAGTACTCACCAGTCACAGAAAAGCATCTTACGGATGGCATGACAGTAAGA
GAATTATGCAGTGCTGCCATAACCATGAGTGATAACACTGCGGCCAACTTACTTCTGACAACGATCGGAG
GACCGAAGGAGCTAACCGCTTTTTTGCACAACATGGGGGATCATGTAACTCGCCTTGATCGTTGGGAACC
GGAGCTGAATGAAGCCATACCAAACGACGAGCGTGACACCACGATGCCTGTAGCAATGGCAACAACGTTG
CGCAAACTATTAACTGGCGAACTACTTACTCTAGCTTCCCGGCAACAATTAATAGACTGGATGGAGGCGG
ATAAAGTTGCAGGACCACTTCTGCGCTCGGCCCTTCCGGCTGGCTGGTTTATTGCTGATAAATCTGGAGC
CGGTGAGCGTGGGTCTCGCGGTATCATTGCAGCACTGGGGCCAGATGGTAAGCCCTCCCGTATCGTAGTT
ATCTACACGACGGGGAGTCAGGCAACTATGGATGAACGAAATAGACAGATCGCTGAGATAGGTGCCTCAC
TGATTAAGCATTGGTAACTGTCAGACCAAGTTTACTCATATATACTTTAGATTGATTTAAAACTTCATTT
TTAATTTAAAAGGATCTAGGTGAAGATCCTTTTTGATAATCTCATGACCAAAATCCCTTAACGTGAGTTT
TCGTTCCACTGAGCGTCAGACCCCGTAGAAAAGATCAAA

pmRNA-EGFP-A120

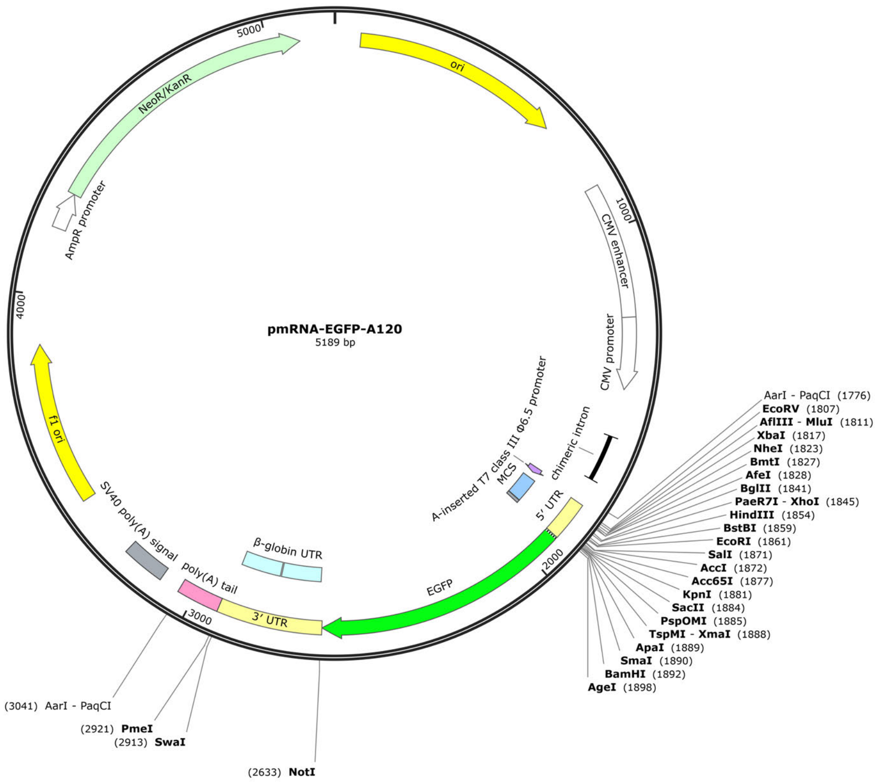

TGACCAAAATCCCTTAACGTGAGTTTTCGTTCCACTGAGCGTCAGACCCCGTAGAAAAGATCAAAGGATC
TTCTTGAGATCCTTTTTTTCTGCGCGTAATCTGCTGCTTGCAAACAAAAAAACCACCGCTACCAGCGGTG
GTTTGTTTGCCGGATCAAGAGCTACCAACTCTTTTTCCGAAGGTAACTGGCTTCAGCAGAGCGCAGATAC
CAAATACTGTTCTTCTAGTGTAGCCGTAGTTAGGCCACCACTTCAAGAACTCTGTAGCACCGCCTACATA
CCTCGCTCTGCTAATCCTGTTACCAGTGGCTGCTGCCAGTGGCGATAAGTCGTGTCTTACCGGGTTGGAC
TCAAGACGATAGTTACCGGATAAGGCGCAGCGGTCGGGCTGAACGGGGGGTTCGTGCACACAGCCCAGCT
TGGAGCGAACGACCTACACCGAACTGAGATACCTACAGCGTGAGCTATGAGAAAGCGCCACGCTTCCCGA
AGGGAGAAAGGCGGACAGGTATCCGGTAAGCGGCAGGGTCGGAACAGGAGAGCGCACGAGGGAGCTTCCA
GGGGGAAACGCCTGGTATCTTTATAGTCCTGTCGGGTTTCGCCACCTCTGACTTGAGCGTCGATTTTTGT
GATGCTCGTCAGGGGGGCGGAGCCTATGGAAAAACGCCAGCAACGCGGCCTTTTTACGGTTCCTGGCCTT
TTGCTGGCCTTTTGCTCACATGGCTCGACAGATCATCAATATTGGCCATTAGCCATATTATTCATTGGTT
ATATAGCATAAATCAATATTGGCTATTGGCCATTGCATACGTTGTATCTATATCATAATATGTACATTTA
TATTGGCTCATGTCCAATATGACCGCCATGTTGGCATTGATTATTGACTAGTTATTAATAGTAATCAATT
ACGGGGTCATTAGTTCATAGCCCATATATGGAGTTCCGCGTTACATAACTTACGGTAAATGGCCCGCCTG
GCTGACCGCCCAACGACCCCCGCCCATTGACGTCAATAATGACGTATGTTCCCATAGTAACGCCAATAGG
GACTTTCCATTGACGTCAATGGGTGGAGTATTTACGGTAAACTGCCCACTTGGCAGTACATCAAGTGTAT
CATATGCCAAGTCCGCCCCCTATTGACGTCAATGACGGTAAATGGCCCGCCTGGCATTATGCCCAGTACA
TGACCTTACGGGACTTTCCTACTTGGCAGTACATCTACGTATTAGTCATCGCTATTACCATGGTGATGCG
GTTTTGGCAGTACACCAATGGGCGTGGATAGCGGTTTGACTCACGGGGATTTCCAAGTCTCCACCCCATT
GACGTCAATGGGAGTTTGTTTTGGCACCAAAATCAACGGGACTTTCCAAAATGTCGTAATAACCCCGCCC
CGTTGACGCAAATGGGCGGTAGGCGTGTACGGTGGGAGGTCTATATAAGCAGAGCTCGTTTAGTGAACCG
TCAGATCACTAGACGCTTTATTGCGGTAGTTTATCACAGTTAAATTGCTAACGCAGTCAGTGCTTCTGAC
ACAACAGTCTCGAACTTAAGCTGCAGAAGTTGGTCGTGAGGCACTGGGCAGGTAAGTATCAAGGTTACAA
GACAGGTTTAAGGAGACCAATAGAAACTGGGCTTGTCGAGACAGAGAAGACTCTTGCGTTTCTGATAGGC
ACCTATTGGTCTTACTGACATCCACTTTGCCTTTCTCTCCACAGGTGTCCACTCCCAGTTCAATTACAGC
TCTTAAGGCTAGAGTCACCTGCAACATGACTTAATACGACTCACTATAAGGAGAGATATCACGCGTTCTA

GAGCTAGCGCTACCGGACTCAGATCTCGAGCTCAAGCTTCGAATTCTGCAGTCGACGGTACCGCGGGCCC
GGGATCCACCGGTCGCCACCATGGTGAGCAAGGGCGAGGAGCTGTTCACCGGGGTGGTGCCCATCCTGGT
CGAGCTGGACGGCGACGTAAACGGCCACAAGTTCAGCGTGTCCGGCGAGGGCGAGGGCGATGCCACCTAC
GGCAAGCTGACCCTGAAGTTCATCTGCACCACCGGCAAGCTGCCCGTGCCCTGGCCCACCCTCGTGACCA
CCCTGACCTACGGCGTGCAGTGCTTCAGCCGCTACCCCGACCACATGAAGCAGCACGACTTCTTCAAGTC
CGCCATGCCCGAAGGCTACGTCCAGGAGCGCACCATCTTCTTCAAGGACGACGGCAACTACAAGACCCGC
GCCGAGGTGAAGTTCGAGGGCGACACCCTGGTGAACCGCATCGAGCTGAAGGGCATCGACTTCAAGGAGG
ACGGCAACATCCTGGGGCACAAGCTGGAGTACAACTACAACAGCCACAACGTCTATATCATGGCCGACAA
GCAGAAGAACGGCATCAAGGTGAACTTCAAGATCCGCCACAACATCGAGGACGGCAGCGTGCAGCTCGCC
GACCACTACCAGCAGAACACCCCCATCGGCGACGGCCCCGTGCTGCTGCCCGACAACCACTACCTGAGCA
CCCAGTCCGCCCTGAGCAAAGACCCCAACGAGAAGCGCGATCACATGGTCCTGCTGGAGTTCGTGACCGC
CGCCGGGATCACTCTCGGCATGGACGAGCTGTACAAGTAAAGCGGCCGCAGCTCGCTTTCTTGCTGTCCA
ATTTCTATTAAAGGTTCCTTTGTTCCCTAAGTCCAACTACTAAACTGGGGGATATTATGAAGGGCCTTGA
GCATTTGGATTCTGCCTAATAAAAAACATTTATTTTCATTGCGTTTAGCTCGCTTTCTTGCTGTCCAATT
TCTATTAAAGGTTCCTTTGTTCCCTAAGTCCAACTACTAAACTGGGGGATATTATGAAGGGCCTTGAGCA
TTTGGATTCTGCCTAATAAAAAACATTTATTTTCATTGCATTTAAATGTTTAAACAAAAAAAAAAAAAAA
AAAAAAAAAAAAAAAAAAAAAAAAAAAAAAAAAAAAAAAAAAAAAAAAAAAAAAAAAAAAAAAAAAAAAA
AAAAAAAAAAAAAAAAAAAAAAAAAAAAAAAAAAAAAAAAAGCAGGTGGGAGGGTACGTTCGTGATAAACAA
ACGGCCGCTTCGAGCAGACATGATAAGATACATTGATGAGTTTGGACAAACCACAACTAGAATGCAGTGA
AAAAAATGCTTTATTTGTGAAATTTGTGATGCTATTGCTTTATTTGTAACCATTATAAGCTGCAATAAAC
AAGTTAACAACAACAATTGCATTCATTTTATGTTTCAGGTTCAGGGGGAGATGTGGGAGGTTTTTTAAAG
CAAGTAAAACCTCTACAAATGTGGTAAAATCGATAAGGATCAGGGCTGGCGTAATAGCGAAGAGGCCCGC
ACCGATCGCCCTTCCCAACAGTTGCGCAGCCTGAATGGCGAATGGACGCGCCCTGTAGCGGCGCATTAAG
CGCGGCGGGTGTGGTGGTTACGCGCAGCGTGACCGCTACACTTGCCAGCGCCCTAGCGCCCGCTCCTTTC
GCTTTCTTCCCTTCCTTTCTCGCCACGTTCGCCGGCTTTCCCCGTCAAGCTCTAAATCGGGGGCTCCCTT
TAGGGTTCCGATTTAGTGCTTTACGGCACCTCGACCCCAAAAAACTTGATTAGGGTGATGGTTCACGTAG
TGGGCCATCGCCCTGATAGACGGTTTTTCGCCCTTTGACGTTGGAGTCCACGTTCTTTAATAGTGGACTC
TTGTTCCAAACTGGAACAACACTCAACCCTATCTCGGTCTATTCTTTTGATTTATAAGGGATTTTGCCGA
TTTCGGCCTATTGGTTAAAAAATGAGCTGATTTAACAAAAATTTAACGCGAATTTTAACAAAATATTAAC
GCTTACAATTTCCTGATGCGGTATTTTCTCCTTACGCATCTGTGCGGTATTTCACACCGCATATGGTGCA
CTCTCAGTACAATCTGCTCTGATGCCGCATAGTTAAGCCAGCCCCGACACCCGCCAACACCCGCTGACGC
GCCCTGACGGGCTTGTCTGCTCCCGGCATCCGCTTACAGACAAGCTGTGACCGTCTCCGGGAGCTGCATG
TGTCAGAGGTTTTCACCGTCATCACCGAAACGCGCGAGACGAAAGGGCCTCGTGATACGCCTATTTTTAT
AGGTTAATGTCATGATAATAATGGTTTCTTAGACGTCAGGTGGCACTTTTCGGGGAAATGTGCGCGGAAC
CCCTATTTGTTTATTTTTCTAAATACATTCAAATATGTATCCGCTCATGAGACAATAACCCTGATAAATG
CTTCAATAATATTGAAAAAGGAAGAGTATGATTGAACAAGATGGATTGCACGCAGGTTCTCCGGCCGCTT
GGGTGGAGAGGCTATTCGGCTATGACTGGGCACAACAGACAATCGGCTGCTCTGATGCCGCCGTGTTCCG
GCTGTCAGCGCAGGGGCGCCCGGTTCTTTTTGTCAAGACCGACCTGTCCGGTGCCCTGAATGAACTGCAA
GACGAGGCAGCGCGGCTATCGTGGCTGGCCACGACGGGCGTTCCTTGCGCAGCTGTGCTCGACGTTGTCA
CTGAAGCGGGAAGGGACTGGCTGCTATTGGGCGAAGTGCCGGGGCAGGATCTCCTGTCATCTCACCTTGC
TCCTGCCGAGAAAGTATCCATCATGGCTGATGCAATGCGGCGGCTGCATACGCTTGATCCGGCTACCTGC
CCATTCGACCACCAAGCGAAACATCGCATCGAGCGAGCACGTACTCGGATGGAAGCCGGTCTTGTCGATC
AGGATGATCTGGACGAAGAGCATCAGGGGCTCGCGCCAGCCGAACTGTTCGCCAGGCTCAAGGCGAGCAT
GCCCGACGGCGAGGATCTCGTCGTGACCCATGGCGATGCCTGCTTGCCGAATATCATGGTGGAAAATGGC
CGCTTTTCTGGATTCATCGACTGTGGCCGGCTGGGTGTGGCGGACCGCTATCAGGACATAGCGTTGGCTA
CCCGTGATATTGCTGAAGAGCTTGGCGGCGAATGGGCTGACCGCTTCCTCGTGCTTTACGGTATCGCCGC
TCCCGATTCGCAGCGCATCGCCTTCTATCGCCTTCTTGACGAGTTCTTCTGACTGTCAGACCAAGTTTAC

TCATATATACTTTAGATTGATTTAAAACTTCATTTTTAATTTAAAAGGATCTAGGTGAAGATCCTTTTTG
ATAATCTCA

pmRNA-mCherry-A120

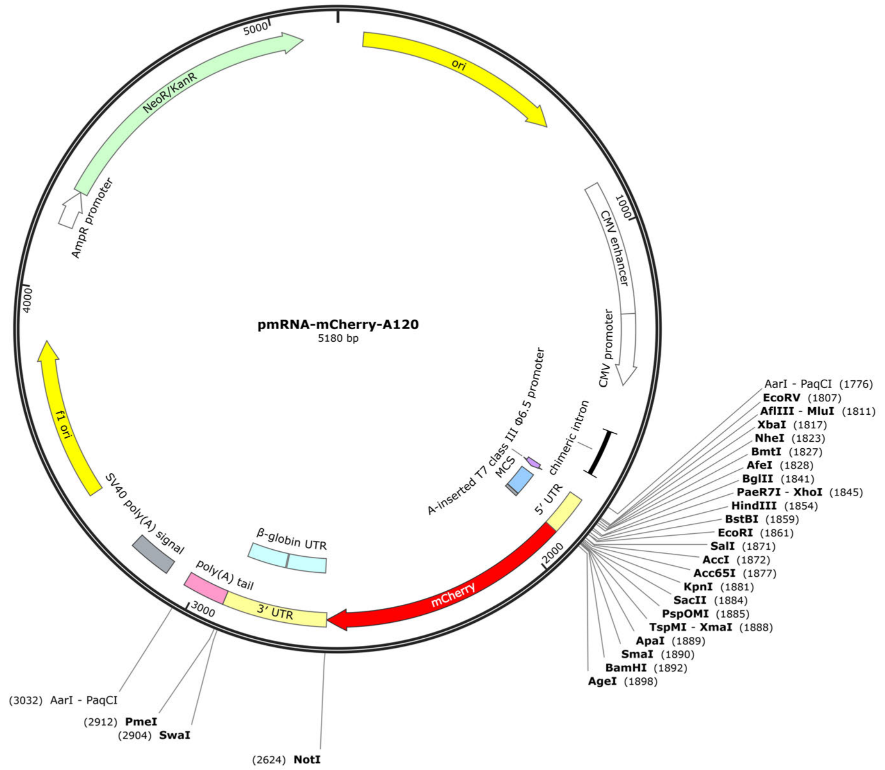

TGACCAAAATCCCTTAACGTGAGTTTTCGTTCCACTGAGCGTCAGACCCCGTAGAAAAGATCAAAGGATC
TTCTTGAGATCCTTTTTTTCTGCGCGTAATCTGCTGCTTGCAAACAAAAAAACCACCGCTACCAGCGGTG
GTTTGTTTGCCGGATCAAGAGCTACCAACTCTTTTTCCGAAGGTAACTGGCTTCAGCAGAGCGCAGATAC
CAAATACTGTTCTTCTAGTGTAGCCGTAGTTAGGCCACCACTTCAAGAACTCTGTAGCACCGCCTACATA
CCTCGCTCTGCTAATCCTGTTACCAGTGGCTGCTGCCAGTGGCGATAAGTCGTGTCTTACCGGGTTGGAC
TCAAGACGATAGTTACCGGATAAGGCGCAGCGGTCGGGCTGAACGGGGGGTTCGTGCACACAGCCCAGCT
TGGAGCGAACGACCTACACCGAACTGAGATACCTACAGCGTGAGCTATGAGAAAGCGCCACGCTTCCCGA
AGGGAGAAAGGCGGACAGGTATCCGGTAAGCGGCAGGGTCGGAACAGGAGAGCGCACGAGGGAGCTTCCA
GGGGGAAACGCCTGGTATCTTTATAGTCCTGTCGGGTTTCGCCACCTCTGACTTGAGCGTCGATTTTTGT
GATGCTCGTCAGGGGGGCGGAGCCTATGGAAAAACGCCAGCAACGCGGCCTTTTTACGGTTCCTGGCCTT
TTGCTGGCCTTTTGCTCACATGGCTCGACAGATCATCAATATTGGCCATTAGCCATATTATTCATTGGTT
ATATAGCATAAATCAATATTGGCTATTGGCCATTGCATACGTTGTATCTATATCATAATATGTACATTTA
TATTGGCTCATGTCCAATATGACCGCCATGTTGGCATTGATTATTGACTAGTTATTAATAGTAATCAATT
ACGGGGTCATTAGTTCATAGCCCATATATGGAGTTCCGCGTTACATAACTTACGGTAAATGGCCCGCCTG
GCTGACCGCCCAACGACCCCCGCCCATTGACGTCAATAATGACGTATGTTCCCATAGTAACGCCAATAGG
GACTTTCCATTGACGTCAATGGGTGGAGTATTTACGGTAAACTGCCCACTTGGCAGTACATCAAGTGTAT
CATATGCCAAGTCCGCCCCCTATTGACGTCAATGACGGTAAATGGCCCGCCTGGCATTATGCCCAGTACA
TGACCTTACGGGACTTTCCTACTTGGCAGTACATCTACGTATTAGTCATCGCTATTACCATGGTGATGCG
GTTTTGGCAGTACACCAATGGGCGTGGATAGCGGTTTGACTCACGGGGATTTCCAAGTCTCCACCCCATT
GACGTCAATGGGAGTTTGTTTTGGCACCAAAATCAACGGGACTTTCCAAAATGTCGTAATAACCCCGCCC
CGTTGACGCAAATGGGCGGTAGGCGTGTACGGTGGGAGGTCTATATAAGCAGAGCTCGTTTAGTGAACCG
TCAGATCACTAGACGCTTTATTGCGGTAGTTTATCACAGTTAAATTGCTAACGCAGTCAGTGCTTCTGAC
ACAACAGTCTCGAACTTAAGCTGCAGAAGTTGGTCGTGAGGCACTGGGCAGGTAAGTATCAAGGTTACAA
GACAGGTTTAAGGAGACCAATAGAAACTGGGCTTGTCGAGACAGAGAAGACTCTTGCGTTTCTGATAGGC

ACCTATTGGTCTTACTGACATCCACTTTGCCTTTCTCTCCACAGGTGTCCACTCCCAGTTCAATTACAGC
TCTTAAGGCTAGAGTCACCTGCAACATGACTTAATACGACTCACTATAAGGAGAGATATCACGCGTTCTA
GAGCTAGCGCTACCGGACTCAGATCTCGAGCTCAAGCTTCGAATTCTGCAGTCGACGGTACCGCGGGCCC
GGGATCCACCGGTCGCCACCATGGTGAGCAAGGGCGAGGAGGATAACATGGCCATCATCAAGGAGTTCAT
GCGCTTCAAGGTGCACATGGAGGGCTCCGTGAACGGCCACGAGTTCGAGATCGAGGGCGAGGGCGAGGGC
CGCCCCTACGAGGGCACCCAGACCGCCAAGCTGAAGGTGACCAAGGGTGGCCCCCTGCCCTTCGCCTGGG
ACATCCTGTCCCCTCAGTTCATGTACGGCTCCAAGGCCTACGTGAAGCACCCCGCCGACATCCCCGACTA
CTTGAAGCTGTCCTTCCCCGAGGGCTTCAAGTGGGAGCGCGTGATGAACTTCGAGGACGGCGGCGTGGTG
ACCGTGACCCAGGACTCCTCCCTGCAGGACGGCGAGTTCATCTACAAGGTGAAGCTGCGCGGCACCAACT
TCCCCTCCGACGGCCCCGTAATGCAGAAGAAGACCATGGGCTGGGAGGCCTCCTCCGAGCGGATGTACCC
CGAGGACGGCGCCCTGAAGGGCGAGATCAAGCAGAGGCTGAAGCTGAAGGACGGCGGCCACTACGACGCT
GAGGTCAAGACCACCTACAAGGCCAAGAAGCCCGTGCAGCTGCCCGGCGCCTACAACGTCAACATCAAGT
TGGACATCACCTCCCACAACGAGGACTACACCATCGTGGAACAGTACGAACGCGCCGAGGGCCGCCACTC
CACCGGCGGCATGGACGAGCTGTACAAGTAAAGCGGCCGCAGCTCGCTTTCTTGCTGTCCAATTTCTATT
AAAGGTTCCTTTGTTCCCTAAGTCCAACTACTAAACTGGGGGATATTATGAAGGGCCTTGAGCATTTGGA
TTCTGCCTAATAAAAAACATTTATTTTCATTGCGTTTAGCTCGCTTTCTTGCTGTCCAATTTCTATTAAA
GGTTCCTTTGTTCCCTAAGTCCAACTACTAAACTGGGGGATATTATGAAGGGCCTTGAGCATTTGGATTC
TGCCTAATAAAAAACATTTATTTTCATTGCATTTAAATGTTTAAACAAAAAAAAAAAAAAAAAAAAAAAA
AAAAAAAAAAAAAAAAAAAAAAAAAAAAAAAAAAAAAAAAAAAAAAAAAAAAAAAAAAAAAAAAAAAAAA
AAAAAAAAAAAAAAAAAAAAAAAAAAAAAAGCAGGTGGGAGGGTACGTTCGTGATAAACAAACGGCCGCT
TCGAGCAGACATGATAAGATACATTGATGAGTTTGGACAAACCACAACTAGAATGCAGTGAAAAAAATGC
TTTATTTGTGAAATTTGTGATGCTATTGCTTTATTTGTAACCATTATAAGCTGCAATAAACAAGTTAACA
ACAACAATTGCATTCATTTTATGTTTCAGGTTCAGGGGGAGATGTGGGAGGTTTTTTAAAGCAAGTAAAA
CCTCTACAAATGTGGTAAAATCGATAAGGATCAGGGCTGGCGTAATAGCGAAGAGGCCCGCACCGATCGC
CCTTCCCAACAGTTGCGCAGCCTGAATGGCGAATGGACGCGCCCTGTAGCGGCGCATTAAGCGCGGCGGG
TGTGGTGGTTACGCGCAGCGTGACCGCTACACTTGCCAGCGCCCTAGCGCCCGCTCCTTTCGCTTTCTTC
CCTTCCTTTCTCGCCACGTTCGCCGGCTTTCCCCGTCAAGCTCTAAATCGGGGGCTCCCTTTAGGGTTCC
GATTTAGTGCTTTACGGCACCTCGACCCCAAAAAACTTGATTAGGGTGATGGTTCACGTAGTGGGCCATC
GCCCTGATAGACGGTTTTTCGCCCTTTGACGTTGGAGTCCACGTTCTTTAATAGTGGACTCTTGTTCCAA
ACTGGAACAACACTCAACCCTATCTCGGTCTATTCTTTTGATTTATAAGGGATTTTGCCGATTTCGGCCT
ATTGGTTAAAAAATGAGCTGATTTAACAAAAATTTAACGCGAATTTTAACAAAATATTAACGCTTACAAT
TTCCTGATGCGGTATTTTCTCCTTACGCATCTGTGCGGTATTTCACACCGCATATGGTGCACTCTCAGTA
CAATCTGCTCTGATGCCGCATAGTTAAGCCAGCCCCGACACCCGCCAACACCCGCTGACGCGCCCTGACG
GGCTTGTCTGCTCCCGGCATCCGCTTACAGACAAGCTGTGACCGTCTCCGGGAGCTGCATGTGTCAGAGG
TTTTCACCGTCATCACCGAAACGCGCGAGACGAAAGGGCCTCGTGATACGCCTATTTTTATAGGTTAATG
TCATGATAATAATGGTTTCTTAGACGTCAGGTGGCACTTTTCGGGGAAATGTGCGCGGAACCCCTATTTG
TTTATTTTTCTAAATACATTCAAATATGTATCCGCTCATGAGACAATAACCCTGATAAATGCTTCAATAA
TATTGAAAAAGGAAGAGTATGATTGAACAAGATGGATTGCACGCAGGTTCTCCGGCCGCTTGGGTGGAGA
GGCTATTCGGCTATGACTGGGCACAACAGACAATCGGCTGCTCTGATGCCGCCGTGTTCCGGCTGTCAGC
GCAGGGGCGCCCGGTTCTTTTTGTCAAGACCGACCTGTCCGGTGCCCTGAATGAACTGCAAGACGAGGCA
GCGCGGCTATCGTGGCTGGCCACGACGGGCGTTCCTTGCGCAGCTGTGCTCGACGTTGTCACTGAAGCGG
GAAGGGACTGGCTGCTATTGGGCGAAGTGCCGGGGCAGGATCTCCTGTCATCTCACCTTGCTCCTGCCGA
GAAAGTATCCATCATGGCTGATGCAATGCGGCGGCTGCATACGCTTGATCCGGCTACCTGCCCATTCGAC
CACCAAGCGAAACATCGCATCGAGCGAGCACGTACTCGGATGGAAGCCGGTCTTGTCGATCAGGATGATC
TGGACGAAGAGCATCAGGGGCTCGCGCCAGCCGAACTGTTCGCCAGGCTCAAGGCGAGCATGCCCGACGG
CGAGGATCTCGTCGTGACCCATGGCGATGCCTGCTTGCCGAATATCATGGTGGAAAATGGCCGCTTTTCT
GGATTCATCGACTGTGGCCGGCTGGGTGTGGCGGACCGCTATCAGGACATAGCGTTGGCTACCCGTGATA

TTGCTGAAGAGCTTGGCGGCGAATGGGCTGACCGCTTCCTCGTGCTTTACGGTATCGCCGCTCCCGATTC
GCAGCGCATCGCCTTCTATCGCCTTCTTGACGAGTTCTTCTGACTGTCAGACCAAGTTTACTCATATATA
CTTTAGATTGATTTAAAACTTCATTTTTAATTTAAAAGGATCTAGGTGAAGATCCTTTTTGATAATCTCA

pmRNA-iEMCV-EGFP-A120

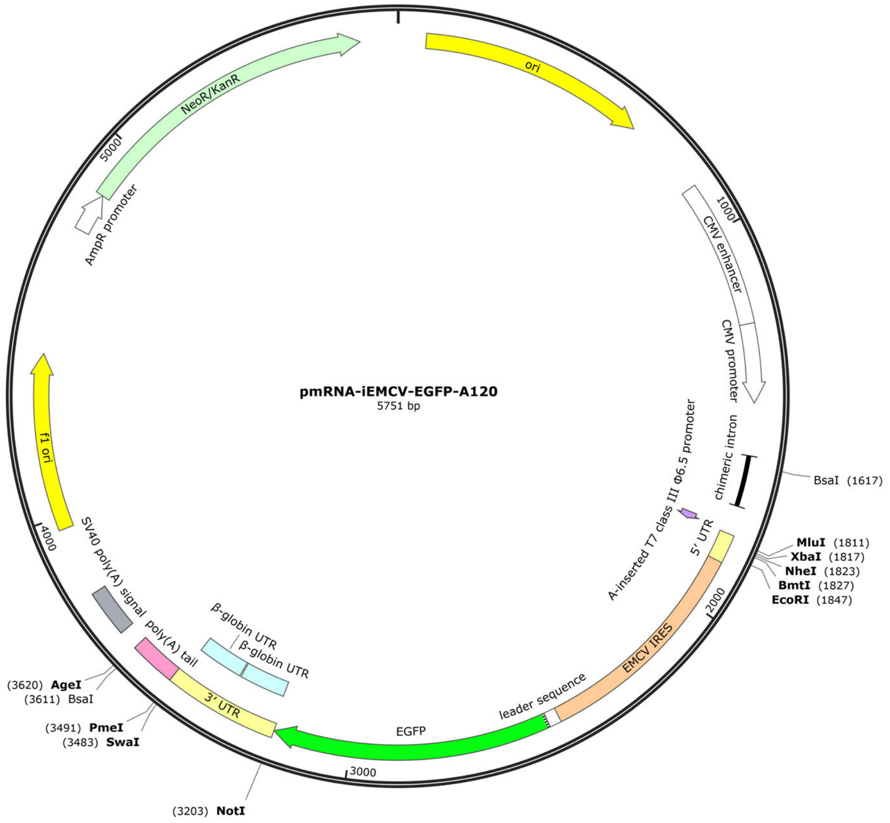

TGACCAAAATCCCTTAACGTGAGTTTTCGTTCCACTGAGCGTCAGACCCCGTAGAAAAGATCAAAGGATC
TTCTTGAGATCCTTTTTTTCTGCGCGTAATCTGCTGCTTGCAAACAAAAAAACCACCGCTACCAGCGGTG
GTTTGTTTGCCGGATCAAGAGCTACCAACTCTTTTTCCGAAGGTAACTGGCTTCAGCAGAGCGCAGATAC
CAAATACTGTTCTTCTAGTGTAGCCGTAGTTAGGCCACCACTTCAAGAACTCTGTAGCACCGCCTACATA
CCTCGCTCTGCTAATCCTGTTACCAGTGGCTGCTGCCAGTGGCGATAAGTCGTGTCTTACCGGGTTGGAC
TCAAGACGATAGTTACCGGATAAGGCGCAGCGGTCGGGCTGAACGGGGGGTTCGTGCACACAGCCCAGCT
TGGAGCGAACGACCTACACCGAACTGAGATACCTACAGCGTGAGCTATGAGAAAGCGCCACGCTTCCCGA
AGGGAGAAAGGCGGACAGGTATCCGGTAAGCGGCAGGGTCGGAACAGGAGAGCGCACGAGGGAGCTTCCA
GGGGGAAACGCCTGGTATCTTTATAGTCCTGTCGGGTTTCGCCACCTCTGACTTGAGCGTCGATTTTTGT
GATGCTCGTCAGGGGGGCGGAGCCTATGGAAAAACGCCAGCAACGCGGCCTTTTTACGGTTCCTGGCCTT
TTGCTGGCCTTTTGCTCACATGGCTCGACAGATCATCAATATTGGCCATTAGCCATATTATTCATTGGTT
ATATAGCATAAATCAATATTGGCTATTGGCCATTGCATACGTTGTATCTATATCATAATATGTACATTTA
TATTGGCTCATGTCCAATATGACCGCCATGTTGGCATTGATTATTGACTAGTTATTAATAGTAATCAATT
ACGGGGTCATTAGTTCATAGCCCATATATGGAGTTCCGCGTTACATAACTTACGGTAAATGGCCCGCCTG
GCTGACCGCCCAACGACCCCCGCCCATTGACGTCAATAATGACGTATGTTCCCATAGTAACGCCAATAGG
GACTTTCCATTGACGTCAATGGGTGGAGTATTTACGGTAAACTGCCCACTTGGCAGTACATCAAGTGTAT
CATATGCCAAGTCCGCCCCCTATTGACGTCAATGACGGTAAATGGCCCGCCTGGCATTATGCCCAGTACA
TGACCTTACGGGACTTTCCTACTTGGCAGTACATCTACGTATTAGTCATCGCTATTACCATGGTGATGCG
GTTTTGGCAGTACACCAATGGGCGTGGATAGCGGTTTGACTCACGGGGATTTCCAAGTCTCCACCCCATT
GACGTCAATGGGAGTTTGTTTTGGCACCAAAATCAACGGGACTTTCCAAAATGTCGTAATAACCCCGCCC
CGTTGACGCAAATGGGCGGTAGGCGTGTACGGTGGGAGGTCTATATAAGCAGAGCTCGTTTAGTGAACCG

TCAGATCACTAGACGCTTTATTGCGGTAGTTTATCACAGTTAAATTGCTAACGCAGTCAGTGCTTCTGAC
ACAACAGTCTCGAACTTAAGCTGCAGAAGTTGGTCGTGAGGCACTGGGCAGGTAAGTATCAAGGTTACAA
GACAGGTTTAAGGAGACCAATAGAAACTGGGCTTGTCGAGACAGAGAAGACTCTTGCGTTTCTGATAGGC
ACCTATTGGTCTTACTGACATCCACTTTGCCTTTCTCTCCACAGGTGTCCACTCCCAGTTCAATTACAGC
TCTTAAGGCTAGAGTCACCTGCAACATGACTTAATACGACTCACTATAAGGAGAGATATCACGCGTTCTA
GAGCTAGCCTCGATAAGCTTGATATCGAATTCCGCCCCCCCCCCCTCTCCCTCCCCCCCCCCTAACGTTA
CTGGCCGAAGCCGCTTGGAATAAGGCCGGTGTGCGTTTGTCTATATGTTATTTTCCACCATATTGCCGTC
TTTTGGCAATGTGAGGGCCCGGAAACCTGGCCCTGTCTTCTTGACGAGCATTCCTAGGGGTCTTTCCCCT
CTCGCCAAAGGAATGCAAGGTCTGTTGAATGTCGTGAAGGAAGCAGTTCCTCTGGAAGCTTCTTGAAGAC
AAACAACGTCTGTAGCGACCCTTTGCAGGCAGCGGAACCCCCCACCTGGCGACAGGTGCCTCTGCGGCCA
AAAGCCACGTGTATAAGATACACCTGCAAAGGCGGCACAACCCCAGTGCCACGTTGTGAGTTGGATAGTT
GTGGAAAGAGTCAAATGGCTCTCCTCAAGCGTATTCAACAAGGGGCTGAAGGATGCCCAGAAGGTACCCC
ATTGTATGGGATCTGATCTGGGGCCTCGGTGCACATGCTTTACATGTGTTTAGTCGAGGTTAAAAAAACG
TCTAGGCCCCCCGAACCACGGGGACGTGGTTTTCCTTTGAAAAACACGATGATAATATGACGATGATAAT
ATGACTTCGAAAGTTTATGATCCAGAACAAATGGTGAGCAAGGGCGAGGAGCTGTTCACCGGGGTGGTGC
CCATCCTGGTCGAGCTGGACGGCGACGTAAACGGCCACAAGTTCAGCGTGTCCGGCGAGGGCGAGGGCGA
TGCCACCTACGGCAAGCTGACCCTGAAGTTCATCTGCACCACCGGCAAGCTGCCCGTGCCCTGGCCCACC
CTCGTGACCACCCTGACCTACGGCGTGCAGTGCTTCAGCCGCTACCCCGACCACATGAAGCAGCACGACT
TCTTCAAGTCCGCCATGCCCGAAGGCTACGTCCAGGAGCGCACCATCTTCTTCAAGGACGACGGCAACTA
CAAGACCCGCGCCGAGGTGAAGTTCGAGGGCGACACCCTGGTGAACCGCATCGAGCTGAAGGGCATCGAC
TTCAAGGAGGACGGCAACATCCTGGGGCACAAGCTGGAGTACAACTACAACAGCCACAACGTCTATATCA
TGGCCGACAAGCAGAAGAACGGCATCAAGGTGAACTTCAAGATCCGCCACAACATCGAGGACGGCAGCGT
GCAGCTCGCCGACCACTACCAGCAGAACACCCCCATCGGCGACGGCCCCGTGCTGCTGCCCGACAACCAC
TACCTGAGCACCCAGTCCGCCCTGAGCAAAGACCCCAACGAGAAGCGCGATCACATGGTCCTGCTGGAGT
TCGTGACCGCCGCCGGGATCACTCTCGGCATGGACGAGCTGTACAAGTAAAGCGGCCGCAGCTCGCTTTC
TTGCTGTCCAATTTCTATTAAAGGTTCCTTTGTTCCCTAAGTCCAACTACTAAACTGGGGGATATTATGA
AGGGCCTTGAGCATTTGGATTCTGCCTAATAAAAAACATTTATTTTCATTGCGTTTAGCTCGCTTTCTTG
CTGTCCAATTTCTATTAAAGGTTCCTTTGTTCCCTAAGTCCAACTACTAAACTGGGGGATATTATGAAGG
GCCTTGAGCATTTGGATTCTGCCTAATAAAAAACATTTATTTTCATTGCATTTAAATGTTTAAACAAAAA
AAAAAAAAAAAAAAAAAAAAAAAAAAAAAAAAAAAAAAAAAAAAAAAAAAAAAAAAAAAAAAAAAAAAAA
AAAAAAAAAAAAAAAAAAAAAAAAAAAAAAAAAAAAAAAAAAAAAAAAGAGACCGGTACGTTCGTGATAAAC
AAACGGCCGCTTCGAGCAGACATGATAAGATACATTGATGAGTTTGGACAAACCACAACTAGAATGCAGT
GAAAAAAATGCTTTATTTGTGAAATTTGTGATGCTATTGCTTTATTTGTAACCATTATAAGCTGCAATAA
ACAAGTTAACAACAACAATTGCATTCATTTTATGTTTCAGGTTCAGGGGGAGATGTGGGAGGTTTTTTAA
AGCAAGTAAAACCTCTACAAATGTGGTAAAATCGATAAGGATCAGGGCTGGCGTAATAGCGAAGAGGCCC
GCACCGATCGCCCTTCCCAACAGTTGCGCAGCCTGAATGGCGAATGGACGCGCCCTGTAGCGGCGCATTA
AGCGCGGCGGGTGTGGTGGTTACGCGCAGCGTGACCGCTACACTTGCCAGCGCCCTAGCGCCCGCTCCTT
TCGCTTTCTTCCCTTCCTTTCTCGCCACGTTCGCCGGCTTTCCCCGTCAAGCTCTAAATCGGGGGCTCCC
TTTAGGGTTCCGATTTAGTGCTTTACGGCACCTCGACCCCAAAAAACTTGATTAGGGTGATGGTTCACGT
AGTGGGCCATCGCCCTGATAGACGGTTTTTCGCCCTTTGACGTTGGAGTCCACGTTCTTTAATAGTGGAC
TCTTGTTCCAAACTGGAACAACACTCAACCCTATCTCGGTCTATTCTTTTGATTTATAAGGGATTTTGCC
GATTTCGGCCTATTGGTTAAAAAATGAGCTGATTTAACAAAAATTTAACGCGAATTTTAACAAAATATTA
ACGCTTACAATTTCCTGATGCGGTATTTTCTCCTTACGCATCTGTGCGGTATTTCACACCGCATATGGTG
CACTCTCAGTACAATCTGCTCTGATGCCGCATAGTTAAGCCAGCCCCGACACCCGCCAACACCCGCTGAC
GCGCCCTGACGGGCTTGTCTGCTCCCGGCATCCGCTTACAGACAAGCTGTGACCGTCTCCGGGAGCTGCA
TGTGTCAGAGGTTTTCACCGTCATCACCGAAACGCGCGAGACGAAAGGGCCTCGTGATACGCCTATTTTT
ATAGGTTAATGTCATGATAATAATGGTTTCTTAGACGTCAGGTGGCACTTTTCGGGGAAATGTGCGCGGA

ACCCCTATTTGTTTATTTTTCTAAATACATTCAAATATGTATCCGCTCATGAGACAATAACCCTGATAAA
TGCTTCAATAATATTGAAAAAGGAAGAGTATGATTGAACAAGATGGATTGCACGCAGGTTCTCCGGCCGC
TTGGGTGGAGAGGCTATTCGGCTATGACTGGGCACAACAGACAATCGGCTGCTCTGATGCCGCCGTGTTC
CGGCTGTCAGCGCAGGGGCGCCCGGTTCTTTTTGTCAAGACCGACCTGTCCGGTGCCCTGAATGAACTGC
AAGACGAGGCAGCGCGGCTATCGTGGCTGGCCACGACGGGCGTTCCTTGCGCAGCTGTGCTCGACGTTGT
CACTGAAGCGGGAAGGGACTGGCTGCTATTGGGCGAAGTGCCGGGGCAGGATCTCCTGTCATCTCACCTT
GCTCCTGCCGAGAAAGTATCCATCATGGCTGATGCAATGCGGCGGCTGCATACGCTTGATCCGGCTACCT
GCCCATTCGACCACCAAGCGAAACATCGCATCGAGCGAGCACGTACTCGGATGGAAGCCGGTCTTGTCGA
TCAGGATGATCTGGACGAAGAGCATCAGGGGCTCGCGCCAGCCGAACTGTTCGCCAGGCTCAAGGCGAGC
ATGCCCGACGGCGAGGATCTCGTCGTGACCCATGGCGATGCCTGCTTGCCGAATATCATGGTGGAAAATG
GCCGCTTTTCTGGATTCATCGACTGTGGCCGGCTGGGTGTGGCGGACCGCTATCAGGACATAGCGTTGGC
TACCCGTGATATTGCTGAAGAGCTTGGCGGCGAATGGGCTGACCGCTTCCTCGTGCTTTACGGTATCGCC
GCTCCCGATTCGCAGCGCATCGCCTTCTATCGCCTTCTTGACGAGTTCTTCTGACTGTCAGACCAAGTTT
ACTCATATATACTTTAGATTGATTTAAAACTTCATTTTTAATTTAAAAGGATCTAGGTGAAGATCCTTTT
TGATAATCTCA

pmRNA-iHCV-EGFP-A120

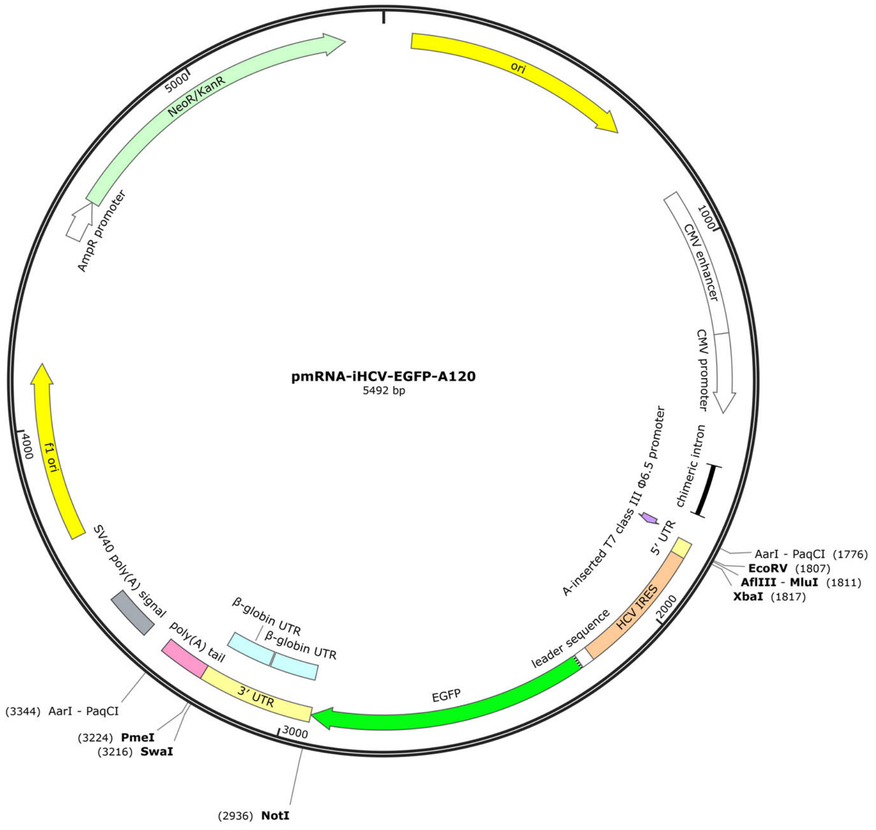

TGACCAAAATCCCTTAACGTGAGTTTTCGTTCCACTGAGCGTCAGACCCCGTAGAAAAGATCAAAGGATC
TTCTTGAGATCCTTTTTTTCTGCGCGTAATCTGCTGCTTGCAAACAAAAAAACCACCGCTACCAGCGGTG
GTTTGTTTGCCGGATCAAGAGCTACCAACTCTTTTTCCGAAGGTAACTGGCTTCAGCAGAGCGCAGATAC
CAAATACTGTTCTTCTAGTGTAGCCGTAGTTAGGCCACCACTTCAAGAACTCTGTAGCACCGCCTACATA
CCTCGCTCTGCTAATCCTGTTACCAGTGGCTGCTGCCAGTGGCGATAAGTCGTGTCTTACCGGGTTGGAC
TCAAGACGATAGTTACCGGATAAGGCGCAGCGGTCGGGCTGAACGGGGGGTTCGTGCACACAGCCCAGCT
TGGAGCGAACGACCTACACCGAACTGAGATACCTACAGCGTGAGCTATGAGAAAGCGCCACGCTTCCCGA
AGGGAGAAAGGCGGACAGGTATCCGGTAAGCGGCAGGGTCGGAACAGGAGAGCGCACGAGGGAGCTTCCA
GGGGGAAACGCCTGGTATCTTTATAGTCCTGTCGGGTTTCGCCACCTCTGACTTGAGCGTCGATTTTTGT

GATGCTCGTCAGGGGGGCGGAGCCTATGGAAAAACGCCAGCAACGCGGCCTTTTTACGGTTCCTGGCCTT
TTGCTGGCCTTTTGCTCACATGGCTCGACAGATCATCAATATTGGCCATTAGCCATATTATTCATTGGTT
ATATAGCATAAATCAATATTGGCTATTGGCCATTGCATACGTTGTATCTATATCATAATATGTACATTTA
TATTGGCTCATGTCCAATATGACCGCCATGTTGGCATTGATTATTGACTAGTTATTAATAGTAATCAATT
ACGGGGTCATTAGTTCATAGCCCATATATGGAGTTCCGCGTTACATAACTTACGGTAAATGGCCCGCCTG
GCTGACCGCCCAACGACCCCCGCCCATTGACGTCAATAATGACGTATGTTCCCATAGTAACGCCAATAGG
GACTTTCCATTGACGTCAATGGGTGGAGTATTTACGGTAAACTGCCCACTTGGCAGTACATCAAGTGTAT
CATATGCCAAGTCCGCCCCCTATTGACGTCAATGACGGTAAATGGCCCGCCTGGCATTATGCCCAGTACA
TGACCTTACGGGACTTTCCTACTTGGCAGTACATCTACGTATTAGTCATCGCTATTACCATGGTGATGCG
GTTTTGGCAGTACACCAATGGGCGTGGATAGCGGTTTGACTCACGGGGATTTCCAAGTCTCCACCCCATT
GACGTCAATGGGAGTTTGTTTTGGCACCAAAATCAACGGGACTTTCCAAAATGTCGTAATAACCCCGCCC
CGTTGACGCAAATGGGCGGTAGGCGTGTACGGTGGGAGGTCTATATAAGCAGAGCTCGTTTAGTGAACCG
TCAGATCACTAGACGCTTTATTGCGGTAGTTTATCACAGTTAAATTGCTAACGCAGTCAGTGCTTCTGAC
ACAACAGTCTCGAACTTAAGCTGCAGAAGTTGGTCGTGAGGCACTGGGCAGGTAAGTATCAAGGTTACAA
GACAGGTTTAAGGAGACCAATAGAAACTGGGCTTGTCGAGACAGAGAAGACTCTTGCGTTTCTGATAGGC
ACCTATTGGTCTTACTGACATCCACTTTGCCTTTCTCTCCACAGGTGTCCACTCCCAGTTCAATTACAGC
TCTTAAGGCTAGAGTCACCTGCAACATGACTTAATACGACTCACTATAAGGAGAGATATCACGCGTTCTA
GAGCTAGCTAGTGTACGTAGCCAGCCCCCGATTGGGGGCGACACTCCACCATAGATCACTCCCCTGTGAG
GAACTACTGTCTTCACGCAGAAAGCGTCTAGCCATGGCGTTAGTATGAGAGTCGTGCAGCCTCCAGGACC
CCCCCTCCCGGGAGAGCCATAGTGGTCTGCGGAACCGGTGAGTACACCGGAATTGCCAGGACGACCGGGT
CCTTTCTTGGATCAACCCGCTCAATGCCTGGAGATTTGGGCGTGCCCCCGCAAGACTGCTAGCCGAGTAG
TGTTGGGTCGCGAAAGGCCTTGTGGTACTGCCTGATAGGGTGCTTGCGAGTGCCCCGGGAGGTCTCGTAG
ACCGTGCACCATGAGCACGAATCCTAAACCTCAAAGAAAAACCATGGTGAGCAAGGGCGAGGAGCTGTTC
ACCGGGGTGGTGCCCATCCTGGTCGAGCTGGACGGCGACGTAAACGGCCACAAGTTCAGCGTGTCCGGCG
AGGGCGAGGGCGATGCCACCTACGGCAAGCTGACCCTGAAGTTCATCTGCACCACCGGCAAGCTGCCCGT
GCCCTGGCCCACCCTCGTGACCACCCTGACCTACGGCGTGCAGTGCTTCAGCCGCTACCCCGACCACATG
AAGCAGCACGACTTCTTCAAGTCCGCCATGCCCGAAGGCTACGTCCAGGAGCGCACCATCTTCTTCAAGG
ACGACGGCAACTACAAGACCCGCGCCGAGGTGAAGTTCGAGGGCGACACCCTGGTGAACCGCATCGAGCT
GAAGGGCATCGACTTCAAGGAGGACGGCAACATCCTGGGGCACAAGCTGGAGTACAACTACAACAGCCAC
AACGTCTATATCATGGCCGACAAGCAGAAGAACGGCATCAAGGTGAACTTCAAGATCCGCCACAACATCG
AGGACGGCAGCGTGCAGCTCGCCGACCACTACCAGCAGAACACCCCCATCGGCGACGGCCCCGTGCTGCT
GCCCGACAACCACTACCTGAGCACCCAGTCCGCCCTGAGCAAAGACCCCAACGAGAAGCGCGATCACATG
GTCCTGCTGGAGTTCGTGACCGCCGCCGGGATCACTCTCGGCATGGACGAGCTGTACAAGTAAAGCGGCC
GCAGCTCGCTTTCTTGCTGTCCAATTTCTATTAAAGGTTCCTTTGTTCCCTAAGTCCAACTACTAAACTG
GGGGATATTATGAAGGGCCTTGAGCATTTGGATTCTGCCTAATAAAAAACATTTATTTTCATTGCGTTTA
GCTCGCTTTCTTGCTGTCCAATTTCTATTAAAGGTTCCTTTGTTCCCTAAGTCCAACTACTAAACTGGGG
GATATTATGAAGGGCCTTGAGCATTTGGATTCTGCCTAATAAAAAACATTTATTTTCATTGCATTTAAAT
GTTTAAACAAAAAAAAAAAAAAAAAAAAAAAAAAAAAAAAAAAAAAAAAAAAAAAAAAAAAAAAAAAAAA
AAAAAAAAAAAAAAAAAAAAAAAAAAAAAAAAAAAAAAAAAAAAAAAAAAAAAAAAAAAAAAGCAGGTGG
GAGGGTACGTTCGTGATAAACAAACGGCCGCTTCGAGCAGACATGATAAGATACATTGATGAGTTTGGAC
AAACCACAACTAGAATGCAGTGAAAAAAATGCTTTATTTGTGAAATTTGTGATGCTATTGCTTTATTTGT
AACCATTATAAGCTGCAATAAACAAGTTAACAACAACAATTGCATTCATTTTATGTTTCAGGTTCAGGGG
GAGATGTGGGAGGTTTTTTAAAGCAAGTAAAACCTCTACAAATGTGGTAAAATCGATAAGGATCAGGGCT
GGCGTAATAGCGAAGAGGCCCGCACCGATCGCCCTTCCCAACAGTTGCGCAGCCTGAATGGCGAATGGAC
GCGCCCTGTAGCGGCGCATTAAGCGCGGCGGGTGTGGTGGTTACGCGCAGCGTGACCGCTACACTTGCCA
GCGCCCTAGCGCCCGCTCCTTTCGCTTTCTTCCCTTCCTTTCTCGCCACGTTCGCCGGCTTTCCCCGTCA
AGCTCTAAATCGGGGGCTCCCTTTAGGGTTCCGATTTAGTGCTTTACGGCACCTCGACCCCAAAAAACTT

GATTAGGGTGATGGTTCACGTAGTGGGCCATCGCCCTGATAGACGGTTTTTCGCCCTTTGACGTTGGAGT
CCACGTTCTTTAATAGTGGACTCTTGTTCCAAACTGGAACAACACTCAACCCTATCTCGGTCTATTCTTT
TGATTTATAAGGGATTTTGCCGATTTCGGCCTATTGGTTAAAAAATGAGCTGATTTAACAAAAATTTAAC
GCGAATTTTAACAAAATATTAACGCTTACAATTTCCTGATGCGGTATTTTCTCCTTACGCATCTGTGCGG
TATTTCACACCGCATATGGTGCACTCTCAGTACAATCTGCTCTGATGCCGCATAGTTAAGCCAGCCCCGA
CACCCGCCAACACCCGCTGACGCGCCCTGACGGGCTTGTCTGCTCCCGGCATCCGCTTACAGACAAGCTG
TGACCGTCTCCGGGAGCTGCATGTGTCAGAGGTTTTCACCGTCATCACCGAAACGCGCGAGACGAAAGGG
CCTCGTGATACGCCTATTTTTATAGGTTAATGTCATGATAATAATGGTTTCTTAGACGTCAGGTGGCACT
TTTCGGGGAAATGTGCGCGGAACCCCTATTTGTTTATTTTTCTAAATACATTCAAATATGTATCCGCTCA
TGAGACAATAACCCTGATAAATGCTTCAATAATATTGAAAAAGGAAGAGTATGATTGAACAAGATGGATT
GCACGCAGGTTCTCCGGCCGCTTGGGTGGAGAGGCTATTCGGCTATGACTGGGCACAACAGACAATCGGC
TGCTCTGATGCCGCCGTGTTCCGGCTGTCAGCGCAGGGGCGCCCGGTTCTTTTTGTCAAGACCGACCTGT
CCGGTGCCCTGAATGAACTGCAAGACGAGGCAGCGCGGCTATCGTGGCTGGCCACGACGGGCGTTCCTTG
CGCAGCTGTGCTCGACGTTGTCACTGAAGCGGGAAGGGACTGGCTGCTATTGGGCGAAGTGCCGGGGCAG
GATCTCCTGTCATCTCACCTTGCTCCTGCCGAGAAAGTATCCATCATGGCTGATGCAATGCGGCGGCTGC
ATACGCTTGATCCGGCTACCTGCCCATTCGACCACCAAGCGAAACATCGCATCGAGCGAGCACGTACTCG
GATGGAAGCCGGTCTTGTCGATCAGGATGATCTGGACGAAGAGCATCAGGGGCTCGCGCCAGCCGAACTG
TTCGCCAGGCTCAAGGCGAGCATGCCCGACGGCGAGGATCTCGTCGTGACCCATGGCGATGCCTGCTTGC
CGAATATCATGGTGGAAAATGGCCGCTTTTCTGGATTCATCGACTGTGGCCGGCTGGGTGTGGCGGACCG
CTATCAGGACATAGCGTTGGCTACCCGTGATATTGCTGAAGAGCTTGGCGGCGAATGGGCTGACCGCTTC
CTCGTGCTTTACGGTATCGCCGCTCCCGATTCGCAGCGCATCGCCTTCTATCGCCTTCTTGACGAGTTCT
TCTGACTGTCAGACCAAGTTTACTCATATATACTTTAGATTGATTTAAAACTTCATTTTTAATTTAAAAG
GATCTAGGTGAAGATCCTTTTTGATAATCTCA

pmRNA-iSyn-EGFP

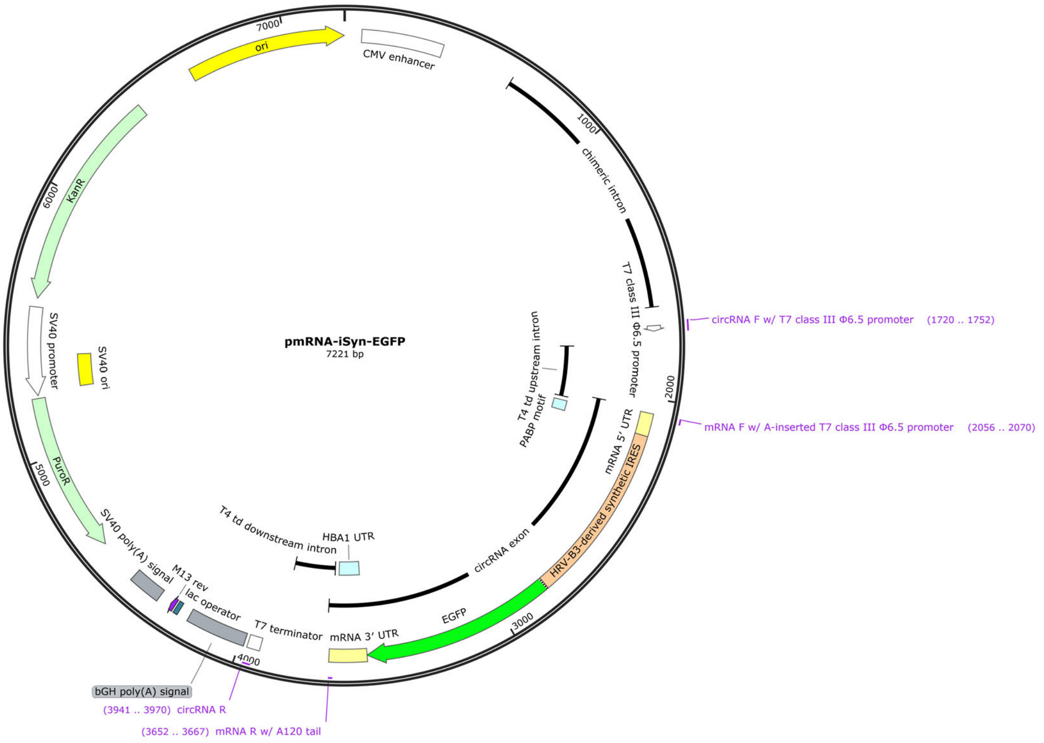

CCTGCAGGTTATTAATAGTAATCAATTACGGGGTCATTAGTTCATAGCCCATATATGGAGTTCCGCGTTA
CATAACTTACGGTAAATGGCCCGCCTGGCTGACCGCCCAACGACCCCCGCCCATTGACGTCAATAATGAC
GTATGTTCCCATAGTAACGCCAATAGGGACTTTCCATTGACGTCAATGGGTGGAGTATTTACGGTAAACT
GCCCACTTGGCAGTACATCAAGTGTATCATATGCCAAGTACGCCCCCTATTGACGTCAATGACGGTAAAT

GGCCCGCCTGGCATTATGCCCAGTACATGACCTTATGGGACTTTCCTACTTGGCAGTACATCTACGTATT
AGTCATCGCTATTACCATGGTCGAGGTGAGCCCCACGTTCTGCTTCACTCTCCCCATCTCCCCCCCCTCC
CCACCCCCAATTTTGTATTTATTTATTTTTTAATTATTTTGTGCAGCGATGGGGGCGGGGGGGGGGGGG
GGCGCGCCAGGCGGGGCGGGGCGGGGCGAGGGGCGGGGCGGGGCGAGGCGGAGAGGTGCGGCGGCAGCCA
ATCAGAGCGGCGCGCTCCGAAAGTTTCCTTTTATGGCGAGGCGGCGGCGGCGGCGGCCCTATAAAAAGCG
AAGCGCGCGGCGGGCGGGAGTCGCTGCGCGCTGCCTTCGCCCCGTGCCCCGCTCCGCCGCCGCCTCGCGC
CGCCCGCCCCGGCTCTGACTGACCGCGTTACTCCCACAGGTGAGCGGGCGGGACGGCCCTTCTCCTCCGG
GCTGTAATTAGCGCTTGGTTTAATGACGGCTTGTTTCTTTTCTGTGGCTGCGTGAAAGCCTTGAGGGGCT
CCGGGAGGGCCCTTTGTGCGGGGGGAGCGGCTCGGGGGGTGCGTGCGTGTGTGTGTGCGTGGGGAGCGCC
GCGTGCGGCTCCGCGCTGCCCGGCGGCTGTGAGCGCTGCGGGCGCGGCGCGGGGCTTTGTGCGCTCCGCA
GTGTGCGCGAGGGGAGCGCGGCCGGGGGCGGTGCCCCGCGGTGCGGGGGGGGCTGCGAGGGGAACAAAGG
CTGCGTGCGGGGTGTGTGCGTGGGGGGGTGAGCAGGGGGTGTGGGCGCGTCGGTCGGGCTGCAACCCCCC
CTGCACCCCCCTCCCCGAGTTGCTGAGCACGGCCCGGCTTCGGGTGCGGGGCTCCGTACGGGGCGTGGCG
CGGGGCTCGCCGTGCCGGGCGGGGGGTGGCGGCAGGTGGGGGTGCCGGGCGGGGCGGGGCCGCCTCGGGC
CGGGGAGGGCTCGGGGGAGGGGCGCGGCGGCCCCCGGAGCGCCGGCGGCTGTCGAGGCGCGGCGAGCCGC
AGCCATTGCCTTTTATGGTAATCGTGCGAGAGGGCGCAGGGACTTCCTTTGTCCCAAATCTGTGCGGAGC
CGAAATCTGGGAGGCGCCGCCGCACCCCCTCTAGCGGGCGCGGGGCGAAGCGGTGCGGCGCCGGCAGGAA
GGAAATGGGCGGGGAGGGCCTTCGTGCGTCGCCGCGCCGCCGTCCCCTTCTCCCTCTCCAGCCTCGGGGC
TGTCCGCGGGGGGACGGCTGCCTTCGGGGGGGACGGGGCAGGGCGGGGTTCGGCTTCTGGCGTGTGACCG
GCGGCTCTAGAGCCTCTGCTAACCATGTTCATGCCTTCTTCTTTTTCCTACAGCTCCTGGGCAACGTGCT
GGTTATTGTGCTGTCTCATCATTTTGGCAAAGCGGCCGCGGCCAGTGAATTGTAATACGACTCACTATAG
GGGTTAACAAAAAAAAAAAAAAAAAAAAAAAAATCACCGACCGATCTATCTGAACCGGTGGGAATTCTAGA
GAAAATTTCGTCTGGATTAGTTACTTATCGTGTAAAATCTGATAAATGGAATTGGTTCTACATAAATGCC
TAACGACTATCCCTTTGGGGAGTAGGGTCAAGTGACTCGAAACGATAGACAACTTGCTTTAACAAGTTGG
AGATATAGTCTGCTCTGCATGGTGACATGCAGCTGGATATAATTCCGGGGTAAGATTAACGACCTTATCT
GAACATAACGCTACCGTTTAATATTGCGTCATATTCGGCGACCATTTGTGTGGTAAAAAAAAAAAAACCAA
AAAAAAAAAACAAAAAAAAAAAAATAATTGACTAAGATATCTTAAAACAGCGGATGGGTACCCCACCATCC
GACCCACTGGGTGTAGTACTCTGGTACTTCGTACCTTTGTACGCCTGTTCTTCCCATTGTACCCTTCCTG
AACTTCCAACCCAAGTAACGTTAGAAGCTCAACATTTAGTACAACAGGAAGCACCACATCCAGTGGTGTT
TAGTACAAGCACTTCTGTTTCCCCGGAGCGAGGTATAGGCTGTACCCACTGCCAAAAACCTTTAACCGTT
ATCCGCCAACCAACTACGTAAAAGCTAGTAGTATTATGTTTTTAACTAGGCGTTCGATCAGGTGGATTTC
CCCTCCACTAGTTTGGTCGATGAGGCTAGGAATTCCCCACGGGTGACCGTGTCCTAGCCTGCGTGGCGGC
CAACCCAGCCCACTCACTATTTGTTTTCGCGCCCAGTTGCAAAAAGTGTCGGGGCTGGACGCCTTTTTA
TAGACATGGTGTGAAGACTCGCATGTGCTTGGTTGTGATTCCTCCGGCCCCTGAATGCGGCTAACCTTAA
CCCTGGAGCCTTGTGTCACAAACCAGTGATGATAAGGTCGTAATGAGCAATTCCGGGACGGGACCGACTA
CTTTGGGTGTCCGTGTTTCTTATTTTTCTTATTATTGTCTTATGGTCACAGCATATATATAACATATACT
GTGATCATGGTGAGCAAGGGCGAGGAGCTGTTCACCGGGGTGGTGCCCATCCTGGTCGAGCTGGACGGCG
ACGTAAACGGCCACAAGTTCAGCGTGTCCGGCGAGGGCGAGGGCGATGCCACCTACGGCAAGCTGACCCT
GAAGTTCATCTGCACCACCGGCAAGCTGCCCGTGCCCTGGCCCACCCTCGTGACCACCCTGACCTACGGC
GTGCAGTGCTTCAGCCGCTACCCCGACCACATGAAGCAGCACGACTTCTTCAAGTCCGCCATGCCCGAAG
GCTACGTCCAGGAGCGCACCATCTTCTTCAAGGACGACGGCAACTACAAGACCCGCGCCGAGGTGAAGTT
CGAGGGCGACACCCTGGTGAACCGCATCGAGCTGAAGGGCATCGACTTCAAGGAGGACGGCAACATCCTG
GGGCACAAGCTGGAGTACAACTACAACAGCCACAACGTCTATATCATGGCCGACAAGCAGAAGAACGGCA
TCAAGGTGAACTTCAAGATCCGCCACAACATCGAGGACGGCAGCGTGCAGCTCGCCGACCACTACCAGCA
GAACACCCCCATCGGCGACGGCCCCGTGCTGCTGCCCGACAACCACTACCTGAGCACCCAGTCCGCCCTG
AGCAAAGACCCCAACGAGAAGCGCGATCACATGGTCCTGCTGGAGTTCGTGACCGCCGCCGGGATCACTC
TCGGCATGGACGAGCTGTACAAGTAATAACAATTGGCTGGAGCCTCGGTGGCCATGCTTCTTGCCCCTTG

GGCCTCCCCCCAGCCCCTCCTCCCCTTCCTGCACCCGTACCCCCGTGGTCTTTGAATAAAGTCTGAACCA
CACAAATGGTCGCCGACTCAGTAGATGTTTTCTTGGGTTAATTGAGGCCTGAGTATAAGGTGACTTATAC
TTGTAATCTATCTAAACGGGGAACCTCTCTAGTAGACAATCCCGTGCTAAATTGTAGGACTGCCCTTTAA
TAAATACTTCTATATTTAAAGAGGTATTTATGAAAAGCGGAATTTATCAGATTAAAAATACTTTCTCTAG
AGTCGACCTGCAGACGCGTTCAGATAGATCGGTCGGTGAAAAAAAAAAAAAAAAAAAAAAAAAAAGCGGCC
GCCTAGCATAACCCCTTGGGGCCTCTAAACGGGTCTTGAGGGGTTTTTTGGCTAGCGGCCGGCCTGTGCC
TTCTAGTTGCCAGCCATCTGTTGTTTGCCCCTCCCCCGTGCCTTCCTTGACCCTGGAAGGTGCCACTCCC
ACTGTCCTTTCCTAATAAAATGAGGAAATTGCATCGCATTGTCTGAGTAGGTGTCATTCTATTCTGGGGG
GTGGGGTGGGGCAGGACAGCAAGGGGGAGGATTGGGAAGACAATAGCAGGCATGCTGGGGATGCGGTGGG
CTCTATGGCGGCGCGCCACTTTATGCTTCCGGCTCGTATGTTGTGTGGAATTGTGAGCGGATAACAATTT
CACACAGGAAACAGCTATGACCATGATTACGCCAAGCTCTAGCTAGAGGTCGACGGTATACAGACATGAT
AAGATACATTGATGAGTTTGGACAAACCACAACTAGAATGCAGTGAAAAAAATGCTTTATTTGTGAAATT
TGTGATGCTATTGCTTTATTTGTAACCATTATAAGCTGCAATAAACAAGTTGGGGTGGGCGAAGAACTCC
AGCATGAGATCCCCGCGCTGGAGGATCATCCAGCCGGCGTCCCGGAAAACGATTCCGAAGCCCAACCTTT
CATAGAAGGCGGCGGTGGAATCGAAATCTCGTGATGGCAGGTTGGGCGTCGCTTGGTCGGTCATTTCGAA
TCAGGCACCGGGCTTGCGGGTCATGCACCAGGTGCGCGGTCCTTCGGGCACCTCGACGTCGGCGGTGACG
GTGAAGCCGAGCCGCTCGTAGAAGGGGAGGTTGCGGGGCGCGGAGGTTTCCAGGAAGGCGGGCACCCCGG
CGCGCTCGGCCGCCTCCACTCCGGGGAGCACGACGGCGCTGCCCAGACCCTTGCCCTGGTGGTCGGGCGA
CACGCCGACGGTGGCCAGGAACCACGCGGGCTCCTTGGGCCGGTGCGGCGCCAGGAGGCCTTCCATCTGT
TGCTGCGCGGCCAGCCGGGAACCGCTCAACTCGGCCATGCGCGGGCCGATCTCGGCGAACACCGCCCCCG
CTTCGACGCTCTCCGGCGTGGTCCAGACCGCCACCGCGGCGCCGTCGTCCGCGACCCACACCTTGCCGAT
GTCGAGCCCGACGCGCGTGAGGAAGAGTTCTTGCAGCTCGGTGACCCGCTCGATGTGGCGGTCCGGATCG
ACGGTGTGGCGCGTGGCGGGGTAGTCGGCGAACGCGGCGGCGAGGGTGCGTACGGCCCTGGGGACGTCGT
CGCGGGTGGCGAGGCGCACCGTGGGCTTGTACTCGGTCATGCGGGAGCTTTTTGCAAAAGCCTAGGCCTC
CAAAAAAGCCTCCTCACTACTTCTGGAATAGCTCAGAGGCAGAGGCGGCCTCGGCCTCTGCATAAATAAA
AAAAATTAGTCAGCCATGGGGCGGAGAATGGGCGGAACTGGGCGGAGTTAGGGGCGGGATGGGCGGAGTT
AGGGGCGGGACTATGGTTGCTGACTAATTGAGATGCATGCTTTGCATACTTCTGCCTGCTGGGGAGCCTG
GGGACTTTCCACACCTGGTTGCTGACTAATTGAGATGCATGCTTTGCATACTTCTGCCTGCTGGGGAGCC
TGGGGACTTTCCACACCCTAACTGACACAGAGCTCGTGTTACAACCAATTAACCAATTCTGATTAGAAAA
ACTCATCGAGCATCAAATGAAACTGCAATTTATTCATATCAGGATTATCAATACCATATTTTTGAAAAAG
CCGTTTCTGTAATGAAGGAGAAAACTCACCGAGGCAGTTCCATAGGATGGCAAGATCCTGGTATCGGTCT
GCGATTCCGACTCGTCCAACATCAATACAACCTATTAATTTCCCCTCGTCAAAAATAAGGTTATCAAGTG
AGAAATCACCATGAGTGACGACTGAATCCGGTGAGAATGGCAAAAGCTTATGCATTTCTTTCCAGACTTG
TTCAACAGGCCAGCCATTACGCTCGTCATCAAAATCACTCGCATCAACCAAACCGTTATTCATTCGTGAT
TGCGCCTGAGCGAGGCGAAATACGCGATCGCTGTTAAAAGGACAATTACAAACAGGAATCGAATGCAACC
GGCGCAGGAACACTGCCAGCGCATCAACAATATTTTCACCTGAATCAGGATATTCTTCTAATACCTGGAA
TGCTGTTTTCCCGGGGATCGCAGTGGTGAGTAACCATGCATCATCAGGAGTACGGATAAAATGCTTGATG
GTCGGAAGAGGCATAAATTCCGTCAGCCAGTTTAGTCTGACCATCTCATCTGTAACATCATTGGCAACGC
TACCTTTGCCATGTTTCAGAAACAACTCTGGCGCATCGGGCTTCCCATACAATCGATAGATTGTCGCACC
TGATTGCCCGACATTATCGCGAGCCCATTTATACCCATATAAATCAGCATCCATGTTGGAATTTAATCGC
GGCCTGGAGCAAGACGTTTCCCGTTGAATATGGCTCATAACACCCCTTGTATTACTGTTTATGTAAGCAG
ACAGTTTTATTGTTCATGATGATATATTTTTATCTTGTGCAATGTAACATCAGAGATTTTGAGACACAAC
GTGGCTTTGTTGAATAAATCGAACTTTTGCTGAGTTGAAGGATCAGTCATGACCAAAATCCCTTAACGTG
AGTTTTCGTTCCACTGAGCGTCAGACCCCGTAGAAAAGATCAAAGGATCTTCTTGAGATCCTTTTTTTCT
GCGCGTAATCTGCTGCTTGCAAACAAAAAAACCACCGCTACCAGCGGTGGTTTGTTTGCCGGATCAAGAG
CTACCAACTCTTTTTCCGAAGGTAACTGGCTTCAGCAGAGCGCAGATACCAAATACTGTTCTTCTAGTGT
AGCCGTAGTTAGGCCACCACTTCAAGAACTCTGTAGCACCGCCTACATACCTCGCTCTGCTAATCCTGTT

ACCAGTGGCTGCTGCCAGTGGCGATAAGTCGTGTCTTACCGGGTTGGACTCAAGACGATAGTTACCGGAT
AAGGCGCAGCGGTCGGGCTGAACGGGGGGTTCGTGCACACAGCCCAGCTTGGAGCGAACGACCTACACCG
AACTGAGATACCTACAGCGTGAGCTATGAGAAAGCGCCACGCTTCCCGAAGGGAGAAAGGCGGACAGGTA
TCCGGTAAGCGGCAGGGTCGGAACAGGAGAGCGCACGAGGGAGCTTCCAGGGGGAAACGCCTGGTATCTT
TATAGTCCTGTCGGGTTTCGCCACCTCTGACTTGAGCGTCGATTTTTGTGATGCTCGTCAGGGGGGCGGA
GCCTATGGAAA

DNA template sequences

tCLuc

AAATAATTCTTACTGTCATGCCAAGTAAGATGCTTTTCTGTGCTGCAATAGCAGGCATGCTGGGGATGCG
GTGGGCTCTATGGCTTCTGAGGCGGAAAGAACCAGCTGGGGCTCTAGGGGGTATCCCCACGCGCCCTGTA
GCGGCGCATTAAGCGCGGCGGGTGTGGTGGTTACGCGCAGCGTGACCGCTACACTTGCCAGCGCCCTAGC
GCCCGCTCCTTTCGCTTTCTTCCCTTCCTTTCTCGCCACGTTCGCCGGCTTTCCCCGTCAAGCTCTAAAT
CGGGGCATCCCTTTAGGGTTCCGATTTAGTGCTTTACGGCACCTCGACCCCAAAAAACTTGATTAGGGTG
ATGGTTCACGTAGTGGGCCATCGCCCTGATAGACGGTTTTTCGCCCTTTGACGTTGGAGTCCACGTTCTT
TAATAGTGGACTCTTGTTCCAAACTGGAACAACACTCAACCCTATCTCGGTCTATTCTTTTGATTTATAA
GGGATTTTGGGGATTTCGGCCTATTGGTTAAAAAATGAGCTGATTTAACAAAAATTTAACGCGAATTAAT
TCTGTGGAATGTGTGTCAGTTAGGGTGTGGAAAGTCCCCAGGCTCCCCAGGCAGGCAGAAGTATGCAAAG
CATGCATCTCAATTAGTCAGCAACCAGGTGTGGAAAGTCCCCAGGCTCCCCAGCAGGCAGAAGTATGCAA
AGCATGCATCTCAATTAGTCAGCAACCATAGTCCCGCCCCTAACTCCGCCCATCCCGCCCCTAACTCCGC
CCAGTTCCGCCCATTCTCCGCCCCATGGCTGACTAATTTTTTTTATTTATGCAGAGGCCGAGGCCGCCTC
TGCCTCTGAGCTATTCCAGAAGTAGTGAGGAGGCTTTTTTGGAGGCCTAGGCTTTTGCAAAAAGCTCCCG
GGAGCTTGTATATCCATTTTCGGATCTGATCAAGAGACAGGATGAGGATCGTTTCGCATGATTGAACAAG
ATGGATTGCACGCAGGTTCTCCGGCCGCTTGGGTGGAGAGGCTATTCGGCTATGACTGGGCACAACAGAC
AATCGGCTGCTCTGATGCCGCCGTGTTCCGGCTGTCAGCGCAGGGGCGCCCGGTTCTTTTTGTCAAGACC
GACCTGTCCGGTGCCCTGAATGAACTGCAGGACGAGGCAGCGCGGCTATCGTGGCTGGCCACGACGGGCG
TTCCTTGCGCAGCTGTGCTCGACGTTGTCACTGAAGCGGGAAGGGACTGGCTGCTATTGGGCGAAGTGCC
GGGGCAGGATCTCCTGTCATCTCACCTTGCTCCTGCCGAGAAAGTATCCATCATGGCTGATGCAATGCGG
CGGCTGCATACGCTTGATCCGGCTACCTGCCCATTCGACCACCAAGCGAAACATCGCATCGAGCGAGCAC
GTACTCGGATGGAAGCCGGTCTTGTCGATCAGGATGATCTGGACGAAGAGCATCAGGGGCTCGCGCCAGC
CGAACTGTTCGCCAGGCTCAAGGCGCGCATGCCCGACGGCGAGGATCTCGTCGTGACCCATGGCGATGCC
TGCTTGCCGAATATCATGGTGGAAAATGGCCGCTTTTCTGGATTCATCGACTGTGGCCGGCTGGGTGTGG
CGGACCGCTATCAGGACATAGCGTTGGCTACCCGTGATATTGCTGAAGAGCTTGGCGGCGAATGGGCTGA
CCGCTTCCTCGTGCTTTACGGTATCGCCGCTCCCGATTCGCAGCGCATCGCCTTCTATCGCCTTCTTGAC
GAGTTCTTCTGAGCGGGACTCTGGGGTTCGAAATGACCGACCAAGCGACGCCCAACCTGCCATCACGAGA
TTTCGATTCCACCGCCGCCTTCTATGAAAGGTTGGGCTTCGGAATCGTTTTCCGGGACGCCGGCTGGATG
ATCCTCCAGCGCGGGGATCTCATGCTGGAGTTCTTCGCCCACCCCAACTTGTTTATTGCAGCTTATAATG
GTTACAAATAAAGCAATAGCATCACAAATTTCACAAATAAAGCATTTTTTTCACTGCATTCTAGTTGTGG
TTTGTCCAAACTCATCAATGTATCTTATCATGTCTGTATACCGTCGACCTCTAGCTAGAGCTTGGCGTAA
TCATGGTCATAGCTGTTTCCTGTGTGAAATTGTTATCCGCTCACAATTCCACACAACATACGAGCCGGAA
GCATAAAGTGTAAAGCCTGGGGTGCCTAATGAGTGAGCTAACTCACATTAATTGCGTTGCGCTCACTGCC
CGCTTTCCAGTCGGGAAACCTGTCGTGCCAGCTGCATTAATGAATCGGCCAACGCGCGGGGAGAGGCGGT
TTGCGTATTGGGCGCTCTTCCGCTTCCTCGCTCACTGACTCGCTGCGCTCGGTCGTTCGGCTGCGGCGAG
CGGTATCAGCTCACTCAAAGGCGGTAATACGGTTATCCACAGAATCAGGGGATAACGCAGGAAAGAACAT
GTGAGCAAAAGGCCAGCAAAAGGCCAGGAACCGTAAAAAGGCCGCGTTGCTGGCGTTTTTCCATAGGCTC
CGCCCCCCTGACGAGCATCACAAAAATCGACGCTCAAGTCAGAGGTGGCGAAACCCGACAGGACTATAAA
GATACCAGGCGTTTCCCCCTGGAAGCTCCCTCGTGCGCTCTCCTGTTCCGACCCTGCCGCTTACCGGATA

CCTGTCCGCCTTTCTCCCTTCGGGAAGCGTGGCGCTTTCTCAATGCTCACGCTGTAGGTATCTCAGTTCG
GTGTAGGTCGTTCGCTCCAAGCTGGGCTGTGTGCACGAACCCCCCGTTCAGCCCGACCGCTGCGCCTTAT
CCGGTAACTATCGTCTTGAGTCCAACCCGGTAAGACACGACTTATCGCCACTGGCAGCAGCCACTGGTAA
CAGGATTAGCAGAGCGAGGTATGTAGGCGGTGCTACAGAGTTCTTGAAGTGGTGGCCTAACTACGGCTAC
ACTAGAAGGACAGTATTTGGTATCTGCGCTCTGCTGAAGCCAGTTACCTTCGGAAAAAGAGTTGGTAGCT
CTTGATCCGGCAAACAAACCACCGCTGGTAGCGGTGGTTTTTTTGTTTGCAAGCAGCAGATTACGCGCAG
AAAAAAAGGATCTCAAGAAGATCCTTTGATCTTTTCTACGGGGTCTGACGCTCAGTGGAACGAAAACTCA
CGTTAAGGGATTTTGGTCATGAGATTATCAAAAAGGATCTTCACCTAGATCCTTTTAAATTAAAAATGAA
GTTTTAAATCAATCTAAAGTATATATGAGTAAACTTGGTCTGACAGTTACCAATGCTTAATCAGTGAGGC
ACCTATCTCAGCGATCTGTCTATTTCGTTCATCCATAGTTGCCTGACTCCCCGTCGTGTAGATAACTACG
ATACGGGAGGGCTTACCATCTGGCCCCAGTGCTGCAATGATACCGCGAGACCCACGCTCACCGGCTCCAG
ATTTATCAGCAATAAACCAGCCAGCCGGAAGGGCCGAGCGCAGAAGTGGTCCTGCAACTTTATCCGCCTC
CATCCAGTCTATTAATTGTTGCCGGGAAGCTAGAGTAAGTAGTTCGCCAGTTAATAGTTTGCGCAACGTT
GTTGCCATTGCTACAGGCATCGTGGTGTCACGCTCGTCGTTTGGTATGGCTTCATTCAGCTCCGGTTCCC
AACGATCAAGGCGAGTTACATGATCCCCCATGTTGTGCAAAAAAGCGGTTAGCTCCTTCGGTCCTCCGAT
CGTTGTCAGAAGTAAGTTGGCCGCAGTGTTATCACTCATGGTTATGGCAGCACTGCATAATTCTCTTACT
GTCATGCCATCCGTAAGATGCTTTTCTGTGACTGGTGAGTACTCAACCAAGTCATTCTGAGAATAGTGTA
TGCGGCGACCGAGTTGCTCTTGCCCGGCGTCAATACGGGATAATACCGCGCCACATAGCAGAACTTTAAA
AGTGCTCATCATTGGAAAACGTTCTTCGGGGCGAAAACTCTCAAGGATCTTACCGCTGTTGAGATCCAGT
TCGATGTAACCCACTCGTGCACCCAACTGATCTTCAGCATCTTTTACTTTCACCAGCGTTTCTGGGTGAG
CAAAAACAGGAAGGCAAAATGCCGCAAAAAAGGGAATAAGGGCGACACGGAAATGTTGAATACTCATACT
CTTCCTTTTTCAATATTATTGAAGCATTTATCAGGGTTATTGTCTCATGAGCGGATACATATTTGAATGT
ATTTAGAAAAATAAACAAATAGGGGTTCCGCGCACATTTCCCCGAAAAGTGCCACCTGACGTCGACGGAT
CGGGAGATCTCCCGATCCCCTATGGTCGACTCTCAGTACAATCTGCTCTGATGCCGCATAGTTAAGCCAG
TATCTGCTCCCTGCTTGTGTGTTGGAGGTCGCTGAGTAGTGCGCGAGCAAAATTTAAGCTACAACAAGGC
AAGGCTTGACCGACAATTGCATGAAGAATCTGCTTAGGGTTAGGCGTTTTGCGCTGCTTCGCGATGTACG
GGCCAGATATACGCGTTGACATTGATTATTGACTAGTTATTAATAGTAATCAATTACGGGGTCATTAGTT
CATAGCCCATATATGGAGTTCCGCGTTACATAACTTACGGTAAATGGCCCGCCTGGCTGACCGCCCAACG
ACCCCCGCCCATTGACGTCAATAATGACGTATGTTCCCATAGTAACGCCAATAGGGACTTTCCATTGACG
TCAATGGGTGGACTATTTACGGTAAACTGCCCACTTGGCAGTACATCAAGTGTATCATATGCCAAGTACG
CCCCCTATTGACGTCAATGACGGTAAATGGCCCGCCTGGCATTATGCCCAGTACATGACCTTATGGGACT
TTCCTACTTGGCAGTACATCTACGTATTAGTCATCGCTATTACCATGGTGATGCGGTTTTGGCAGTACAT
CAATGGGCGTGGATAGCGGTTTGACTCACGGGGATTTCCAAGTCTCCACCCCATTGACGTCAATGGGAGT
TTGTTTTGGCACCAAAATCAACGGGACTTTCCAAAATGTCGTAACAACTCCGCCCCATTGACGCAAATGG
GCGGTAGGCGTGTACGGTGGGAGGTCTATATAAGCAGAGCTCTCTGGCTAACTAGAGAACCCACTGCTTA
CTGGCTTATCGAAATTAATACGACTCACTATAAGGAGACCCAAGCTTGGTACCGAGCTCGGATCCGCCAC
CATGAAGACCTTAATTCTTGCCGTTGCATTAGTCTACTGCGCCACTGTTCATTGCCAGGACTGTCCTTAC
GAACCTGATCCACCAAACACAGTTCCAACTTCCTGTGAAGCTAAAGAAGGAGAATGTATTGATAGCAGCT
GTGGCACCTGCACGAGAGACATACTATCAGATGGACTGTGTGAAAATAAACCAGGAAAAACATGTTGCCG
AATGTGTCAGTATGTAATTGAATGCAGAGTAGAGGCCGCAGGATGGTTTAGAACATTCTATGGAAAGAGA
TTCCAGTTCCAGGAACCTGGTACATACGTGTTGGGTCAAGGAACCAAGGGCGGCGACTGGAAGGTGTCCA
TCACCCTGGAGAACCTGGATGGAACCAAGGGGGCTGTGCTGACCAAGACAAGACTGGAAGTGGCTGGAGA
CATCATTGACATCGCTCAAGCTACTGAGAATCCCATCACTGTAAACGGTGGAGCTGACCCTATCATCGCC
AACCCGTACACCATCGGCGAGGTCACCATCGCTGTTGTTGAGATGCCAGGCTTCAACATCACCGTCATTG
AGTTCTTCAAACTGATCGTGATCGACATCCTCGGAGGAAGATCTGTAAGAATCGCCCCAGACACAGCAAA
CAAAGGAATGATCTCTGGCCTCTGTGGAGATCTTAAAATGATGGAAGATACAGACTTCACTTCAGATCCA
GAACAACTCGCTATTCAGCCTAAGATCAACCAGGAGTTTGACGGTTGTCCACTCTATGGAAATCCTGATG

ACGTTGCATACTGCAAAGGTCTTCTGGAGCCGTACAAGGACAGCTGCCGCAACCCCATCAACTTCTACTA
CTACACCATCTCCTGCGCCTTCGCCCGCTGTATGGGTGGAGACGAGCGAGCCTCACACGTGCTGCTTGAC
TACAGGGAGACGTGCGCTGCTCCCGAAACTAGAGGAACCTGCGTTTTGTCTGGACATACTTTCTACGATA
CATTTGACAAAGCAAGATACCAATTCCAGGGTCCCTGCAAGGAGATTCTTATGGCCGCCGACTGTTTCTG
GAACACTTGGGATGTGAAGGTTTCACACAGGAATGTTGACTCTTACACTGAAGTAGAGAAAGTACGAATC
AGGAAACAATCGACTGTAGTAGAACTCATTGTTGATGGAAAACAGATTCTGGTTGGAGGAGAAGCCGTGT
CCGTCCCGTACAGCTCTCAGAACACTTCCATCTACTGGCAAGATGGTGACATACTGACTACAGCCATCCT
ACCTGAAGCTCTGGTGGTCAAGTTCAACTTCAAGCAACTGCTCGTCGTACATATTAGAGATCCATTCGAT
GGTAAGACTTGCGGTATTTGCGGTAACTACAACCAGGATTTCAGTGATGATTCTTTTGATGCTGAAGGAG
CCTGTGATCTGACCCCCAACCCACCGGGATGCACCGAAGAACAGAAACCTGAAGCTGAACGACTCTGCAA
TAGTCTCTTCGCCGGTCAAAGTGATCTTGATCAGAAATGTAACGTGTGCCACAAGCCTGACCGTGTCGAA
CGATGCATGTACGAGTATTGCCTGAGGGGACAACAGGGTTTCTGTGACCACGCATGGGAGTTCAAGAAAG
AATGCTACATAAAGCATGGAGACACCCTAGAAGTACCAGATGAATGCAAATAGGCGGCCGCAATAAAATA
TCTTTATTTTCATTACATCTGTGTGTTGGTTTTTTGTGTGTCTAG

tiEMCV-EGFP

GGAGACCAATAGAAACTGGGCTTGTCGAGACAGAGAAGACTCTTGCGTTTCTGATAGGCACCTATTGGTC
TTACTGACATCCACTTTGCCTTTCTCTCCACAGGTGTCCACTCCCAGTTCAATTACAGCTCTTAAGGCTA
GAGTCACCTGCAACATGACTTAATACGACTCACTATAGGAGAGATATCACGCGTTCTAGAGCTAGCCTC
GATAAGCTTGATATCGAATTCCGCCCCCCCCCCTCTCCCTCCCCCCCCCCTAACGTTACTGGCCGAAGC
CGCTTGGAATAAGGCCGGTGTGCGTTTGTCTATATGTTATTTTCCACCATATTGCCGTCTTTTGGCAATG
TGAGGGCCCGGAAACCTGGCCCTGTCTTCTTGACGAGCATTCCTAGGGGTCTTTCCCCTCTCGCCAAAGG
AATGCAAGGTCTGTTGAATGTCGTGAAGGAAGCAGTTCCTCTGGAAGCTTCTTGAAGACAAACAACGTCT
GTAGCGACCCTTTGCAGGCAGCGGAACCCCCCACCTGGCGACAGGTGCCTCTGCGGCCAAAAGCCACGTG
TATAAGATACACCTGCAAAGGCGGCACAACCCCAGTGCCACGTTGTGAGTTGGATAGTTGTGGAAAGAGT
CAAATGGCTCTCCTCAAGCGTATTCAACAAGGGGCTGAAGGATGCCCAGAAGGTACCCCATTGTATGGGA
TCTGATCTGGGGCCTCGGTGCACATGCTTTACATGTGTTTAGTCGAGGTTAAAAAAACGTCTAGGCCCCC
CGAACCACGGGGACGTGGTTTTCCTTTGAAAAACACGATGATAATATGACGATGATAATATGACTTCGAA
AGTTTATGATCCAGAACAAATGGTGAGCAAGGGCGAGGAGCTGTTCACCGGGGTGGTGCCCATCCTGGTC
GAGCTGGACGGCGACGTAAACGGCCACAAGTTCAGCGTGTCCGGCGAGGGCGAGGGCGATGCCACCTACG
GCAAGCTGACCCTGAAGTTCATCTGCACCACCGGCAAGCTGCCCGTGCCCTGGCCCACCCTCGTGACCAC
CCTGACCTACGGCGTGCAGTGCTTCAGCCGCTACCCCGACCACATGAAGCAGCACGACTTCTTCAAGTCC
GCCATGCCCGAAGGCTACGTCCAGGAGCGCACCATCTTCTTCAAGGACGACGGCAACTACAAGACCCGCG
CCGAGGTGAAGTTCGAGGGCGACACCCTGGTGAACCGCATCGAGCTGAAGGGCATCGACTTCAAGGAGGA
CGGCAACATCCTGGGGCACAAGCTGGAGTACAACTACAACAGCCACAACGTCTATATCATGGCCGACAAG
CAGAAGAACGGCATCAAGGTGAACTTCAAGATCCGCCACAACATCGAGGACGGCAGCGTGCAGCTCGCCG
ACCACTACCAGCAGAACACCCCCATCGGCGACGGCCCCGTGCTGCTGCCCGACAACCACTACCTGAGCAC
CCAGTCCGCCCTGAGCAAAGACCCCAACGAGAAGCGCGATCACATGGTCCTGCTGGAGTTCGTGACCGCC
GCCGGGATCACTCTCGGCATGGACGAGCTGTACAAGTAAAGCGGCCGCAGCTCGCTTTCTTGCTGTCCAA
TTTCTATTAAAGGTTCCTTTGTTCCCTAAGTCCAACTACTAAACTGGGGGATATTATGAAGGGCCTTGAG
CATTTGGATTCTGCCTAATAAAAAACATTTATTTTCATTGCGTTTAGCTCGCTTTCTTGCTGTCCAATTT
CTATTAAAGGTTCCTTTGTTCCCTAAGTCCAACTACTAAACTGGGGGATATTATGAAGGGCCTTGAGCAT
TTGGATTCTGCCTAATAAAAAACATTTATTTTCATTGCATTTAAATGTTTAAACAAAAAAAAAAAAAAAA
AAAAAAAAAAAAAAAAAAAAAAAAAAAAAAAAAAAAAAAAAAAAAAAAAAAAAAAAAAAAAAAAAAAAAA
AAAAAAAAAAAAAAAAAAAAAAAAAAAAAAAA

tiEMCV-EGFP (φ6.5), tiEMCV-EGFP (φ2.5)

φ6.5: X = –; φ2.5: X = T

ACTTAATACGACTCACTATXAGGAGAGATATCACGCGTTCTAGAGCTAGCCTCGATAAGCTTGATATCGA
ATTCCGCCCCCCCCCCCTCTCCCTCCCCCCCCCCTAACGTTACTGGCCGAAGCCGCTTGGAATAAGGCCG
GTGTGCGTTTGTCTATATGTTATTTTCCACCATATTGCCGTCTTTTGGCAATGTGAGGGCCCGGAAACCT
GGCCCTGTCTTCTTGACGAGCATTCCTAGGGGTCTTTCCCCTCTCGCCAAAGGAATGCAAGGTCTGTTGA
ATGTCGTGAAGGAAGCAGTTCCTCTGGAAGCTTCTTGAAGACAAACAACGTCTGTAGCGACCCTTTGCAG
GCAGCGGAACCCCCCACCTGGCGACAGGTGCCTCTGCGGCCAAAAGCCACGTGTATAAGATACACCTGCA
AAGGCGGCACAACCCCAGTGCCACGTTGTGAGTTGGATAGTTGTGGAAAGAGTCAAATGGCTCTCCTCAA
GCGTATTCAACAAGGGGCTGAAGGATGCCCAGAAGGTACCCCATTGTATGGGATCTGATCTGGGGCCTCG
GTGCACATGCTTTACATGTGTTTAGTCGAGGTTAAAAAAACGTCTAGGCCCCCCGAACCACGGGGACGTG
GTTTTCCTTTGAAAAACACGATGATAATATGACGATGATAATATGACTTCGAAAGTTTATGATCCAGAAC
AAATGGTGAGCAAGGGCGAGGAGCTGTTCACCGGGGTGGTGCCCATCCTGGTCGAGCTGGACGGCGACGT
AAACGGCCACAAGTTCAGCGTGTCCGGCGAGGGCGAGGGCGATGCCACCTACGGCAAGCTGACCCTGAAG
TTCATCTGCACCACCGGCAAGCTGCCCGTGCCCTGGCCCACCCTCGTGACCACCCTGACCTACGGCGTGC
AGTGCTTCAGCCGCTACCCCGACCACATGAAGCAGCACGACTTCTTCAAGTCCGCCATGCCCGAAGGCTA
CGTCCAGGAGCGCACCATCTTCTTCAAGGACGACGGCAACTACAAGACCCGCGCCGAGGTGAAGTTCGAG
GGCGACACCCTGGTGAACCGCATCGAGCTGAAGGGCATCGACTTCAAGGAGGACGGCAACATCCTGGGGC
ACAAGCTGGAGTACAACTACAACAGCCACAACGTCTATATCATGGCCGACAAGCAGAAGAACGGCATCAA
GGTGAACTTCAAGATCCGCCACAACATCGAGGACGGCAGCGTGCAGCTCGCCGACCACTACCAGCAGAAC
ACCCCCATCGGCGACGGCCCCGTGCTGCTGCCCGACAACCACTACCTGAGCACCCAGTCCGCCCTGAGCA
AAGACCCCAACGAGAAGCGCGATCACATGGTCCTGCTGGAGTTCGTGACCGCCGCCGGGATCACTCTCGG
CATGGACGAGCTGTACAAGTAAAGCGGCCGCAGCTCGCTTTCTTGCTGTCCAATTTCTATTAAAGGTTCC
TTTGTTCCCTAAGTCCAACTACTAAACTGGGGGATATTATGAAGGGCCTTGAGCATTTGGATTCTGCCTA
ATAAAAAACATTTATTTTCATTGCGTTTAGCTCGCTTTCTTGCTGTCCAATTTCTATTAAAGGTTCCTTT
GTTCCCTAAGTCCAACTACTAAACTGGGGGATATTATGAAGGGCCTTGAGCATTTGGATTCTGCCTAATA
AAAAACATTTATTTTCATTGCATTTAAATGTTTAAACAAAAAAAAAAAAAAAAAAAAAAAAAAAAAAAAA
AAAAAAAAAAAAAAAAAAAAAAAAAAAAAAAAAAAAAAAAAAAAAAAAAAAAAAAAAAAAAAAAAAAAAA
AAAAAAAAAAAAAAAAAA

tiEMCV-Cas9

GACTTAATACGACTCACTATAAGGAGAGATATCACGCGTCACAAATACCACTGAGATCCGCCCCTCTCCC
TCCCCCCCCCCTAACGTTACTGGCCGAAGCCGCTTGGAATAAGGCCGGTGTGCGTTTGTCTATATGTTAT
TTTCCACCATATTGCCGTCTTTTGGCAATGTGAGGGCCCGGAAACCTGGCCCTGTCTTCTTGACGAGCAT
TCCTAGGGGTCTTTCCCCTCTCGCCAAAGGAATGCAAGGTCTGTTGAATGTCGTGAAGGAAGCAGTTCCT
CTGGAAGCTTCTTGAAGACAAACAACGTCTGTAGCGACCCTTTGCAGGCAGCGGAACCCCCCACCTGGCG
ACAGGTGCCTCTGCGGCCAAAAGCCACGTGTATAAGATACACCTGCAAAGGCGGCACAACCCCAGTGCCA
CGTTGTGAGTTGGATAGTTGTGGAAAGAGTCAAATGGCTCTCCTCAAGCGTATTCAACAAGGGGCTGAAG
GATGCCCAGAAGGTACCCCATTGTATGGGATCTGATCTGGGGCCTCGGTACACATGCTTTACATGTGTTT
AGTCGAGGTTAAAAAAACGTCTAGGCCCCCCGAACCACGGGGACGTGGTTTTCCTTTGAAAAACACGATG
ATAATATGGCCACAACCATGGATTACAAGGATGACGACGATAAGATCATGGCCCCAAAGAAGAAGCGGAA
GGTCGGTATCCACGGAGTCCCAGCAGCCGACAAGAAGTACTCCATTGGGCTCGATATCGGCACAAACAGC
GTCGGCTGGGCCGTCATTACGGACGAGTACAAGGTGCCGAGCAAAAAATTCAAAGTTCTGGGCAATACCG
ATCGCCACAGCATAAAGAAGAACCTCATTGGCGCCCTCCTGTTCGACTCCGGGGAGACCGCCGAAGCCAC
GCGGCTCAAAAGAACAGCACGGCGCAGATATACCCGCAGAAAGAATCGGATCTGCTACCTGCAGGAGATC
TTTAGTAATGAGATGGCTAAGGTGGATGACTCTTTCTTCCATAGGCTGGAGGAGTCCTTTTTGGTGGAGG
AGGATAAAAAGCACGAGCGCCACCCAATCTTTGGCAATATCGTGGACGAGGTGGCGTACCATGAAAAGTA

CCCAACCATATATCATCTGAGGAAGAAGCTTGTAGACAGTACTGATAAGGCTGACTTGCGGTTGATCTAT
CTCGCGCTGGCGCATATGATCAAATTTCGGGGACACTTCCTCATCGAGGGGGACCTGAACCCAGACAACA
GCGATGTCGACAAACTCTTTATCCAACTGGTTCAGACTTACAATCAGCTTTTCGAAGAGAACCCGATCAA
CGCATCCGGAGTTGACGCCAAAGCAATCCTGAGCGCTAGGCTGTCCAAATCCCGGCGGCTCGAAAACCTC
ATCGCACAGCTCCCTGGGGAGAAGAAGAACGGCCTGTTTGGTAATCTTATCGCCCTGTCACTCGGGCTGA
CCCCCAACTTTAAATCTAACTTCGACCTGGCCGAAGATGCCAAGCTTCAACTGAGCAAAGACACCTACGA
TGATGATCTCGACAATCTGCTGGCCCAGATCGGCGACCAGTACGCAGACCTTTTTTTGGCGGCAAAGAAC
CTGTCAGACGCCATTCTGCTGAGTGATATTCTGCGAGTGAACACGGAGATCACCAAAGCTCCGCTGAGCG
CTAGTATGATCAAGCGCTATGATGAGCACCACCAAGACTTGACTTTGCTGAAGGCCCTTGTCAGACAGCA
ACTGCCTGAGAAGTACAAGGAAATTTTCTTCGATCAGTCTAAAAATGGCTACGCCGGATACATTGACGGC
GGAGCAAGCCAGGAGGAATTTTACAAATTTATTAAGCCCATCTTGGAAAAAATGGACGGCACCGAGGAGC
TGCTGGTAAAGCTTAACAGAGAAGATCTGTTGCGCAAACAGCGCACTTTCGACAATGGAAGCATCCCCCA
CCAGATTCACCTGGGCGAACTGCACGCTATCCTCAGGCGGCAAGAGGATTTCTACCCCTTTTTGAAAGAT
AACAGGGAAAAGATTGAGAAAATCCTCACATTTCGGATACCCTACTATGTAGGCCCCCTCGCCCGGGGAA
ATTCCAGATTCGCGTGGATGACTCGCAAATCAGAAGAGACCATCACTCCCTGGAACTTCGAGGAAGTCGT
GGATAAGGGGGCCTCTGCCCAGTCCTTCATCGAAAGGATGACTAACTTTGATAAAAATCTGCCTAACGAA
AAGGTGCTTCCTAAACACTCTCTGCTGTACGAGTACTTCACAGTTTATAACGAGCTCACCAAGGTCAAAT
ACGTCACAGAAGGGATGAGAAAGCCAGCATTCCTGTCTGGAGAGCAGAAGAAAGCTATCGTGGACCTCCT
CTTCAAGACGAACCGGAAAGTTACCGTGAAACAGCTCAAAGAAGACTATTTCAAAAAGATTGAATGTTTC
GACTCTGTTGAAATCAGCGGAGTGGAGGATCGCTTCAACGCATCCCTGGGAACGTATCACGATCTCCTGA
AAATCATTAAAGACAAGGACTTCCTGGACAATGAGGAGAACGAGGACATTCTTGAGGACATTGTCCTCAC
CCTTACGTTGTTTGAAGATAGGGAGATGATTGAAGAACGCTTGAAAACTTACGCTCATCTCTTCGACGAC
AAAGTCATGAAACAGCTCAAGAGGCGCCGATATACAGGATGGGGGCGGCTGTCAAGAAAACTGATCAATG
GGATCCGAGACAAGCAGAGTGGAAAGACAATCCTGGATTTTCTTAAGTCCGATGGATTTGCCAACCGGAA
CTTCATGCAGTTGATCCATGATGACTCTCTCACCTTTAAGGAGGACATCCAGAAAGCACAAGTTTCTGGC
CAGGGGGACAGTCTTCACGAGCACATCGCTAATCTTGCAGGTAGCCCAGCTATCAAAAAGGGAATACTGC
AGACCGTTAAGGTCGTGGATGAACTCGTCAAAGTAATGGGAAGGCATAAGCCCGAGAATATCGTTATCGA
GATGGCCCGAGAGAACCAAACTACCCAGAAGGGACAGAAGAACAGTAGGGAAAGGATGAAGAGGATTGAA
GAGGGTATAAAAGAACTGGGGTCCCAAATCCTTAAGGAACACCCAGTTGAAAACACCCAGCTTCAGAATG
AGAAGCTCTACCTGTACTACCTGCAGAACGGCAGGGACATGTACGTGGATCAGGAACTGGACATCAATCG
GCTCTCCGACTACGACGTGGATCATATCGTGCCCCAGTCTTTTCTCAAAGATGATTCTATTGATAATAAA
GTGTTGACAAGATCCGATAAAAATAGAGGGAAGAGTGATAACGTCCCCTCAGAAGAAGTTGTCAAGAAAA
TGAAAAATTATTGGCGGCAGCTGCTGAACGCCAAACTGATCACACAACGGAAGTTCGATAATCTGACTAA
GGCTGAACGAGGTGGCCTGTCTGAGTTGGATAAAGCCGGCTTCATCAAAAGGCAGCTTGTTGAGACACGC
CAGATCACCAAGCACGTGGCCCAAATTCTCGATTCACGCATGAACACCAAGTACGATGAAAATGACAAAC
TGATTCGAGAGGTGAAAGTTATTACTCTGAAGTCTAAGCTGGTCTCAGATTTCAGAAAGGACTTTCAGTT
TTATAAGGTGAGAGAGATCAACAATTACCACCATGCGCATGATGCCTACCTGAATGCAGTGGTAGGCACT
GCACTTATCAAAAAATATCCCAAGCTTGAATCTGAATTTGTTTACGGAGACTATAAAGTGTACGATGTTA
GGAAAATGATCGCAAAGTCTGAGCAGGAAATAGGCAAGGCCACCGCTAAGTACTTCTTTTACAGCAATAT
TATGAATTTTTTCAAGACCGAGATTACACTGGCCAATGGAGAGATTCGGAAGCGACCACTTATCGAAACA
AACGGAGAAACAGGAGAAATCGTGTGGGACAAGGGTAGGGATTTCGCGACAGTCCGGAAGGTCCTGTCCA
TGCCGCAGGTGAACATCGTTAAAAAGACCGAAGTACAGACCGGAGGCTTCTCCAAGGAAAGTATCCTCCC
GAAAAGGAACAGCGACAAGCTGATCGCACGCAAAAAAGATTGGGACCCCAAGAAATACGGCGGATTCGAT
TCTCCTACAGTCGCTTACAGTGTACTGGTTGTGGCCAAAGTGGAGAAAGGGAAGTCTAAAAAACTCAAAA
GCGTCAAGGAACTGCTGGGCATCACAATCATGGAGCGATCAAGCTTCGAAAAAAACCCCATCGACTTTCT
CGAGGCGAAAGGATATAAAGAGGTCAAAAAAGACCTCATCATTAAGCTTCCCAAGTACTCTCTCTTTGAG
CTTGAAAACGGCCGGAAACGAATGCTCGCTAGTGCGGGCGAGCTGCAGAAAGGTAACGAGCTGGCACTGC

CCTCTAAATACGTTAATTTCTTGTATCTGGCCAGCCACTATGAAAAGCTCAAAGGGTCTCCCGAAGATAA
TGAGCAGAAGCAGCTGTTCGTGGAACAACACAAACACTACCTTGATGAGATCATCGAGCAAATAAGCGAA
TTCTCCAAAAGAGTGATCCTCGCCGACGCTAACCTCGATAAGGTGCTTTCTGCTTACAATAAGCACAGGG
ATAAGCCCATCAGGGAGCAGGCAGAAAACATTATCCACTTGTTTACTCTGACCAACTTGGGCGCGCCTGC
AGCCTTCAAGTACTTCGACACCACCATAGACAGAAAGCGGTACACCTCTACAAAGGAGGTCCTGGACGCC
ACACTGATTCATCAGTCAATTACGGGGCTCTATGAAACAAGAATCGACCTCTCTCAGCTCGGTGGAGACA
GCAGGGCTGACCCCAAGAAGAAGAGGAAGGTGTGAATCGATGGTACAAGTAAGGATCTTTTTCCCTCTGC
CAAAAATTATGGGGACATCATGAAGCCCCTTGAGCATCTGACTTCTGGCTAATAAAGGAAATTTATTTTC
ATTGCAATAGTGTGTTGGAATTTTTTGTGTCTCACTCGGAAGGACATATGGAAAAAAAAAAAAAAAAAAA
AAAAAAAAAAAAAAAAAAAAAAAAAAAAAAAAAAAAAAAAAAAAAAAAAAAAAAAAAAAAAAAAAAAAAA
AAAAAAAAAAAAAAAAAAAAAAAAAAAAAAAA

tiHCV-EGFP

TTAATACGACTCACTATAAGGAGAGATATCACGCGTTCTAGAGCTAGCTAGTGTACGTAGCCAGCCCCCG
ATTGGGGGCGACACTCCACCATAGATCACTCCCCTGTGAGGAACTACTGTCTTCACGCAGAAAGCGTCTA
GCCATGGCGTTAGTATGAGAGTCGTGCAGCCTCCAGGACCCCCCCTCCCGGGAGAGCCATAGTGGTCTGC
GGAACCGGTGAGTACACCGGAATTGCCAGGACGACCGGGTCCTTTCTTGGATCAACCCGCTCAATGCCTG
GAGATTTGGGCGTGCCCCCGCAAGACTGCTAGCCGAGTAGTGTTGGGTCGCGAAAGGCCTTGTGGTACTG
CCTGATAGGGTGCTTGCGAGTGCCCCGGGAGGTCTCGTAGACCGTGCACCATGAGCACGAATCCTAAACC
TCAAAGAAAAACCATGGTGAGCAAGGGCGAGGAGCTGTTCACCGGGGTGGTGCCCATCCTGGTCGAGCTG
GACGGCGACGTAAACGGCCACAAGTTCAGCGTGTCCGGCGAGGGCGAGGGCGATGCCACCTACGGCAAGC
TGACCCTGAAGTTCATCTGCACCACCGGCAAGCTGCCCGTGCCCTGGCCCACCCTCGTGACCACCCTGAC
CTACGGCGTGCAGTGCTTCAGCCGCTACCCCGACCACATGAAGCAGCACGACTTCTTCAAGTCCGCCATG
CCCGAAGGCTACGTCCAGGAGCGCACCATCTTCTTCAAGGACGACGGCAACTACAAGACCCGCGCCGAGG
TGAAGTTCGAGGGCGACACCCTGGTGAACCGCATCGAGCTGAAGGGCATCGACTTCAAGGAGGACGGCAA
CATCCTGGGGCACAAGCTGGAGTACAACTACAACAGCCACAACGTCTATATCATGGCCGACAAGCAGAAG
AACGGCATCAAGGTGAACTTCAAGATCCGCCACAACATCGAGGACGGCAGCGTGCAGCTCGCCGACCACT
ACCAGCAGAACACCCCCATCGGCGACGGCCCCGTGCTGCTGCCCGACAACCACTACCTGAGCACCCAGTC
CGCCCTGAGCAAAGACCCCAACGAGAAGCGCGATCACATGGTCCTGCTGGAGTTCGTGACCGCCGCCGGG
ATCACTCTCGGCATGGACGAGCTGTACAAGTAAAGCGGCCGCAGCTCGCTTTCTTGCTGTCCAATTTCTA
TTAAAGGTTCCTTTGTTCCCTAAGTCCAACTACTAAACTGGGGGATATTATGAAGGGCCTTGAGCATTTG
GATTCTGCCTAATAAAAAACATTTATTTTCATTGCGTTTAGCTCGCTTTCTTGCTGTCCAATTTCTATTA
AAGGTTCCTTTGTTCCCTAAGTCCAACTACTAAACTGGGGGATATTATGAAGGGCCTTGAGCATTTGGAT
TCTGCCTAATAAAAAACATTTATTTTCATTGCATTTAAATGTTTAAACAAAAAAAAAAAAAAAAAAAAAA
AAAAAAAAAAAAAAAAAAAAAAAAAAAAAAAAAAAAAAAAAAAAAAAAAAAAAAAAAAAAAAAAAAAAAA
AAAAAAAAAAAAAAAAAAAAAAAAAAAA

tEGFP

TTAATACGACTCACTATAAGGAGAGATATCACGCGTTCTAGAGCTAGCGCTACCGGACTCAGATCTCGAG
CTCAAGCTTCGAATTCTGCAGTCGACGGTACCGCGGGCCCGGGATCCACCGGTCGCCACCATGGTGAGCA
AGGGCGAGGAGCTGTTCACCGGGGTGGTGCCCATCCTGGTCGAGCTGGACGGCGACGTAAACGGCCACAA
GTTCAGCGTGTCCGGCGAGGGCGAGGGCGATGCCACCTACGGCAAGCTGACCCTGAAGTTCATCTGCACC
ACCGGCAAGCTGCCCGTGCCCTGGCCCACCCTCGTGACCACCCTGACCTACGGCGTGCAGTGCTTCAGCC
GCTACCCCGACCACATGAAGCAGCACGACTTCTTCAAGTCCGCCATGCCCGAAGGCTACGTCCAGGAGCG
CACCATCTTCTTCAAGGACGACGGCAACTACAAGACCCGCGCCGAGGTGAAGTTCGAGGGCGACACCCTG
GTGAACCGCATCGAGCTGAAGGGCATCGACTTCAAGGAGGACGGCAACATCCTGGGGCACAAGCTGGAGT
ACAACTACAACAGCCACAACGTCTATATCATGGCCGACAAGCAGAAGAACGGCATCAAGGTGAACTTCAA
GATCCGCCACAACATCGAGGACGGCAGCGTGCAGCTCGCCGACCACTACCAGCAGAACACCCCCATCGGC

GACGGCCCCGTGCTGCTGCCCGACAACCACTACCTGAGCACCCAGTCCGCCCTGAGCAAAGACCCCAACG
AGAAGCGCGATCACATGGTCCTGCTGGAGTTCGTGACCGCCGCCGGGATCACTCTCGGCATGGACGAGCT
GTACAAGTAAAGCGGCCGCAGCTCGCTTTCTTGCTGTCCAATTTCTATTAAAGGTTCCTTTGTTCCCTAA
GTCCAACTACTAAACTGGGGGATATTATGAAGGGCCTTGAGCATTTGGATTCTGCCTAATAAAAAACATT
TATTTTCATTGCGTTTAGCTCGCTTTCTTGCTGTCCAATTTCTATTAAAGGTTCCTTTGTTCCCTAAGTC
CAACTACTAAACTGGGGGATATTATGAAGGGCCTTGAGCATTTGGATTCTGCCTAATAAAAAACATTTAT
TTTCATTGCATTTAAATGTTTAAACAAAAAAAAAAAAAAAAAAAAAAAAAAAAAAAAAAAAAAAAAAAAA
AAAAAAAAAAAAAAAAAAAAAAAAAAAAAAAAAAAAAAAAAAAAAAAAAAAAAAAAAAAAAAAAAAAAAA
AAAAA

tmCherry

TTAATACGACTCACTATAAGGAGAGATATCACGCGTTCTAGAGCTAGCGCTACCGGACTCAGATCTCGAG
CTCAAGCTTCGAATTCTGCAGTCGACGGTACCGCGGGCCCGGGATCCACCGGTCGCCACCATGGTGAGCA
AGGGCGAGGAGGATAACATGGCCATCATCAAGGAGTTCATGCGCTTCAAGGTGCACATGGAGGGCTCCGT
GAACGGCCACGAGTTCGAGATCGAGGGCGAGGGCGAGGGCCGCCCCTACGAGGGCACCCAGACCGCCAAG
CTGAAGGTGACCAAGGGTGGCCCCCTGCCCTTCGCCTGGGACATCCTGTCCCCTCAGTTCATGTACGGCT
CCAAGGCCTACGTGAAGCACCCCGCCGACATCCCCGACTACTTGAAGCTGTCCTTCCCCGAGGGCTTCAA
GTGGGAGCGCGTGATGAACTTCGAGGACGGCGGCGTGGTGACCGTGACCCAGGACTCCTCCCTGCAGGAC
GGCGAGTTCATCTACAAGGTGAAGCTGCGCGGCACCAACTTCCCCTCCGACGGCCCCGTAATGCAGAAGA
AGACCATGGGCTGGGAGGCCTCCTCCGAGCGGATGTACCCCGAGGACGGCGCCCTGAAGGGCGAGATCAA
GCAGAGGCTGAAGCTGAAGGACGGCGGCCACTACGACGCTGAGGTCAAGACCACCTACAAGGCCAAGAAG
CCCGTGCAGCTGCCCGGCGCCTACAACGTCAACATCAAGTTGGACATCACCTCCCACAACGAGGACTACA
CCATCGTGGAACAGTACGAACGCGCCGAGGGCCGCCACTCCACCGGCGGCATGGACGAGCTGTACAAGTA
AAGCGGCCGCAGCTCGCTTTCTTGCTGTCCAATTTCTATTAAAGGTTCCTTTGTTCCCTAAGTCCAACTA
CTAAACTGGGGGATATTATGAAGGGCCTTGAGCATTTGGATTCTGCCTAATAAAAAACATTTATTTTCAT
TGCGTTTAGCTCGCTTTCTTGCTGTCCAATTTCTATTAAAGGTTCCTTTGTTCCCTAAGTCCAACTACTA
AACTGGGGGATATTATGAAGGGCCTTGAGCATTTGGATTCTGCCTAATAAAAAACATTTATTTTCATTGC
ATTTAAATGTTTAAACAAAAAAAAAAAAAAAAAAAAAAAAAAAAAAAAAAAAAAAAAAAAAAAAAAAAAA
AAAAAAAAAAAAAAAAAAAAAAAAAAAAAAAAAAAAAAAAAAAAAAAAAAAAAAAAAAAAAAAAAA

tiSyn-EGFP

GACTTAATACGACTCACTATAAGGCGTCATATTCGGCGACCATTTGTGTGGTAAAAAAAAAAAACCAAAA
AAAAAAAACAAAAAAAAAAATAATTGACTAAGATATCTTAAAACAGCGGATGGGTACCCCACCATCCGA
CCCACTGGGTGTAGTACTCTGGTACTTCGTACCTTTGTACGCCTGTTCTTCCCATTGTACCCTTCCTGAA
CTTCCAACCCAAGTAACGTTAGAAGCTCAACATTTAGTACAACAGGAAGCACCACATCCAGTGGTGTTTA
GTACAAGCACTTCTGTTTCCCCGGAGCGAGGTATAGGCTGTACCCACTGCCAAAAACCTTTAACCGTTAT
CCGCCAACCAACTACGTAAAAGCTAGTAGTATTATGTTTTTAACTAGGCGTTCGATCAGGTGGATTTCCC
CTCCACTAGTTTGGTCGATGAGGCTAGGAATTCCCCACGGGTGACCGTGTCCTAGCCTGCGTGGCGGCCA
ACCCAGCCCACTCACTATTTGTTTTCGCGCCCAGTTGCAAAAAGTGTCGGGGCTGGACGCCTTTTTATA
GACATGGTGTGAAGACTCGCATGTGCTTGGTTGTGATTCCTCCGGCCCCTGAATGCGGCTAACCTTAACC
CTGGAGCCTTGTGTCACAAACCAGTGATGATAAGGTCGTAATGAGCAATTCCGGGACGGGACCGACTACT
TTGGGTGTCCGTGTTTCTTATTTTTCTTATTATTGTCTTATGGTCACAGCATATATATAACATATACTGT
GATCATGGTGAGCAAGGGCGAGGAGCTGTTCACCGGGGTGGTGCCCATCCTGGTCGAGCTGGACGGCGAC
GTAAACGGCCACAAGTTCAGCGTGTCCGGCGAGGGCGAGGGCGATGCCACCTACGGCAAGCTGACCCTGA
AGTTCATCTGCACCACCGGCAAGCTGCCCGTGCCCTGGCCCACCCTCGTGACCACCCTGACCTACGGCGT
GCAGTGCTTCAGCCGCTACCCCGACCACATGAAGCAGCACGACTTCTTCAAGTCCGCCATGCCCGAAGGC
TACGTCCAGGAGCGCACCATCTTCTTCAAGGACGACGGCAACTACAAGACCCGCGCCGAGGTGAAGTTCG
AGGGCGACACCCTGGTGAACCGCATCGAGCTGAAGGGCATCGACTTCAAGGAGGACGGCAACATCCTGGG

GCACAAGCTGGAGTACAACTACAACAGCCACAACGTCTATATCATGGCCGACAAGCAGAAGAACGGCATC
AAGGTGAACTTCAAGATCCGCCACAACATCGAGGACGGCAGCGTGCAGCTCGCCGACCACTACCAGCAGA
ACACCCCCATCGGCGACGGCCCCGTGCTGCTGCCCGACAACCACTACCTGAGCACCCAGTCCGCCCTGAG
CAAAGACCCCAACGAGAAGCGCGATCACATGGTCCTGCTGGAGTTCGTGACCGCCGCCGGGATCACTCTC
GGCATGGACGAGCTGTACAAGTAATAACAATTGGCTGGAGCCTCGGTGGCCATGCTTCTTGCCCCTTGGG
CCTCCCCCCAGCCCCTCCTCCCCTTCCTGCACCCGTACCCCCGTGGTCTTTGAATAAAGTCTGAACCACA
CAAATGGTCGCCGACTCAGTAGATGAAAAAAAAAAAAAAAAAAAAAAAAAAAAAAAAAAAAAAAAAAAAA
AAAAAAAAAAAAAAAAAAAAAAAAAAAAAAAAAAAAAAAAAAAAAAAAAAAAAAAAAAAAAAAAAAAAAA
AAAAA

tcirc-iSyn-EGFP

AAAAAAAAAAAAAAAAAAAAAAAAAAAGGCCAGTGAATTGTAATACGACTCACTATAGGGGTTAACAAAA
AAAAAAAAAAAAAAAAAAAATCACCGACCGATCTATCTGAACCGGTGGGAATTCTAGAGAAAATTTCGTC
TGGATTAGTTACTTATCGTGTAAAATCTGATAAATGGAATTGGTTCTACATAAATGCCTAACGACTATCC
CTTTGGGGAGTAGGGTCAAGTGACTCGAAACGATAGACAACTTGCTTTAACAAGTTGGAGATATAGTCTG
CTCTGCATGGTGACATGCAGCTGGATATAATTCCGGGGTAAGATTAACGACCTTATCTGAACATAACGCT
ACCGTTTAATATTGCGTCATATTCGGCGACCATTTGTGGTAAAAAAAAAAAAAACCAAAAAAAAAAAACA
AAAAAAAAAAATAATTGACTAAGATATCTTAAAACAGCGGATGGGTACCCCACCATCCGACCCACTGGGT
GTAGTACTCTGGTACTTCGTACCTTTGTACGCCTGTTCTTCCCATTGTACCCTTCCTGAACTTCCAACCC
AAGTAACGTTAGAAGCTCAACATTTAGTACAACAGGAAGCACCACATCCAGTGGTGTTTAGTACAAGCAC
TTCTGTTTCCCCGGAGCGAGGTATAGGCTGTACCCACTGCCAAAAACCTTTAACCGTTATCCGCCAACCA
ACTACGTAAAAGCTAGTAGTATTATGTTTTTAACTAGGCGTTCGATCAGGTGGATTTCCCCTCCACTAGT
TTGGTCGATGAGGCTAGGAATTCCCCACGGGTGACCGTGTCCTAGCCTGCGTGGCGGCCAACCCAGCCCA
CTCACTATTTGTTTTCGCGCCCAGTTGCAAAAAGTGTCGGGGCTGGGACGCCTTTTTATAGACATGGTGT
GAAGACTCGCATGTGCTTGGTTGTGATTCCTCCGGCCCCTGAATGCGGCTAACCTTAACCCTGGAGCCTT
GTGTCACAAACCAGTGATGATAAGGTCGTAATGAGCAATTCCGGGACGGGACCGACTACTTTGGGTGTCC
GTGTTTCTTATTTTTCTTATTATTGTCTTATGGTCACAGCATATATATAACATATACTGTGATCATGGTG
AGCAAGGGCGAGGAGCTGTTCACCGGGGTGGTGCCCATCCTGGTCGAGCTGGACGGCGACGTAAACGGCC
ACAAGTTCAGCGTGTCCGGCGAGGGCGAGGGCGATGCCACCTACGGCAAGCTGACCCTGAAGTTCATCTG
CACCACCGGCAAGCTGCCCGTGCCCTGGCCCACCCTCGTGACCACCCTGACCTACGGCGTGCAGTGCTTC
AGCCGCTACCCCGACCACATGAAGCAGCACGACTTCTTCAAGTCCGCCATGCCCGAAGGCTACGTCCAGG
AGCGCACCATCTTCTTCAAGGACGACGGCAACTACAAGACCCGCGCCGAGGTGAAGTTCGAGGGCGACAC
CCTGGTGAACCGCATCGAGCTGAAGGGCATCGACTTCAAGGAGGACGGCAACATCCTGGGGCACAAGCTG
GAGTACAACTACAACAGCCACAACGTCTATATCATGGCCGACAAGCAGAAGAACGGCATCAAGGTGAACT
TCAAGATCCGCCACAACATCGAGGACGGCAGCGTGCAGCTCGCCGACCACTACCAGCAGAACACCCCCAT
CGGCGACGGCCCCGTGCTGCTGCCCGACAACCACTACCTGAGCACCCAGTCCGCCCTGAGCAAAGACCCC
AACGAGAAGCGCGATCACATGGTCCTGCTGGAGTTCGTGACCGCCGCCGGGATCACTCTCGGCATGGACG
AGCTGTACAAGTAATAACAATTGGCTGGAGCCTCGGTGGCCATGCTTCTTGCCCCTTGGGCCTCCCCCCA
GCCCCTCCTCCCCTTCCTGCACCCGTACCCCCGTGGTCTTTGAATAAAGTCTGAACCACACAAATGGTCG
CCGACTCAGTAGATGTTTTCTTGGGTTAATTGAGGCCTGAGTATAAGGTGACTTATACTTGTAATCTATC
TAAACGGGGAACCTCTCTAGTAGACAATCCCGTGCTAAATTGTAGGACTGCCCTTTAATAAATACTTCTA
TATTTAAAGAGGTATTTATGAAAAGCGGAATTTATCAGATTAAAAATACTTTCTCTAGAGTCGACCTGCA
GACGCGTTCAGATAGATCGGTCGGTGAAAAAAAAAAAAAAAAAAAAAAAAAAGCGGCCGCCTAGCATAAC
CCCTTGGGGCCTCTAAACGGGTCTTGAGGGGTTTTTGAAAAAAAAAAAAAAAAAAAAAAAAAAAAAAA

mRNA sequences

CLuc-control

5′-triphosphate: X = pppAG; **5′-CleaN3:** X = N_3 ($m^{2'O}$A) G; **5′-CleanCap:** X = m^7Gppp ($m^{2'O}$A) G

XGAGACCCAAGCUUGGUACCGAGCUCGGAUCCGCCACCAUGAAGACCUUAAUUCUUGCCGUUGCAUUAGU
CUACUGCGCCACUGUUCAUUGCCAGGACUGUCCUUACGAACCUGAUCCACCAAACACAGUUCCAACUUCC
UGUGAAGCUAAAGAAGGAGAAUGUAUUGAUAGCAGCUGUGGCACCUGCACGAGAGACAUACUAUCAGAUG
GACUGUGUGAAAAUAAACCAGGAAAAACAUGUUGCCGAAUGUGUCAGUAUGUAAUUGAAUGCAGAGUAGA
GGCCGCAGGAUGGUUUAGAACAUUCUAUGGAAAGAGAUUCCAGUUCCAGGAACCUGGUACAUACGUGUUG
GGUCAAGGAACCAAGGGCGGCGACUGGAAGGUGUCCAUCACCCUGGAGAACCUGGAUGGAACCAAGGGGG
CUGUGCUGACCAAGACAAGACUGGAAGUGGCUGGAGACAUCAUUGACAUCGCUCAAGCUACUGAGAAUCC
CAUCACUGUAAACGGUGGAGCUGACCCUAUCAUCGCCAACCCGUACACCAUCGGCGAGGUCACCAUCGCU
GUUGUUGAGAUGCCAGGCUUCAACAUCACCGUCAUUGAGUUCUUCAAACUGAUCGUGAUCGACAUCCUCG
GAGGAAGAUCUGUAAGAAUCGCCCCAGACACAGCAAACAAAGGAAUGAUCUCUGGCCUCUGUGGAGAUCU
UAAAAUGAUGGAAGAUACAGACUUCACUUCAGAUCCAGAACAACUCGCUAUUCAGCCUAAGAUCAACCAG
GAGUUUGACGGUUGUCCACUCUAUGGAAAUCCUGAUGACGUUGCAUACUGCAAAGGUCUUCUGGAGCCGU
ACAAGGACAGCUGCCGCAACCCCAUCAACUUCUACUACUACACCAUCUCCUGCGCCUUCGCCCGCUGUAU
GGGUGGAGACGAGCGAGCCUCACACGUGCUGCUUGACUACAGGGAGACGUGCGCUGCUCCCGAAACUAGA
GGAACCUGCGUUUUGUCUGGACAUACUUUCUACGAUACAUUUGACAAAGCAAGAUACCAAUUCCAGGGUC
CCUGCAAGGAGAUUCUUAUGGCCGCCGACUGUUUCUGGAACACUUGGGAUGUGAAGGUUUCACACAGGAA
UGUUGACUCUUACACUGAAGUAGAGAAAGUACGAAUCAGGAAACAAUCGACUGUAGUAGAACUCAUUGUU
GAUGGAAAACAGAUUCUGGUUGGAGGAGAAGCCGUGUCCGUCCCGUACAGCUCUCAGAACACUUCCAUCU
ACUGGCAAGAUGGUGACAUACUGACUACAGCCAUCCUACCUGAAGCUCUGGUGGUCAAGUUCAACUUCAA
GCAACUGCUCGUCGUACAUAUUAGAGAUCCAUUCGAUGGUAAGACUUGCGGUAUUUGCGGUAACUACAAC
CAGGAUUUCAGUGAUGAUUCUUUUGAUGCUGAAGGAGCCUGUGAUCUGACCCCCAACCCACCGGGAUGCA
CCGAAGAACAGAAACCUGAAGCUGAACGACUCUGCAAUAGUCUCUUCGCCGGUCAAAGUGAUCUUGAUCA
GAAAUGUAACGUGUGCCACAAGCCUGACCGUGUCGAACGAUGCAUGUACGAGUAUUGCCUGAGGGGACAA
CAGGGUUUCUGUGACCACGCAUGGGAGUUCAAGAAAGAAUGCUACAUAAAGCAUGGAGACACCCUAGAAG
UACCAGAUGAAUGCAAAUAGGCGGCCGCAAUAAAAUAUCUUUAUUUUCAUUACAUCUGUGUGUUGGUUUU
UUGUGUGUCUAG

iEMCV-EGFP

5′-triphosphate: X = pppAG; **5′-CleaN3:** X = N_3($m^{2'O}$A)G; **5′-AF647:** X = AF647($m^{2'O}$A)G; **5′-CleanCap:** X = m^7Gppp ($m^{2'O}$A) G

XGAGAGAUAUCACGCGUUCUAGAGCUAGCCUCGAUAAGCUUGAUAUCGAAUUCCGCCCCCCCCCCCUCUC
CCUCCCCCCCCCCUAACGUUACUGGCCGAAGCCGCUUGGAAUAAGGCCGGUGUGCGUUUGUCUAUAUGUU
AUUUUCCACCAUAUUGCCGUCUUUUGGCAAUGUGAGGGCCCGGAAACCUGGCCCUGUCUUCUUGACGAGC
AUUCCUAGGGGUCUUUCCCCUCUCGCCAAAGGAAUGCAAGGUCUGUUGAAUGUCGUGAAGGAAGCAGUUC
CUCUGGAAGCUUCUUGAAGACAAACAACGUCUGUAGCGACCCUUUGCAGGCAGCGGAACCCCCCACCUGG
CGACAGGUGCCUCUGCGGCCAAAAGCCACGUGUAUAAGAUACACCUGCAAAGGCGGCACAACCCCAGUGC
CACGUUGUGAGUUGGAUAGUUGUGGAAAGAGUCAAAUGGCUCUCCUCAAGCGUAUUCAACAAGGGGCUGA
AGGAUGCCCAGAAGGUACCCCAUUGUAUGGGAUCUGAUCUGGGGCCUCGGUGCACAUGCUUUACAUGUGU
UUAGUCGAGGUUAAAAAAACGUCUAGGCCCCCCGAACCACGGGGACGUGGUUUUCCUUUGAAAAACACGA
UGAUAAUAUGACGAUGAUAAUAUGACUUCGAAAGUUUAUGAUCCAGAACAAAUGGUGAGCAAGGGCGAGG
AGCUGUUCACCGGGGUGGUGCCCAUCCUGGUCGAGCUGGACGGCGACGUAAACGGCCACAAGUUCAGCGU
GUCCGGCGAGGGCGAGGGCGAUGCCACCUACGGCAAGCUGACCCUGAAGUUCAUCUGCACCACCGGCAAG
CUGCCCGUGCCCUGGCCCACCCUCGUGACCACCCUGACCUACGGCGUGCAGUGCUUCAGCCGCUACCCCG

ACCACAUGAAGCAGCACGACUUCUUCAAGUCCGCCAUGCCCGAAGGCUACGUCCAGGAGCGCACCAUCUU
CUUCAAGGACGACGGCAACUACAAGACCCGCGCCGAGGUGAAGUUCGAGGGCGACACCCUGGUGAACCGC
AUCGAGCUGAAGGGCAUCGACUUCAAGGAGGACGGCAACAUCCUGGGGCACAAGCUGGAGUACAACUACA
ACAGCCACAACGUCUAUAUCAUGGCCGACAAGCAGAAGAACGGCAUCAAGGUGAACUUCAAGAUCCGCCA
CAACAUCGAGGACGGCAGCGUGCAGCUCGCCGACCACUACCAGCAGAACACCCCCAUCGGCGACGGCCCC
GUGCUGCUGCCCGACAACCACUACCUGAGCACCCAGUCCGCCCUGAGCAAAGACCCCAACGAGAAGCGCG
AUCACAUGGUCCUGCUGGAGUUCGUGACCGCCGCCGGGAUCACUCUCGGCAUGGACGAGCUGUACAAGUA
AAGCGGCCGCAGCUCGCUUUCUUGCUGUCCAAUUUCUAUUAAAGGUUCCUUUGUUCCCUAAGUCCAACUA
CUAAACUGGGGGAUAUUAUGAAGGGCCUUGAGCAUUUGGAUUCUGCCUAAUAAAAAACAUUUAUUUUCAU
UGCGUUUAGCUCGCUUUCUUGCUGUCCAAUUUCUAUUAAAGGUUCCUUUGUUCCCUAAGUCCAACUACUA
AACUGGGGGAUAUUAUGAAGGGCCUUGAGCAUUUGGAUUCUGCCUAAUAAAAAACAUUUAUUUUCAUUGC
AUUUAAAUGUUUAAACAAAAAAAAAAAAAAAAAAAAAAAAAAAAAAAAAAAAAAAAAAAAAAAAAAAAAA
AAAAAAAAAAAAAAAAAAAAAAAAAAAAAAAAAAAAAAAAAAAAAAAAAAAAAAAAAAAAAAAAAA

iEMCV-Cas9

5′-triphosphate: X = pppAG; **5′-CleaN3:** X = N_3 $(m^{2'O}A)$ G; **5′-CleanCap:** X = m^7Gppp $(m^{2'O}A)$ G

XGAGAGAUAUCACGCGUCACAAAUACCACUGAGAUCCGCCCCUCUCCCUCCCCCCCCCCUAACGUUACUG
GCCGAAGCCGCUUGGAAUAAGGCCGGUGUGCGUUUGUCUAUAUGUUAUUUUCCACCAUAUUGCCGUCUUU
UGGCAAUGUGAGGGCCCGGAAACCUGGCCCUGUCUUCUUGACGAGCAUUCCUAGGGGUCUUUCCCCUCUC
GCCAAAGGAAUGCAAGGUCUGUUGAAUGUCGUGAAGGAAGCAGUUCCUCUGGAAGCUUCUUGAAGACAAA
CAACGUCUGUAGCGACCCUUUGCAGGCAGCGGAACCCCCCACCUGGCGACAGGUGCCUCUGCGGCCAAAA
GCCACGUGUAUAAGAUACACCUGCAAAGGCGGCACAACCCCAGUGCCACGUUGUGAGUUGGAUAGUUGUG
GAAAGAGUCAAAUGGCUCUCCUCAAGCGUAUUCAACAAGGGGCUGAAGGAUGCCCAGAAGGUACCCCAUU
GUAUGGGAUCUGAUCUGGGGCCUCGGUACACAUGCUUUACAUGUGUUUAGUCGAGGUUAAAAAAACGUCU
AGGCCCCCCGAACCACGGGGACGUGGUUUUCCUUUGAAAAACACGAUGAUAAUAUGGCCACAACCAUGGA
UUACAAGGAUGACGACGAUAAGAUCAUGGCCCCAAAGAAGAAGCGGAAGGUCGGUAUCCACGGAGUCCCA
GCAGCCGACAAGAAGUACUCCAUUGGGCUCGAUAUCGGCACAAACAGCGUCGGCUGGGCCGUCAUUACGG
ACGAGUACAAGGUGCCGAGCAAAAAAUUCAAAGUUCUGGGCAAUACCGAUCGCCACAGCAUAAAGAAGAA
CCUCAUUGGCGCCCUCCUGUUCGACUCCGGGGAGACCGCCGAAGCCACGCGGCUCAAAAGAACAGCACGG
CGCAGAUAUACCCGCAGAAAGAAUCGGAUCUGCUACCUGCAGGAGAUCUUUAGUAAUGAGAUGGCUAAGG
UGGAUGACUCUUUCUUCCAUAGGCUGGAGGAGUCCUUUUUGGUGGAGGAGGAUAAAAAGCACGAGCGCCA
CCCAAUCUUUGGCAAUAUCGUGGACGAGGUGGCGUACCAUGAAAAGUACCCAACCAUAUAUCAUCUGAGG
AAGAAGCUUGUAGACAGUACUGAUAAGGCUGACUUGCGGUUGAUCUAUCUCGCGCUGGCGCAUAUGAUCA
AAUUUCGGGGACACUUCCUCAUCGAGGGGGACCUGAACCCAGACAACAGCGAUGUCGACAAACUCUUUAU
CCAACUGGUUCAGACUUACAAUCAGCUUUUCGAAGAGAACCCGAUCAACGCAUCCGGAGUUGACGCCAAA
GCAAUCCUGAGCGCUAGGCUGUCCAAAUCCCGGCGGCUCGAAAACCUCAUCGCACAGCUCCCUGGGGAGA
AGAAGAACGGCCUGUUUGGUAAUCUUAUCGCCCUGUCACUCGGGCUGACCCCCAACUUUAAAUCUAACUU
CGACCUGGCCGAAGAUGCCAAGCUUCAACUGAGCAAAGACACCUACGAUGAUGAUCUCGACAAUCUGCUG
GCCCAGAUCGGCGACCAGUACGCAGACCUUUUUUUGGCGGCAAAGAACCUGUCAGACGCCAUUCUGCUGA
GUGAUAUUCUGCGAGUGAACACGGAGAUCACCAAAGCUCCGCUGAGCGCUAGUAUGAUCAAGCGCUAUGA
UGAGCACCACCAAGACUUGACUUUGCUGAAGGCCCUUGUCAGACAGCAACUGCCUGAGAAGUACAAGGAA
AUUUUCUUCGAUCAGUCUAAAAAUGGCUACGCCGGAUACAUUGACGGCGGAGCAAGCCAGGAGGAAUUUU
ACAAAUUUAUUAAGCCCAUCUUGGAAAAAAUGGACGGCACCGAGGAGCUGCUGGUAAAGCUUAACAGAGA
AGAUCUGUUGCGCAAACAGCGCACUUUCGACAAUGGAAGCAUCCCCCACCAGAUUCACCUGGGCGAACUG
CACGCUAUCCUCAGGCGGCAAGAGGAUUUCUACCCCUUUUUGAAAGAUAACAGGGAAAAGAUUGAGAAAA
UCCUCACAUUUCGGAUACCCUACUAUGUAGGCCCCCUCGCCCGGGGAAAUUCCAGAUUCGCGUGGAUGAC
UCGCAAAUCAGAAGAGACCAUCACUCCCUGGAACUUCGAGGAAGUCGUGGAUAAGGGGGCCUCUGCCCAG

UCCUUCAUCGAAAGGAUGACUAACUUUGAUAAAAAUCUGCCUAACGAAAAGGUGCUUCCUAAACACUCUC
UGCUGUACGAGUACUUCACAGUUUAUAACGAGCUCACCAAGGUCAAAUACGUCACAGAAGGGAUGAGAAA
GCCAGCAUUCCUGUCUGGAGAGCAGAAGAAAGCUAUCGUGGACCUCCUCUUCAAGACGAACCGGAAAGUU
ACCGUGAAACAGCUCAAAGAAGACUAUUUCAAAAAGAUUGAAUGUUUCGACUCUGUUGAAAUCAGCGGAG
UGGAGGAUCGCUUCAACGCAUCCCUGGGAACGUAUCACGAUCUCCUGAAAAUCAUUAAAGACAAGGACUU
CCUGGACAAUGAGGAGAACGAGGACAUUCUUGAGGACAUUGUCCUCACCCUUACGUUGUUUGAAGAUAGG
GAGAUGAUUGAAGAACGCUUGAAAACUUACGCUCAUCUCUUCGACGACAAAGUCAUGAAACAGCUCAAGA
GGCGCCGAUAUACAGGAUGGGGGCGGCUGUCAAGAAAACUGAUCAAUGGGAUCCGAGACAAGCAGAGUGG
AAAGACAAUCCUGGAUUUUCUUAAGUCCGAUGGAUUUGCCAACCGGAACUUCAUGCAGUUGAUCCAUGAU
GACUCUCUCACCUUUAAGGAGGACAUCCAGAAAGCACAAGUUUCUGGCCAGGGGGACAGUCUUCACGAGC
ACAUCGCUAAUCUUGCAGGUAGCCCAGCUAUCAAAAAGGGAAUACUGCAGACCGUUAAGGUCGUGGAUGA
ACUCGUCAAAGUAAUGGGAAGGCAUAAGCCCGAGAAUAUCGUUAUCGAGAUGGCCCGAGAGAACCAAACU
ACCCAGAAGGGACAGAAGAACAGUAGGGAAAGGAUGAAGAGGAUUGAAGAGGGUAUAAAAGAACUGGGGU
CCCAAAUCCUUAAGGAACACCCAGUUGAAAACACCCAGCUUCAGAAUGAGAAGCUCUACCUGUACUACCU
GCAGAACGGCAGGGACAUGUACGUGGAUCAGGAACUGGACAUCAAUCGGCUCUCCGACUACGACGUGGAU
CAUAUCGUGCCCCAGUCUUUUCUCAAAGAUGAUUCUAUUGAUAAUAAAGUGUUGACAAGAUCCGAUAAAA
AUAGAGGGAAGAGUGAUAACGUCCCCUCAGAAGAAGUUGUCAAGAAAAUGAAAAAUUAUUGGCGGCAGCU
GCUGAACGCCAAACUGAUCACACAACGGAAGUUCGAUAAUCUGACUAAGGCUGAACGAGGUGGCCUGUCU
GAGUUGGAUAAAGCCGGCUUCAUCAAAAGGCAGCUUGUUGAGACACGCCAGAUCACCAAGCACGUGGCCC
AAAUUCUCGAUUCACGCAUGAACACCAAGUACGAUGAAAAUGACAAACUGAUUCGAGAGGUGAAAGUUAU
UACUCUGAAGUCUAAGCUGGUCUCAGAUUUCAGAAAGGACUUUCAGUUUUAUAAGGUGAGAGAGAUCAAC
AAUUACCACCAUGCGCAUGAUGCCUACCUGAAUGCAGUGGUAGGCACUGCACUUAUCAAAAAAUAUCCCA
AGCUUGAAUCUGAAUUUGUUUACGGAGACUAUAAAGUGUACGAUGUUAGGAAAAUGAUCGCAAAGUCUGA
GCAGGAAAUAGGCAAGGCCACCGCUAAGUACUUCUUUUACAGCAAUAUUAUGAAUUUUUUCAAGACCGAG
AUUACACUGGCCAAUGGAGAGAUUCGGAAGCGACCACUUAUCGAAACAAACGGAGAAACAGGAGAAAUCG
UGUGGGACAAGGGUAGGGAUUUCGCGACAGUCCGGAAGGUCCUGUCCAUGCCGCAGGUGAACAUCGUUAA
AAAGACCGAAGUACAGACCGGAGGCUUCUCCAAGGAAAGUAUCCUCCCGAAAAGGAACAGCGACAAGCUG
AUCGCACGCAAAAAAGAUUGGGACCCCAAGAAAUACGGCGGAUUCGAUUCUCCUACAGUCGCUUACAGUG
UACUGGUUGUGGCCAAAGUGGAGAAAGGGAAGUCUAAAAAACUCAAAAGCGUCAAGGAACUGCUGGGCAU
CACAAUCAUGGAGCGAUCAAGCUUCGAAAAAAACCCCAUCGACUUUCUCGAGGCGAAAGGAUAUAAAGAG
GUCAAAAAAGACCUCAUCAUUAAGCUUCCCAAGUACUCUCUCUUUGAGCUUGAAAACGGCCGGAAACGAA
UGCUCGCUAGUGCGGGCGAGCUGCAGAAAGGUAACGAGCUGGCACUGCCCUCUAAAUACGUUAAUUUCUU
GUAUCUGGCCAGCCACUAUGAAAAGCUCAAAGGGUCUCCCGAAGAUAAUGAGCAGAAGCAGCUGUUCGUG
GAACAACACAAACACUACCUUGAUGAGAUCAUCGAGCAAAUAAGCGAAUUCUCCAAAAGAGUGAUCCUCG
CCGACGCUAACCUCGAUAAGGUGCUUUCUGCUUACAAUAAGCACAGGGAUAAGCCCAUCAGGGAGCAGGC
AGAAAACAUUAUCCACUUGUUUACUCUGACCAACUUGGGCGCGCCUGCAGCCUUCAAGUACUUCGACACC
ACCAUAGACAGAAAGCGGUACACCUCUACAAAGGAGGUCCUGGACGCCACACUGAUUCAUCAGUCAAUUA
CGGGGCUCUAUGAAACAAGAAUCGACCUCUCUCAGCUCGGUGGAGACAGCAGGGCUGACCCCAAGAAGAA
GAGGAAGGUGUGAAUCGAUGGUACAAGUAAGGAUCUUUUUCCCUCUGCCAAAAAUUAUGGGGACAUCAUG
AAGCCCCUUGAGCAUCUGACUUCUGGCUAAUAAAGGAAAUUUAUUUUCAUUGCAAUAGUGUGUUGGAAUU
UUUUGUGUCUCUCACUCGGAAGGACAUAUGGAAAAAAAAAAAAAAAAAAAAAAAAAAAAAAAAAAAAAAA
AAAAAAAAAAAAAAAAAAAAAAAAAAAAAAAAAAAAAAAAAAAAAAAAAAAAAAAAAAAAAAAAAAAAAA
AAAAAAAAAAAA

iHCV-EGFP

5′-triphosphate: X = pppAG; **5′-CleaN3:** X = N_3 (m$^{2'O}$A) G; **5′-AF647:** X = AF647 (m$^{2'O}$A) G

XGAGAGAUAUCACGCGUUCUAGAGCUAGCUAGUGUACGUAGCCAGCCCCCGAUUGGGGGCGACACUCCAC
CAUAGAUCACUCCCCUGUGAGGAACUACUGUCUUCACGCAGAAAGCGUCUAGCCAUGGCGUUAGUAUGAG
AGUCGUGCAGCCUCCAGGACCCCCCCUCCCGGGAGAGCCAUAGUGGUCUGCGGAACCGGUGAGUACACCG
GAAUUGCCAGGACGACCGGGUCCUUUCUUGGAUCAACCCGCUCAAUGCCUGGAGAUUUGGGCGUGCCCCC
GCAAGACUGCUAGCCGAGUAGUGUUGGGUCGCGAAAGGCCUUGUGGUACUGCCUGAUAGGGUGCUUGCGA
GUGCCCCGGGAGGUCUCGUAGACCGUGCACCAUGAGCACGAAUCCUAAACCUCAAAGAAAAACCAUGGUG
AGCAAGGGCGAGGAGCUGUUCACCGGGGUGGUGCCCAUCCUGGUCGAGCUGGACGGCGACGUAAACGGCC
ACAAGUUCAGCGUGUCCGGCGAGGGCGAGGGCGAUGCCACCUACGGCAAGCUGACCCUGAAGUUCAUCUG
CACCACCGGCAAGCUGCCCGUGCCCUGGCCCACCCUCGUGACCACCCUGACCUACGGCGUGCAGUGCUUC
AGCCGCUACCCCGACCACAUGAAGCAGCACGACUUCUUCAAGUCCGCCAUGCCCGAAGGCUACGUCCAGG
AGCGCACCAUCUUCUUCAAGGACGACGGCAACUACAAGACCCGCGCCGAGGUGAAGUUCGAGGGCGACAC
CCUGGUGAACCGCAUCGAGCUGAAGGGCAUCGACUUCAAGGAGGACGGCAACAUCCUGGGGCACAAGCUG
GAGUACAACUACAACAGCCACAACGUCUAUAUCAUGGCCGACAAGCAGAAGAACGGCAUCAAGGUGAACU
UCAAGAUCCGCCACAACAUCGAGGACGGCAGCGUGCAGCUCGCCGACCACUACCAGCAGAACACCCCCAU
CGGCGACGGCCCCGUGCUGCUGCCCGACAACCACUACCUGAGCACCCAGUCCGCCCUGAGCAAAGACCCC
AACGAGAAGCGCGAUCACAUGGUCCUGCUGGAGUUCGUGACCGCCGCCGGGAUCACUCUCGGCAUGGACG
AGCUGUACAAGUAAAGCGGCCGCAGCUCGCUUUCUUGCUGUCCAAUUUCUAUUAAAGGUUCCUUUGUUCC
CUAAGUCCAACUACUAAACUGGGGGAUAUUAUGAAGGGCCUUGAGCAUUUGGAUUCUGCCUAAUAAAAAA
CAUUUAUUUUCAUUGCGUUUAGCUCGCUUUCUUGCUGUCCAAUUUCUAUUAAAGGUUCCUUUGUUCCCUA
AGUCCAACUACUAAACUGGGGGAUAUUAUGAAGGGCCUUGAGCAUUUGGAUUCUGCCUAAUAAAAAACAU
UUAUUUUCAUUGCAUUUAAAUGUUUAAACAAAAAAAAAAAAAAAAAAAAAAAAAAAAAAAAAAAAAAAAA
AAAAAAAAAAAAAAAAAAAAAAAAAAAAAAAAAAAAAAAAAAAAAAAAAAAAAAAAAAAAAAAAAAAAAA
AAAAAAAAA

EGFP

m^{7}Gppp (m$^{2'O}$A) GGAGAGAUAUCACGCGUUCUAGAGCUAGCGCUACCGGACUCAGAUCUCGAGCUCAAGCU
UCGAAUUCUGCAGUCGACGGUACCGCGGGCCCGGGAUCCACCGGUCGCCACCAUGGUGAGCAAGGGCGAG
GAGCUGUUCACCGGGGUGGUGCCCAUCCUGGUCGAGCUGGACGGCGACGUAAACGGCCACAAGUUCAGCG
UGUCCGGCGAGGGCGAGGGCGAUGCCACCUACGGCAAGCUGACCCUGAAGUUCAUCUGCACCACCGGCAA
GCUGCCCGUGCCCUGGCCCACCCUCGUGACCACCCUGACCUACGGCGUGCAGUGCUUCAGCCGCUACCCC
GACCACAUGAAGCAGCACGACUUCUUCAAGUCCGCCAUGCCCGAAGGCUACGUCCAGGAGCGCACCAUCU
UCUUCAAGGACGACGGCAACUACAAGACCCGCGCCGAGGUGAAGUUCGAGGGCGACACCCUGGUGAACCG
CAUCGAGCUGAAGGGCAUCGACUUCAAGGAGGACGGCAACAUCCUGGGGCACAAGCUGGAGUACAACUAC
AACAGCCACAACGUCUAUAUCAUGGCCGACAAGCAGAAGAACGGCAUCAAGGUGAACUUCAAGAUCCGCC
ACAACAUCGAGGACGGCAGCGUGCAGCUCGCCGACCACUACCAGCAGAACACCCCCAUCGGCGACGGCCC
CGUGCUGCUGCCCGACAACCACUACCUGAGCACCCAGUCCGCCCUGAGCAAAGACCCCAACGAGAAGCGC
GAUCACAUGGUCCUGCUGGAGUUCGUGACCGCCGCCGGGAUCACUCUCGGCAUGGACGAGCUGUACAAGU
AAAGCGGCCGCAGCUCGCUUUCUUGCUGUCCAAUUUCUAUUAAAGGUUCCUUUGUUCCCUAAGUCCAACU
ACUAAACUGGGGGAUAUUAUGAAGGGCCUUGAGCAUUUGGAUUCUGCCUAAUAAAAAACAUUUAUUUUCA
UUGCGUUUAGCUCGCUUUCUUGCUGUCCAAUUUCUAUUAAAGGUUCCUUUGUUCCCUAAGUCCAACUACU
AAACUGGGGGAUAUUAUGAAGGGCCUUGAGCAUUUGGAUUCUGCCUAAUAAAAAACAUUUAUUUUCAUUG
CAUUUAAAUGUUUAAACAAAAAAAAAAAAAAAAAAAAAAAAAAAAAAAAAAAAAAAAAAAAAAAAAAAAA
AAAAAAAAAAAAAAAAAAAAAAAAAAAAAAAAAAAAAAAAAAAAAAAAAAAAAAAAAAAAAAAAAA

mCherry

m^{7}Gppp (m$^{2'O}$A) GGAGAGAUAUCACGCGUUCUAGAGCUAGCGCUACCGGACUCAGAUCUCGAGCUCAAGCU
UCGAAUUCUGCAGUCGACGGUACCGCGGGCCCGGGAUCCACCGGUCGCCACCAUGGUGAGCAAGGGCGAG
GAGGAUAACAUGGCCAUCAUCAAGGAGUUCAUGCGCUUCAAGGUGCACAUGGAGGGCUCCGUGAACGGCC
ACGAGUUCGAGAUCGAGGGCGAGGGCGAGGGCCGCCCCUACGAGGGCACCCAGACCGCCAAGCUGAAGGU
GACCAAGGGUGGCCCCCUGCCCUUCGCCUGGGACAUCCUGUCCCCUCAGUUCAUGUACGGCUCCAAGGCC
UACGUGAAGCACCCCGCCGACAUCCCCGACUACUUGAAGCUGUCCUUCCCCGAGGGCUUCAAGUGGGAGC
GCGUGAUGAACUUCGAGGACGGCGGCGUGGUGACCGUGACCCAGGACUCCUCCCUGCAGGACGGCGAGUU
CAUCUACAAGGUGAAGCUGCGCGGCACCAACUUCCCCUCCGACGGCCCCGUAAUGCAGAAGAAGACCAUG
GGCUGGGAGGCCUCCUCCGAGCGGAUGUACCCCGAGGACGGCGCCCUGAAGGGCGAGAUCAAGCAGAGGC
UGAAGCUGAAGGACGGCGGCCACUACGACGCUGAGGUCAAGACCACCUACAAGGCCAAGAAGCCCGUGCA
GCUGCCCGGCGCCUACAACGUCAACAUCAAGUUGGACAUCACCUCCCACAACGAGGACUACACCAUCGUG
GAACAGUACGAACGCGCCGAGGGCCGCCACUCCACCGGCGGCAUGGACGAGCUGUACAAGUAAAGCGGCC
GCAGCUCGCUUUCUUGCUGUCCAAUUUCUAUUAAAGGUUCCUUUGUUCCCUAAGUCCAACUACUAAACUG
GGGGAUAUUAUGAAGGGCCUUGAGCAUUUGGAUUCUGCCUAAUAAAAAACAUUUAUUUUCAUUGCGUUUA
GCUCGCUUUCUUGCUGUCCAAUUUCUAUUAAAGGUUCCUUUGUUCCCUAAGUCCAACUACUAAACUGGGG
GAUAUUAUGAAGGGCCUUGAGCAUUUGGAUUCUGCCUAAUAAAAAACAUUUAUUUUCAUUGCAUUUAAAU
GUUUAAACAAAAAAAAAAAAAAAAAAAAAAAAAAAAAAAAAAAAAAAAAAAAAAAAAAAAAAAAAA
AAAAAAAAAAAAAAAAAAAAAAAAAAAAAAAAAAAAAAAAAAAAAAAAAAAAAAAAAA

iSyn-EGFP

AF647 (m$^{2'O}$A) GGCGUCAUAUUCGGCGACCAUUUGUGUGGUAAAAAAAAAAAACCAAAAAAAAAAAACAA
AAAAAAAAAAUAAUUGACUAAGAUAUCUUAAAACAGCGGAUGGGUACCCCACCAUCCGACCCACUGGGUG
UAGUACUCUGGUACUUCGUACCUUUGUACGCCUGUUCUUCCCAUUGUACCCUUCCUGAACUUCCAACCCA
AGUAACGUUAGAAGCUCAACAUUUAGUACAACAGGAAGCACCACAUCCAGUGGUGUUUAGUACAAGCACU
UCUGUUUCCCCGGAGCGAGGUAUAGGCUGUACCCACUGCCAAAAACCUUUAACCGUUAUCCGCCAACCAA
CUACGUAAAAGCUAGUAGUAUUAUGUUUUUAACUAGGCGUUCGAUCAGGUGGAUUUCCCCUCCACUAGUU
UGGUCGAUGAGGCUAGGAAUUCCCCACGGGUGACCGUGUCCUAGCCUGCGUGGCGGCCAACCCAGCCCAC
UCACUAUUUGUUUUCGCGCCCAGUUGCAAAAAGUGUCGGGGCUGGGACGCCUUUUUAUAGACAUGGUGUG
AAGACUCGCAUGUGCUUGGUUGUGAUUCCUCCGGCCCCUGAAUGCGGCUAACCUUAACCCUGGAGCCUUG
UGUCACAAACCAGUGAUGAUAAGGUCGUAAUGAGCAAUUCCGGGACGGGACCGACUACUUUGGGUGUCCG
UGUUUCUUAUUUUUCUUAUUAUUGUCUUAUGGUCACAGCAUAUAUAUAACAUAUACUGUGAUCAUGGUGA
GCAAGGGCGAGGAGCUGUUCACCGGGGUGGUGCCCAUCCUGGUCGAGCUGGACGGCGACGUAAACGGCCA
CAAGUUCAGCGUGUCCGGCGAGGGCGAGGGCGAUGCCACCUACGGCAAGCUGACCCUGAAGUUCAUCUGC
ACCACCGGCAAGCUGCCCGUGCCCUGGCCCACCCUCGUGACCACCCUGACCUACGGCGUGCAGUGCUUCA
GCCGCUACCCCGACCACAUGAAGCAGCACGACUUCUUCAAGUCCGCCAUGCCCGAAGGCUACGUCCAGGA
GCGCACCAUCUUCUUCAAGGACGACGGCAACUACAAGACCCGCGCCGAGGUGAAGUUCGAGGGCGACACC
CUGGUGAACCGCAUCGAGCUGAAGGGCAUCGACUUCAAGGAGGACGGCAACAUCCUGGGGCACAAGCUGG
AGUACAACUACAACAGCCACAACGUCUAUAUCAUGGCCGACAAGCAGAAGAACGGCAUCAAGGUGAACUU
CAAGAUCCGCCACAACAUCGAGGACGGCAGCGUGCAGCUCGCCGACCACUACCAGCAGAACACCCCCAUC
GGCGACGGCCCCGUGCUGCUGCCCGACAACCACUACCUGAGCACCCAGUCCGCCCUGAGCAAAGACCCCA
ACGAGAAGCGCGAUCACAUGGUCCUGCUGGAGUUCGUGACCGCCGCCGGGAUCACUCUCGGCAUGGACGA
GCUGUACAAGUAAUAACAAUUGGCUGGAGCCUCGGUGGCCAUGCUUCUUGCCCCUUGGGCCUCCCCCCAG
CCCCUCCUCCCCUUCCUGCACCCGUACCCCCGUGGUCUUUGAAUAAAGUCUGAACCACACAAAUGGUCGC
CGACUCAGUAGAUGAAAAAAAAAAAAAAAAAAAAAAAAAAAAAAAAAAAAAAAAAAAAAAAAAA
AAAAAAAAAAAAAAAAAAAAAAAAAAAAAAAAAAAAAAAAAAAAAAAAAAAAAAAAAAAAAAAA

circ-iSyn-EGFP

CUACCGUUUAAUAUUGCGUCAUAUUCGGCGACCAUUUGUGUGGUAAAAAAAAAAAACCAAAAAAAAAAAA
CAAAAAAAAAAAAUAAUUGACUAAGAUAUCUUAAAACAGCGGAUGGGUACCCCACCAUCCGACCCACUGG
GUGUAGUACUCUGGUACUUCGUACCUUUGUACGCCUGUUCUUCCCAUUGUACCCUUCCUGAACUUCCAAC
CCAAGUAACGUUAGAAGCUCAACAUUUAGUACAACAGGAAGCACCACAUCCAGUGGUGUUUAGUACAAGC
ACUUCUGUUUCCCCGGAGCGAGGUAUAGGCUGUACCCACUGCCAAAAACCUUUAACCGUUAUCCGCCAAC
CAACUACGUAAAAGCUAGUAGUAUUAUGUUUUUAACUAGGCGUUCGAUCAGGUGGAUUUCCCCUCCACUA
GUUUGGUCGAUGAGGCUAGGAAUUCCCCACGGGUGACCGUGUCCUAGCCUGCGUGGCGGCCAACCCAGCC
CACUCACUAUUUGUUUUCGCGCCCAGUUGCAAAAAGUGUCGGGGCUGGGACGCCUUUUUAUAGACAUGGU
GUGAAGACUCGCAUGUGCUUGGUUGUGAUUCCUCCGGCCCCUGAAUGCGGCUAACCUUAACCCUGGAGCC
UUGUGUCACAAACCAGUGAUGAUAAGGUCGUAAUGAGCAAUUCCGGGACGGGACCGACUACUUUGGGUGU
CCGUGUUUCUUAUUUUUCUUAUUAUUGUCUUAUGGUCACAGCAUAUAUAUAACAUAUACUGUGAUCAUGG
UGAGCAAGGGCGAGGAGCUGUUCACCGGGGUGGUGCCCAUCCUGGUCGAGCUGGACGGCGACGUAAACGG
CCACAAGUUCAGCGUGUCCGGCGAGGGCGAGGGCGAUGCCACCUACGGCAAGCUGACCCUGAAGUUCAUC
UGCACCACCGGCAAGCUGCCCGUGCCCUGGCCCACCCUCGUGACCACCCUGACCUACGGCGUGCAGUGCU
UCAGCCGCUACCCCGACCACAUGAAGCAGCACGACUUCUUCAAGUCCGCCAUGCCCGAAGGCUACGUCCA
GGAGCGCACCAUCUUCUUCAAGGACGACGGCAACUACAAGACCCGCGCCGAGGUGAAGUUCGAGGGCGAC
ACCCUGGUGAACCGCAUCGAGCUGAAGGGCAUCGACUUCAAGGAGGACGGCAACAUCCUGGGGCACAAGC
UGGAGUACAACUACAACAGCCACAACGUCUAUAUCAUGGCCGACAAGCAGAAGAACGGCAUCAAGGUGAA
CUUCAAGAUCCGCCACAACAUCGAGGACGGCAGCGUGCAGCUCGCCGACCACUACCAGCAGAACACCCCC
AUCGGCGACGGCCCCGUGCUGCUGCCCGACAACCACUACCUGAGCACCCAGUCCGCCCUGAGCAAAGACC
CCAACGAGAAGCGCGAUCACAUGGUCCUGCUGGAGUUCGUGACCGCCGCCGGGAUCACUCUCGGCAUGGA
CGAGCUGUACAAGUAAUAACAAUUGGCUGGAGCCUCGGUGGCCAUGCUUCUUGCCCCUUGGGCCUCCCCC
CAGCCCCUCCUCCCCUUCCUGCACCCGUACCCCCGUGGUCUUUGAAUAAAGUCUGAACCACACAAAUGGU
CGCCGACUCAGUAGAUGUUUUCUUGGGU

NMR and MS spectra

Structure

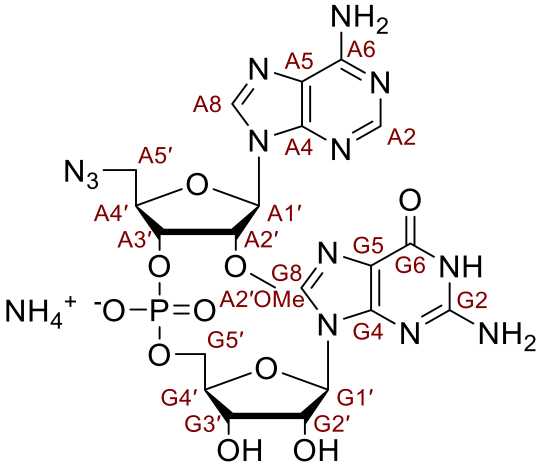

HRMS spectrum

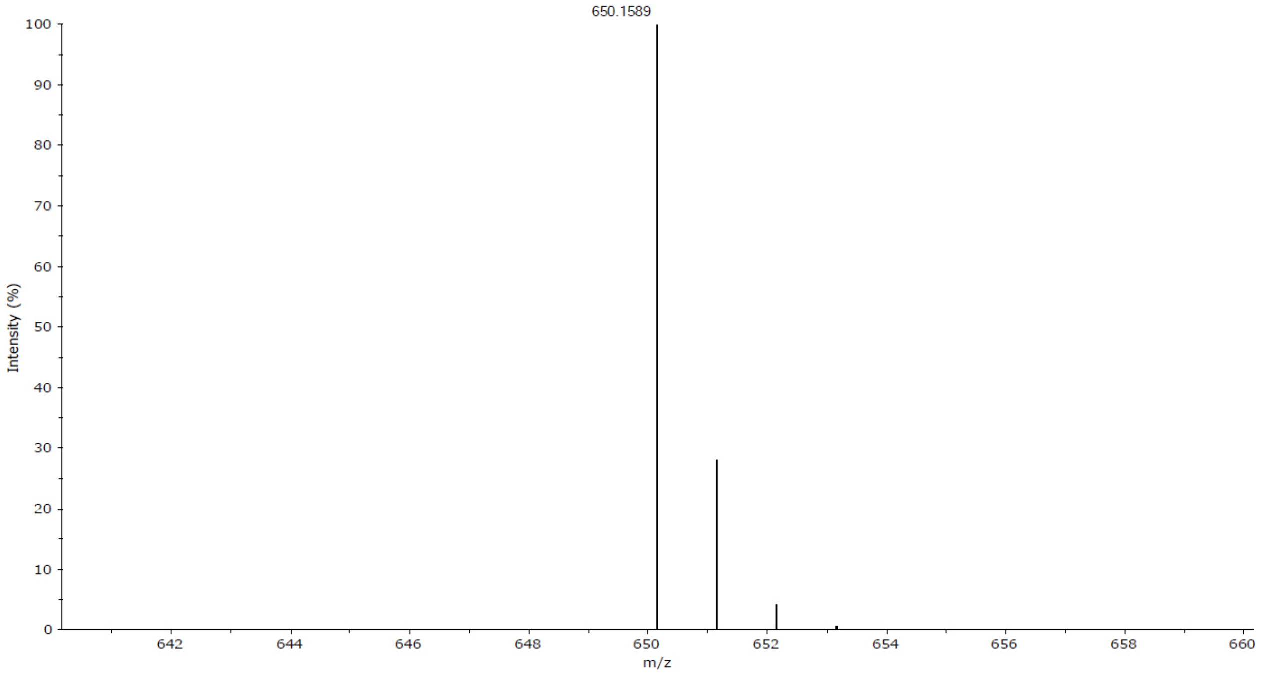

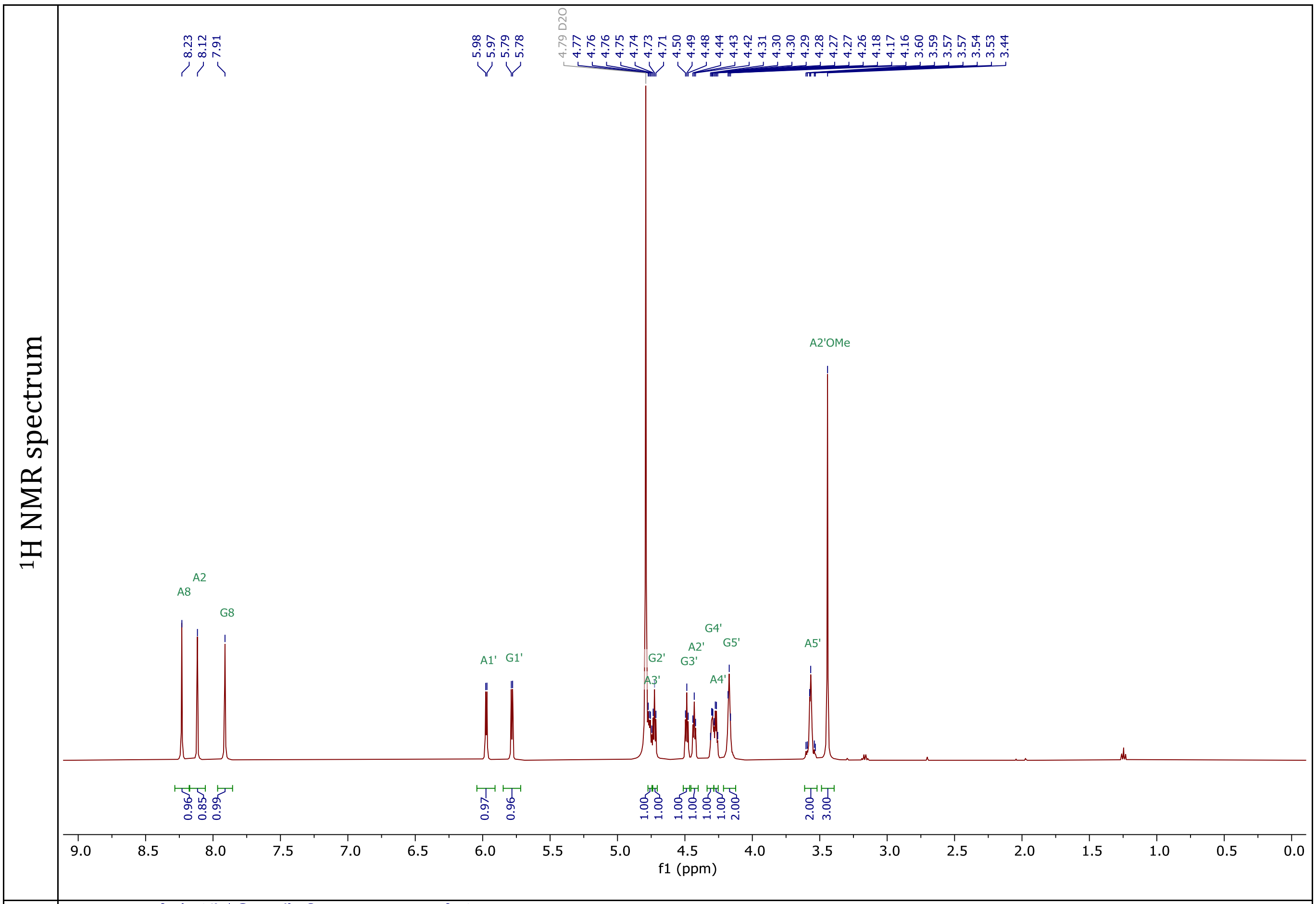
1H NMR spectrum
8.23
8.12
7.91
5.98
5.97
5.79
5.78
4.79 D2O
A8
A2
G8
A1'
G1'
G2'
A3'
G3'
A2'
G4'
G5'
A4'
A5'
A2'OMe
f1 (ppm)

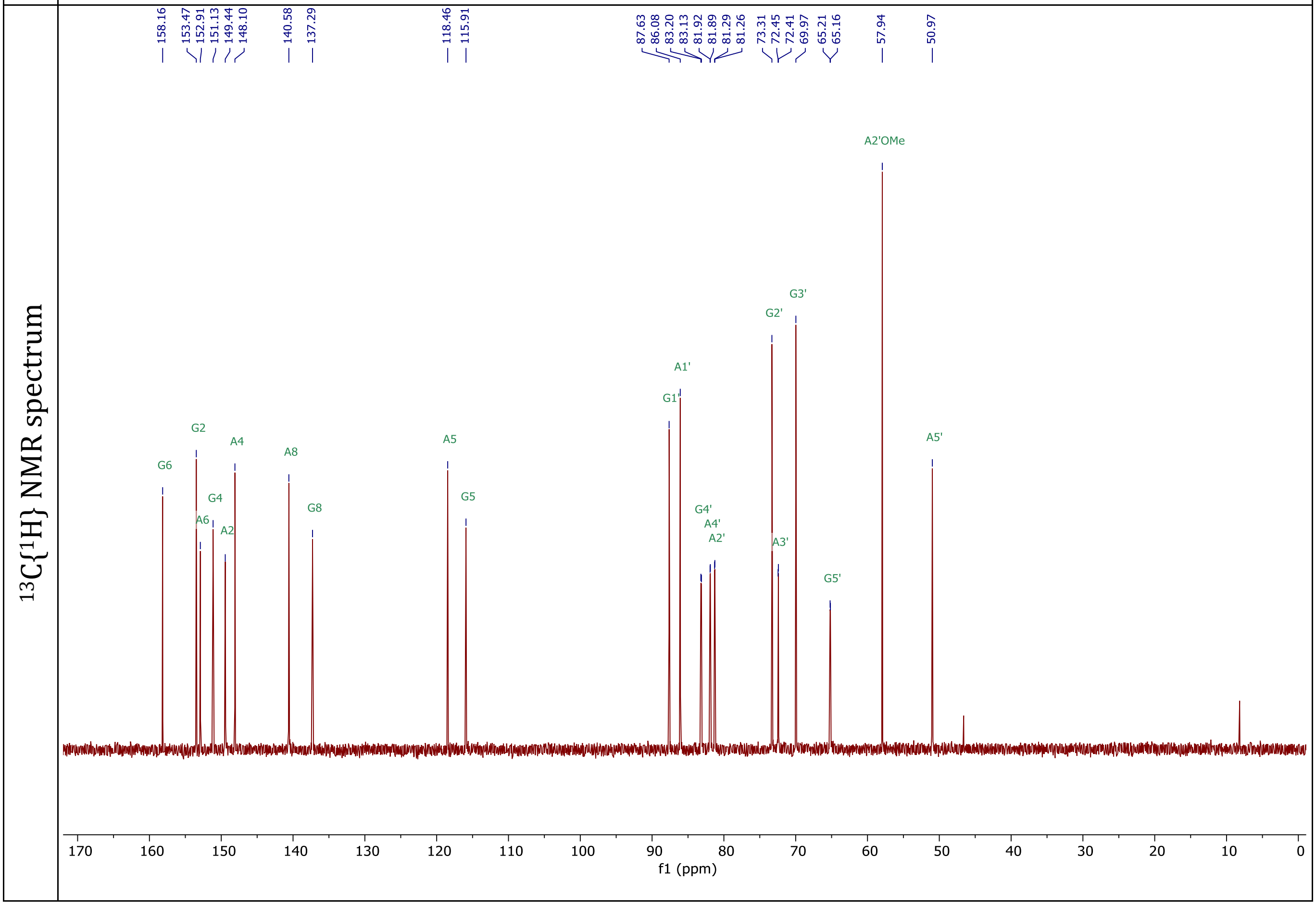
13C{1H} NMR spectrum
158.16
153.47
152.91
151.13
149.44
148.10
140.58
137.29
118.46
115.91
87.63
86.08
83.20
83.13
81.92
81.89
81.29
81.26
73.31
72.45
72.41
69.97
65.21
65.16
57.94
50.97
G6
G2
A6
G4
A2
A4
A8
G8
A5
G5
G1'
A1'
G4'
A4'
A2'
G2'
G3'
A3'
G5'
A2'OMe
A5'
f1 (ppm)

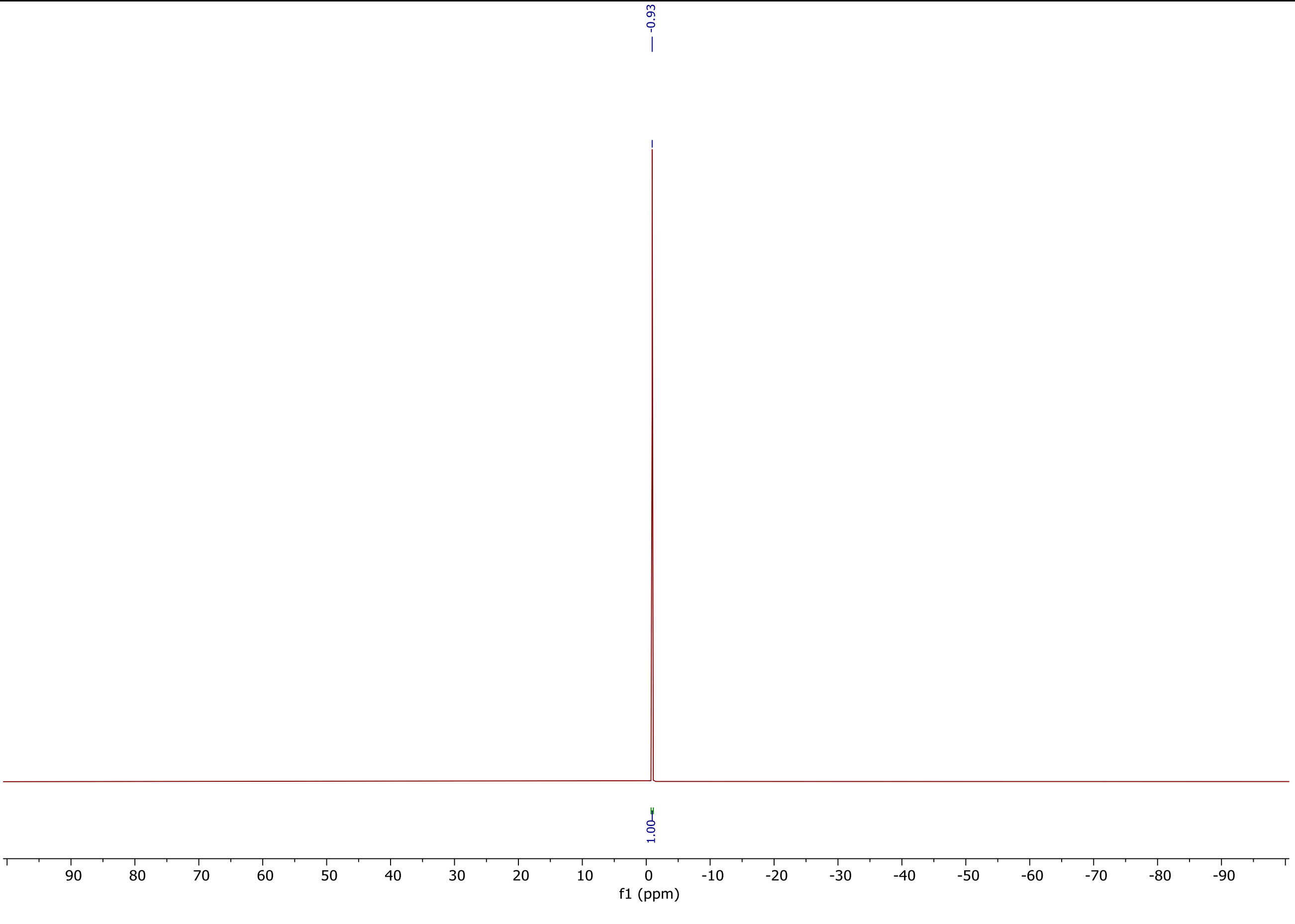

$^{31}P\{^{1}H\}$ NMR spectrum

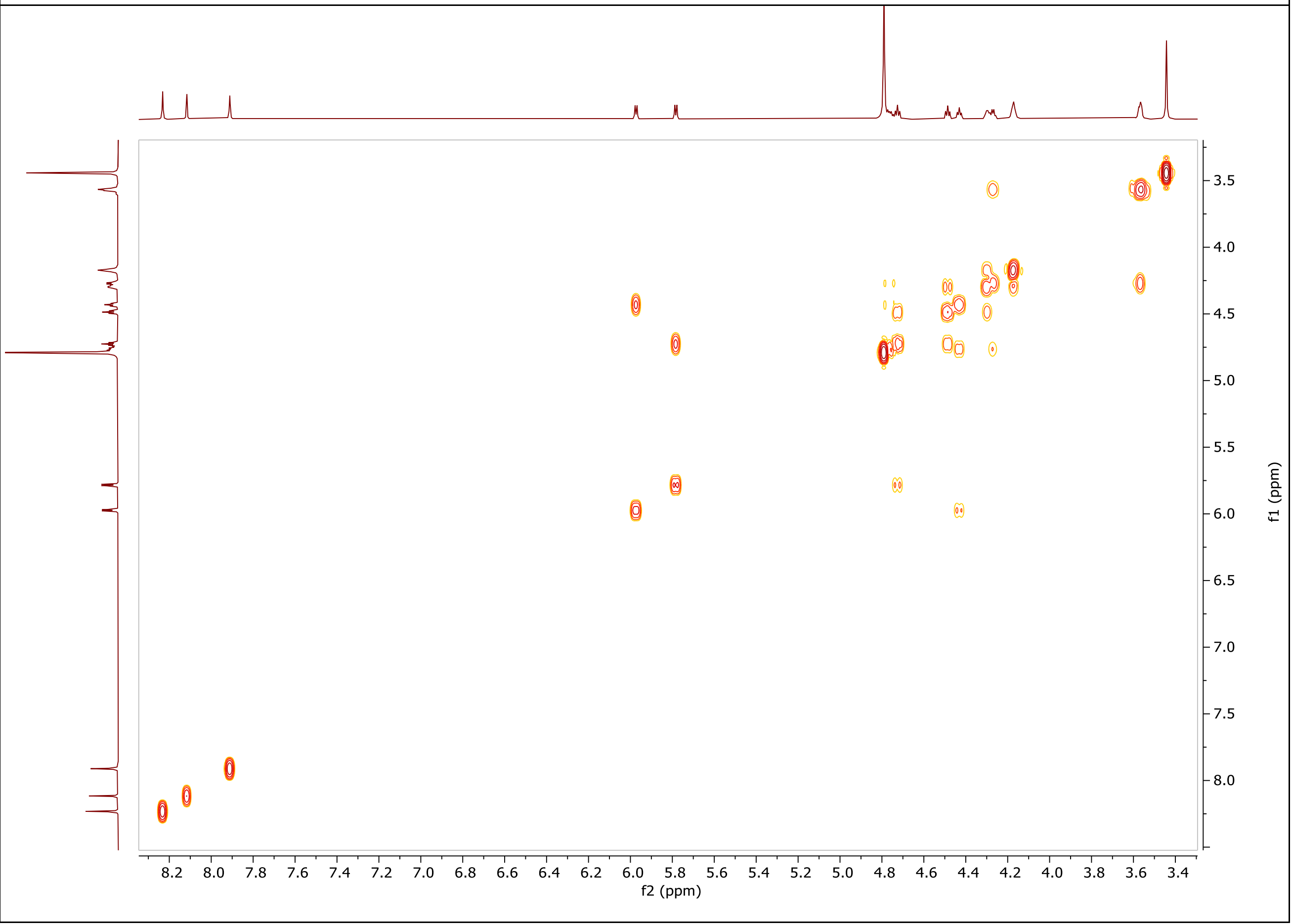

^{1}H-^{1}H COSY spectrum

^{1}H-^{1}H NOESY spectrum

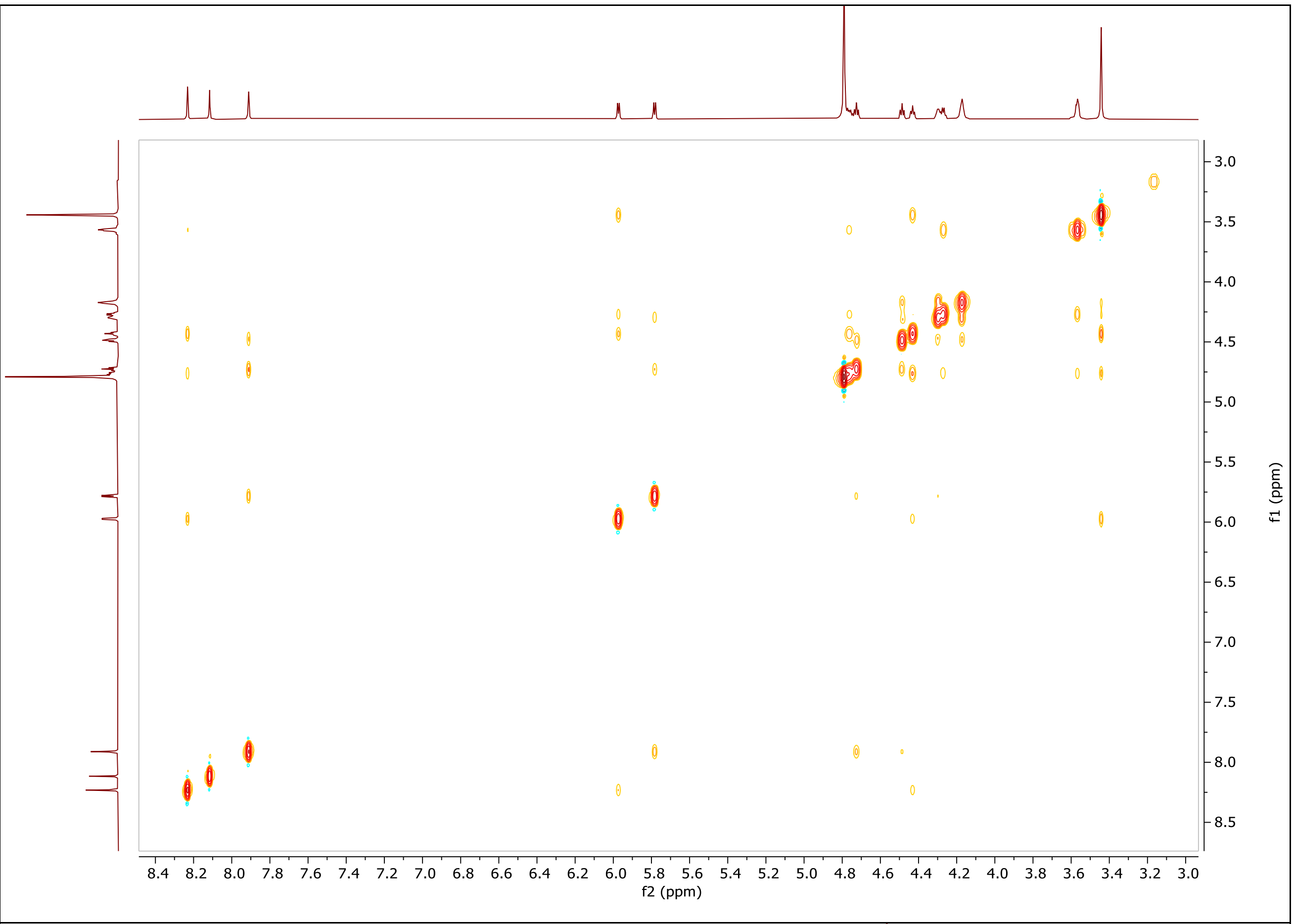

^{1}H-^{13}C HSQC spectrum

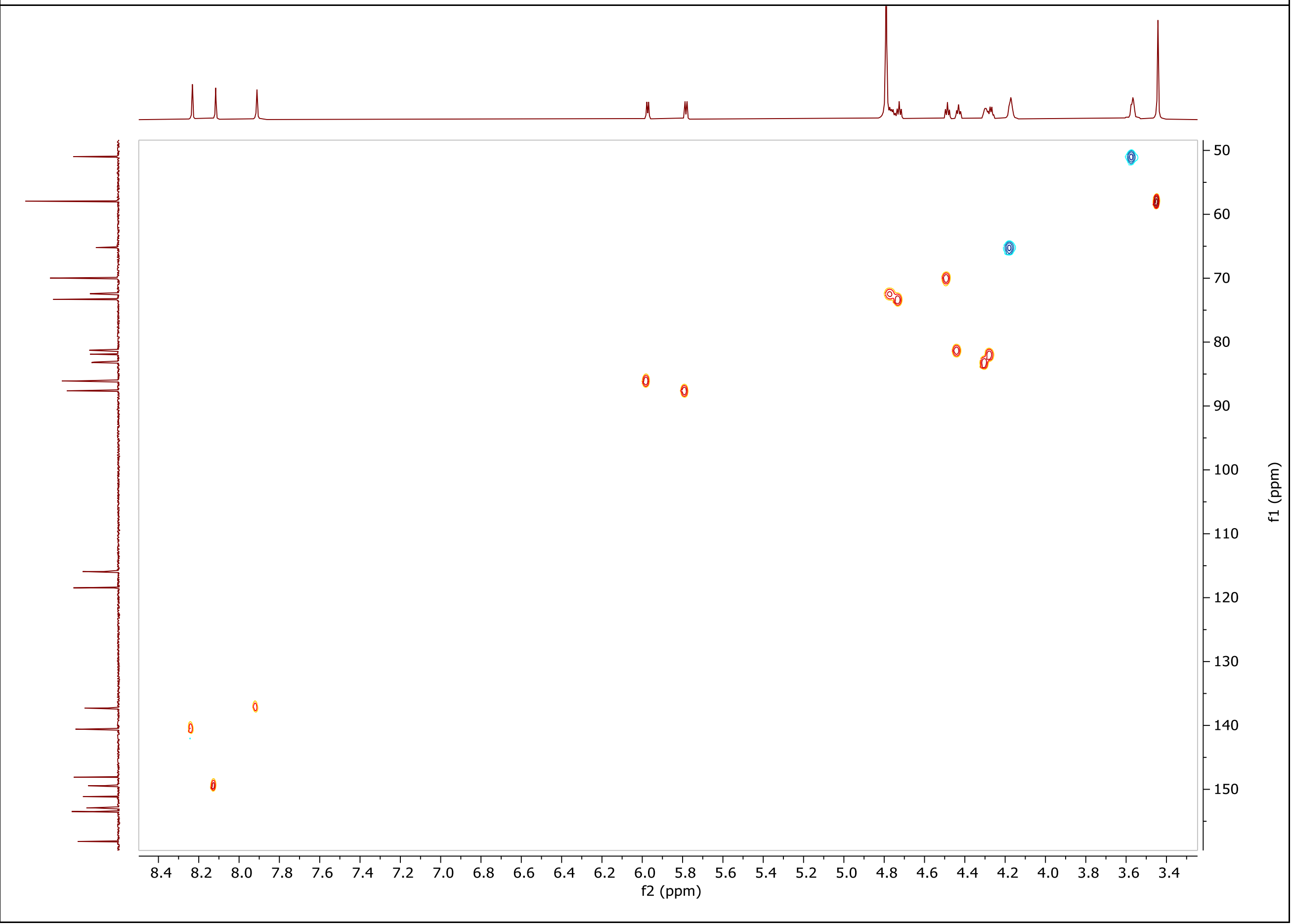

^{1}H-^{13}C HMBC spectrum

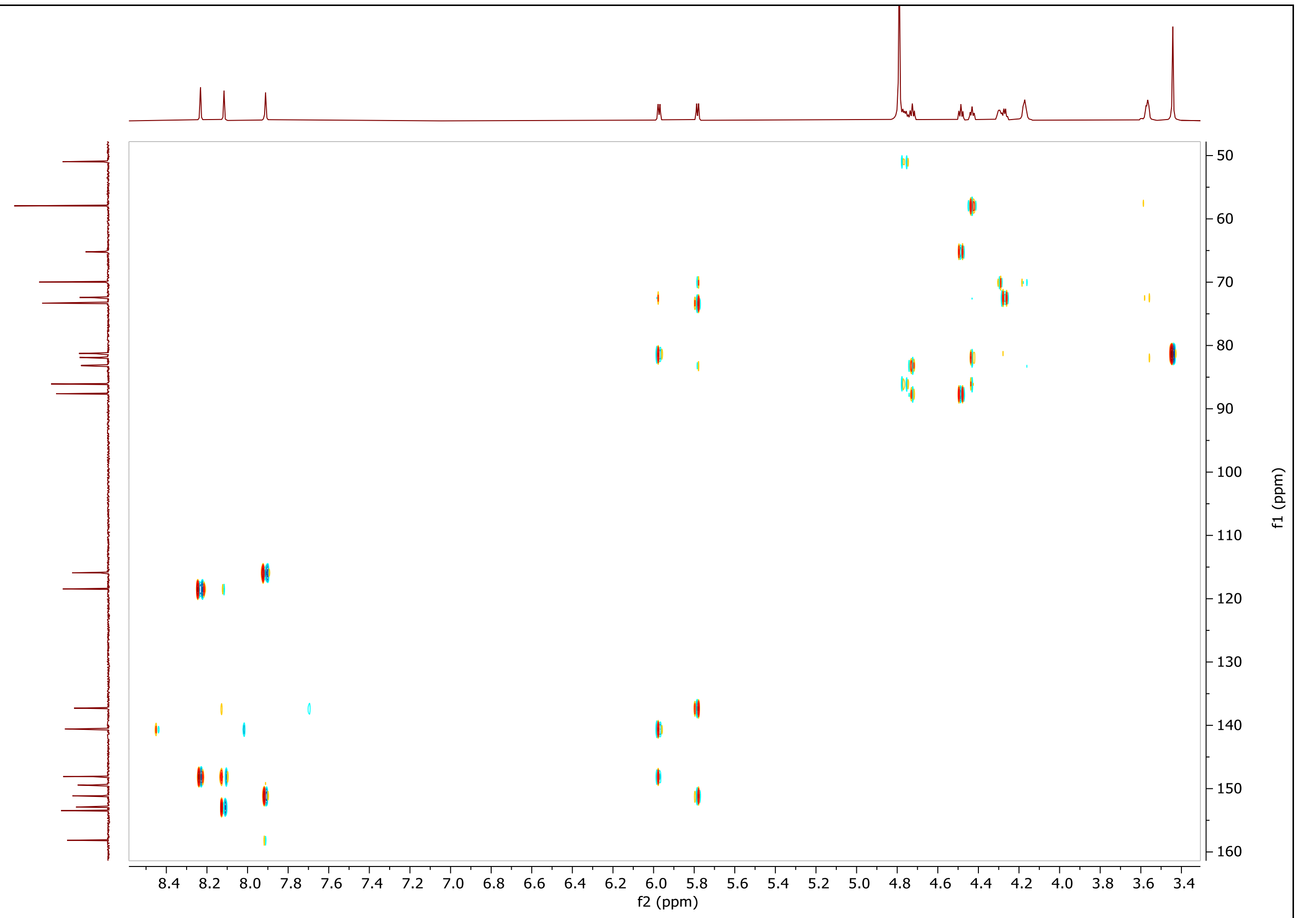

^{1}H-^{31}P HMBC spectrum

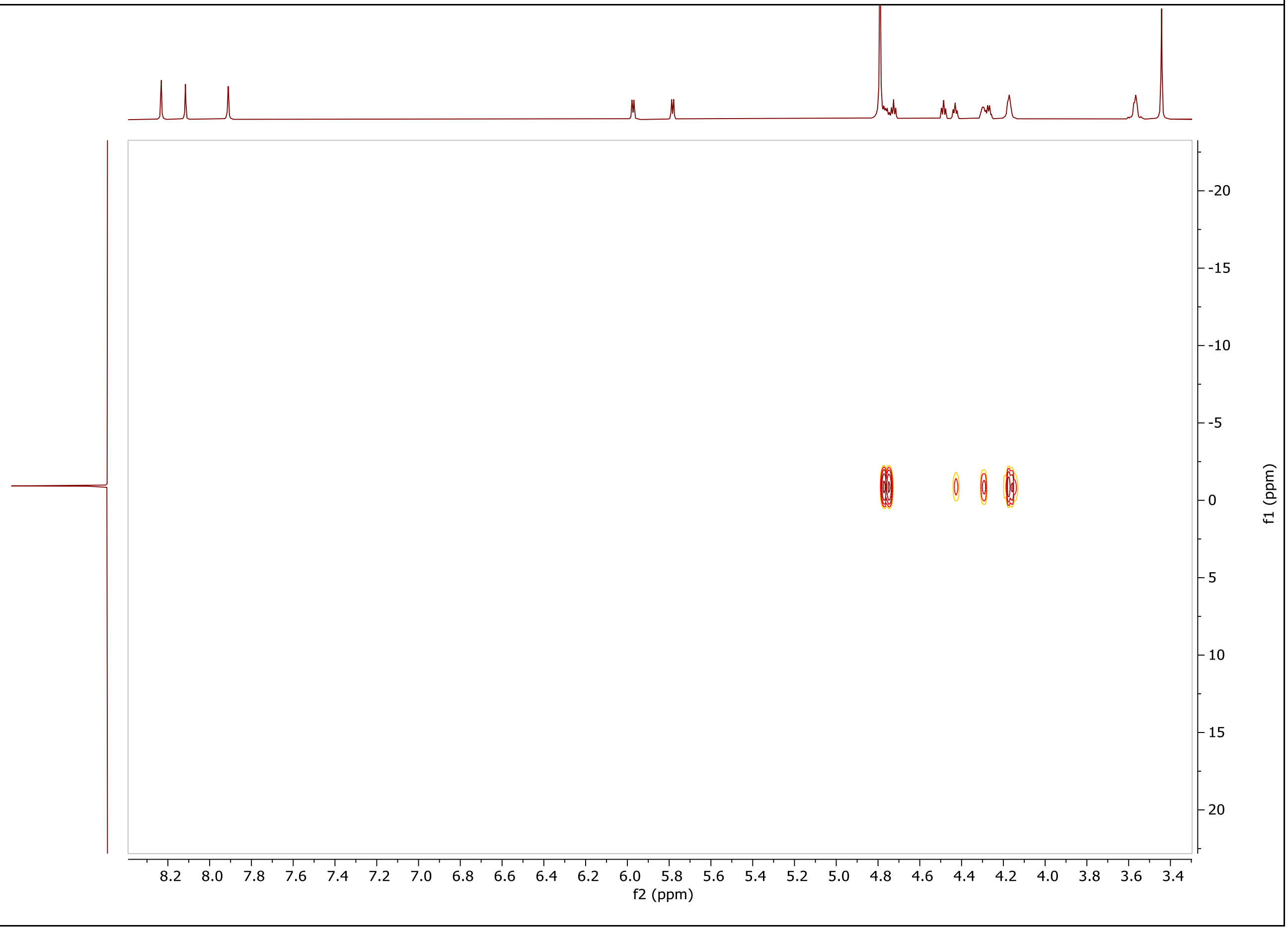

Supplement: Supplementary file 1 — Supplementary Information [file 41467_2025_64257_MOESM1_ESM.pdf]
