# Supplementary material for: Enhancing cap-independent translation of linear mRNA
Source: Nat Commun. 2025 Oct 16;16:9205. doi: 10.1038/s41467-025-64257-6 (PMC12532787; doi:10.1038/s41467-025-64257-6)
Supplement: Supplementary file 2 — Description of Additional Supplementary Information [file 41467_2025_64257_MOESM2_ESM.pdf]

Description of Additional Supplementary Files

File Name: Supplementary Movie 1
Description: Cellular uptake of 5′-AF647 iSyn-EGFP mRNA and expression of EGFP.

File Name: Supplementary Movie 2
Description: Cellular uptake of 5′-AF647 iSyn-EGFP mRNA (single cells).

File Name: Supplementary Movie 3
Description: 5′-AF647 iSynEGFP mRNA dynamics in single cell.

File Name: Supplementary Movie 4
Description: 5′-AF647 iSynEGFP mRNA split during cell division.

File Name: Supplementary Movie 5
Description: 5′-AF647 iSynEGFP mRNA localisation in single cell.

File Name: Supplementary Movie 6
Description: 5′-AF647 iSynEGFP mRNA localisation in single dividing cell.
