# Supplementary material for: Enhancing cap-independent translation of linear mRNA
Source: Nat Commun. 2025 Oct 16;16:9205. doi: 10.1038/s41467-025-64257-6 (PMC12532787; doi:10.1038/s41467-025-64257-6)
Supplement: Supplementary file 9 — Reporting Summary [file 41467_2025_64257_MOESM9_ESM.pdf]

nature portfolio

Corresponding author(s): Sebastian Golojuch
Afaf H. El-Sagheer
Tom Brown

Last updated by author(s): Aug 21, 2025

Reporting Summary

Nature Portfolio wishes to improve the reproducibility of the work that we publish. This form provides structure for consistency and transparency in reporting. For further information on Nature Portfolio policies, see our Editorial Policies and the Editorial Policy Checklist.

Statistics

For all statistical analyses, confirm that the following items are present in the figure legend, table legend, main text, or Methods section.

n/a	Confirmed	
☐	☒	The exact sample size (*n*) for each experimental group/condition, given as a discrete number and unit of measurement
☐	☒	A statement on whether measurements were taken from distinct samples or whether the same sample was measured repeatedly
☐	☒	The statistical test(s) used AND whether they are one- or two-sided<br>*Only common tests should be described solely by name; describe more complex techniques in the Methods section.*
☒	☐	A description of all covariates tested
☐	☒	A description of any assumptions or corrections, such as tests of normality and adjustment for multiple comparisons
☐	☒	A full description of the statistical parameters including central tendency (e.g. means) or other basic estimates (e.g. regression coefficient) AND variation (e.g. standard deviation) or associated estimates of uncertainty (e.g. confidence intervals)
☐	☒	For null hypothesis testing, the test statistic (e.g. *F*, *t*, *r*) with confidence intervals, effect sizes, degrees of freedom and *P* value noted<br>*Give P values as exact values whenever suitable.*
☒	☐	For Bayesian analysis, information on the choice of priors and Markov chain Monte Carlo settings
☒	☐	For hierarchical and complex designs, identification of the appropriate level for tests and full reporting of outcomes
☐	☒	Estimates of effect sizes (e.g. Cohen's *d*, Pearson's *r*), indicating how they were calculated

Our web collection on statistics for biologists contains articles on many of the points above.

Software and code

Policy information about availability of computer code

Data collection	FACSDiva 9.2 (BD), CFX Manager 3.1 (Bio-Rad), OpenLab CDS 2.7 (Agilent Technologies), NanoDrop 2000/2000c 1.6 (Thermo Scientific), MassLynx 4.1 (Waters), 2100 Expert B.02.11 (Agilent Technologies), OligoNet 1.0.1 (Applied Biosystems), cellSens Dimension 3.2 (Olympus), Omega 5.10 R2 (BMG LABTECH)
Data analysis	FlowJo 10.8 (BD Life Sciences), GraphPad Prism 10.0.1 (GraphPad Software), Biolegend LEGENDplex™ 2024-06-15 (Qognit), MestReNova 14.3 (Mestrelab Research), MassLynx 4.1 (Waters), 2100 Expert B.02.11 (Agilent Technologies), Fiji 2.14.0 (National Institutes of Health, USA), Imaris 10.2 (Bitplane), JACoP 1.1.10 (Fiji plugin), Bleach Correction 2.1.0 (Fiji plugin), Huygens 24.04 (Scientific Volume Imaging), DaVinci Resolve Studio 18.5 (Blackmagic Design), Excel LTSC MSO 16.0.14332.20631 (Microsoft), OriginPro 2023 10.0.0.154 (OriginLab Corporation), SnapGene 8.1 (GSL Biotech)

For manuscripts utilizing custom algorithms or software that are central to the research but not yet described in published literature, software must be made available to editors and reviewers. We strongly encourage code deposition in a community repository (e.g. GitHub). See the Nature Portfolio guidelines for submitting code & software for further information.

Data

Policy information about availability of data

All manuscripts must include a data availability statement. This statement should provide the following information, where applicable:

- Accession codes, unique identifiers, or web links for publicly available datasets
- A description of any restrictions on data availability
- For clinical datasets or third party data, please ensure that the statement adheres to our policy

Source data are provided with this paper. Raw microscopy data generated in this study have been submitted to Zenodo (https://doi.org/10.5281/zenodo.14589630)

Research involving human participants, their data, or biological material

Policy information about studies with human participants or human data. See also policy information about sex, gender (identity/presentation), and sexual orientation and race, ethnicity and racism.

Reporting on sex and gender	n/a
Reporting on race, ethnicity, or other socially relevant groupings	n/a
Population characteristics	n/a
Recruitment	n/a
Ethics oversight	n/a

Note that full information on the approval of the study protocol must also be provided in the manuscript.

Field-specific reporting

Please select the one below that is the best fit for your research. If you are not sure, read the appropriate sections before making your selection.

[x] Life sciences [ ] Behavioural & social sciences [ ] Ecological, evolutionary & environmental sciences

For a reference copy of the document with all sections, see nature.com/documents/nr-reporting-summary-flat.pdf

Life sciences study design

All studies must disclose on these points even when the disclosure is negative.

Sample size	No sample-size calculation was performed. Sample sizes were chosen to ensure the reproducibility of the results.
Data exclusions	No data were excluded from the analyses.
Replication	Most experiments were replicated three times. The information regarding replication is included in the figure legends. All attempts at replication were successful.
Randomization	Randomisation was not relevant to this study. All experiments were carried out under controlled experimental conditions.
Blinding	Blinding was not relevant to this study. The experiments were performed and the results were analysed by the experimenters.

Reporting for specific materials, systems and methods

We require information from authors about some types of materials, experimental systems and methods used in many studies. Here, indicate whether each material, system or method listed is relevant to your study. If you are not sure if a list item applies to your research, read the appropriate section before selecting a response.

Materials & experimental systems

n/a	Involved in the study
☒	☐ Antibodies
☐	☒ Eukaryotic cell lines
☒	☐ Palaeontology and archaeology
☒	☐ Animals and other organisms
☒	☐ Clinical data
☒	☐ Dual use research of concern
☒	☐ Plants

Methods

n/a	Involved in the study
☒	☐ ChIP-seq
☐	☒ Flow cytometry
☒	☐ MRI-based neuroimaging

Eukaryotic cell lines

Policy information about cell lines and Sex and Gender in Research

Cell line source(s)	HepG2 cells (ATCC #HB-8065) were purchased from ATCC. MUTZ-3 cells (DSMZ #ACC 295) were purchased from DSMZ. HEK293 cells (ATCC #CRL-1573) were a gift from Elizabeth J. Robertson (Sir William Dunn School of Pathology, University of Oxford). HeLa (ATCC #CCL-2) and HCT-116 (ATCC #CCL-247) cells were a gift from Katherine A. Vallis (Oxford Institute for Radiation Oncology, Department of Oncology, University of Oxford).
Authentication	All cell lines used in this study were authenticated by ECACC. STR DNA profiles for all cell lines were generated and compared to the profiles from the Cellosaurus database (ref. CVCL_0045 for HEK293, ref. CVCL_0030 for HeLa, ref. CVCL_1433 for MUTZ-3, ref. CVCL_0027 for HepG2, ref CVCL_0291 for HCT-116). For all tested cell lines, the profiles matched.
Mycoplasma contamination	All cell lines used in this study tested negative for mycoplasma contamination.
Commonly misidentified lines (See ICLAC register)	No commonly misidentified cell lines were used in this study.

Plants

Seed stocks	n/a
Novel plant genotypes	n/a
Authentication	n/a

Flow Cytometry

Plots

Confirm that:

☒ The axis labels state the marker and fluorochrome used (e.g. CD4-FITC).

☒ The axis scales are clearly visible. Include numbers along axes only for bottom left plot of group (a 'group' is an analysis of identical markers).

☒ All plots are contour plots with outliers or pseudocolor plots.

☒ A numerical value for number of cells or percentage (with statistics) is provided.

Methodology

Sample preparation	Treated cells in 96-well plates were detached with TrypLE™ Express Enzyme (1X), no phenol red (#12604013, Thermo Fisher Scientific, 50 µL) and vigorously pipetted with PBS (#10010023, Thermo Fisher Scientific) containing 2% (v/v) fetal bovine serum (#A5209402, Thermo Fisher Scientific) and 2 mM EDTA (#E4884-425 100G, Sigma-Aldrich) (100 µL). Harvested cells were immediately analysed.
Instrument	LSRFortessa™ X-20 (BD Biosciences)
Software	FACSDiva 9.2 (BD), FlowJo 10.8 (BD Life Sciences)

Cell population abundance	~10,000 cell events per well after SSC-A vs FSC-A and FSC-H vs FSC-A gating were recorded.
Gating strategy	Gating strategies are shown in Supplementary Figs 4, 5, 6.

☒ Tick this box to confirm that a figure exemplifying the gating strategy is provided in the Supplementary Information.
