# Supplementary material for: Enhancing cap-independent translation of linear mRNA
Source: Nat Commun. 2025 Oct 16;16:9205. doi: 10.1038/s41467-025-64257-6 (PMC12532787; doi:10.1038/s41467-025-64257-6)
Supplement: Supplementary file 11 — Source Data [file 41467_2025_64257_MOESM11_ESM.zip › Source Data/Figure 3/electrophoregrams.pdf]

Assay Class: mRNA Nano
Data Path: C:\...pert\data\2024-09-07\2100_mRNA Nano_2024-09-07_14-52-59.xad
Created: 9/7/2024 2:52:58 pm
Modified: 12/20/2024 3:45:21 pm

Electrophoresis File Run Summary

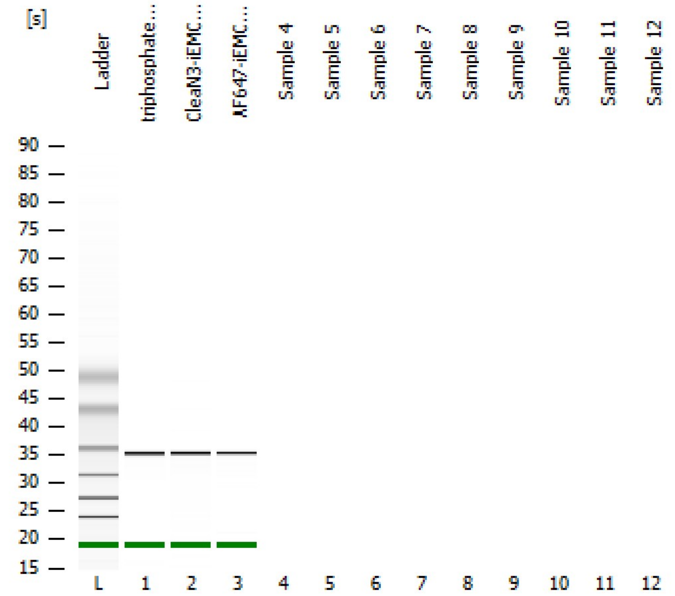

Instrument Information:

Instrument Name: DE54107469
Firmware: C.01.069
Serial#: DE54107469
Type: G2939A

Assay Information:

Assay Origin Path: C:\Program Files (x86)\Agilent\2100 bioanalyzer\2100 expert\assays\RNA\mRNA Nano Series II.xsy

Assay Class: mRNA Nano

Version: 2.5

Assay Comments: mRNA Analysis ng sensitivity

© Copyright 2003 - 2009 Agilent Technologies, Inc.

Chip Information:

Chip Lot #:

Reagent Kit Lot #:

Chip Comments:

triphosphate-iEMCV-EGFP-A120

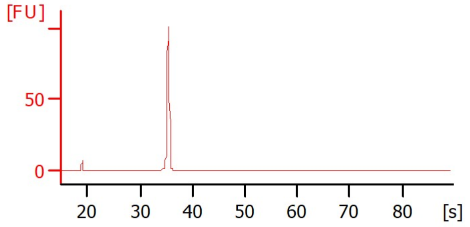

CleaN3-iEMCV-EGFP-A120

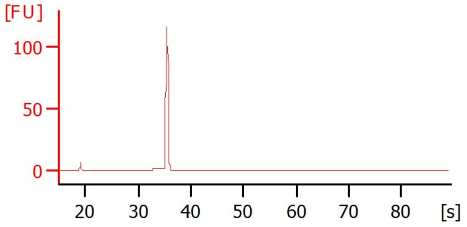

AF647-iEMCV-EGFP-A120

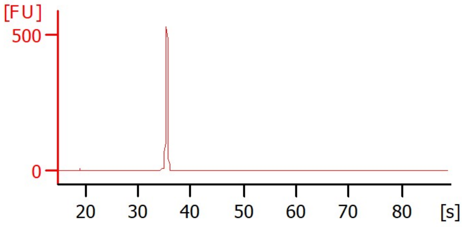

Assay Class: mRNA Nano
Data Path: C:\...pert\data\2024-09-07\2100_mRNA Nano_2024-09-07_14-52-59.xad
Created: 9/7/2024 2:52:58 pm
Modified: 12/20/2024 3:45:21 pm

Electrophoresis File Run Summary (Chip Summary)

Sample Name	Sample Comment	Status	Result Label	Result Color
triphosphate-iEMCV-EGFP-A120		✔		
CleaN3-iEMCV-EGFP-A120		✔		
AF647-iEMCV-EGFP-A120		✔		
Sample 4				
Sample 5				
Sample 6				
Sample 7				
Sample 8				
Sample 9				
Sample 10				
Sample 11				
Sample 12				
Ladder		✔		

Chip Lot # **Reagent Kit Lot #**

Chip Comments :

© Copyright 2003 - 2021 Agilent, Inc.

Assay Class: mRNA Nano
Data Path: C:\...pert\data\2024-09-07\2100_mRNA Nano_2024-09-07_14-52-59.xad
Created: 9/7/2024 2:52:58 pm
Modified: 12/20/2024 3:45:21 pm

Electrophoresis Assay Details

General Analysis Settings

Number of Available Sample and Ladder Wells (Max.) : 13
Minimum Visible Range [s] : 14
Maximum Visible Range [s] : 85
Start Analysis Time Range [s] : 15
End Analysis Time Range [s] : 84
Ladder Concentration [ng/µl] : 150
Lower Marker Concentration [ng/µl] : 0
Upper Marker Concentration [ng/µl] : 0
Used Lower Marker for Quantitation
Standard Curve Fit is Logarithmic
Show Data Aligned to Lower Marker

Integrator Settings

Integration Start Time [s] : 15
Integration End Time [s] : 84
Slope Threshold : 0.2
Height Threshold [FU] : 0.5
Area Threshold : 0.1
Width Threshold [s] : 0.2
Baseline Plateau [s] : 0.5

Filter Settings

Filter Width [s] : 0.5
Polynomial Order : 4

Ladder

Ladder Peak	Size
1	25
2	200
3	500
4	1000
5	2000
6	4000

© Copyright 2003 - 2021 Agilent, Inc.

Assay Class: mRNA Nano
Data Path: C:\...pert\data\2024-09-07\2100_mRNA Nano_2024-09-07_14-52-59.xad
Created: 9/7/2024 2:52:58 pm
Modified: 12/20/2024 3:45:21 pm

Electropherogram Summary

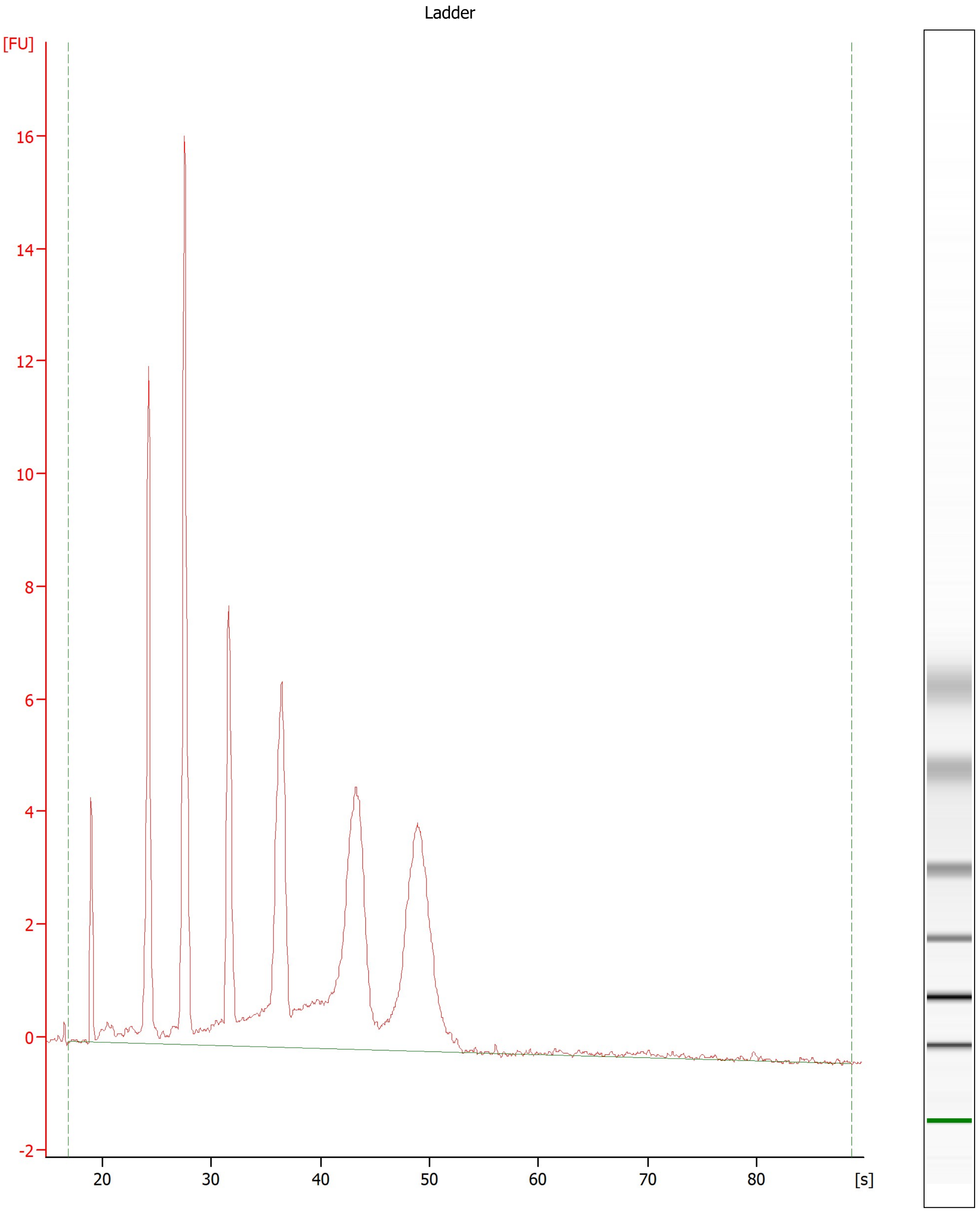

 © Copyright 2003 - 2021 Agilent, Inc.

Assay Class: mRNA Nano
Data Path: C:\...pert\data\2024-09-07\2100_mRNA Nano_2024-09-07_14-52-59.xad

Created: 9/7/2024 2:52:58 pm
Modified: 12/20/2024 3:45:21 pm

Electropherogram Summary Continued ...

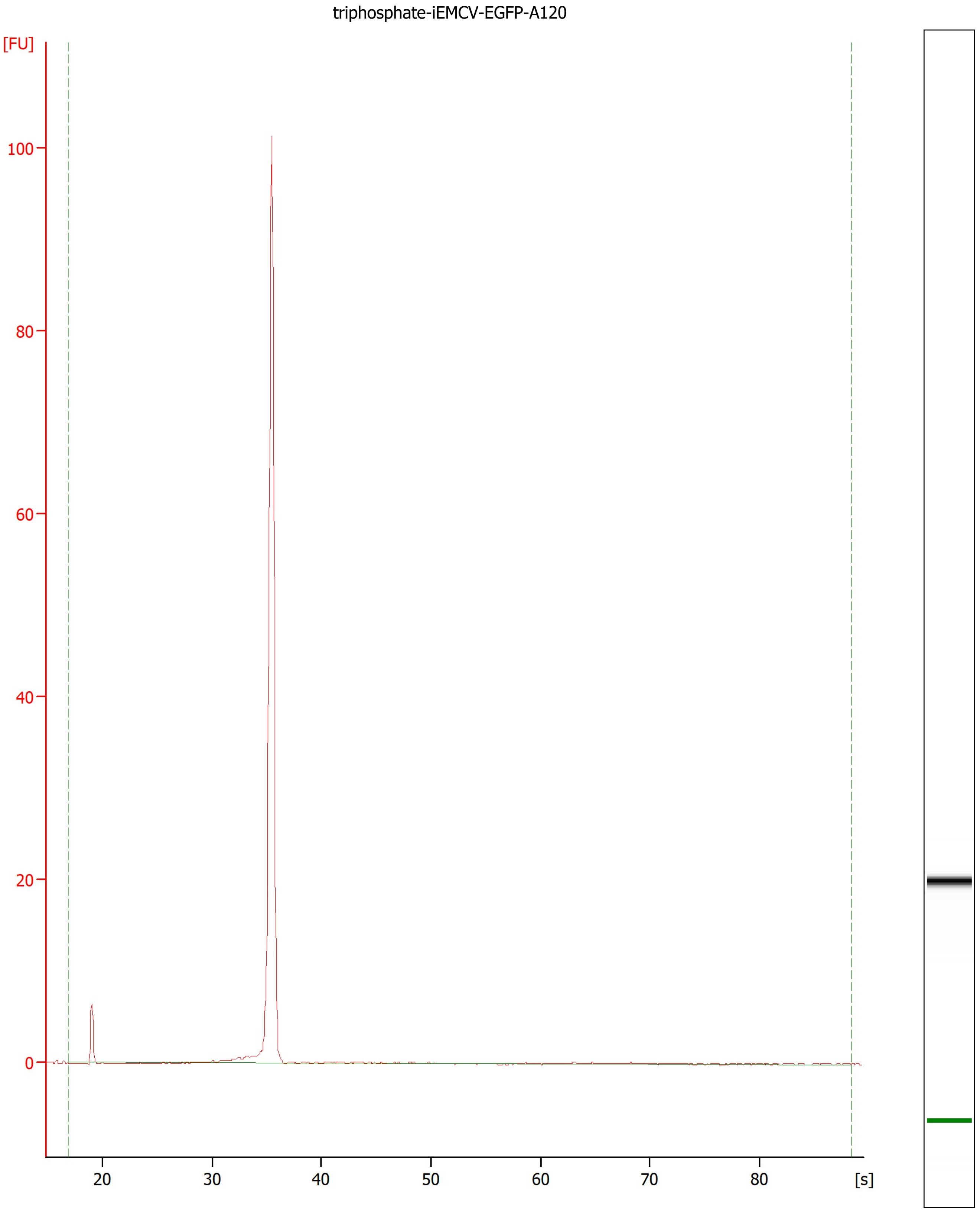

Assay Class: mRNA Nano
Data Path: C:\...pert\data\2024-09-07\2100_mRNA Nano_2024-09-07_14-52-59.xad

Created: 9/7/2024 2:52:58 pm
Modified: 12/20/2024 3:45:21 pm

Electropherogram Summary Continued ...

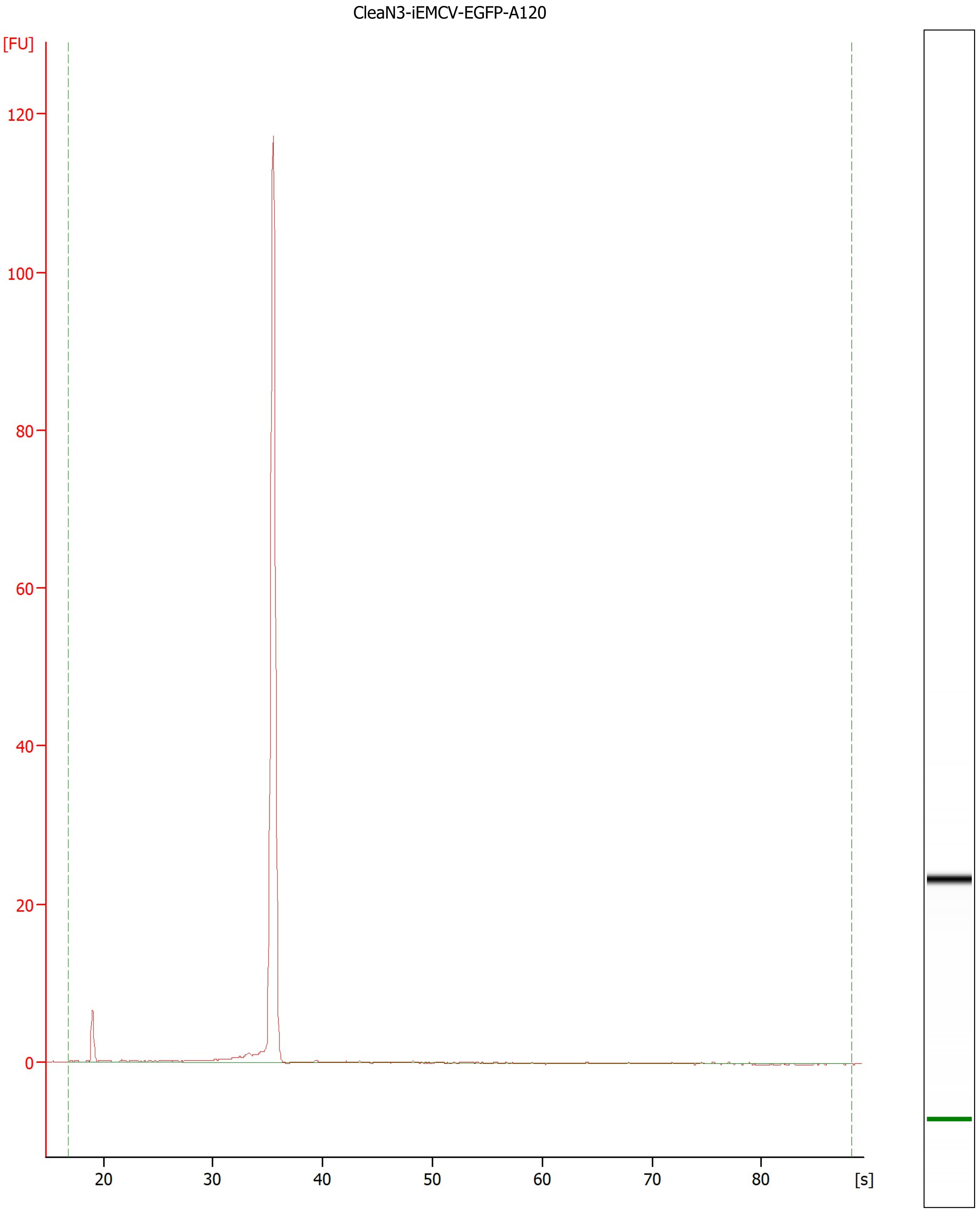

© Copyright 2003 - 2021 Agilent, Inc.

Assay Class: mRNA Nano
Data Path: C:\...pert\data\2024-09-07\2100_mRNA Nano_2024-09-07_14-52-59.xad

Created: 9/7/2024 2:52:58 pm
Modified: 12/20/2024 3:45:21 pm

Electropherogram Summary Continued ...

AF647-iEMCV-EGFP-A120

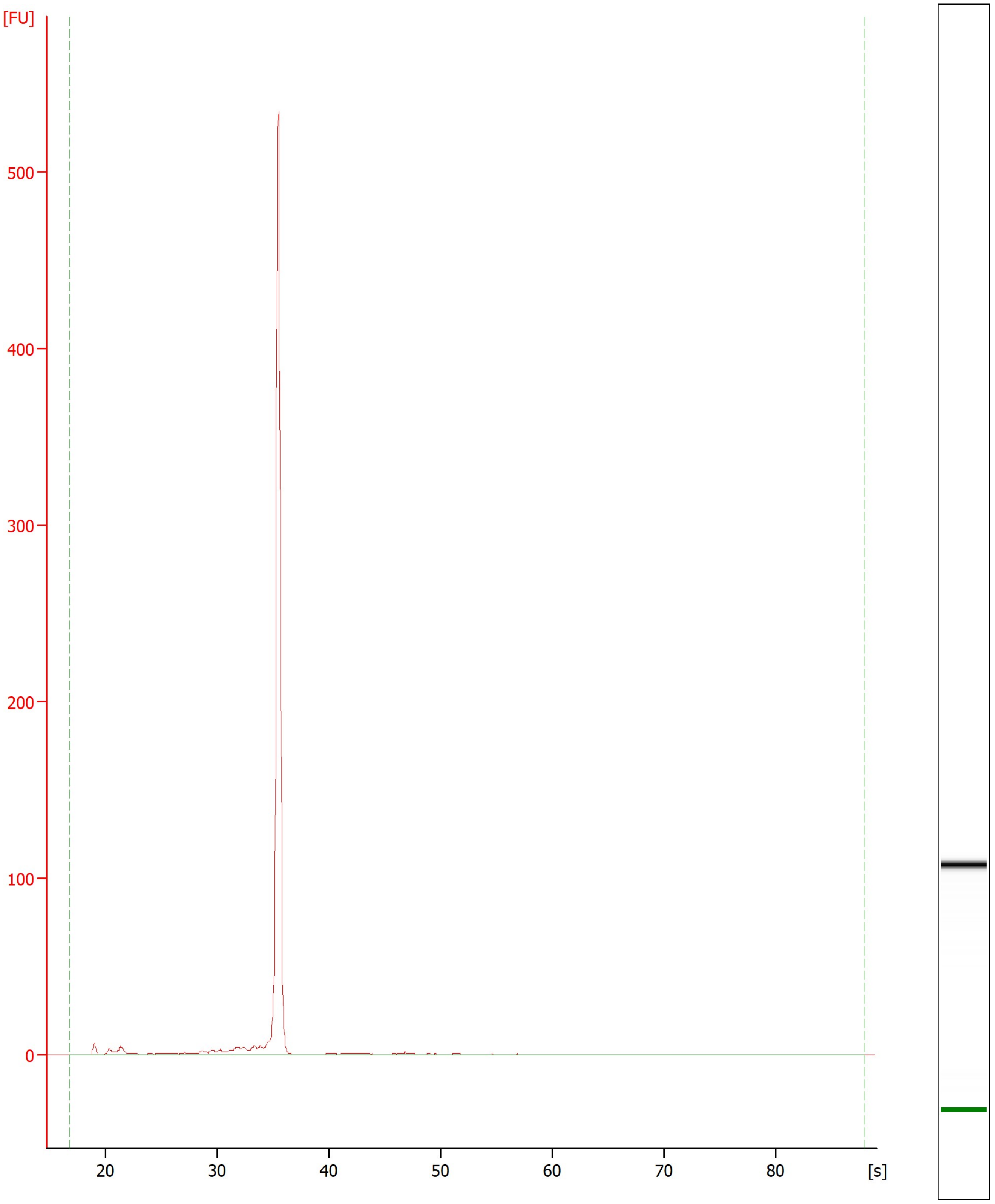

Assay Class: mRNA Nano
Data Path: C:\...pert\data\2024-09-07\2100_mRNA Nano_2024-09-07_14-52-59.xad

Created: 9/7/2024 2:52:58 pm
Modified: 12/20/2024 3:45:21 pm

Gel Image

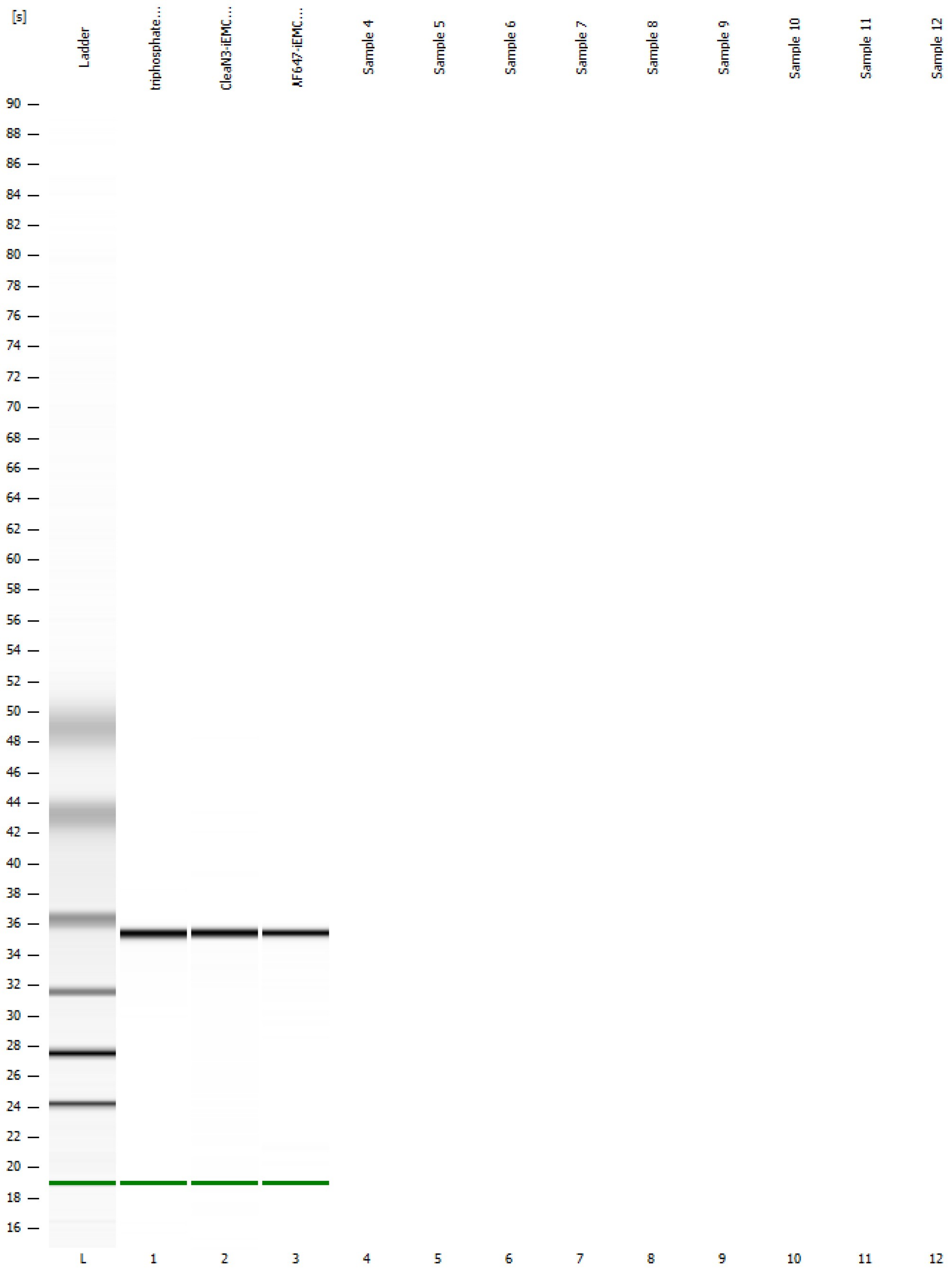

 © Copyright 2003 - 2021 Agilent, Inc.

Assay Class: mRNA Nano
Data Path: C:\...pert\data\2024-09-07\2100_mRNA Nano_2024-09-07_14-52-59.xad
Created: 9/7/2024 2:52:58 pm
Modified: 12/20/2024 3:45:21 pm

Invalid Samples

Sample 4 has not been run, no results available.

Sample 5 has not been run, no results available.

Sample 6 has not been run, no results available.

Sample 7 has not been run, no results available.

Sample 8 has not been run, no results available.

Sample 9 has not been run, no results available.

Sample 10 has not been run, no results available.

Sample 11 has not been run, no results available.

Sample 12 has not been run, no results available.

 © Copyright 2003 - 2021 Agilent, Inc.
