# Supplementary material for: Enhancing cap-independent translation of linear mRNA
Source: Nat Commun. 2025 Oct 16;16:9205. doi: 10.1038/s41467-025-64257-6 (PMC12532787; doi:10.1038/s41467-025-64257-6)
Supplement: Supplementary file 11 — Source Data [file 41467_2025_64257_MOESM11_ESM.zip › Source Data/Supplementary Figure 1/internally modified mRNAs.pdf]

Assay Class: mRNA Nano
Data Path: C:\...pert\data\2024-09-10\2100_mRNA Nano_2024-09-10_16-38-44.xad
Created: 9/10/2024 4:38:43 pm
Modified: 12/20/2024 2:56:38 pm

## Electrophoresis File Run Summary

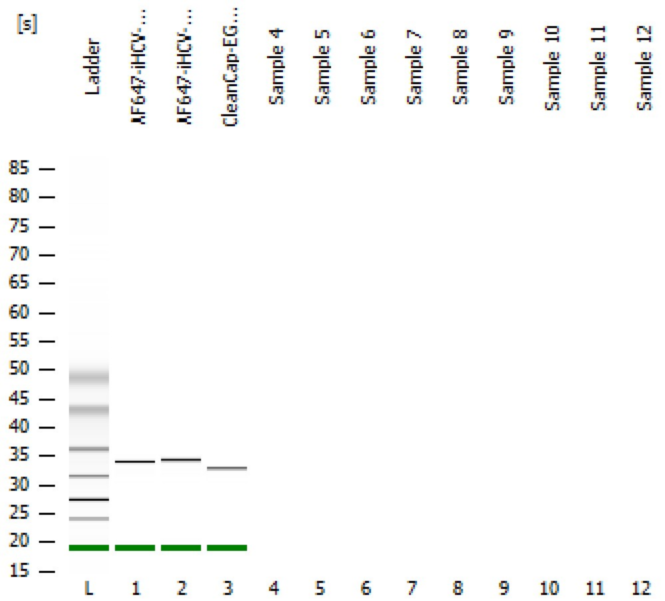


Instrument Information:

| | | | |
|---|---|---|---|
| Instrument Name: | DE54107469 | Firmware: | C.01.069 |
| Serial#: | DE54107469 | Type: | G2939A |

Assay Information:

| | |
|---|---|
| Assay Origin Path: | C:\Program Files (x86)\Agilent\2100 bioanalyzer\2100 expert\assays\RNA\mRNA Nano Series II.xsy |
| Assay Class: | mRNA Nano |
| Version: | 2.5 |
| Assay Comments: | mRNA Analysis ng sensitivity |

© Copyright 2003 - 2009 Agilent Technologies, Inc.

Chip Information:

Chip Lot #:

Reagent Kit Lot #:

Chip Comments:

**AF647-iHCV-EGFP-A120 100 m1Psi**

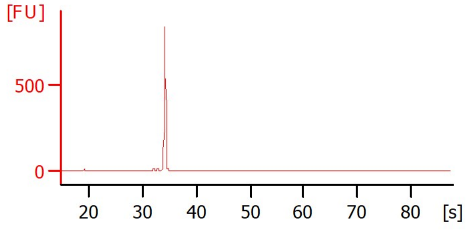


**AF647-iHCV-EGFP-A120 5 m6A**

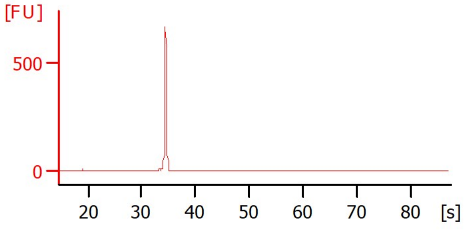


**CleanCap-EGFP-A120 100 m1Psi**

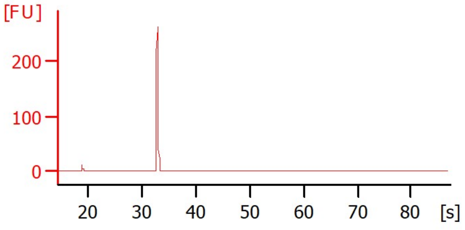

Assay Class: mRNA Nano
Data Path: C:\...pert\data\2024-09-10\2100_mRNA Nano_2024-09-10_16-38-44.xad

Created: 9/10/2024 4:38:43 pm
Modified: 12/20/2024 2:56:38 pm

## Electrophoresis File Run Summary (Chip Summary)

| Sample Name | Sample Comment | Status | Result Label | Result Color |
|---|---|---|---|---|
| AF647-iHCV-EGFP-A120 100 m1Psi | | ✔ | | |
| AF647-iHCV-EGFP-A120 5 m6A | | ✔ | | |
| CleanCap-EGFP-A120 100 m1Psi | | ✔ | | |
| Sample 4 | | | | |
| Sample 5 | | | | |
| Sample 6 | | | | |
| Sample 7 | | | | |
| Sample 8 | | | | |
| Sample 9 | | | | |
| Sample 10 | | | | |
| Sample 11 | | | | |
| Sample 12 | | | | |
| Ladder | | ✔ | | |

**Chip Lot #** **Reagent Kit Lot #**

**Chip Comments :**

 © Copyright 2003 - 2021 Agilent, Inc. 

Assay Class: mRNA Nano
Data Path: C:\...pert\data\2024-09-10\2100_mRNA Nano_2024-09-10_16-38-44.xad
Created: 9/10/2024 4:38:43 pm
Modified: 12/20/2024 2:56:38 pm

## Electrophoresis Assay Details

### General Analysis Settings

Number of Available Sample and Ladder Wells (Max.) : 13
Minimum Visible Range [s] : 14
Maximum Visible Range [s] : 85
Start Analysis Time Range [s] : 15
End Analysis Time Range [s] : 84
Ladder Concentration [ng/µl] : 150
Lower Marker Concentration [ng/µl] : 0
Upper Marker Concentration [ng/µl] : 0
Used Lower Marker for Quantitation
Standard Curve Fit is Logarithmic
Show Data Aligned to Lower Marker

### Integrator Settings

Integration Start Time [s] : 15
Integration End Time [s] : 84
Slope Threshold : 0.2
Height Threshold [FU] : 0.5
Area Threshold : 0.1
Width Threshold [s] : 0.2
Baseline Plateau [s] : 0.5

### Filter Settings

Filter Width [s] : 0.5
Polynomial Order : 4

### Ladder

| Ladder Peak | Size |
|---|---|
| 1 | 25 |
| 2 | 200 |
| 3 | 500 |
| 4 | 1000 |
| 5 | 2000 |
| 6 | 4000 |


© Copyright 2003 - 2021 Agilent, Inc.

Assay Class: mRNA Nano
Data Path: C:\...pert\data\2024-09-10\2100_mRNA Nano_2024-09-10_16-38-44.xad

Created: 9/10/2024 4:38:43 pm
Modified: 12/20/2024 2:56:38 pm

**Electropherogram Summary**

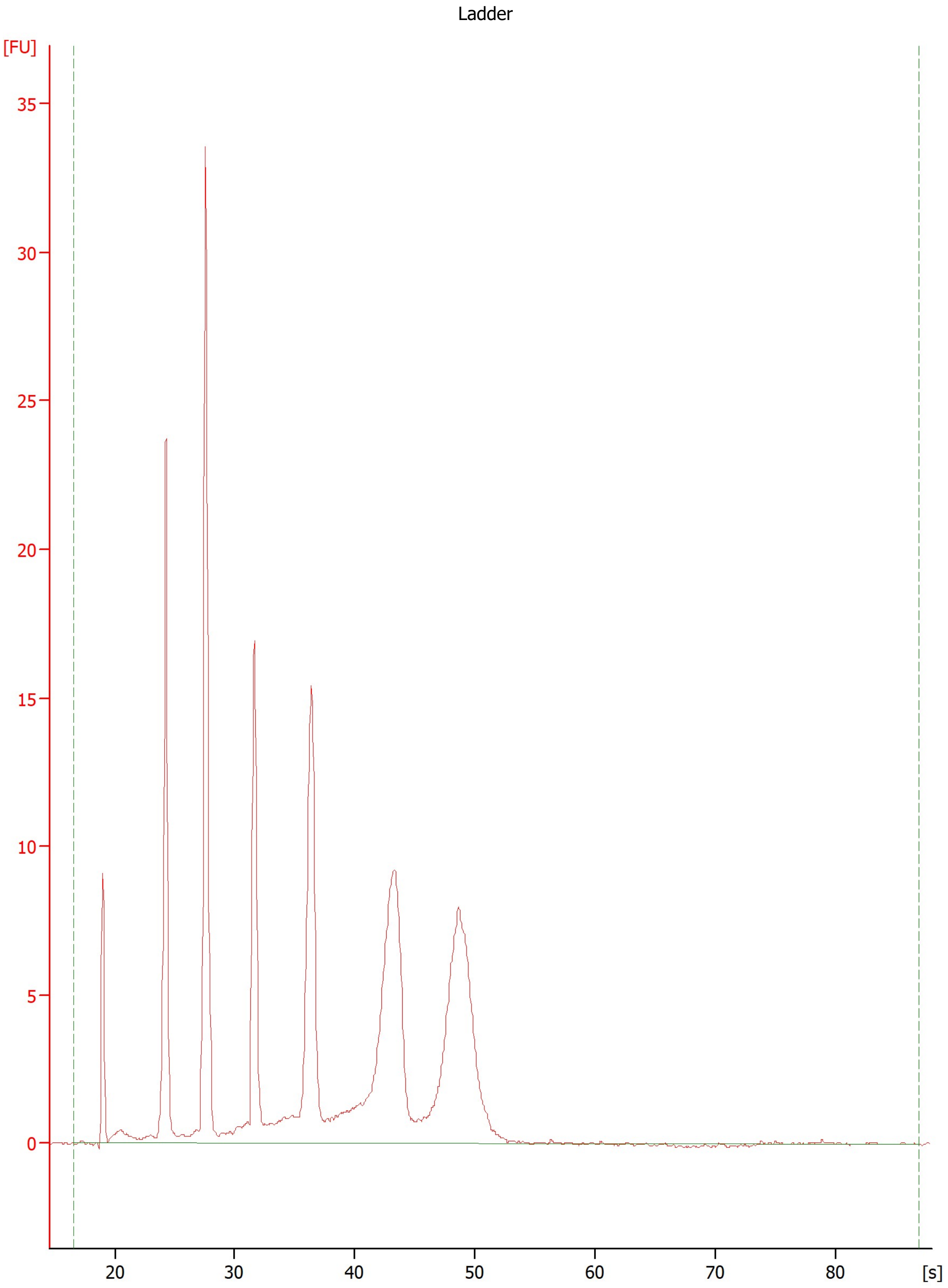


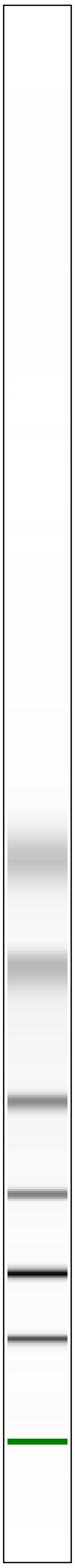

 © Copyright 2003 - 2021 Agilent, Inc. 

Assay Class: mRNA Nano
Data Path: C:\...pert\data\2024-09-10\2100_mRNA Nano_2024-09-10_16-38-44.xad
Created: 9/10/2024 4:38:43 pm
Modified: 12/20/2024 2:56:38 pm

**Electropherogram Summary Continued ...**

AF647-iHCV-EGFP-A120 100 m1Psi

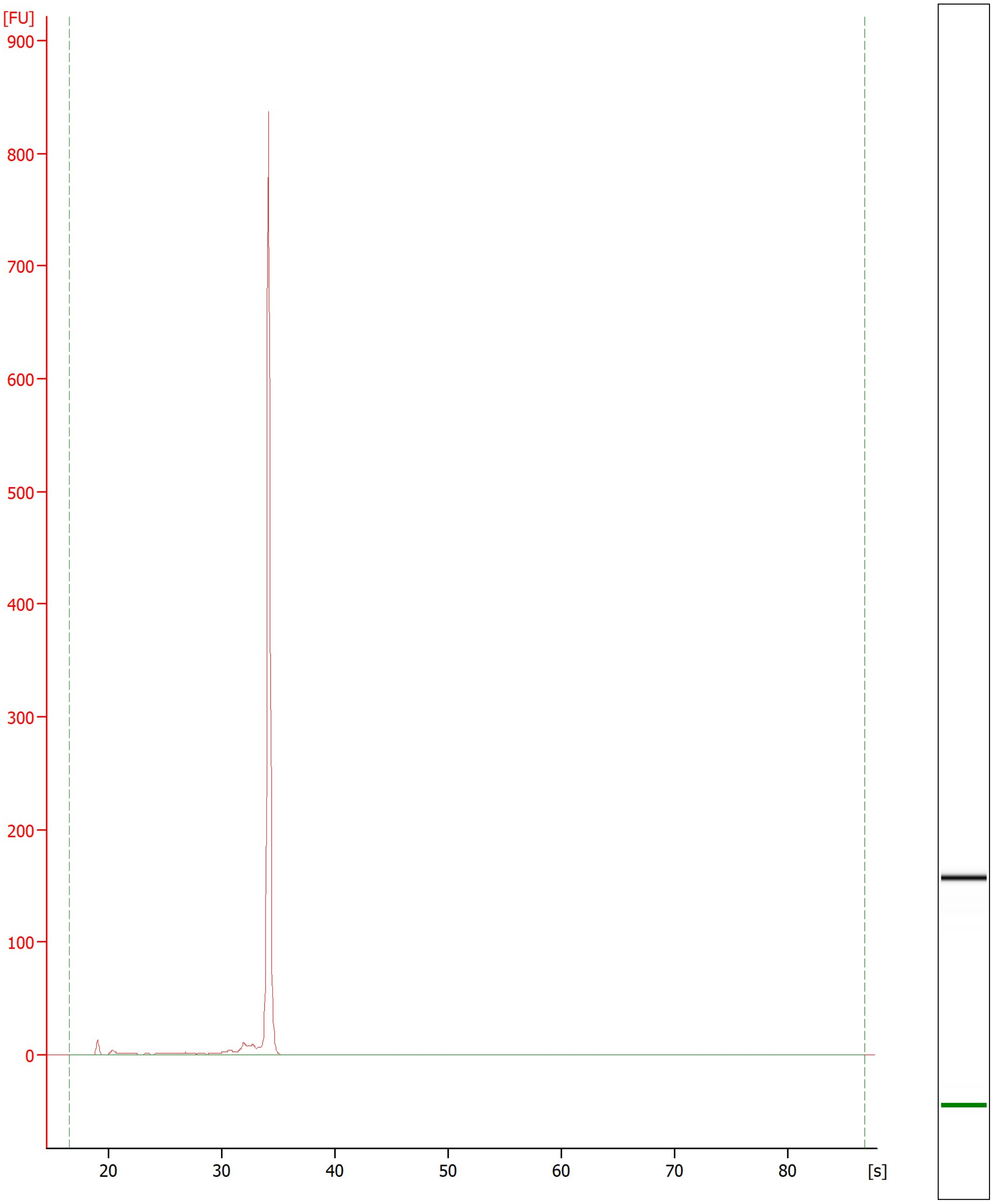



© Copyright 2003 - 2021 Agilent, Inc.

Assay Class: mRNA Nano
Data Path: C:\...pert\data\2024-09-10\2100_mRNA Nano_2024-09-10_16-38-44.xad
Created: 9/10/2024 4:38:43 pm
Modified: 12/20/2024 2:56:38 pm

**Electropherogram Summary Continued ...**

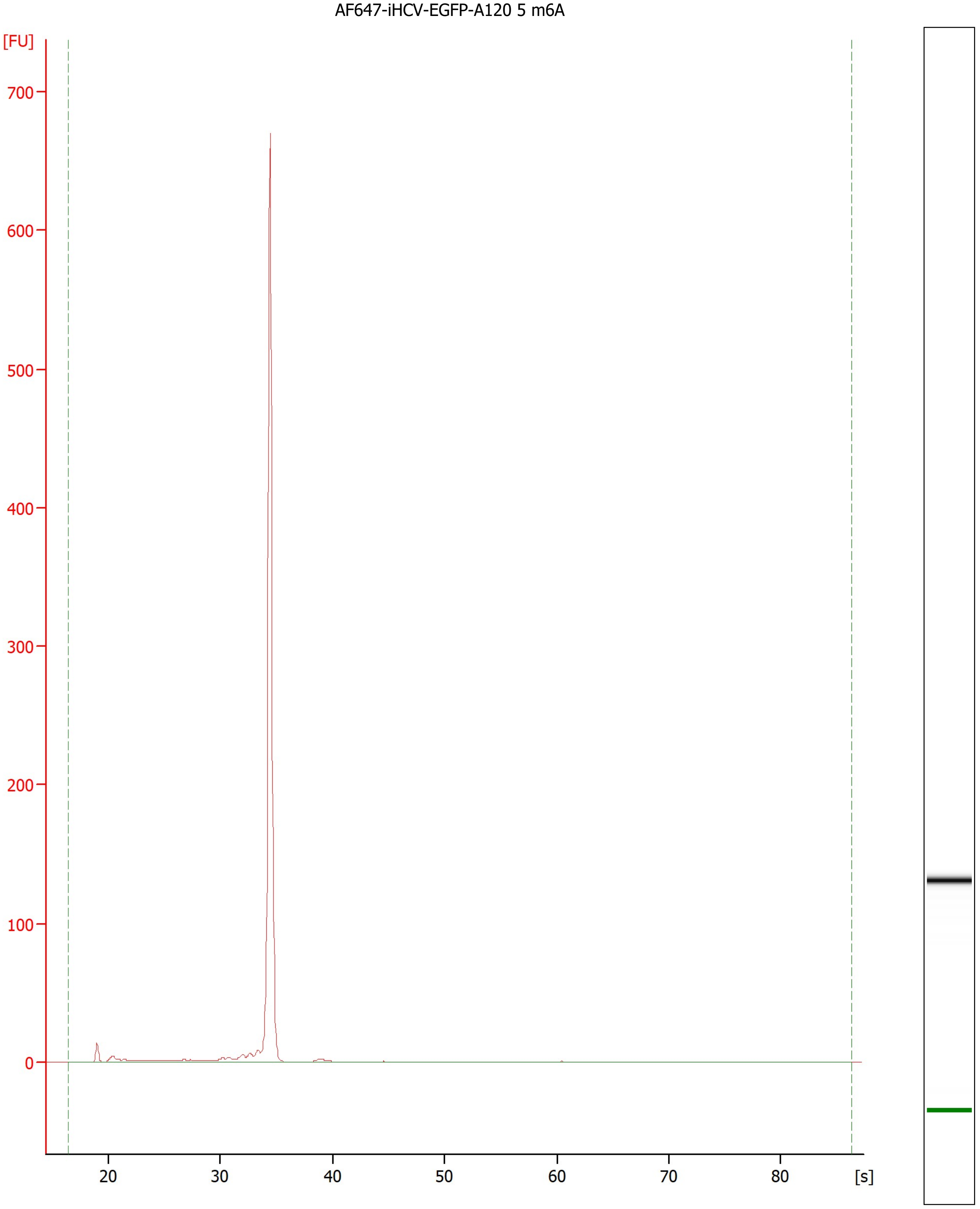


 © Copyright 2003 - 2021 Agilent, Inc. 

Assay Class: mRNA Nano
Data Path: C:\...pert\data\2024-09-10\2100_mRNA Nano_2024-09-10_16-38-44.xad
Created: 9/10/2024 4:38:43 pm
Modified: 12/20/2024 2:56:38 pm

**Electropherogram Summary Continued ...**

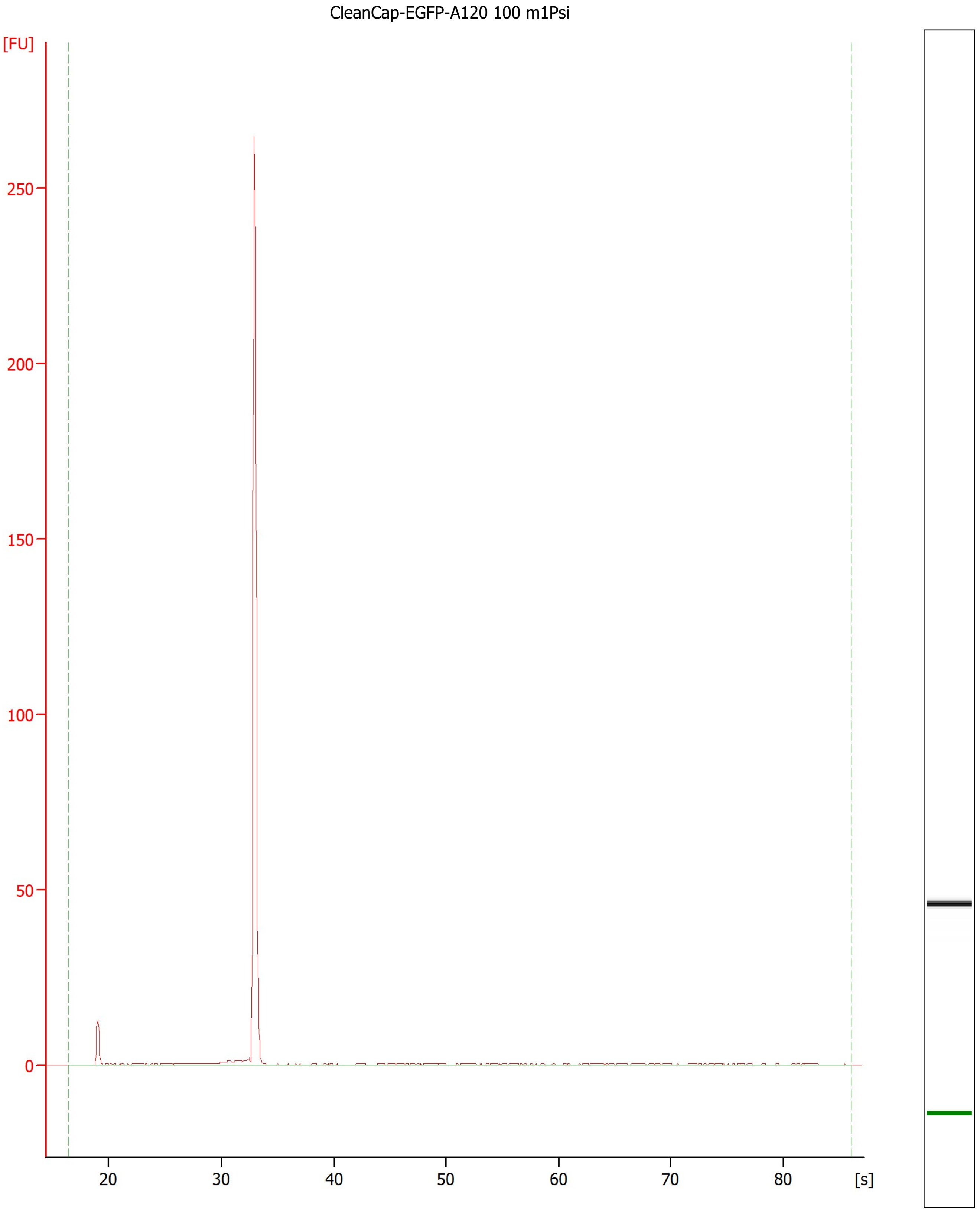



© Copyright 2003 - 2021 Agilent, Inc.

Assay Class: mRNA Nano
Data Path: C:\...pert\data\2024-09-10\2100_mRNA Nano_2024-09-10_16-38-44.xad

Created: 9/10/2024 4:38:43 pm
Modified: 12/20/2024 2:56:38 pm

**Gel Image**

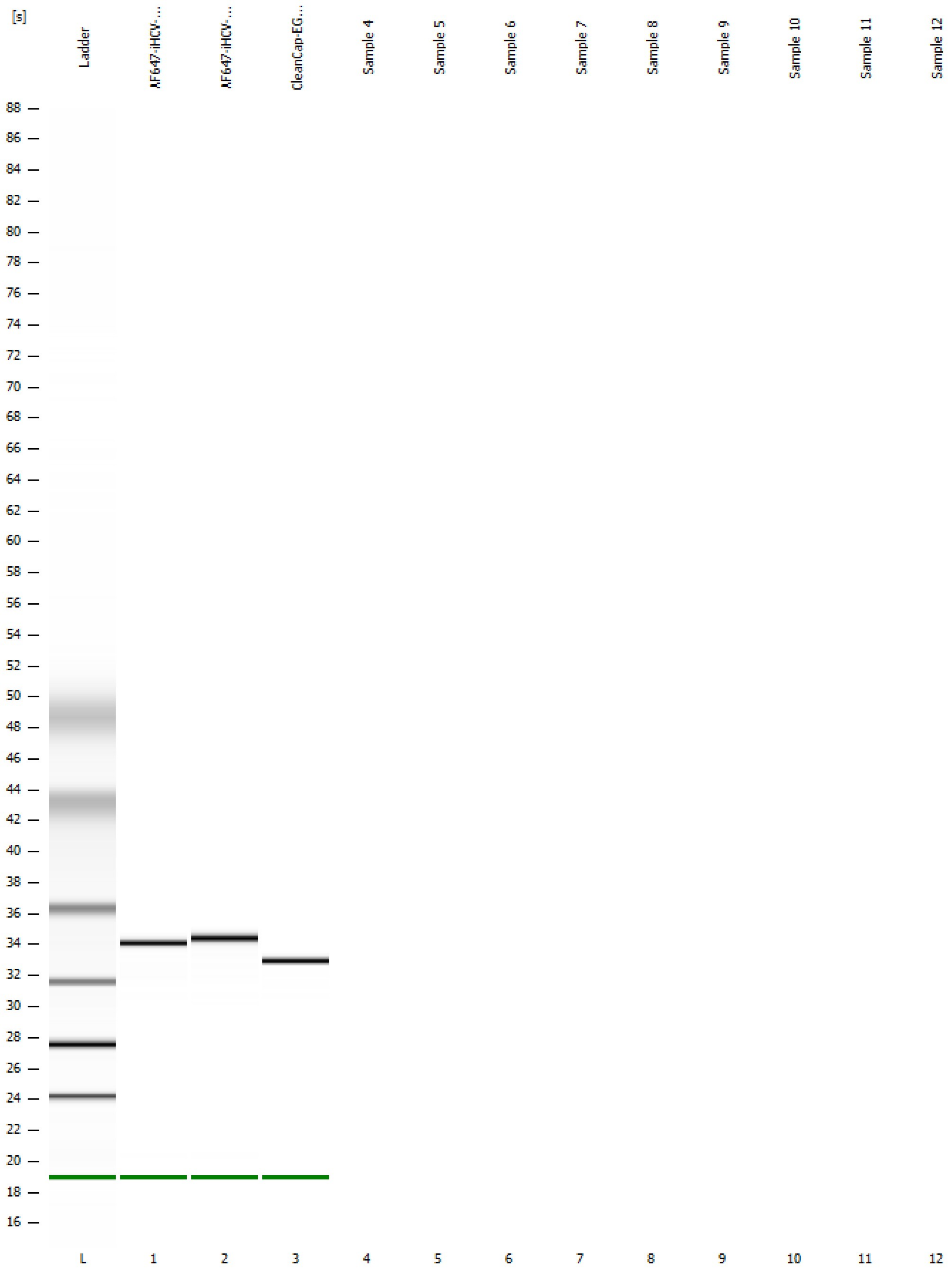


 © Copyright 2003 - 2021 Agilent, Inc. 

Assay Class: mRNA Nano
Data Path: C:\...pert\data\2024-09-10\2100_mRNA Nano_2024-09-10_16-38-44.xad
Created: 9/10/2024 4:38:43 pm
Modified: 12/20/2024 2:56:38 pm

**Invalid Samples**

Sample 4 has not been run, no results available.

Sample 5 has not been run, no results available.

Sample 6 has not been run, no results available.

Sample 7 has not been run, no results available.

Sample 8 has not been run, no results available.

Sample 9 has not been run, no results available.

Sample 10 has not been run, no results available.

Sample 11 has not been run, no results available.

Sample 12 has not been run, no results available.


© Copyright 2003 - 2021 Agilent, Inc.
