# Supplementary material for: Enhancing cap-independent translation of linear mRNA
Source: Nat Commun. 2025 Oct 16;16:9205. doi: 10.1038/s41467-025-64257-6 (PMC12532787; doi:10.1038/s41467-025-64257-6)
Supplement: Supplementary file 11 — Source Data [file 41467_2025_64257_MOESM11_ESM.zip › Source Data/Supplementary Figure 2/CleaN3 priming optimisation integration report 4 mM.pdf]

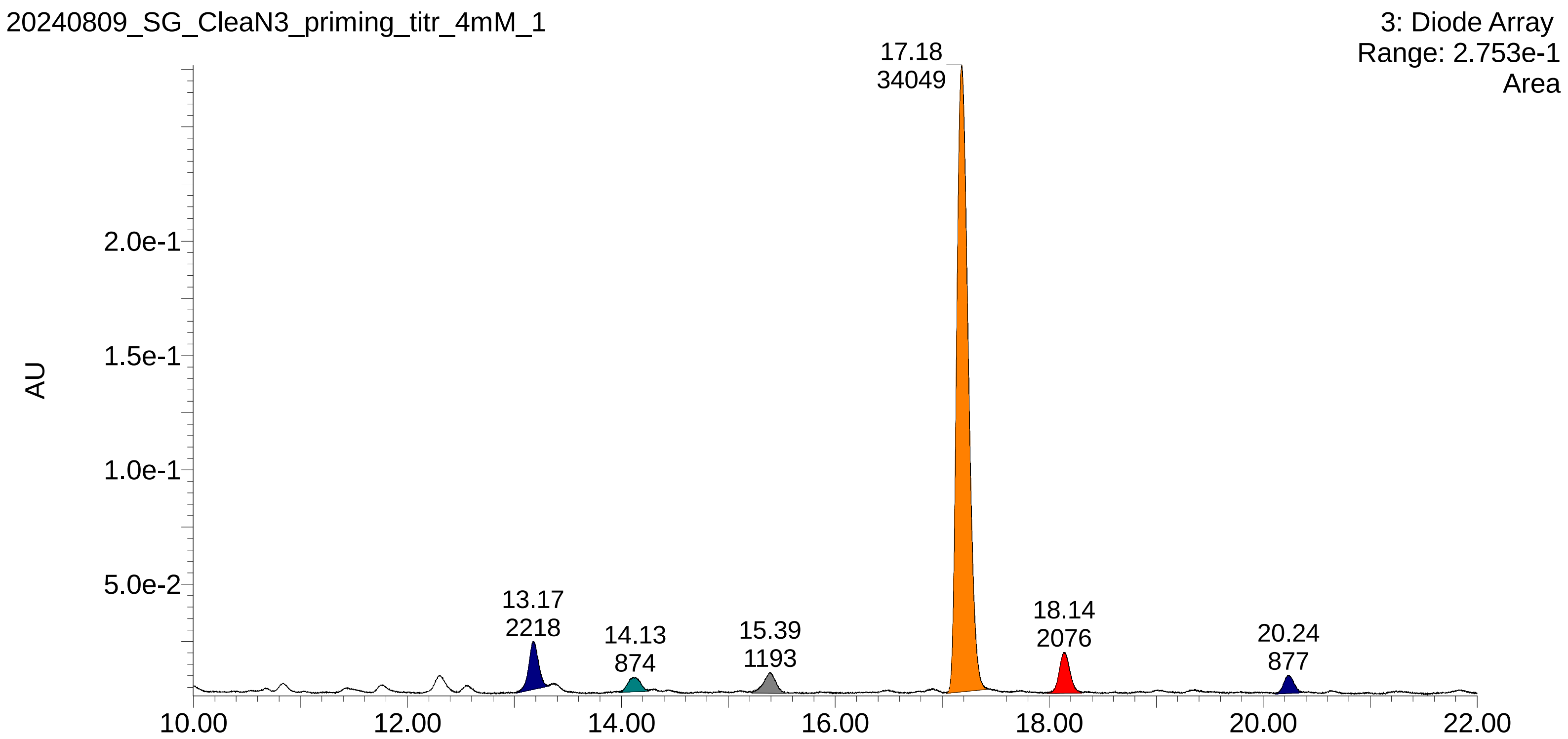
20240809_SG_CleaN3_priming_titr_4mM_1
3: Diode Array
Range: 2.753e-1
Area
AU
2.0e-1
1.5e-1
1.0e-1
5.0e-2
10.00
12.00
14.00
16.00
18.00
20.00
22.00
13.17
2218
14.13
874
15.39
1193
17.18
34049
18.14
2076
20.24
877

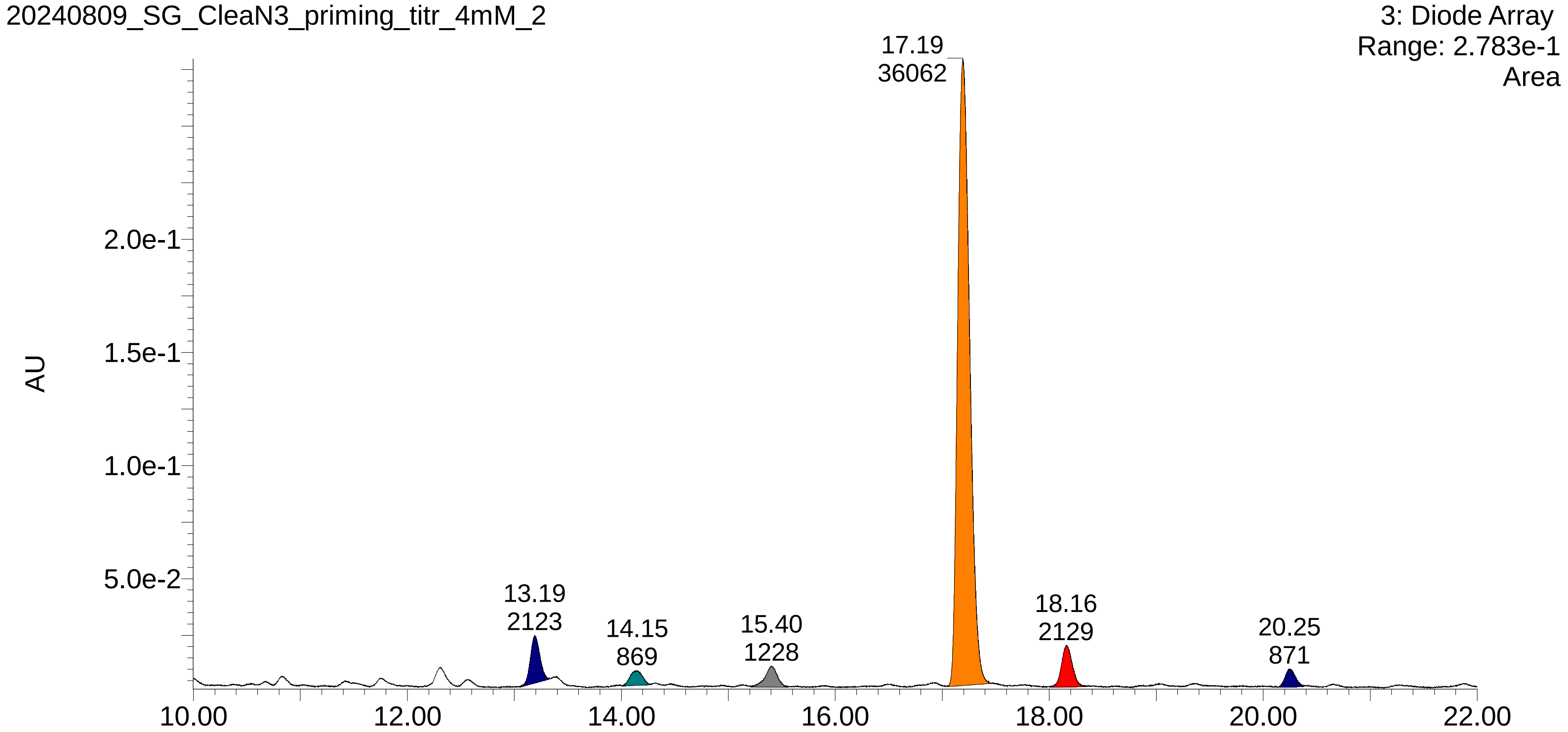
20240809_SG_CleaN3_priming_titr_4mM_2
3: Diode Array
Range: 2.783e-1
Area
AU
2.0e-1
1.5e-1
1.0e-1
5.0e-2
10.00
12.00
14.00
16.00
18.00
20.00
22.00
13.19
2123
14.15
869
15.40
1228
17.19
36062
18.16
2129
20.25
871

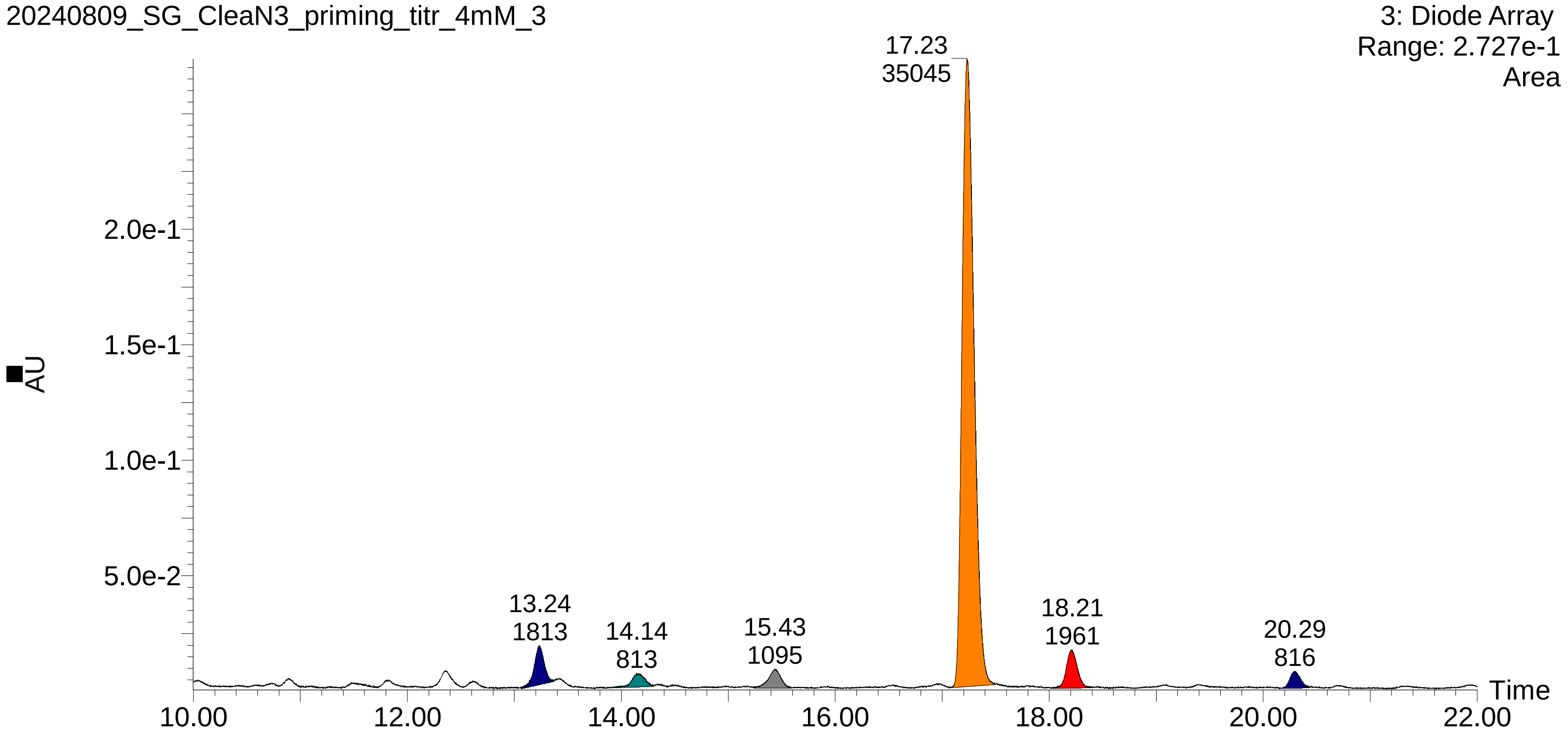
20240809_SG_CleaN3_priming_titr_4mM_3
3: Diode Array
Range: 2.727e-1
Area
AU
2.0e-1
1.5e-1
1.0e-1
5.0e-2
10.00
12.00
14.00
16.00
18.00
20.00
22.00
Time
13.24
1813
14.14
813
15.43
1095
17.23
35045
18.21
1961
20.29
816
