# Supplementary figures and images for: Enhancing cap-independent translation of linear mRNA
Source: Nat Commun. 2025 Oct 16;16:9205. doi: 10.1038/s41467-025-64257-6 (PMC12532787; doi:10.1038/s41467-025-64257-6)

20231024\_blankx111

3: Diode Array  
Range: 9.606e-2

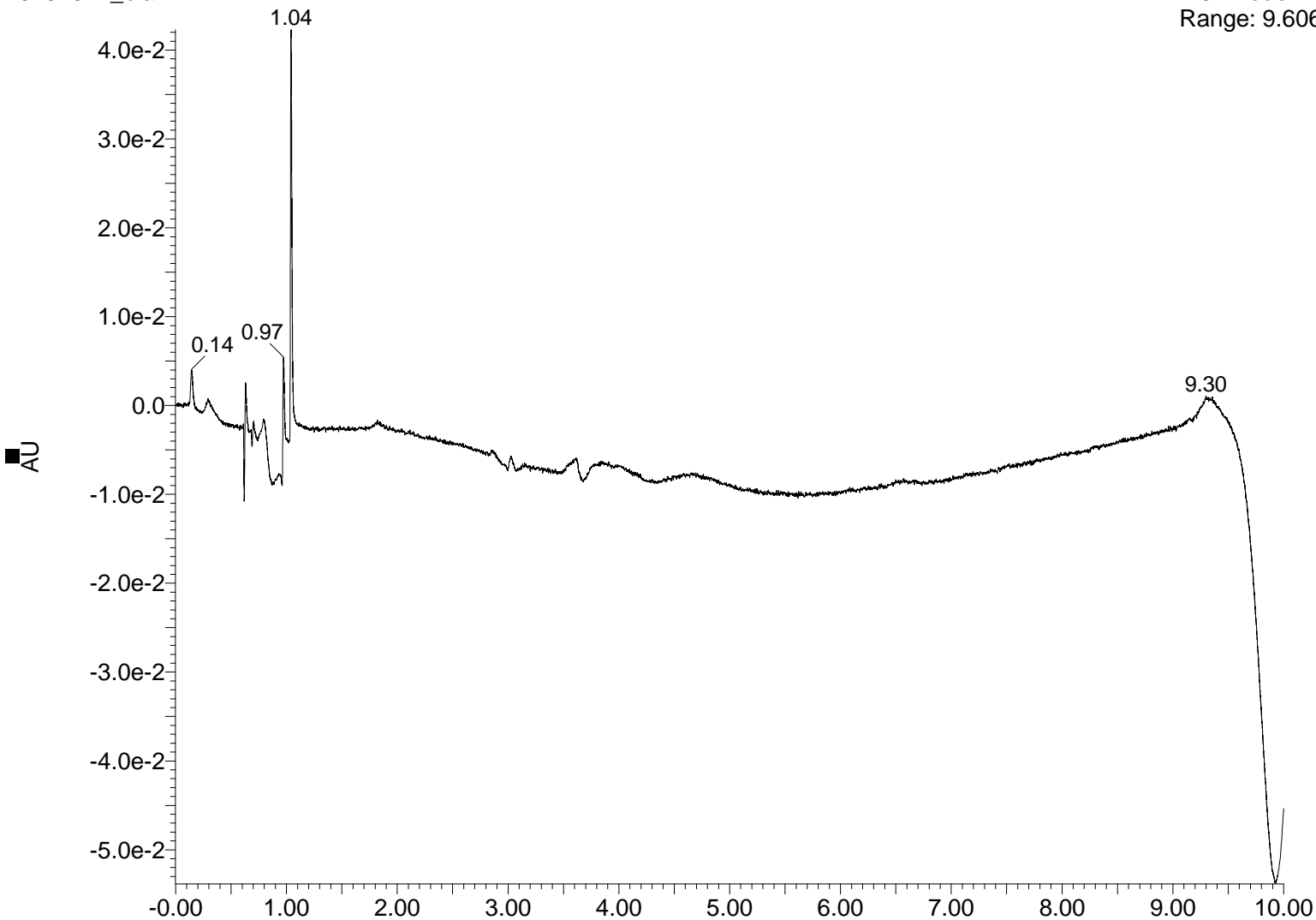

20231024\_SG\_azido-primer\_1\_8ul

3: Diode Array  
Range: 8.28e-1  
Area

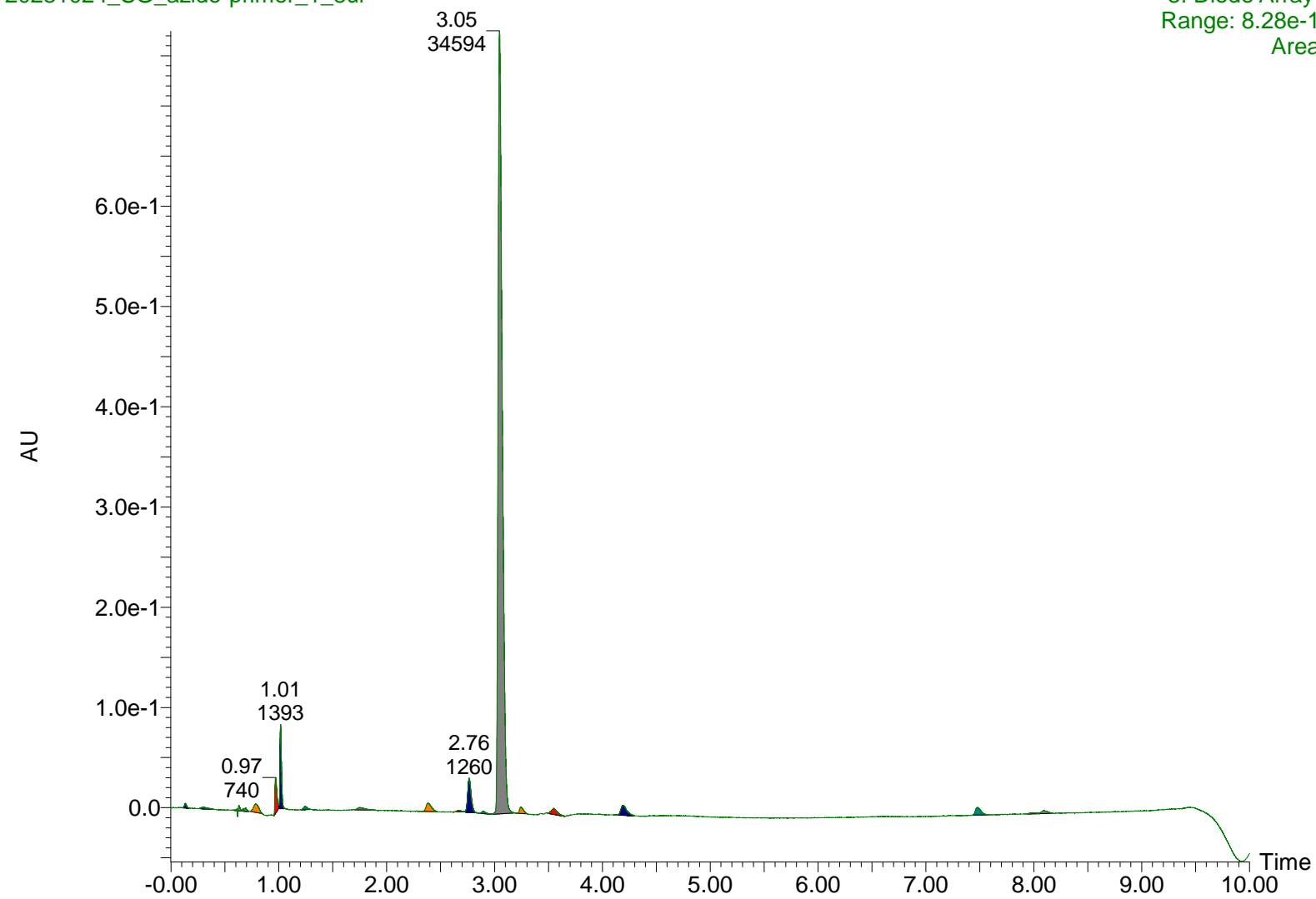

Supplement: Supplementary file 11 — Source Data [file 41467_2025_64257_MOESM11_ESM.zip › Source Data/Figure 2/CleaN3 crude analysis integration results.pdf]

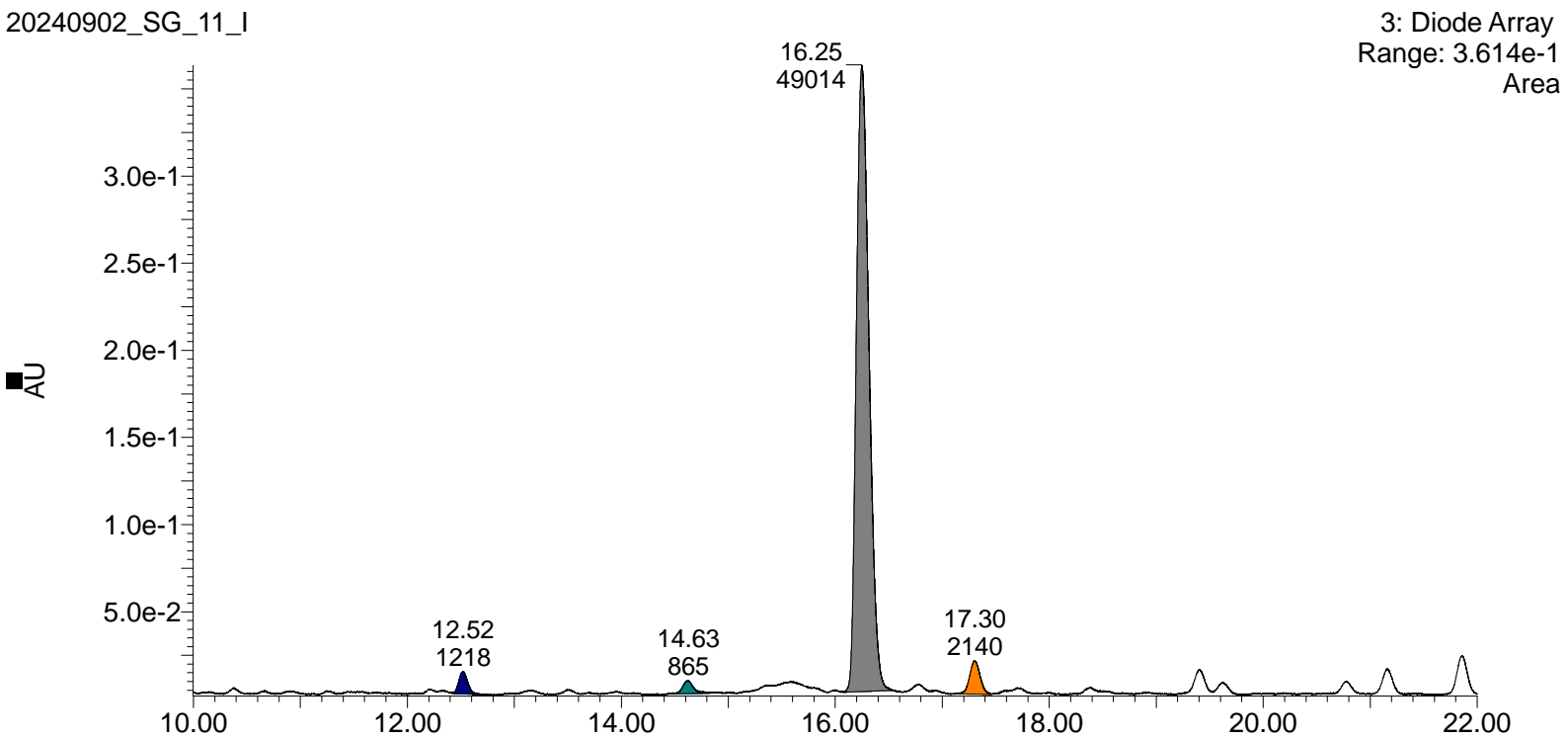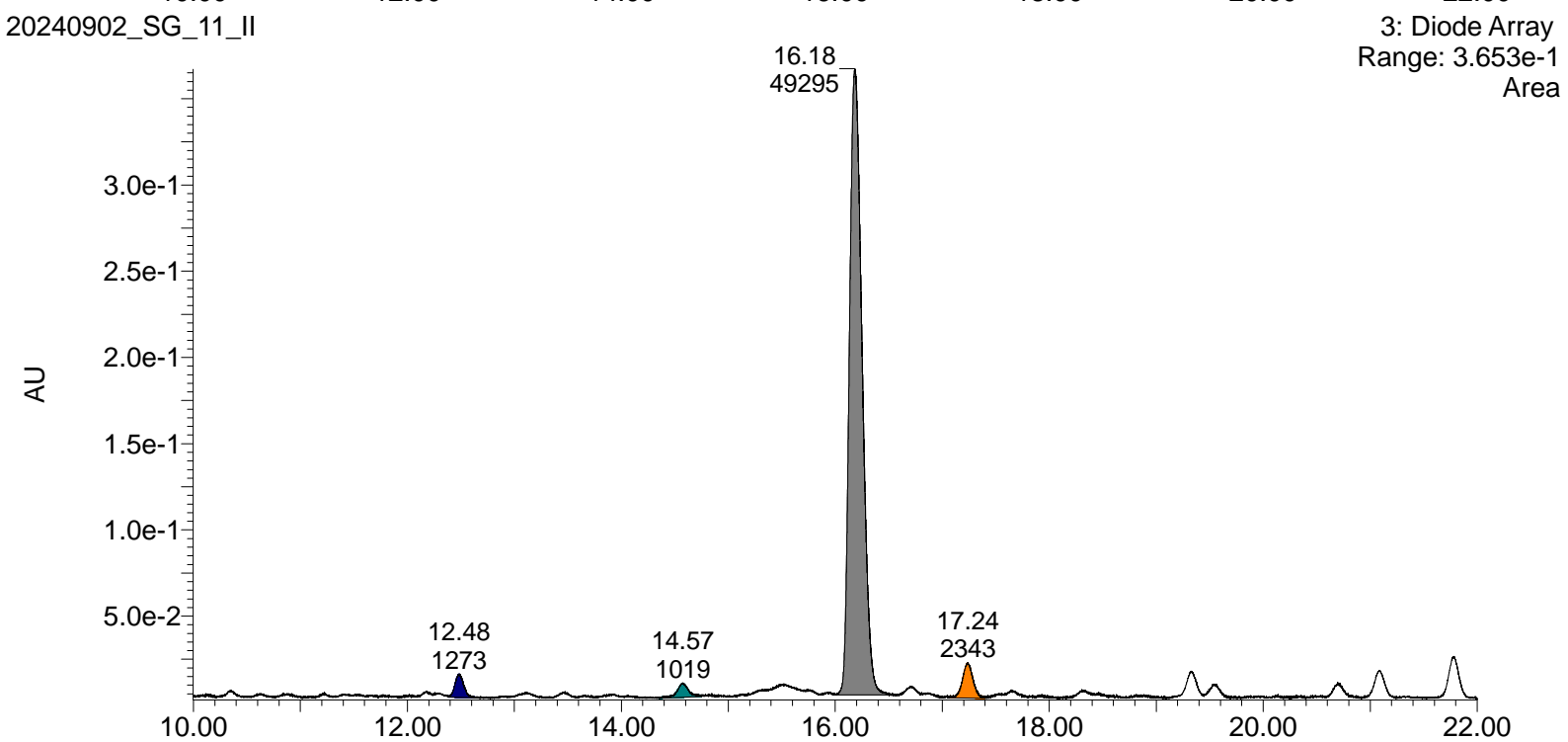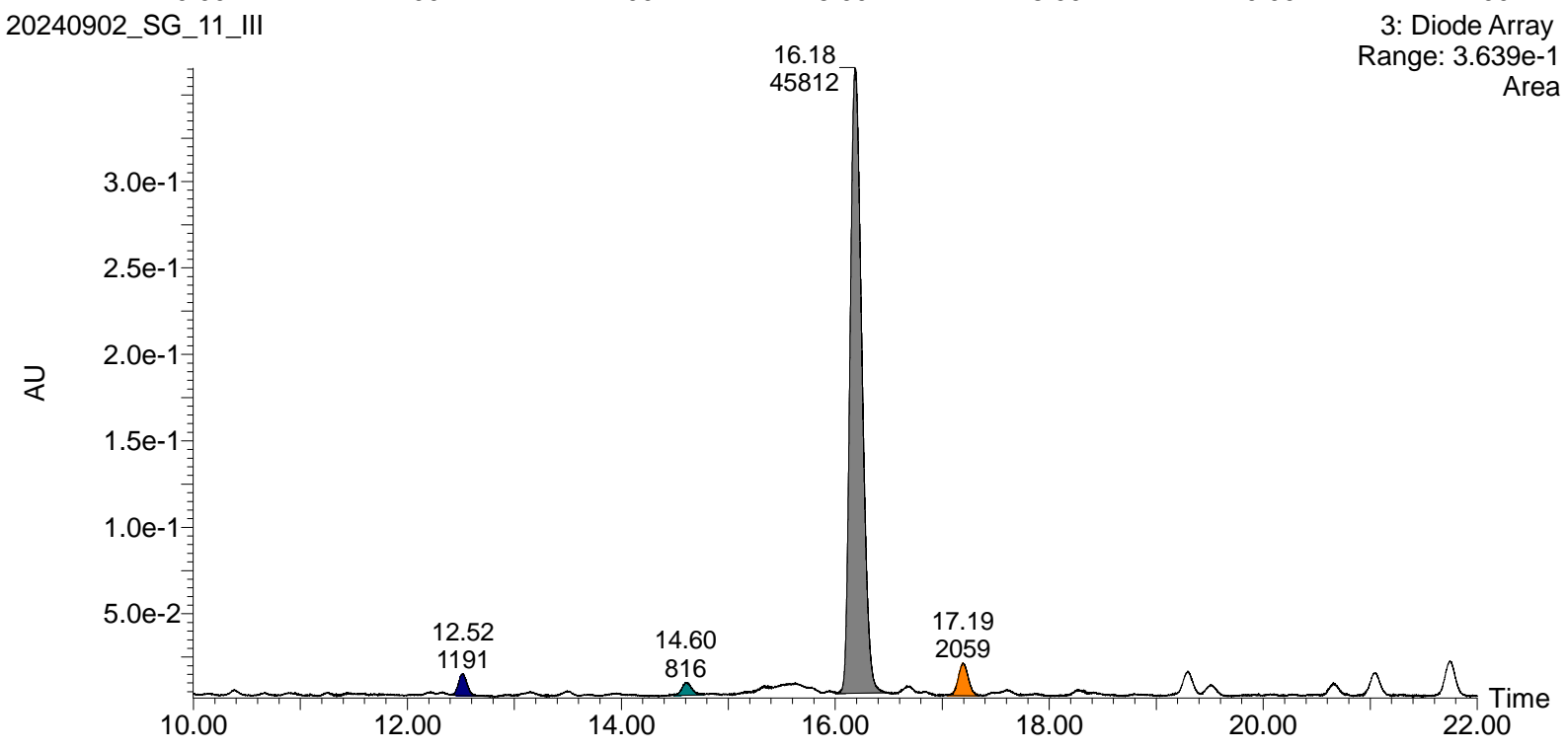

Supplement: Supplementary file 11 — Source Data [file 41467_2025_64257_MOESM11_ESM.zip › Source Data/Figure 3/tCLuc A-ins 6-5 CleaN3.pdf]

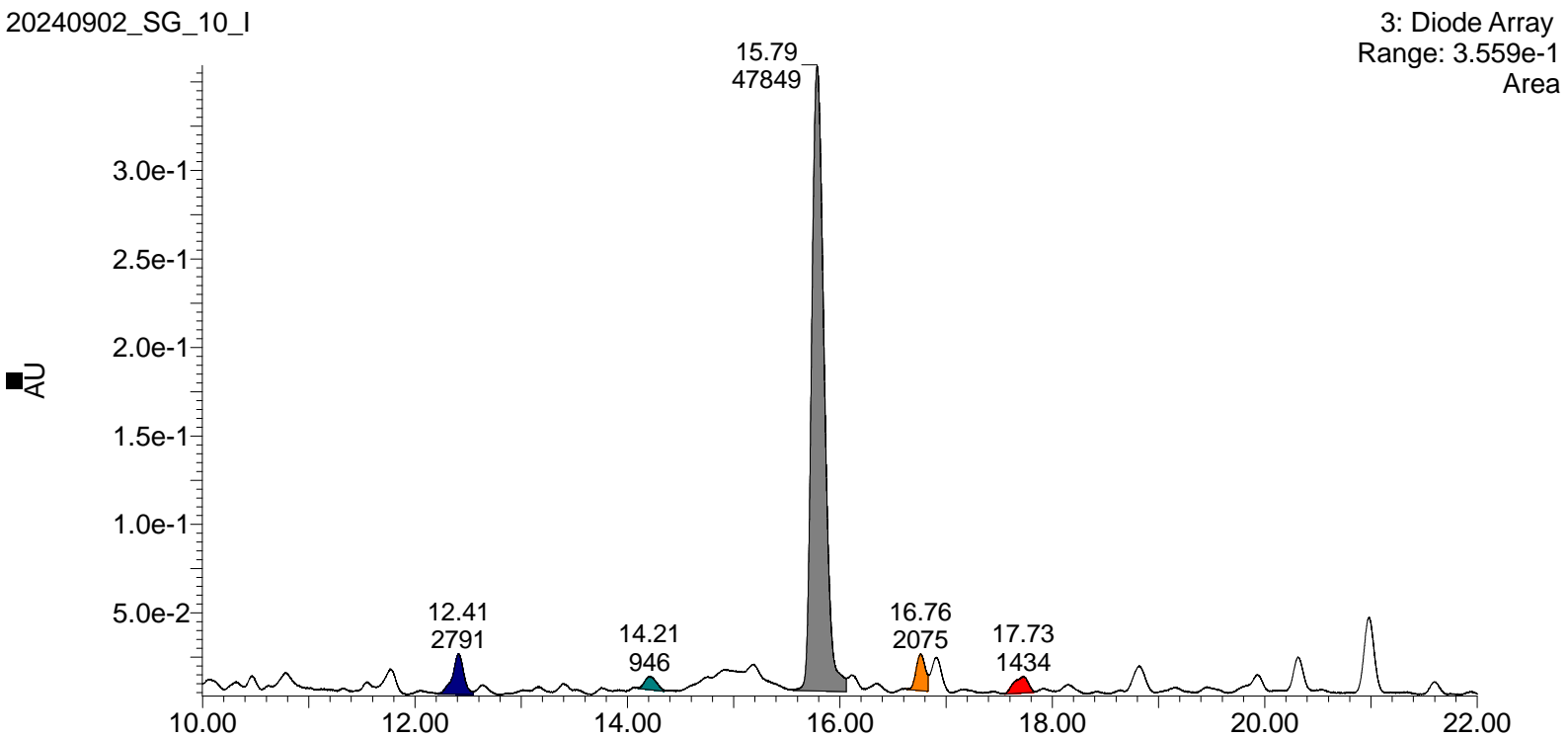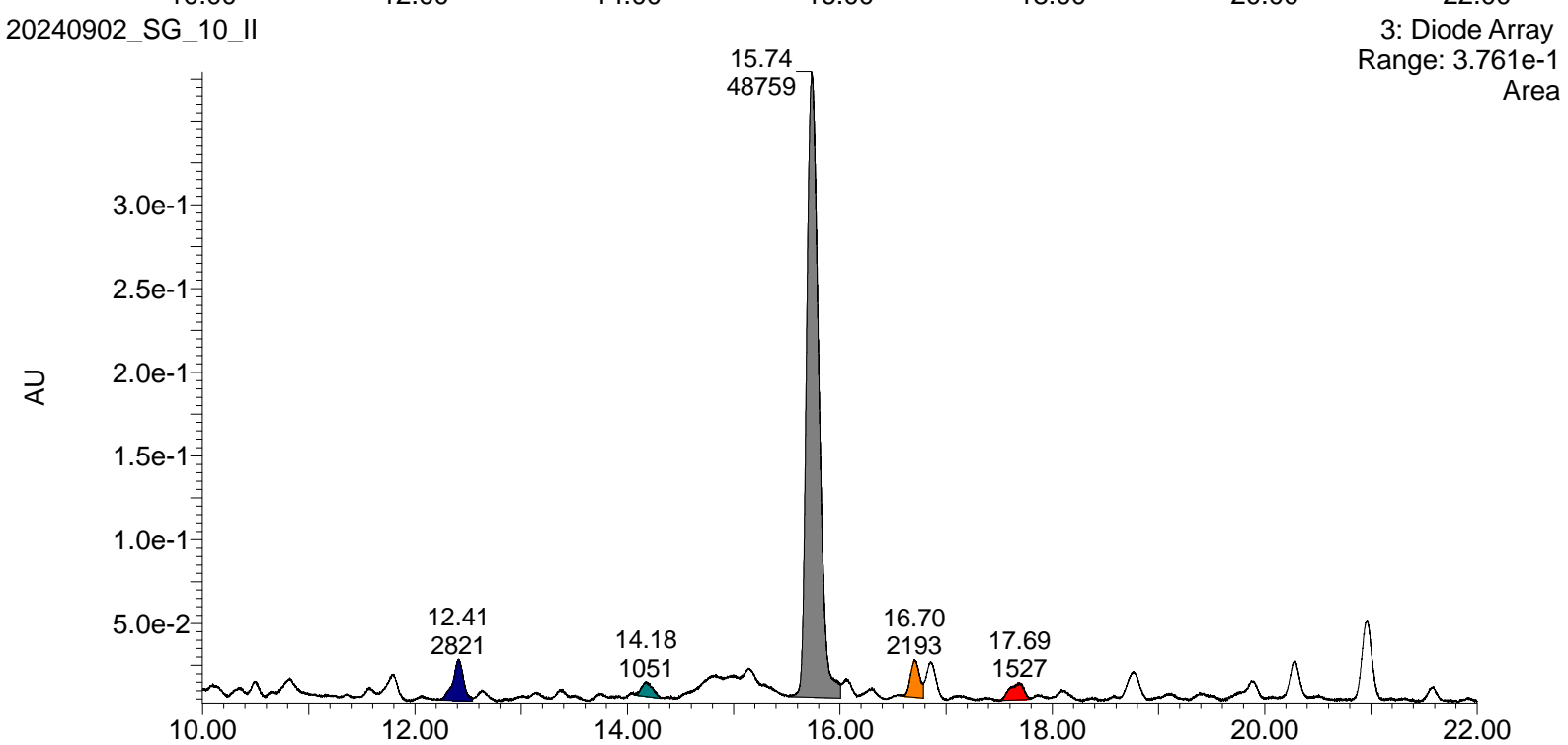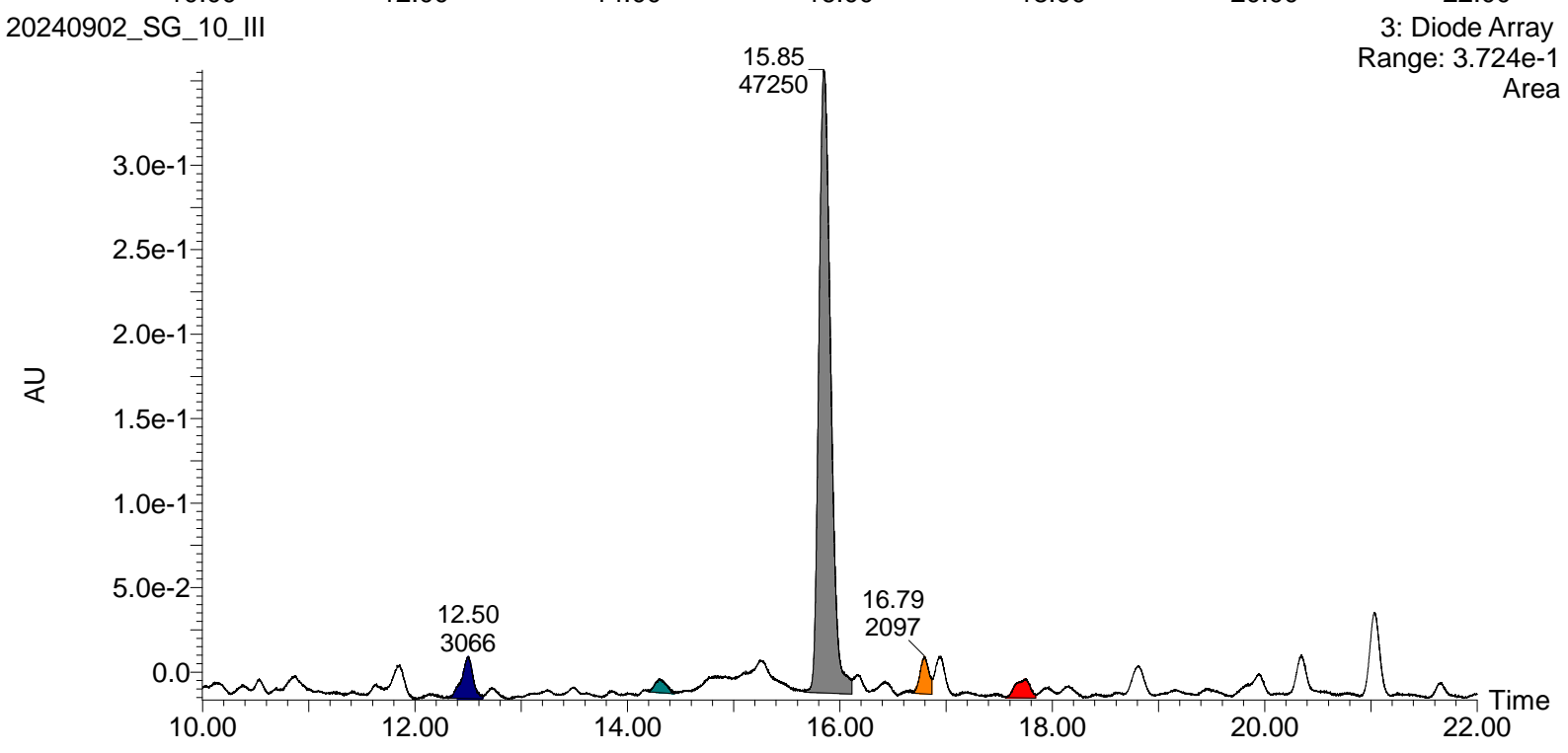

Supplement: Supplementary file 11 — Source Data [file 41467_2025_64257_MOESM11_ESM.zip › Source Data/Figure 3/tCLuc A-ins 6-5 CleanCap.pdf]

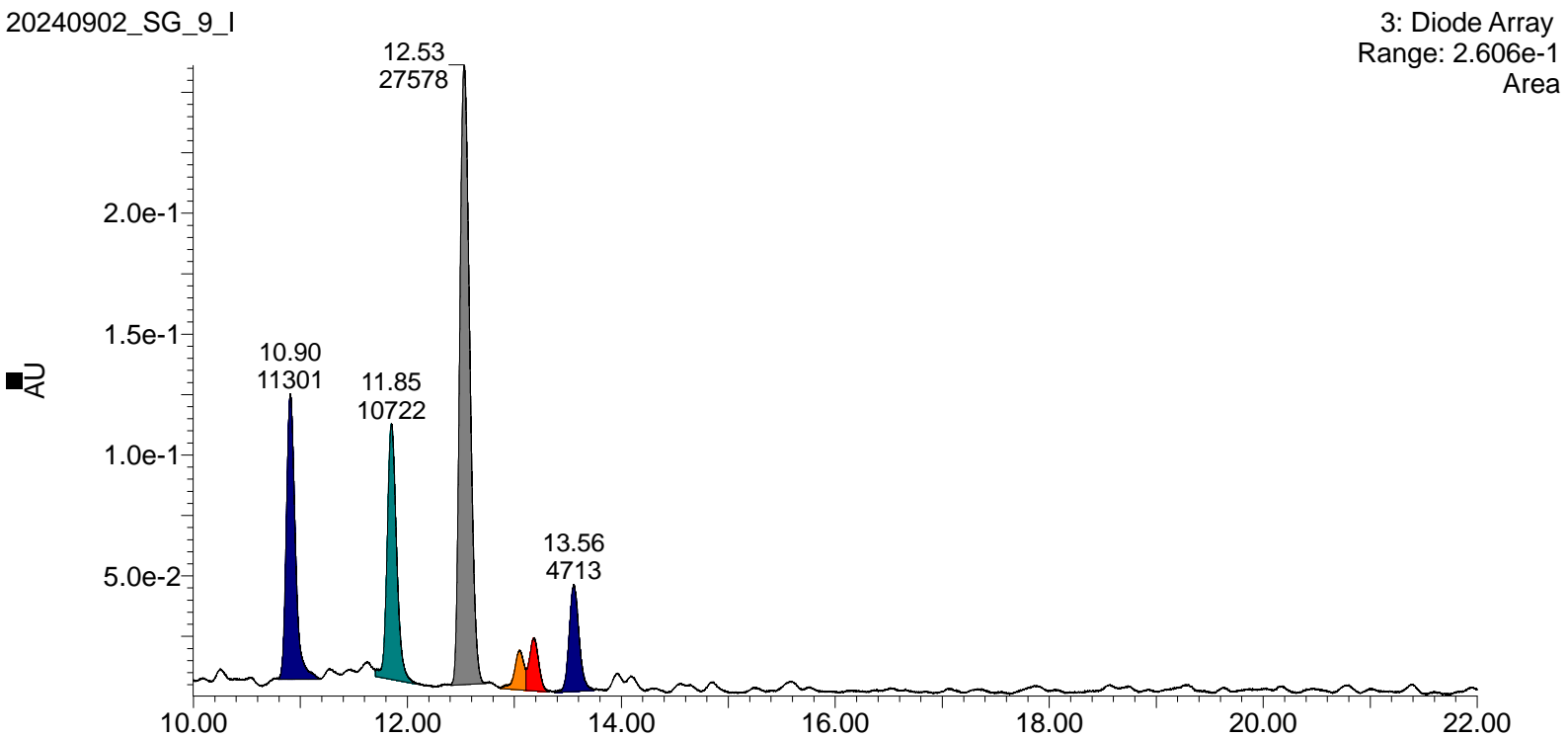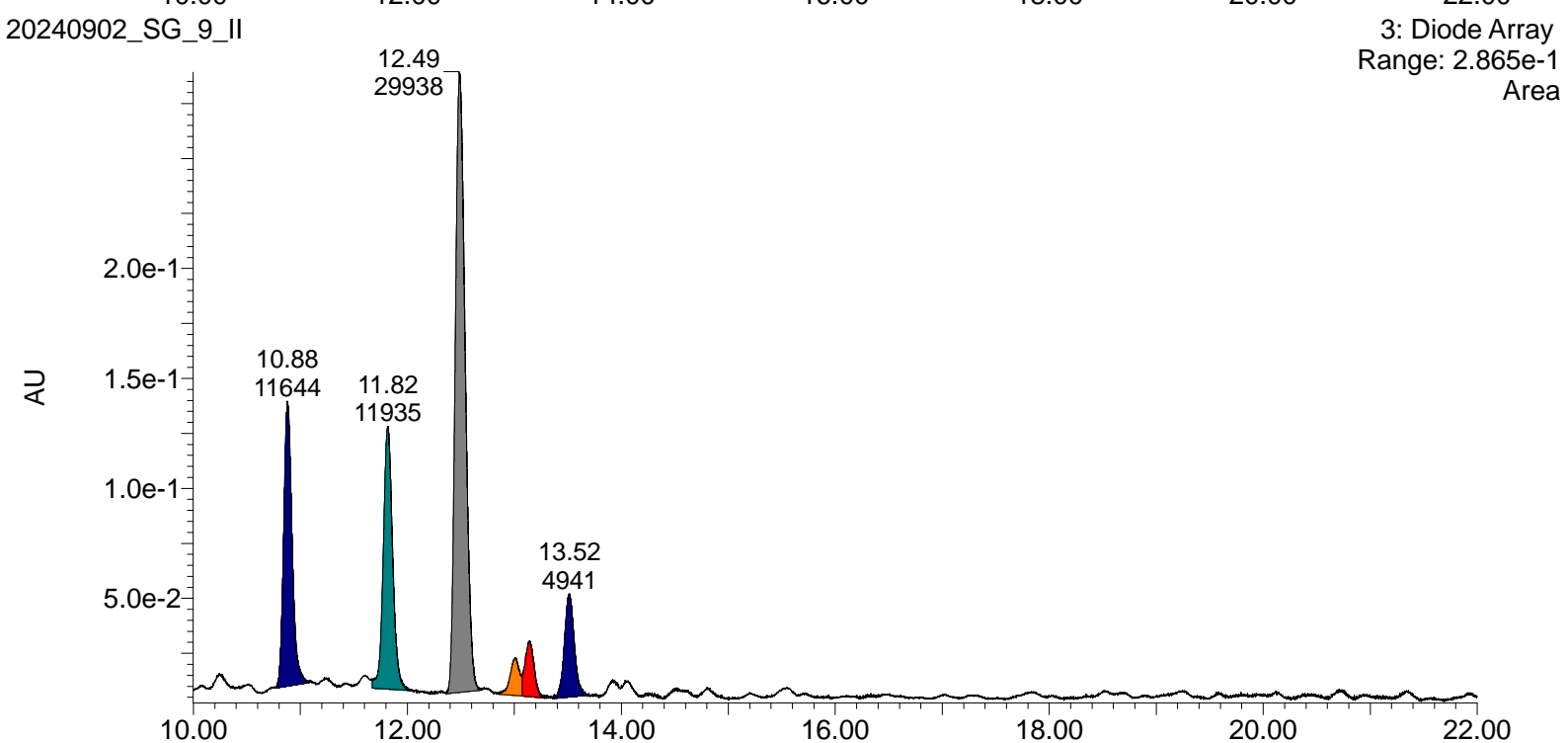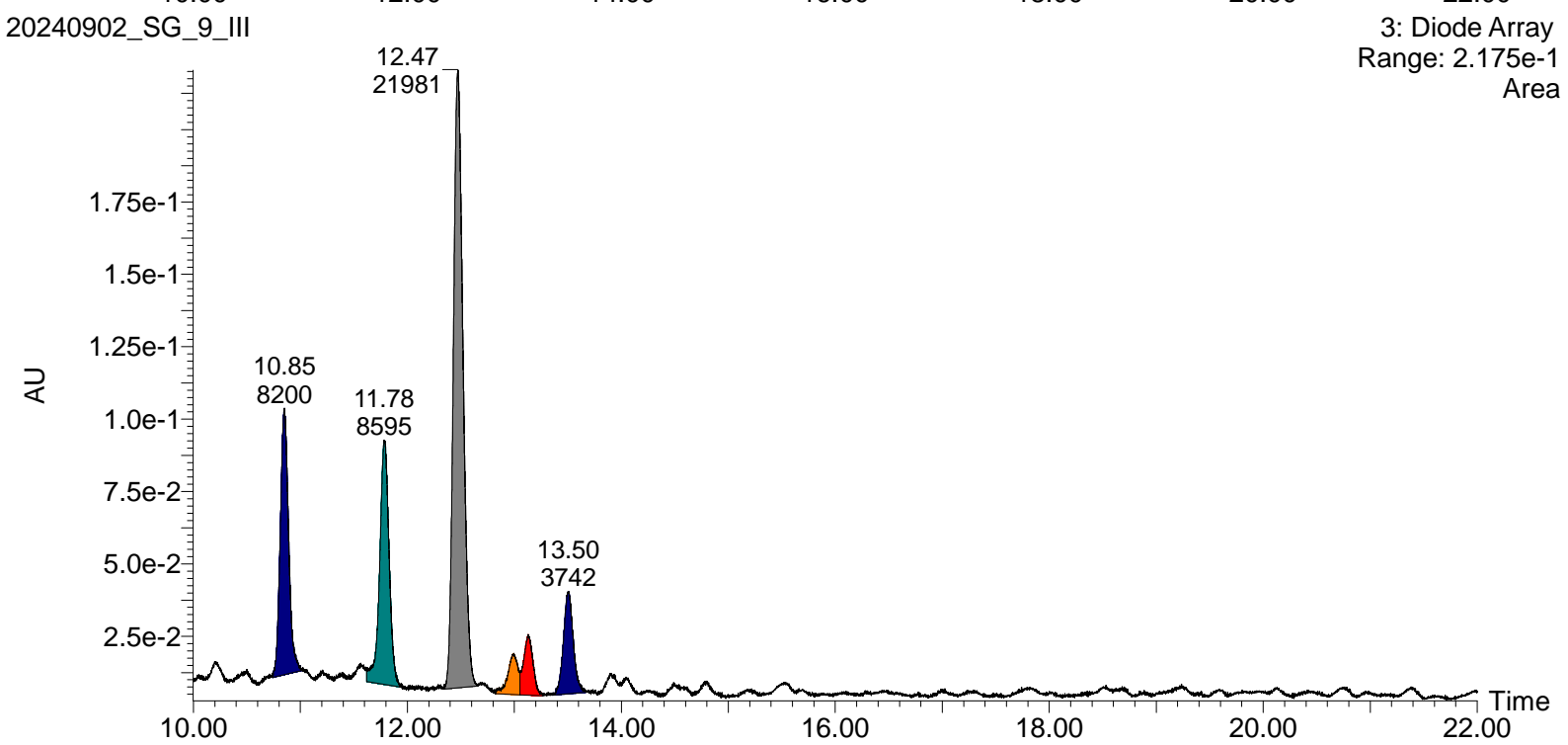

Supplement: Supplementary file 11 — Source Data [file 41467_2025_64257_MOESM11_ESM.zip › Source Data/Figure 3/tCLuc A-ins 6-5 non-primed.pdf]

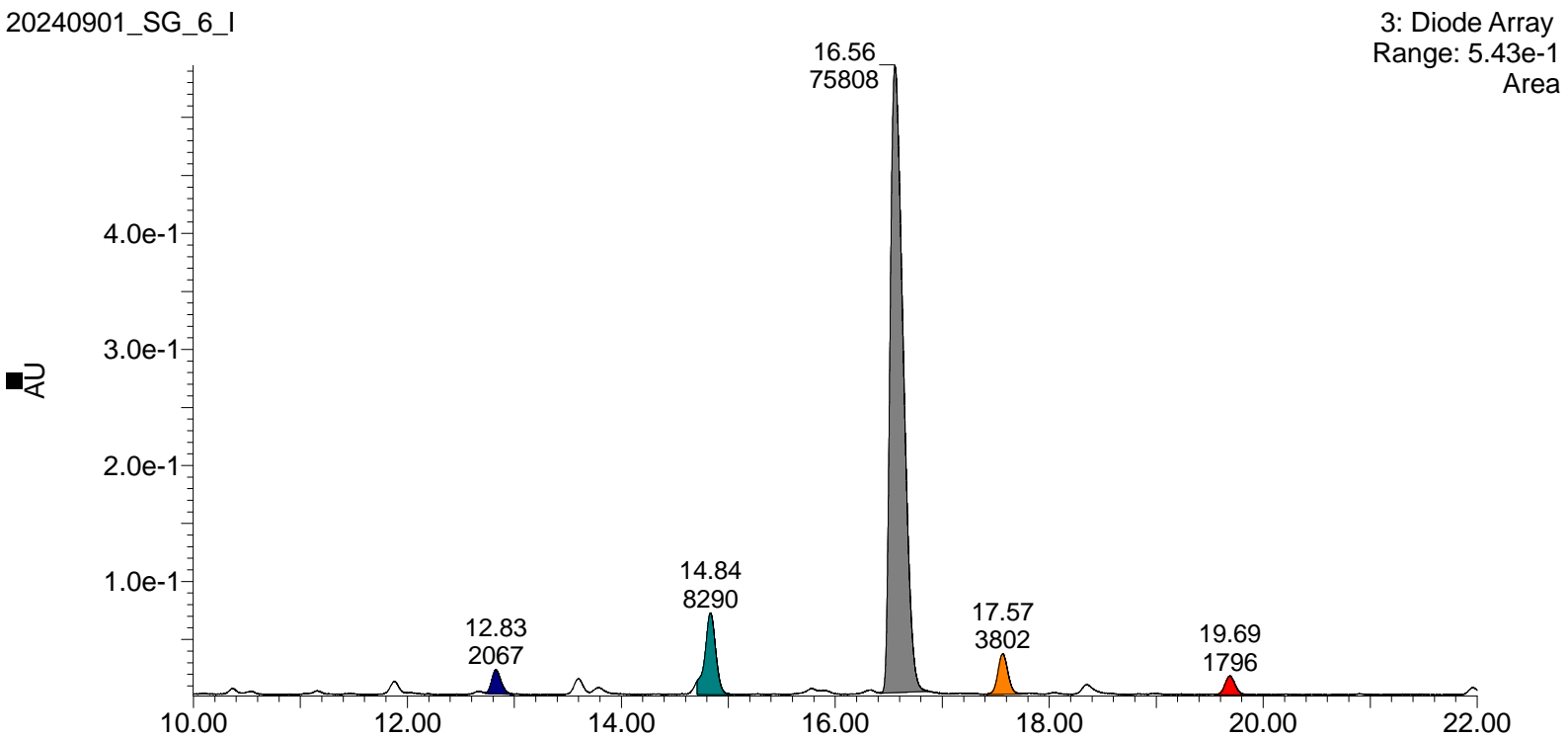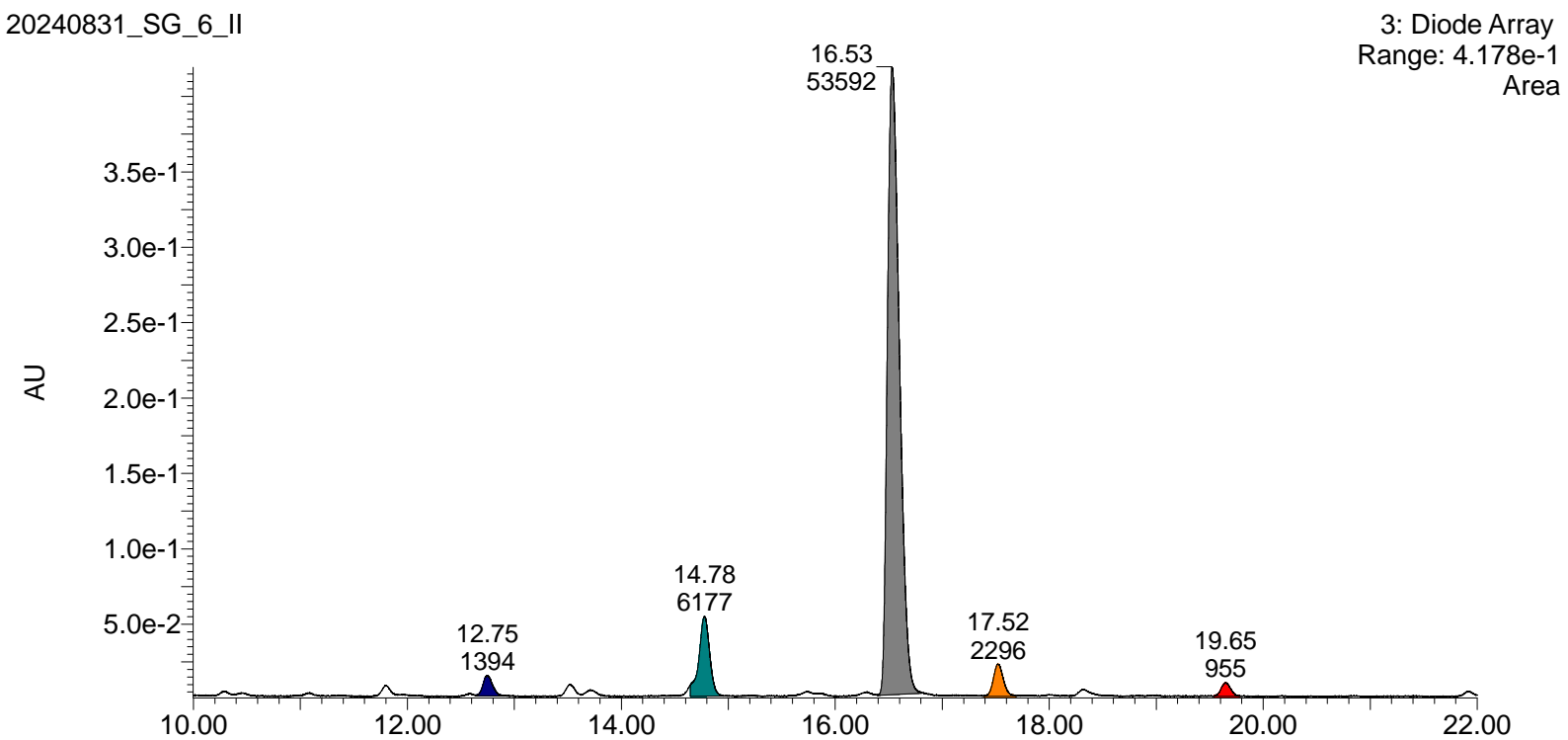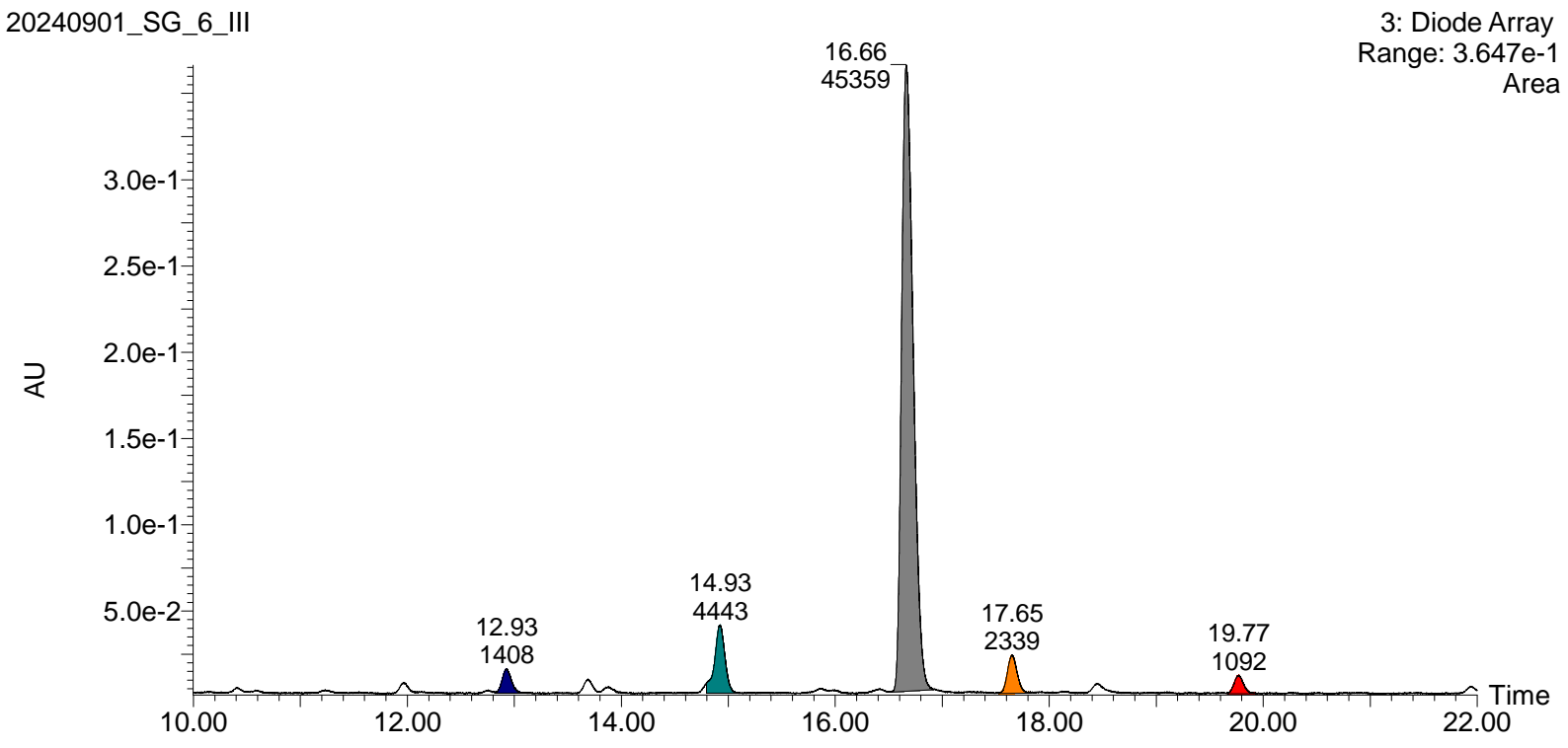

Supplement: Supplementary file 11 — Source Data [file 41467_2025_64257_MOESM11_ESM.zip › Source Data/Figure 3/tiEMCV-Cas9 A-ins 6-5 CleaN3.pdf]

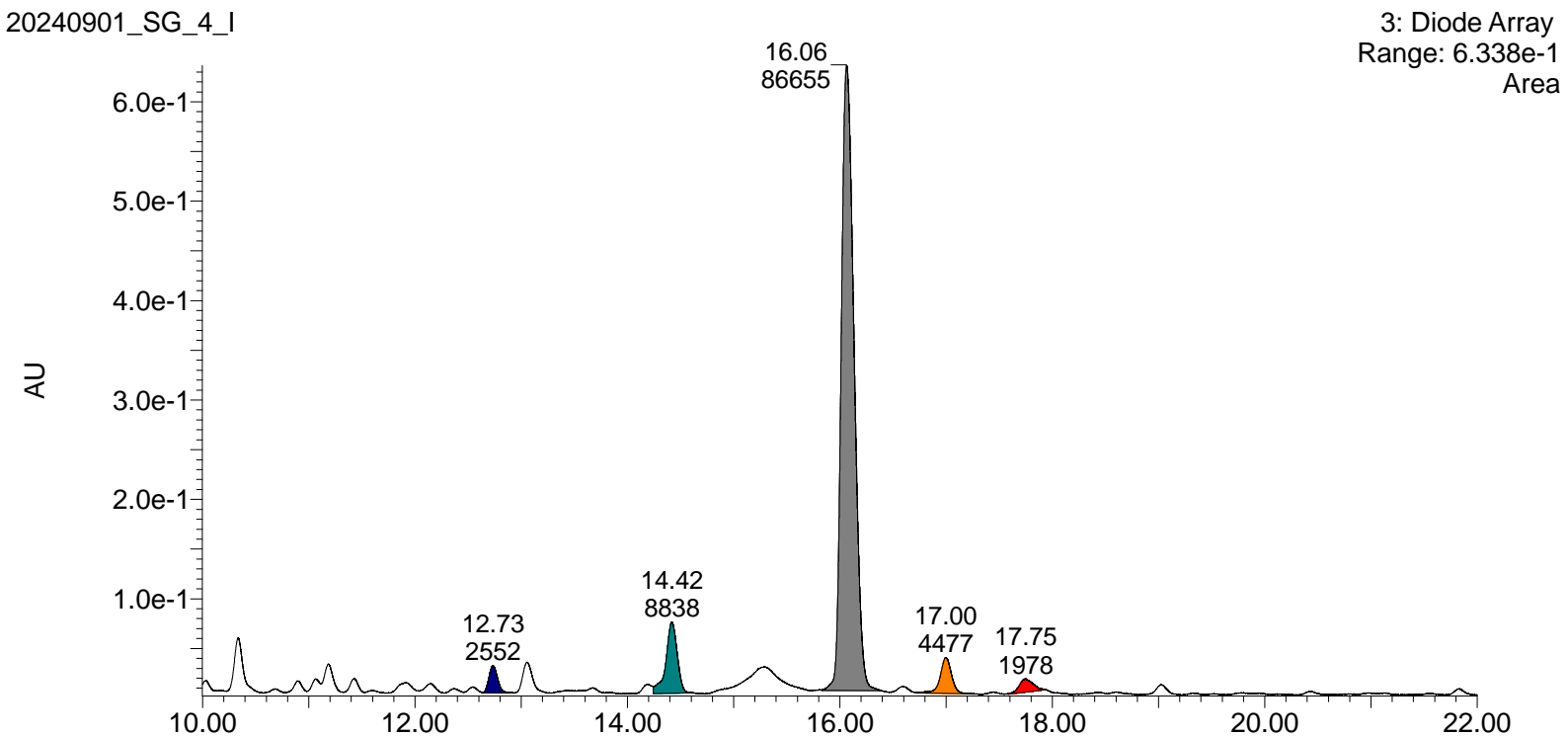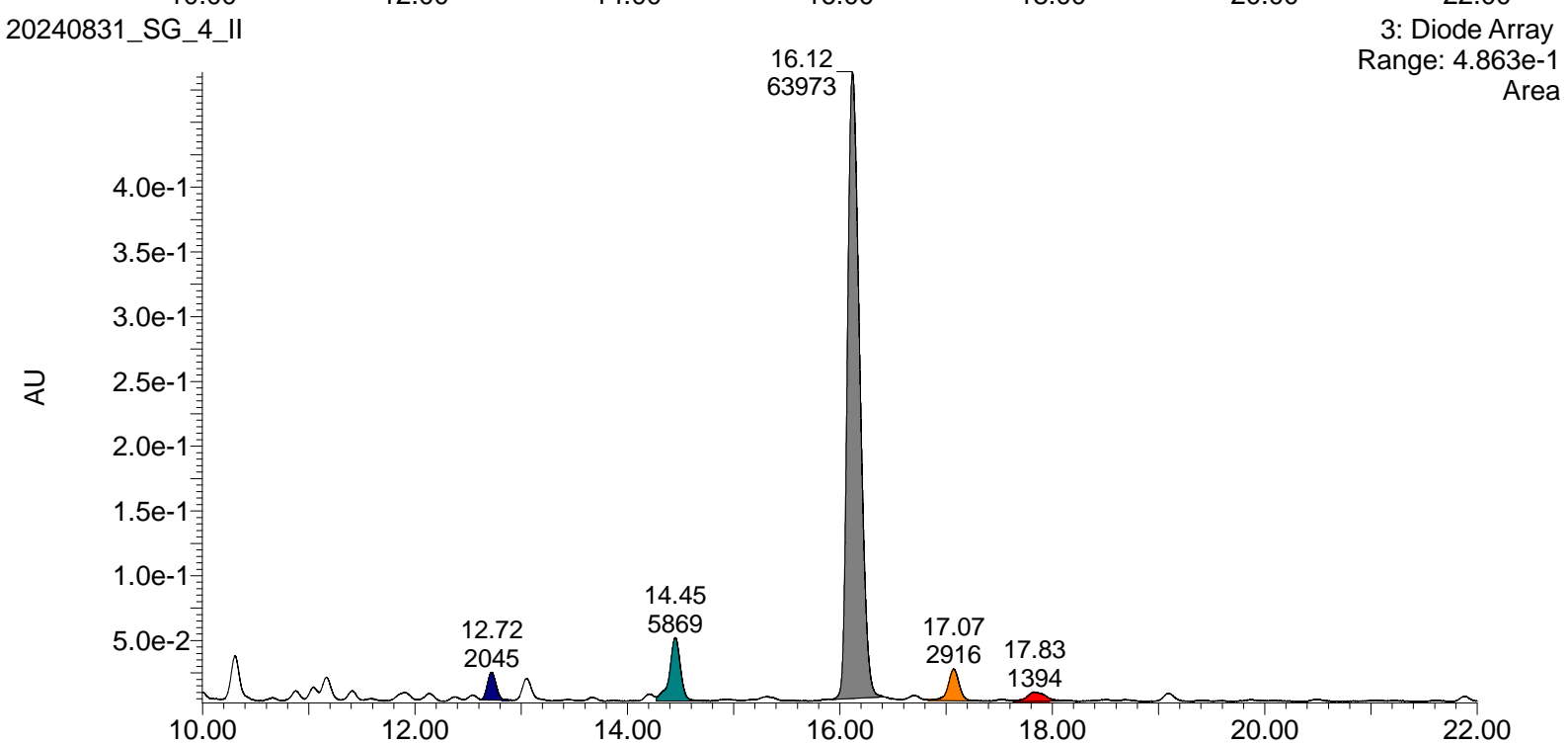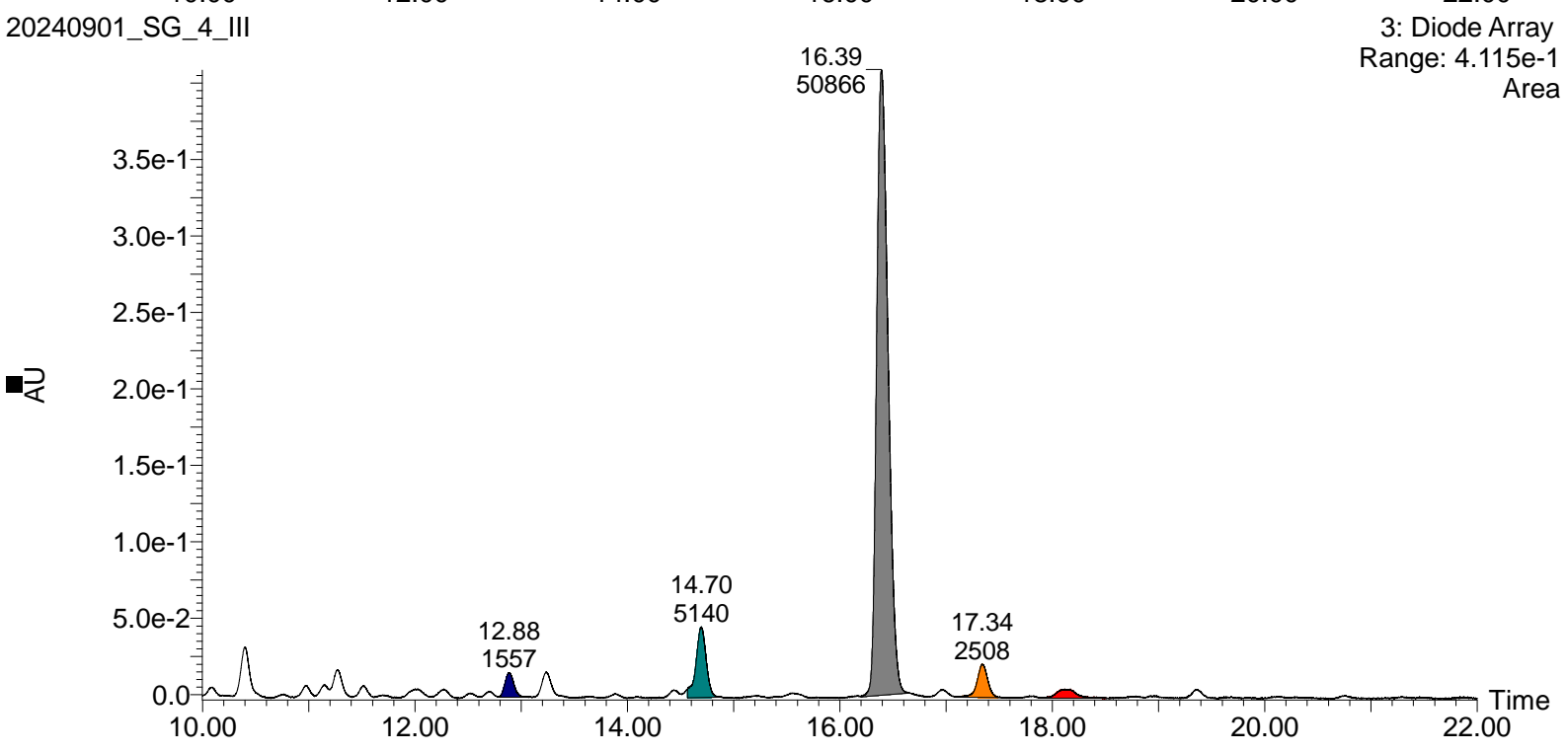

Supplement: Supplementary file 11 — Source Data [file 41467_2025_64257_MOESM11_ESM.zip › Source Data/Figure 3/tiEMCV-Cas9 A-ins 6-5 CleanCap.pdf]

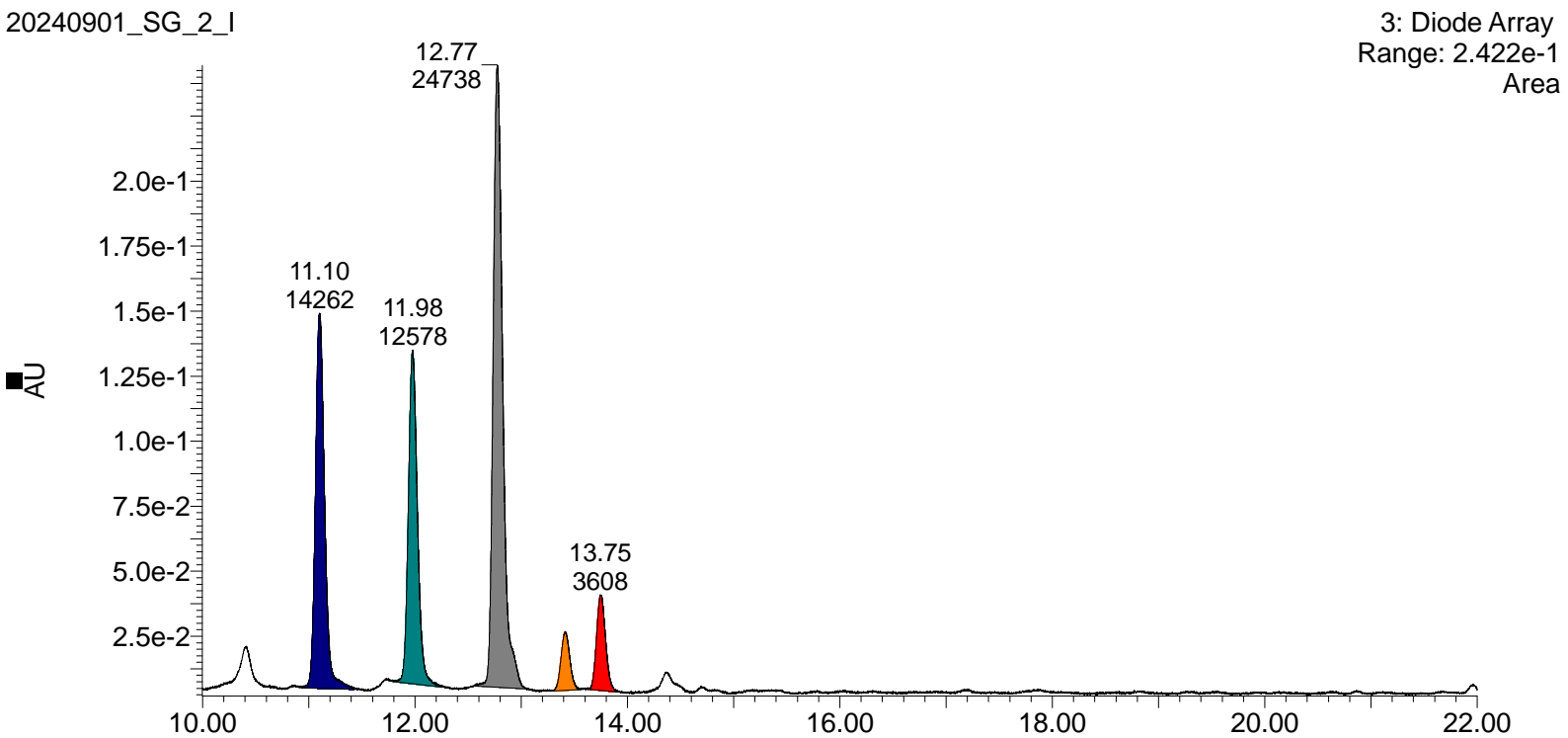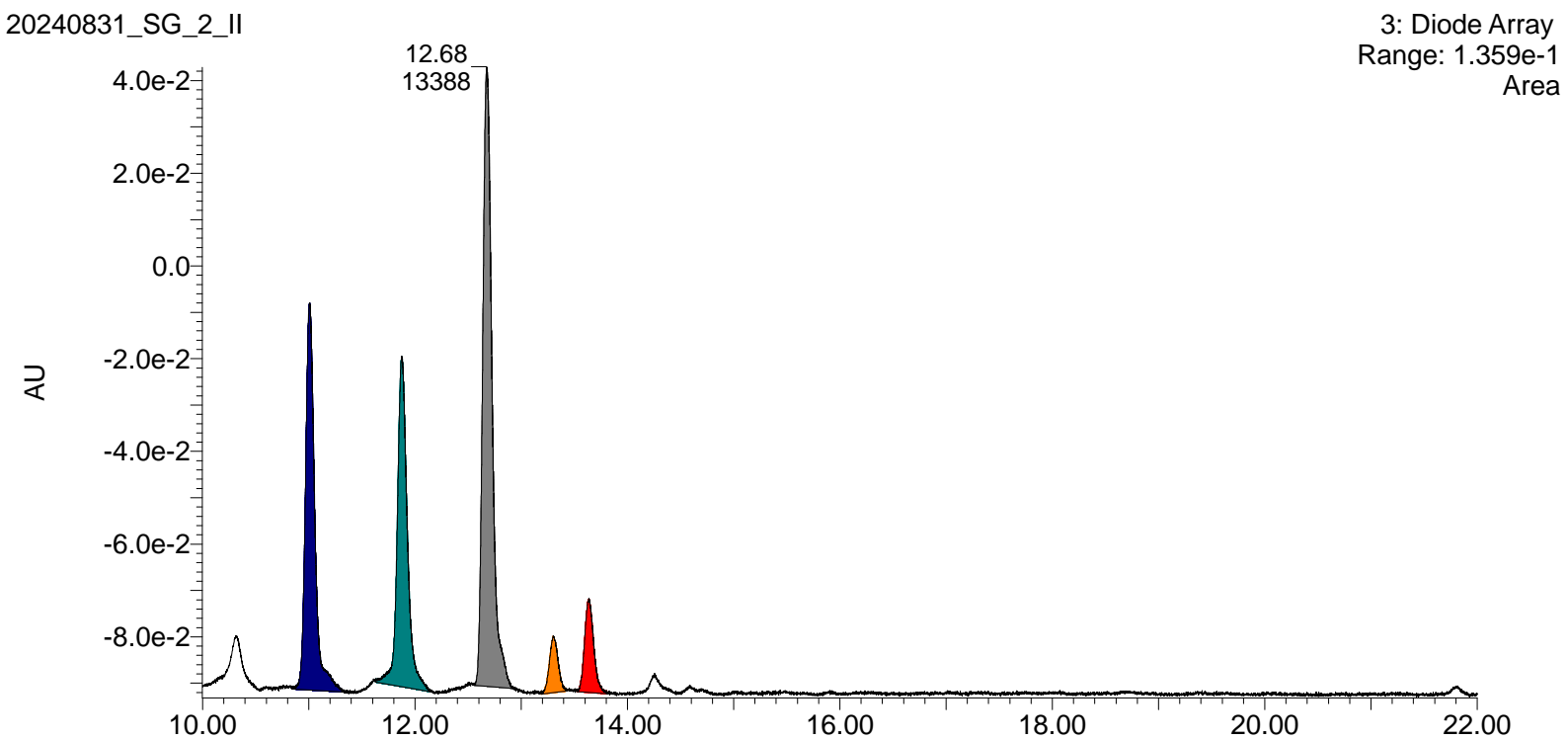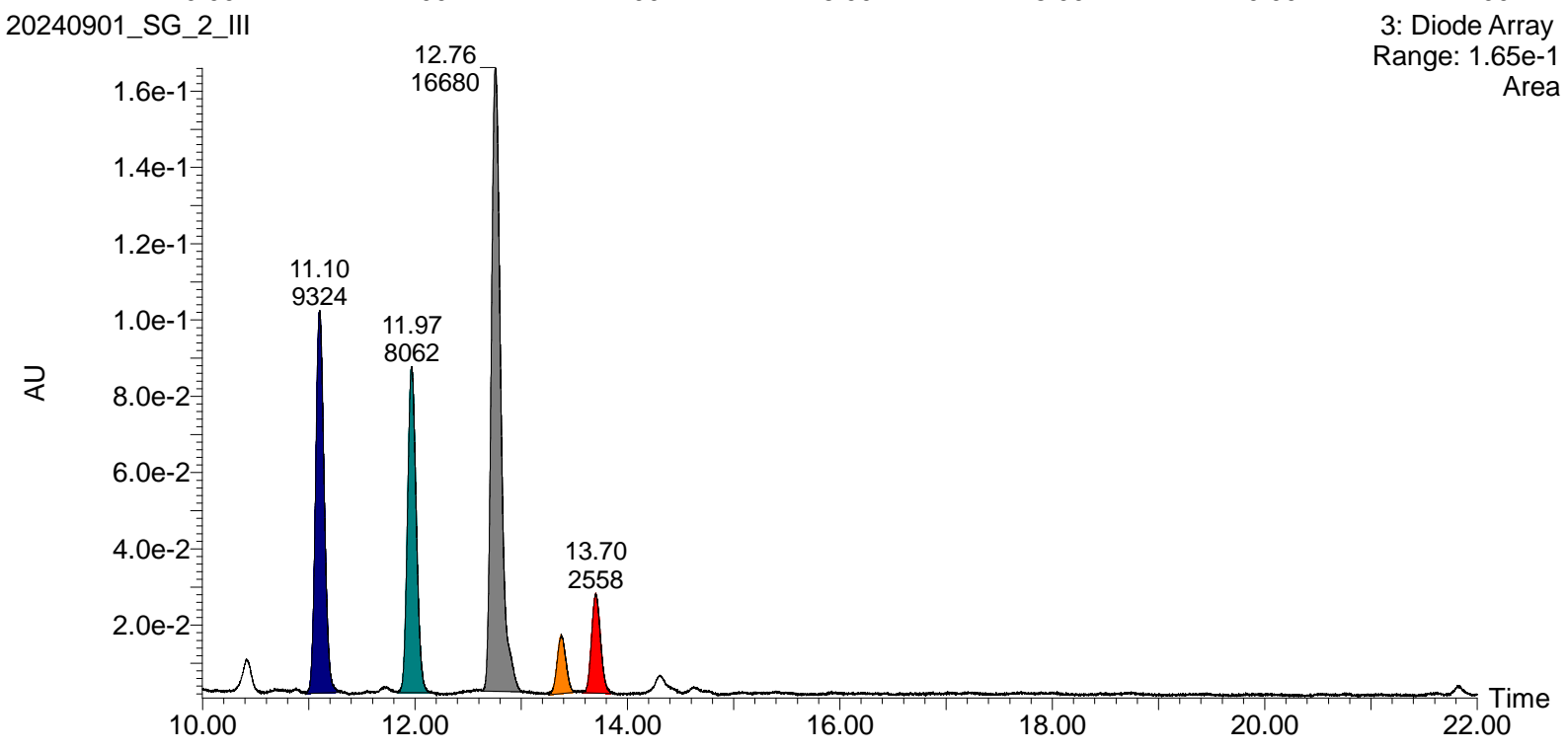

Supplement: Supplementary file 11 — Source Data [file 41467_2025_64257_MOESM11_ESM.zip › Source Data/Figure 3/tiEMCV-Cas9 A-ins 6-5 non-primed.pdf]

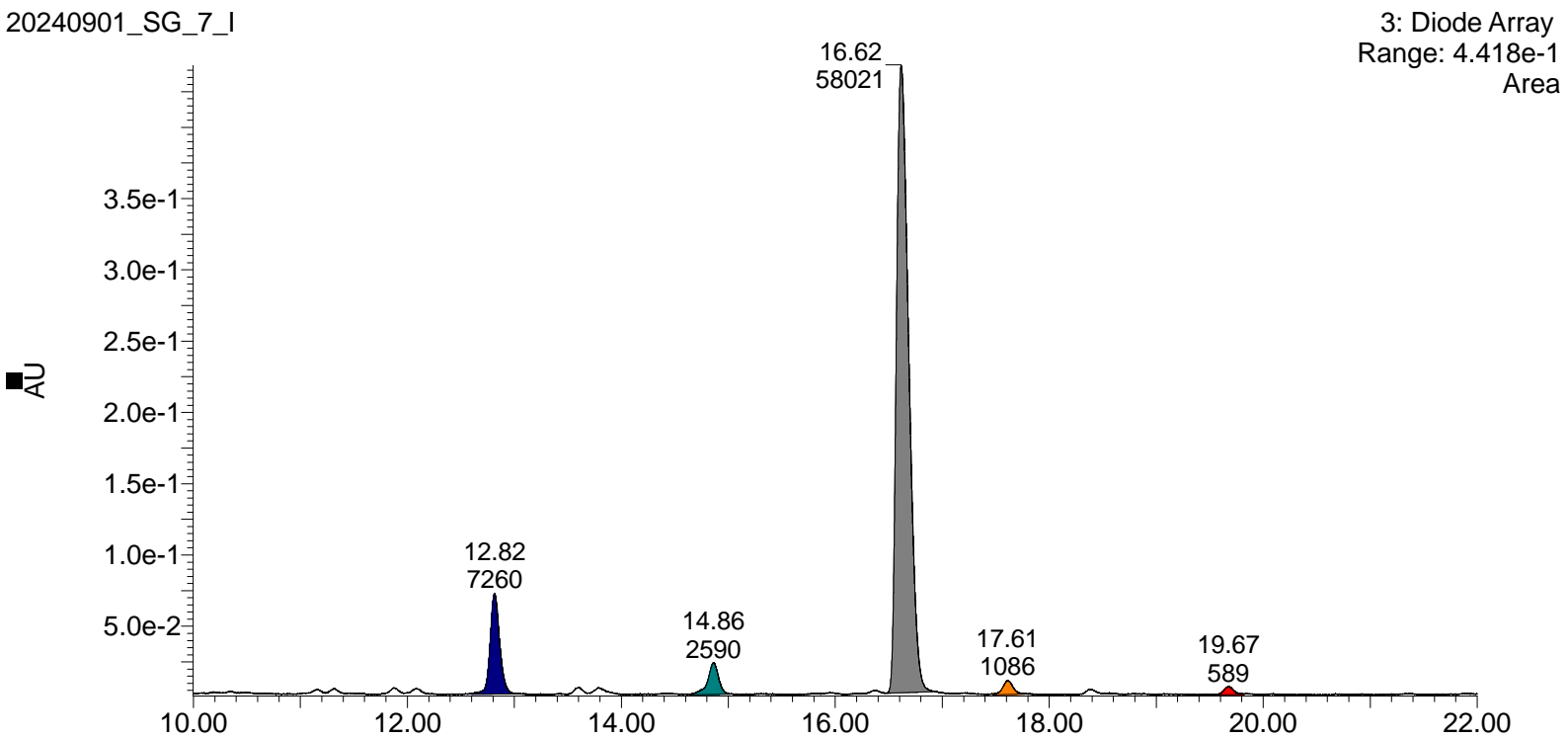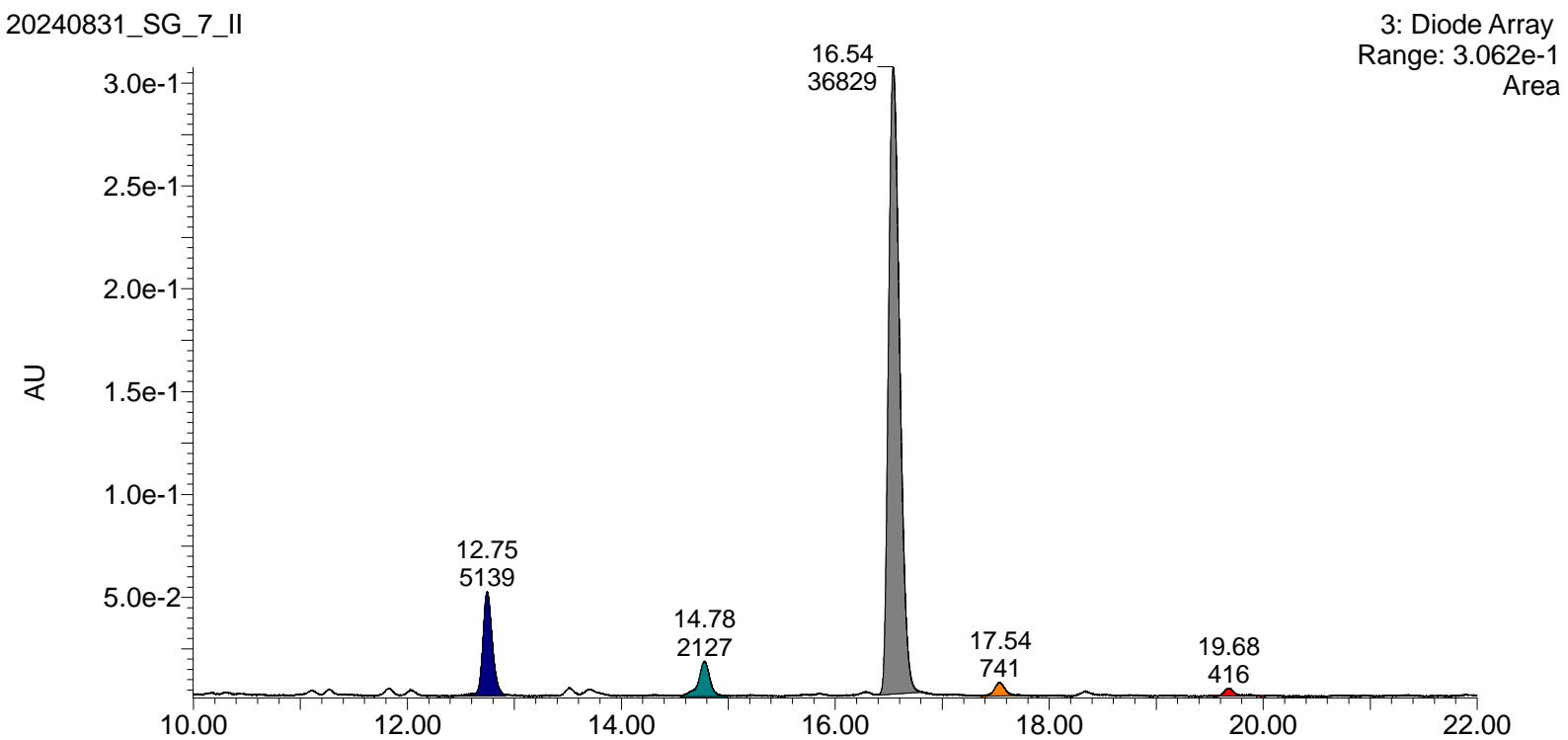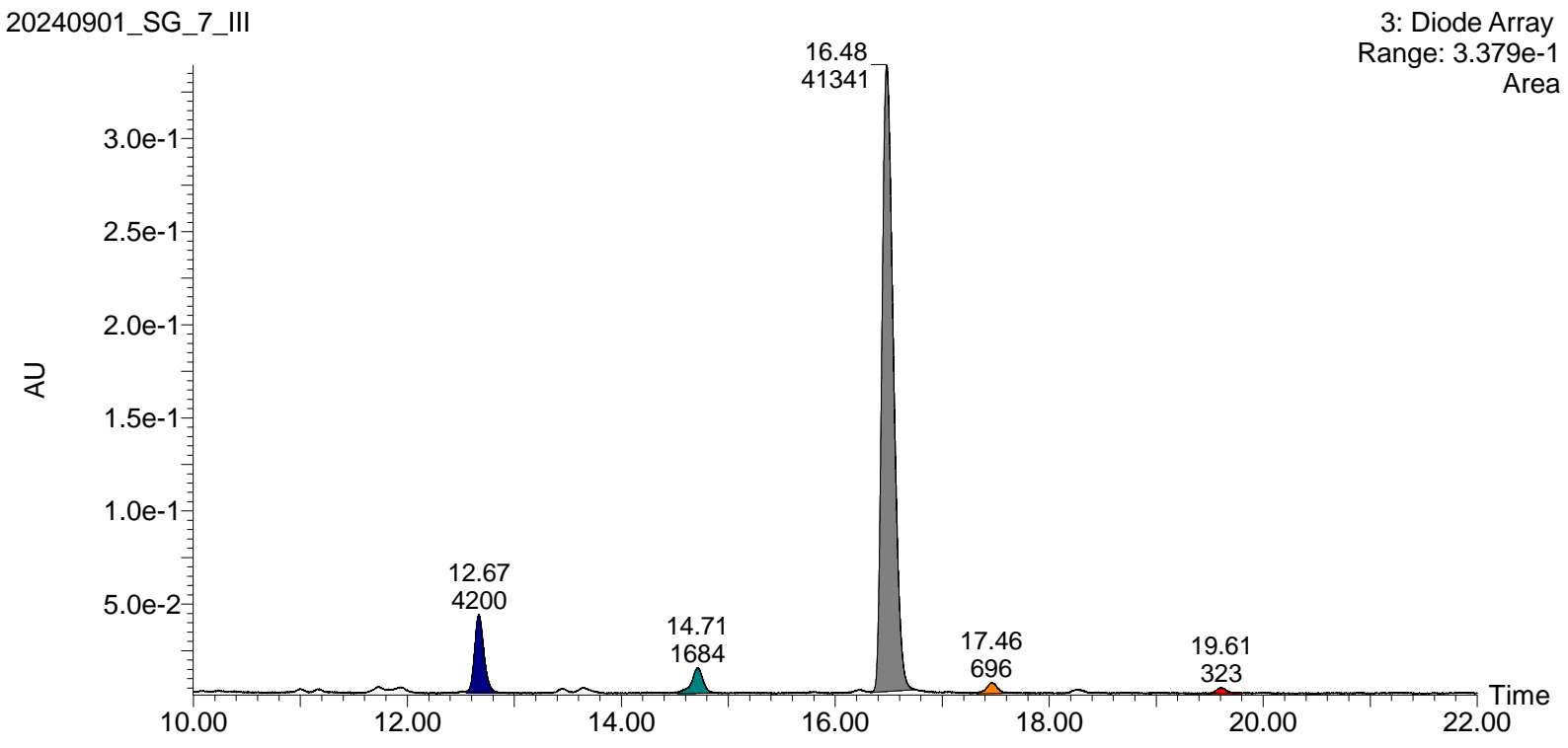

Supplement: Supplementary file 11 — Source Data [file 41467_2025_64257_MOESM11_ESM.zip › Source Data/Figure 3/tiEMCV-EGFP 2-5 CleaN3.pdf]

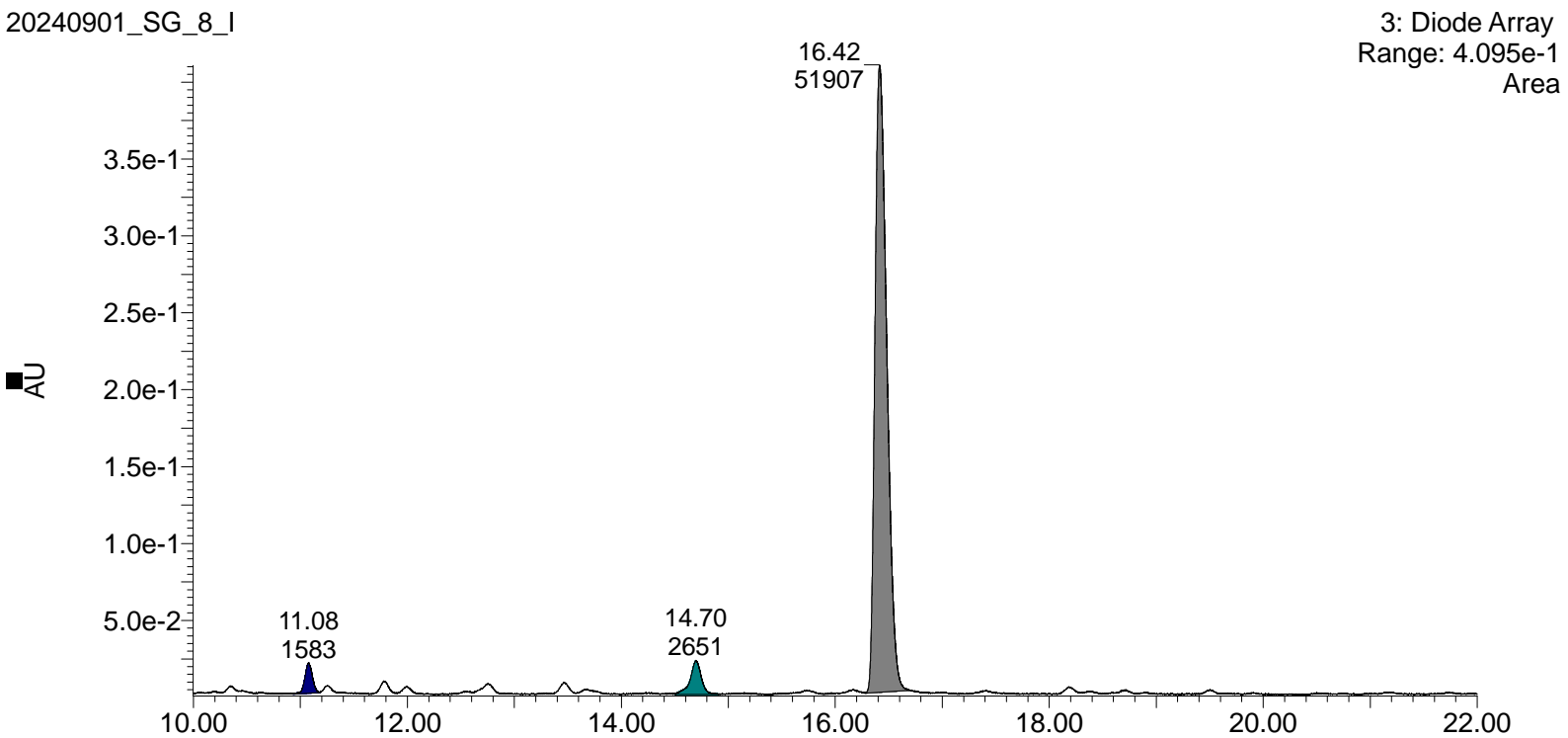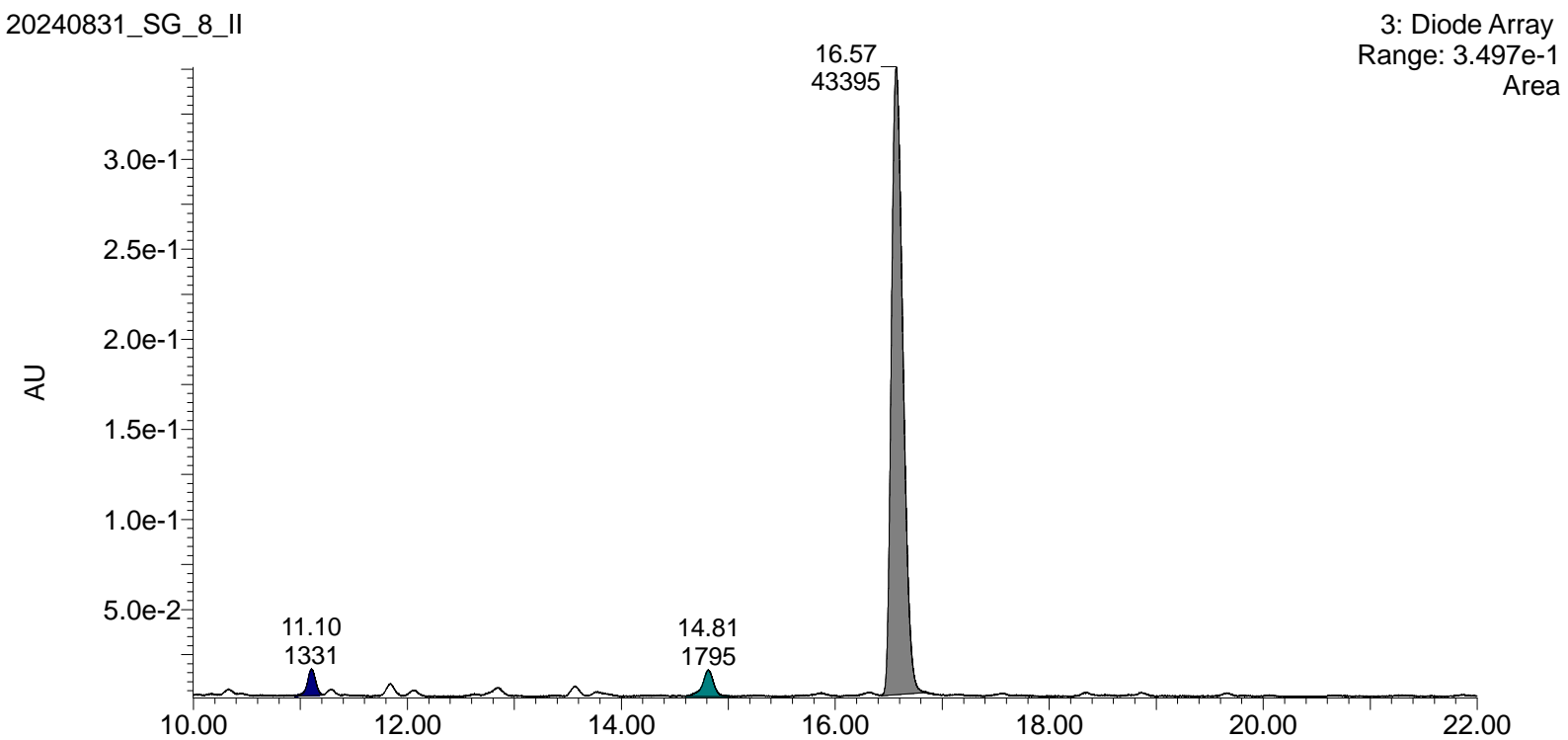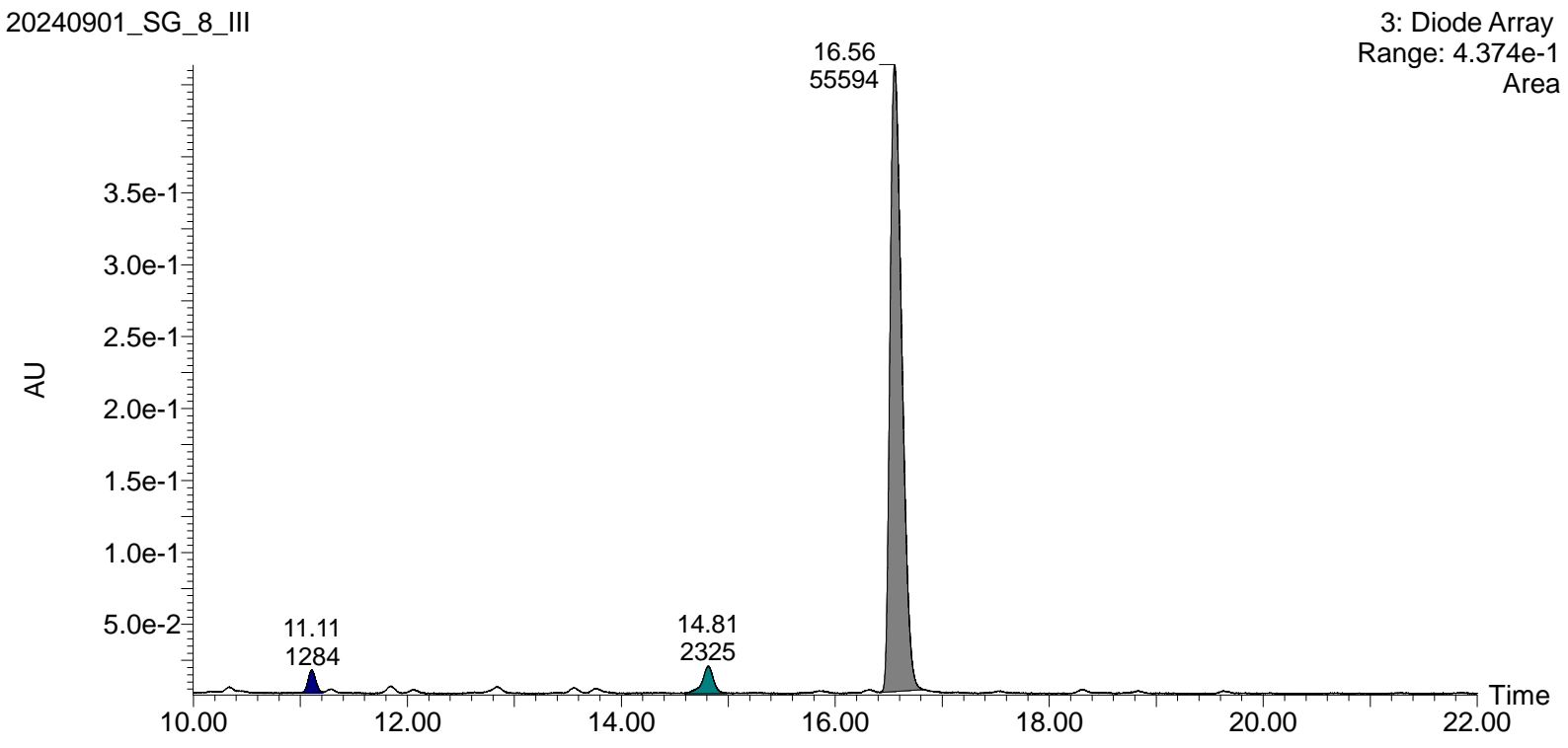

Supplement: Supplementary file 11 — Source Data [file 41467_2025_64257_MOESM11_ESM.zip › Source Data/Figure 3/tiEMCV-EGFP 6-5 CleaN3.pdf]

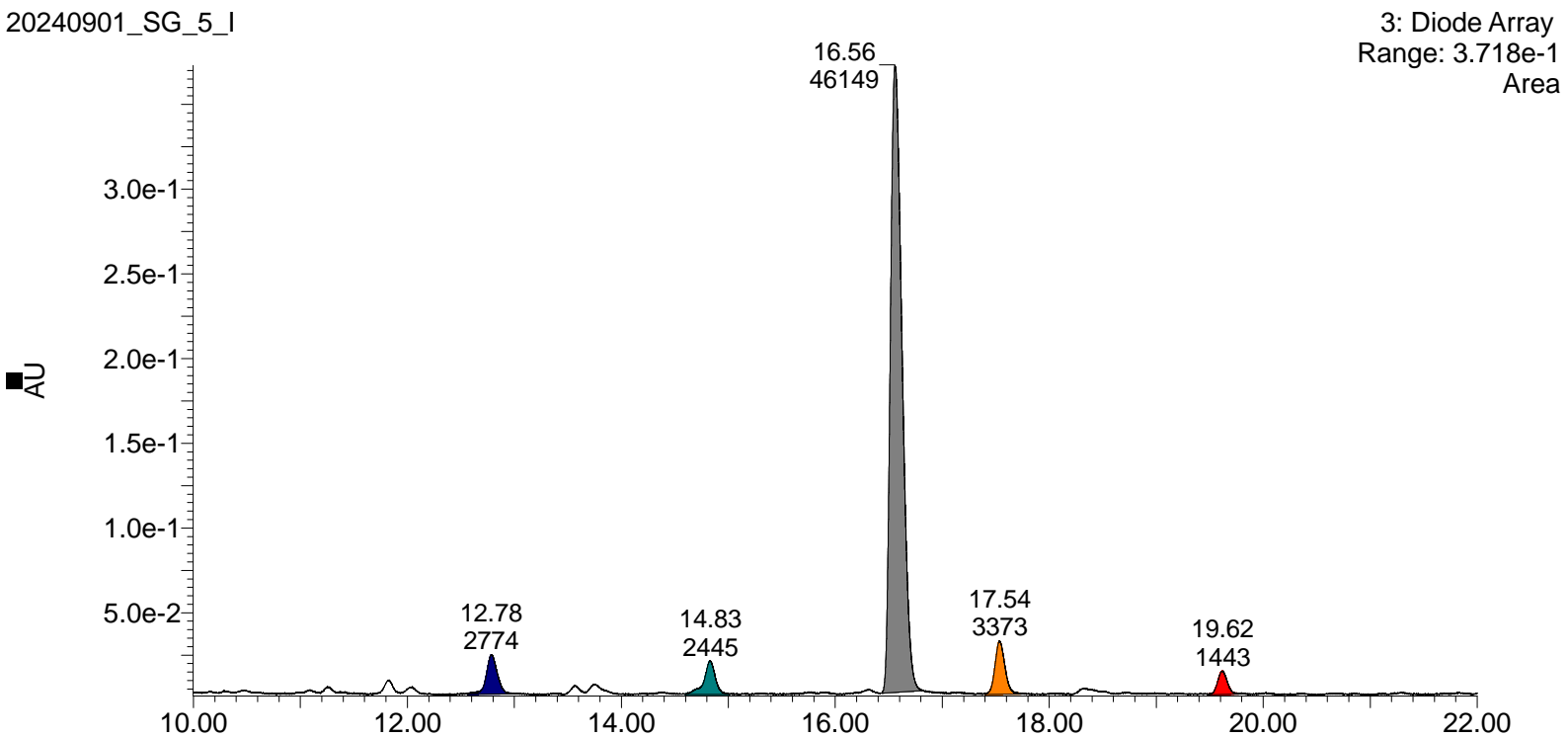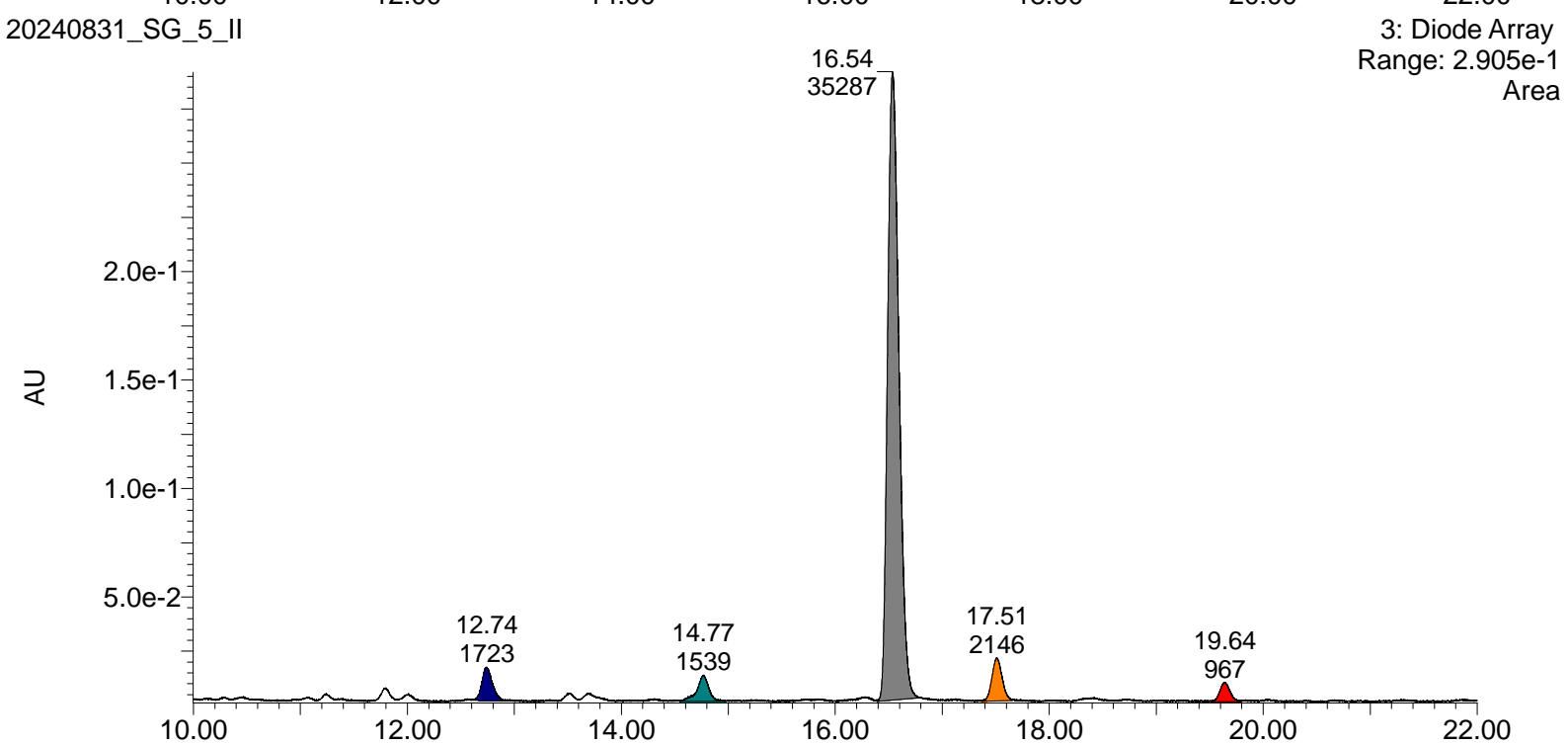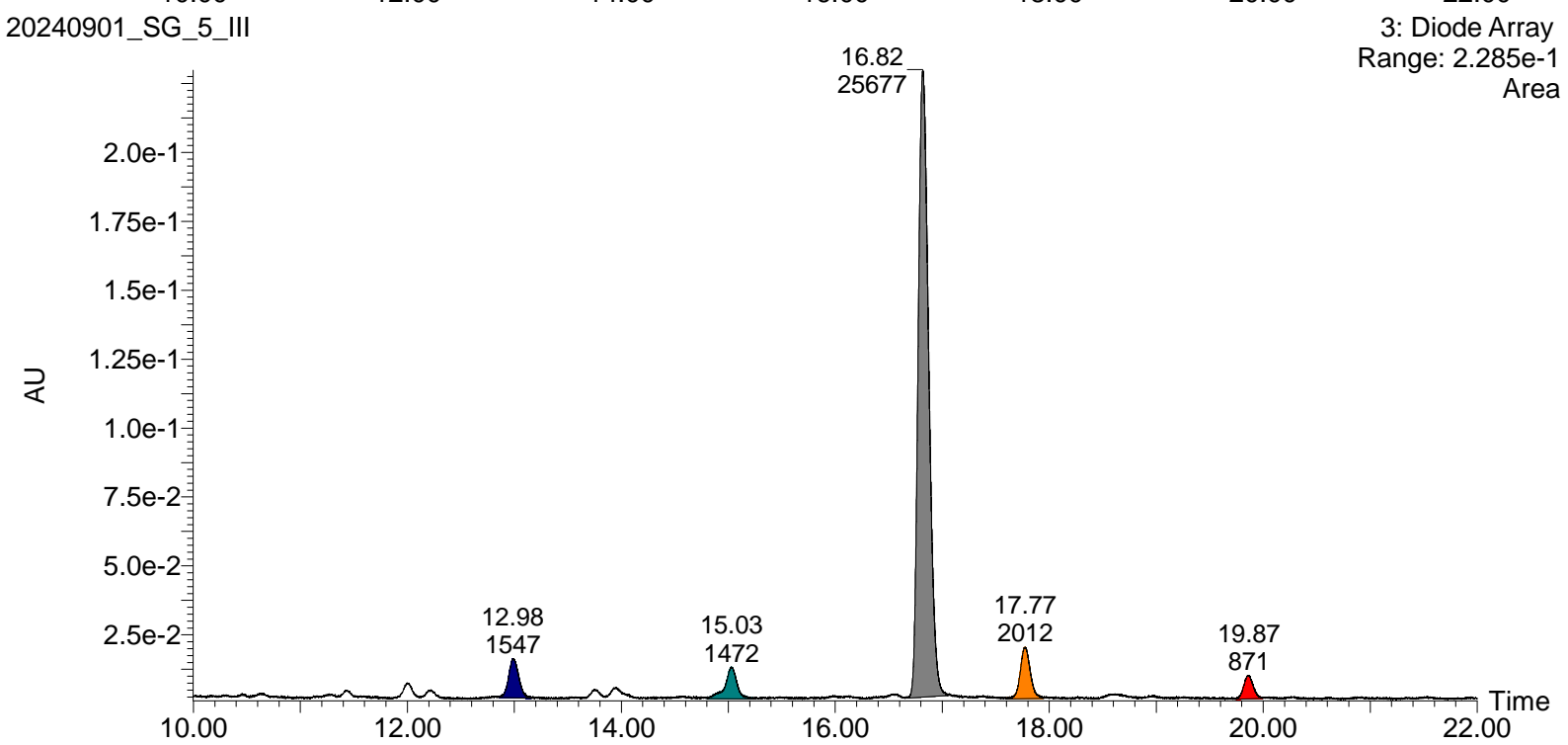

Supplement: Supplementary file 11 — Source Data [file 41467_2025_64257_MOESM11_ESM.zip › Source Data/Figure 3/tiEMCV-EGFP A-ins 6-5 CleaN3.pdf]

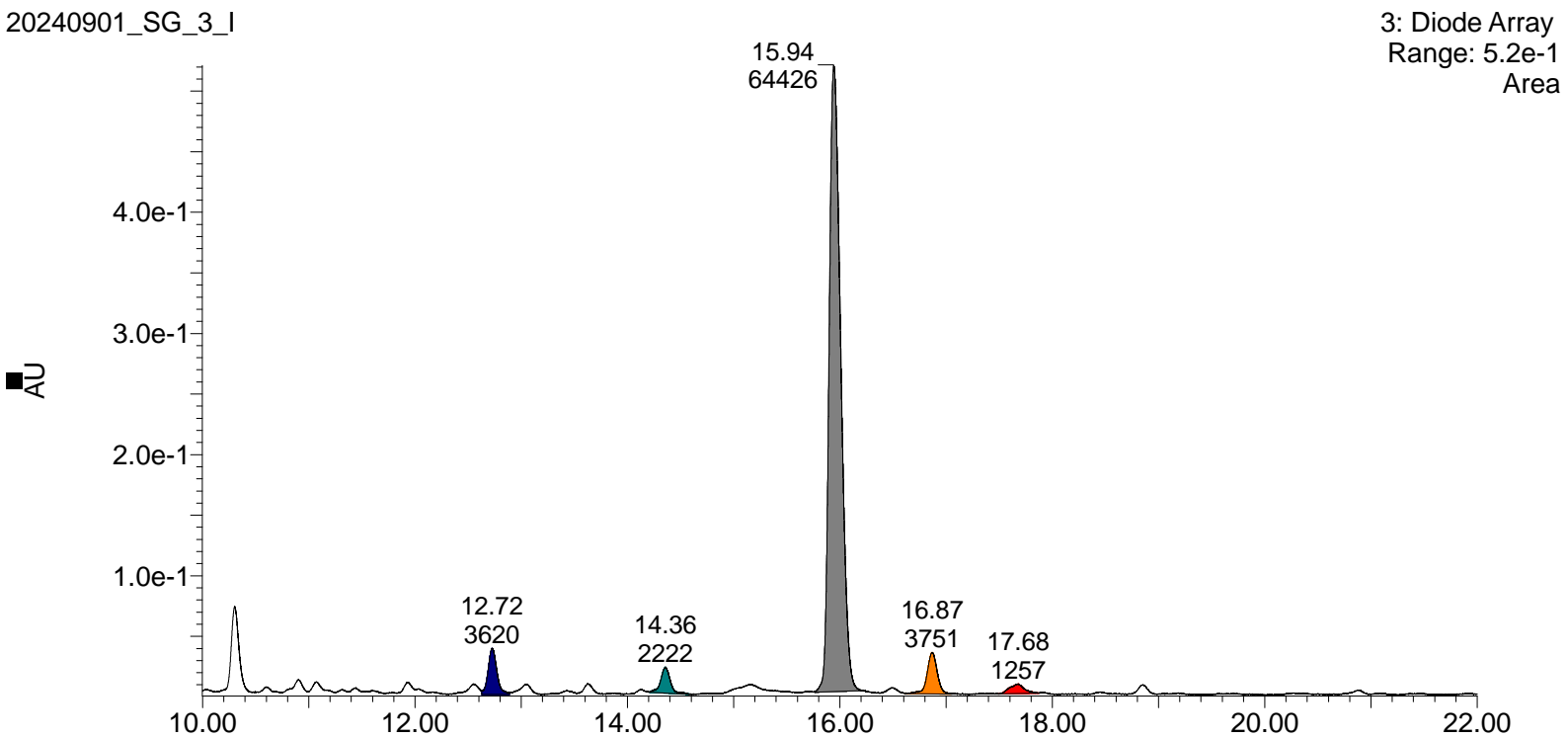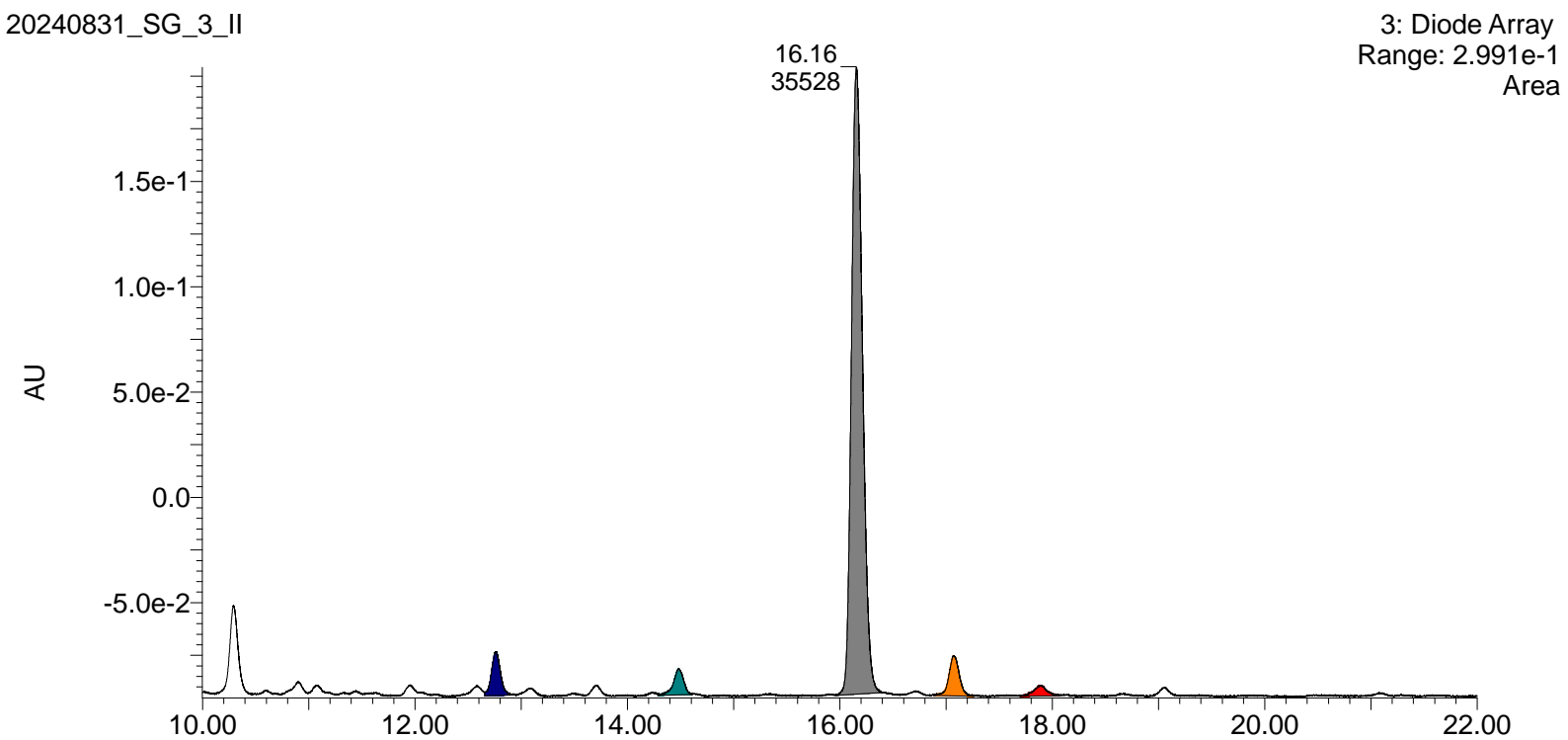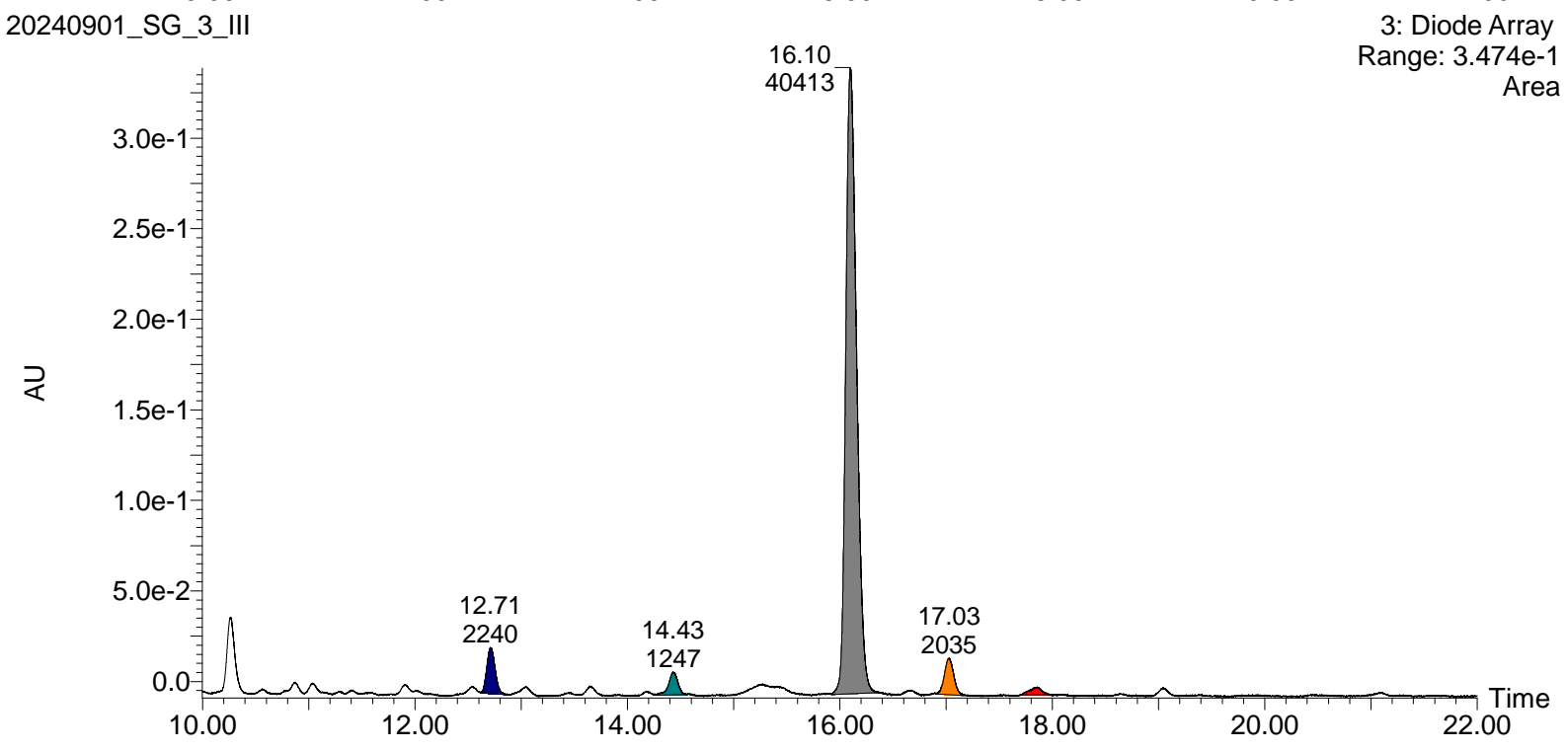

Supplement: Supplementary file 11 — Source Data [file 41467_2025_64257_MOESM11_ESM.zip › Source Data/Figure 3/tiEMCV-EGFP A-ins 6-5 CleanCap.pdf]

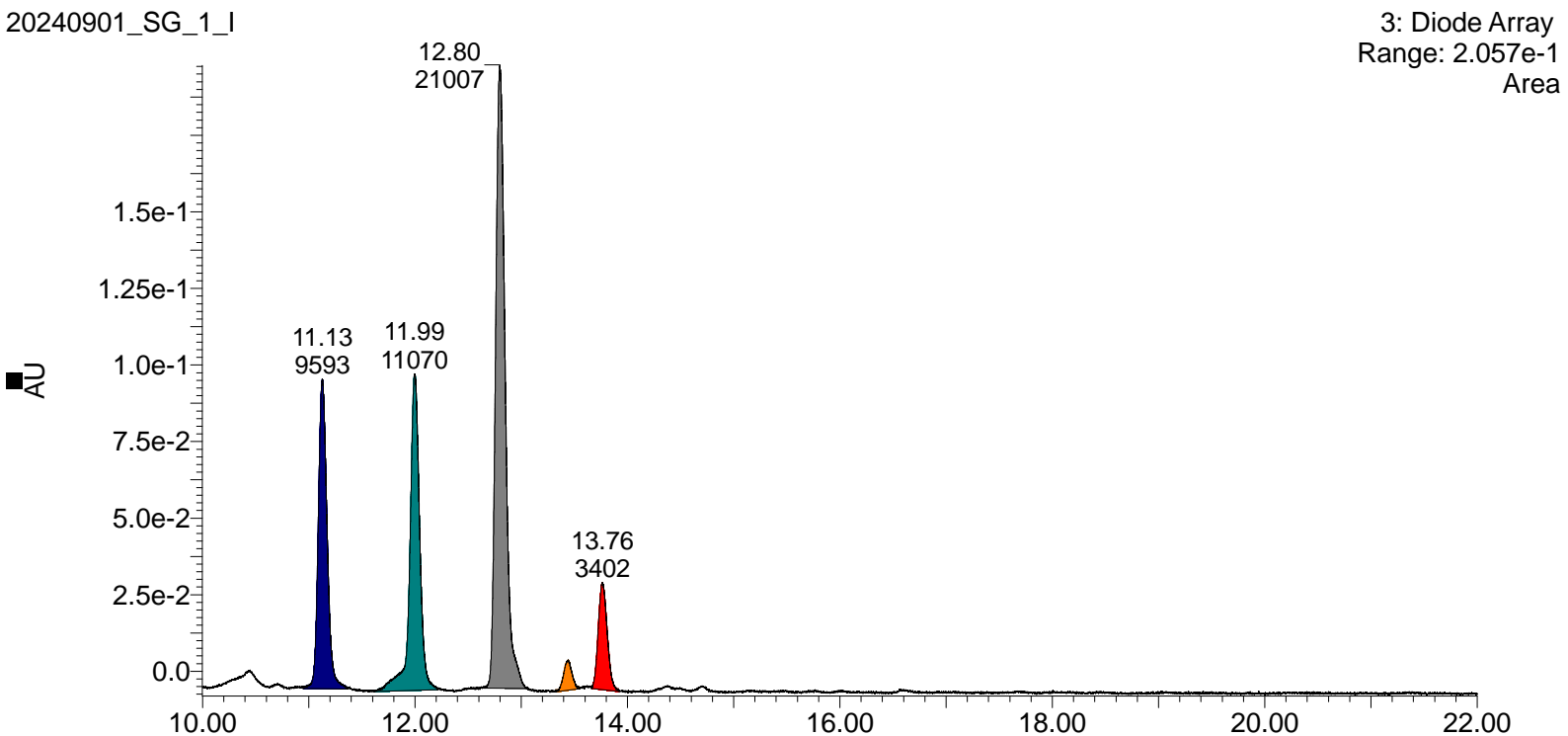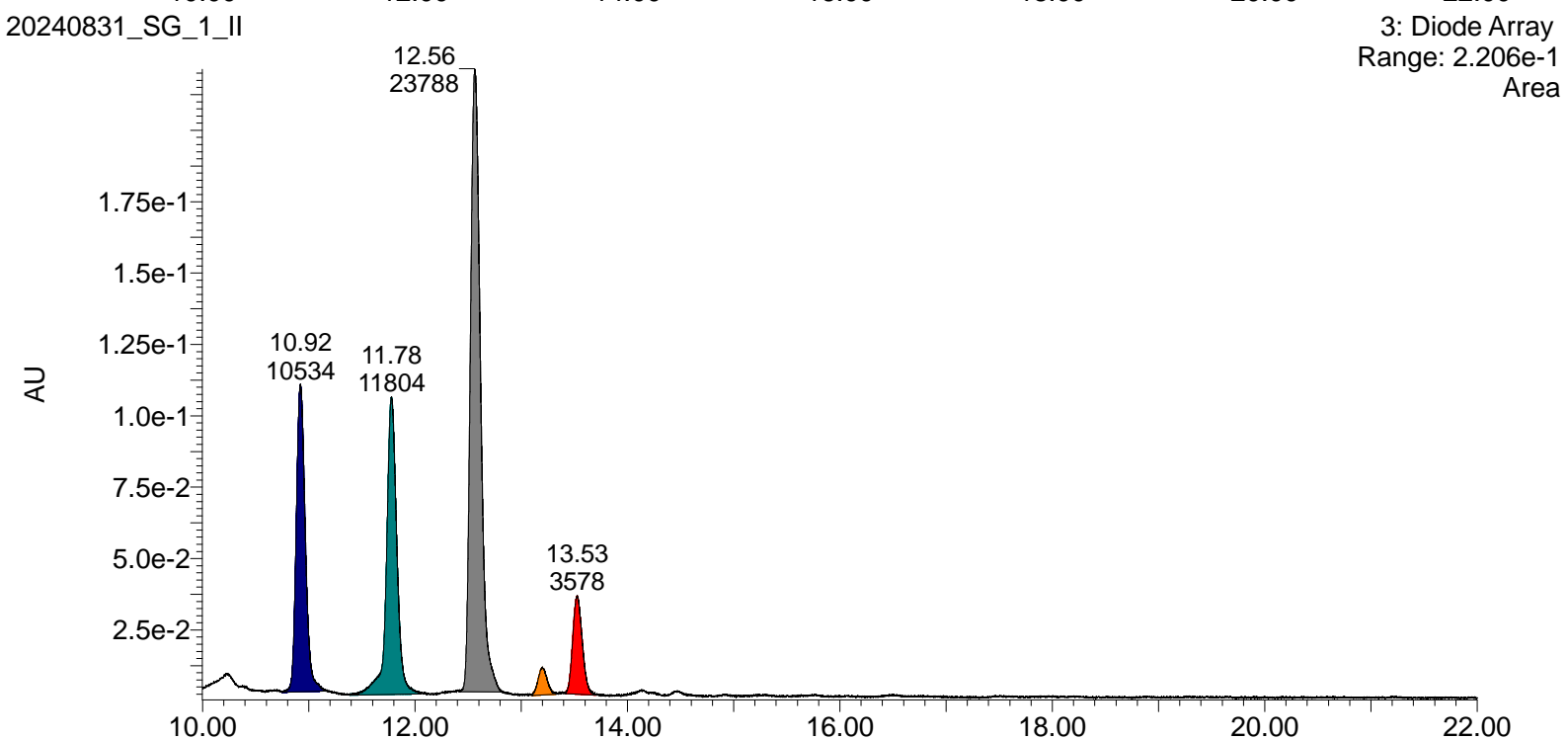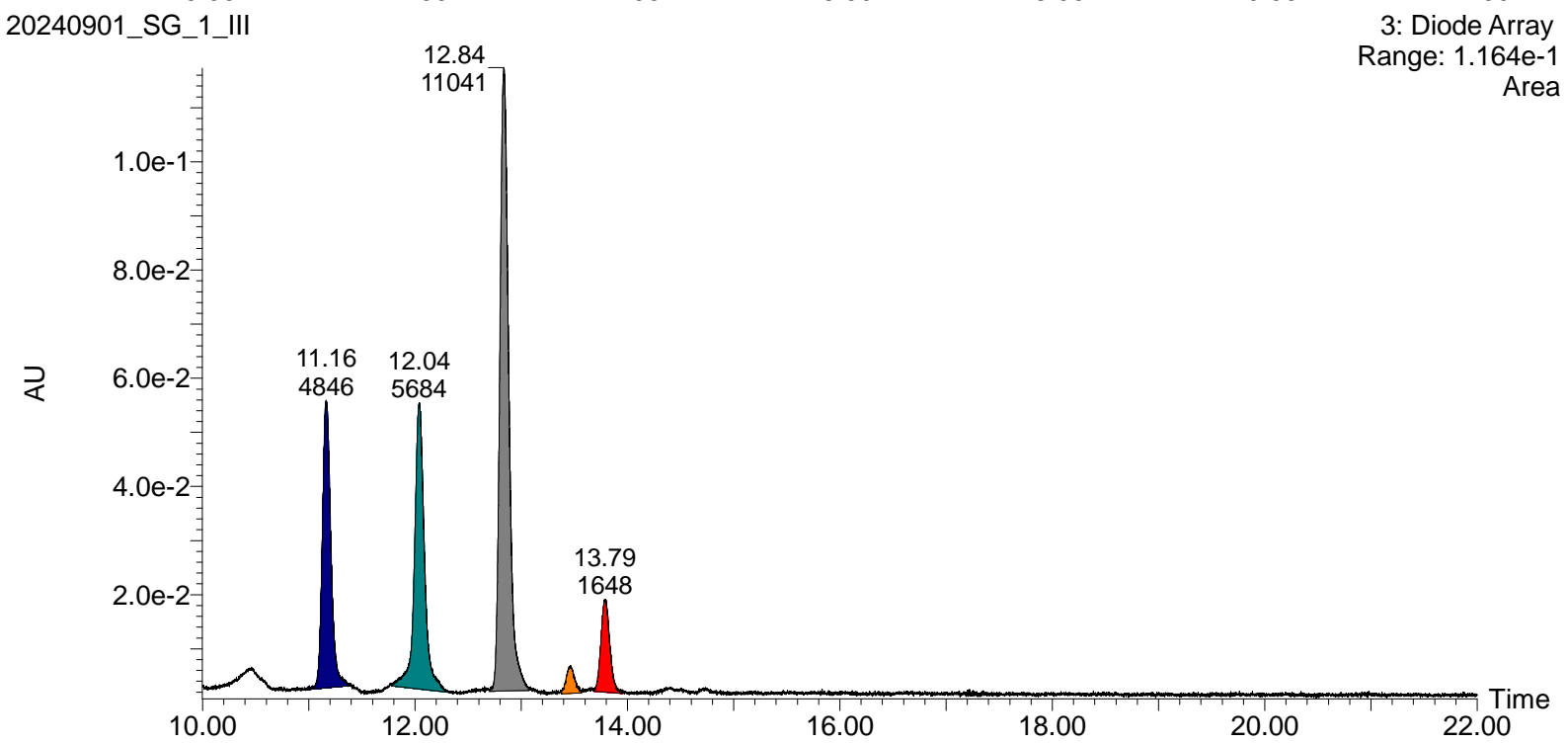

Supplement: Supplementary file 11 — Source Data [file 41467_2025_64257_MOESM11_ESM.zip › Source Data/Figure 3/tiEMCV-EGFP A-ins 6-5 non-primed.pdf]

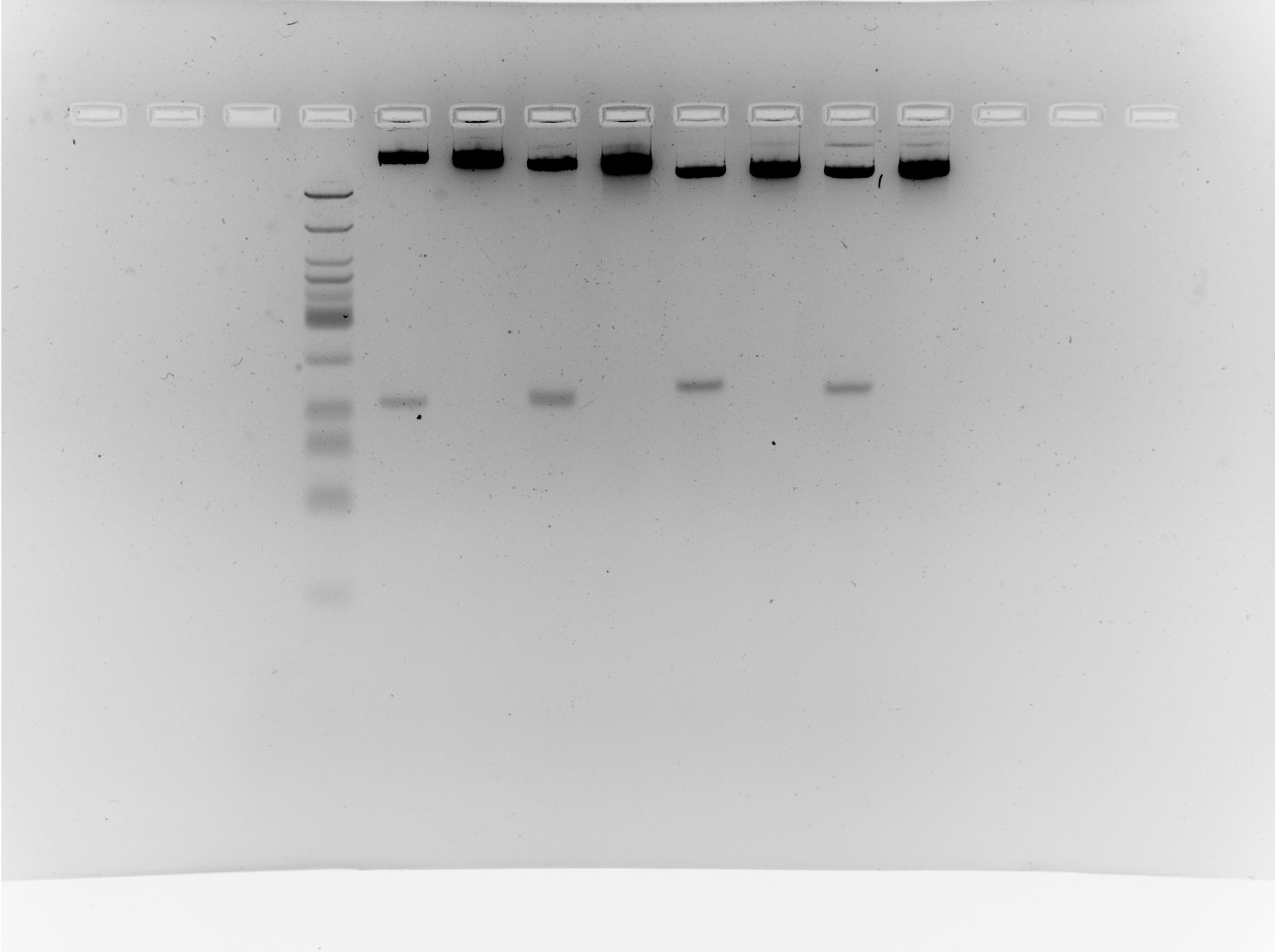

Supplement: Supplementary file 11 — Source Data [file 41467_2025_64257_MOESM11_ESM.zip › Source Data/Supplementary Figure 1/encoded poly(A) tail length.tif]

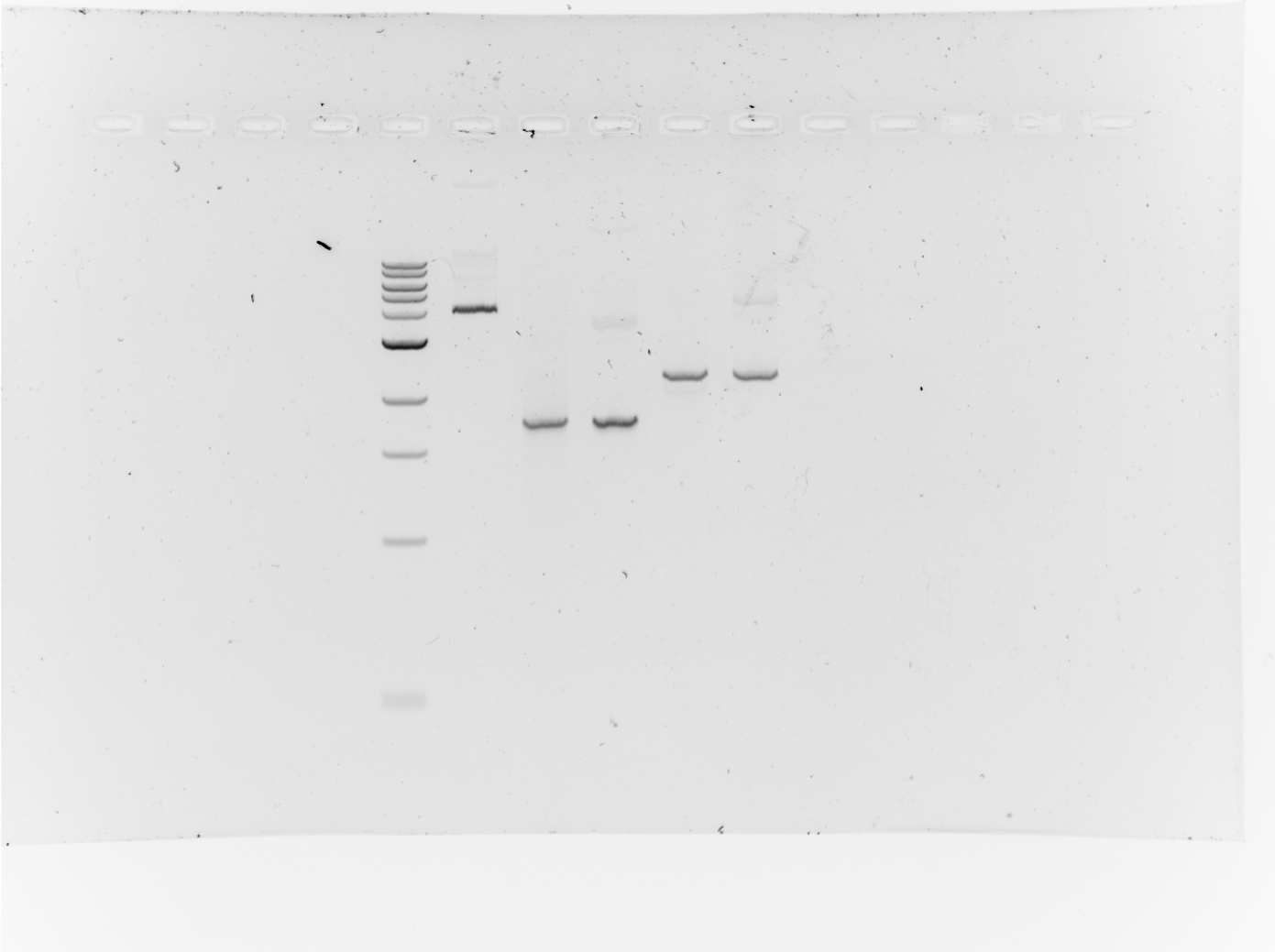

Supplement: Supplementary file 11 — Source Data [file 41467_2025_64257_MOESM11_ESM.zip › Source Data/Supplementary Figure 1/iSyn templates.tif]

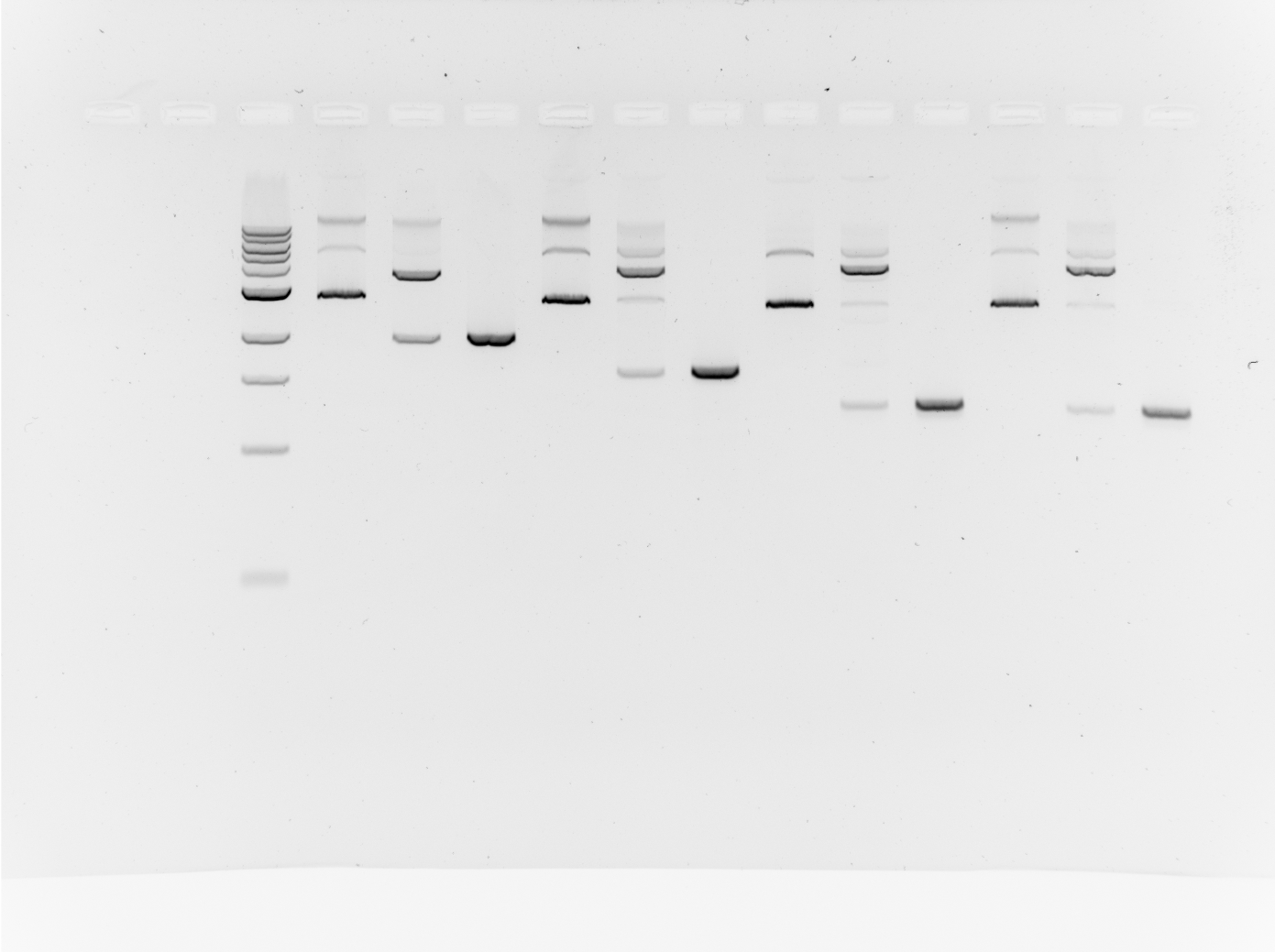

Supplement: Supplementary file 11 — Source Data [file 41467_2025_64257_MOESM11_ESM.zip › Source Data/Supplementary Figure 1/templates from pmRNA plasmids.tif]

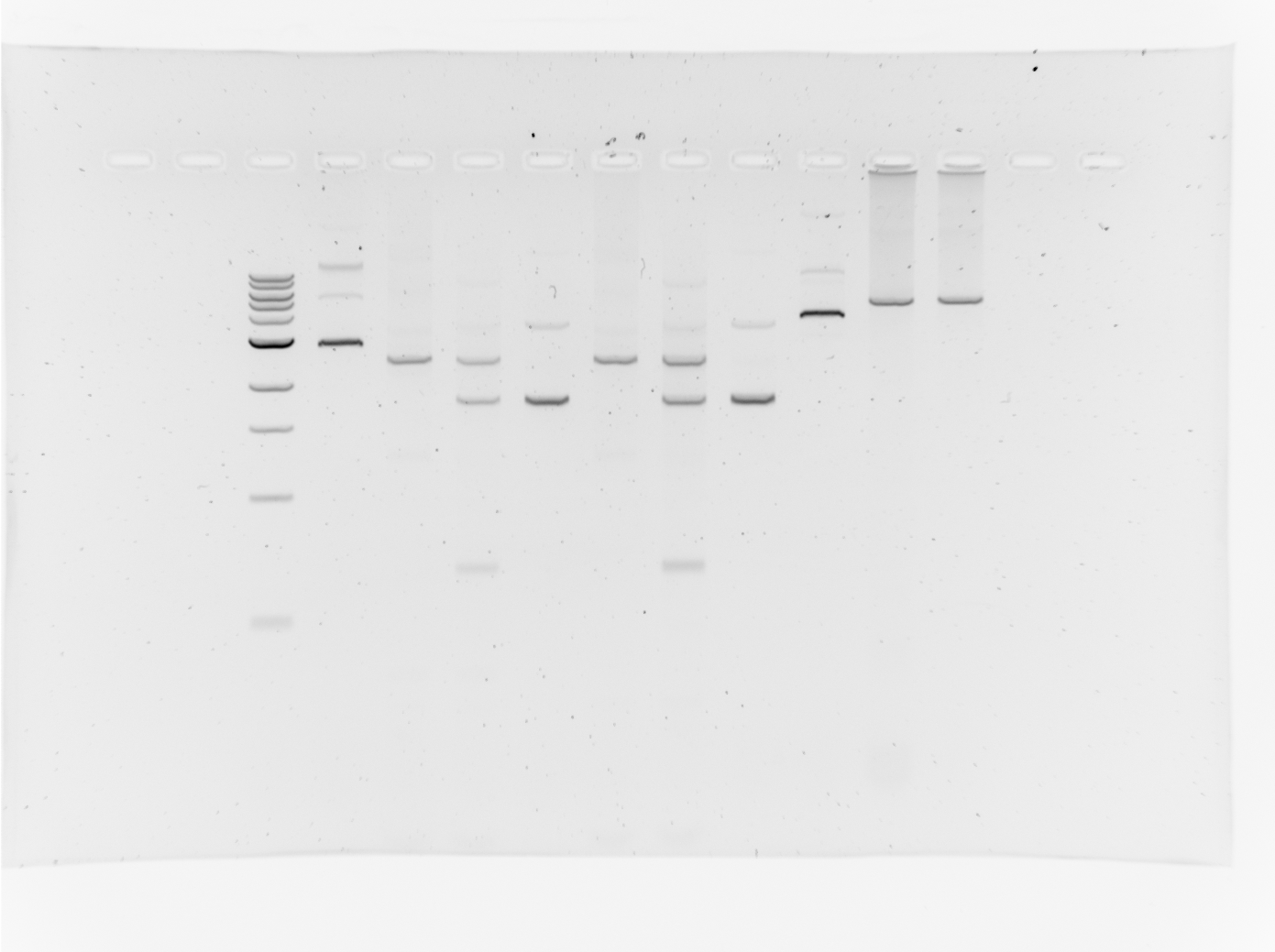

Supplement: Supplementary file 11 — Source Data [file 41467_2025_64257_MOESM11_ESM.zip › Source Data/Supplementary Figure 1/templates made via PCR.tif]

20240809\_SG\_CleanN3\_priming\_titr\_10mM\_1

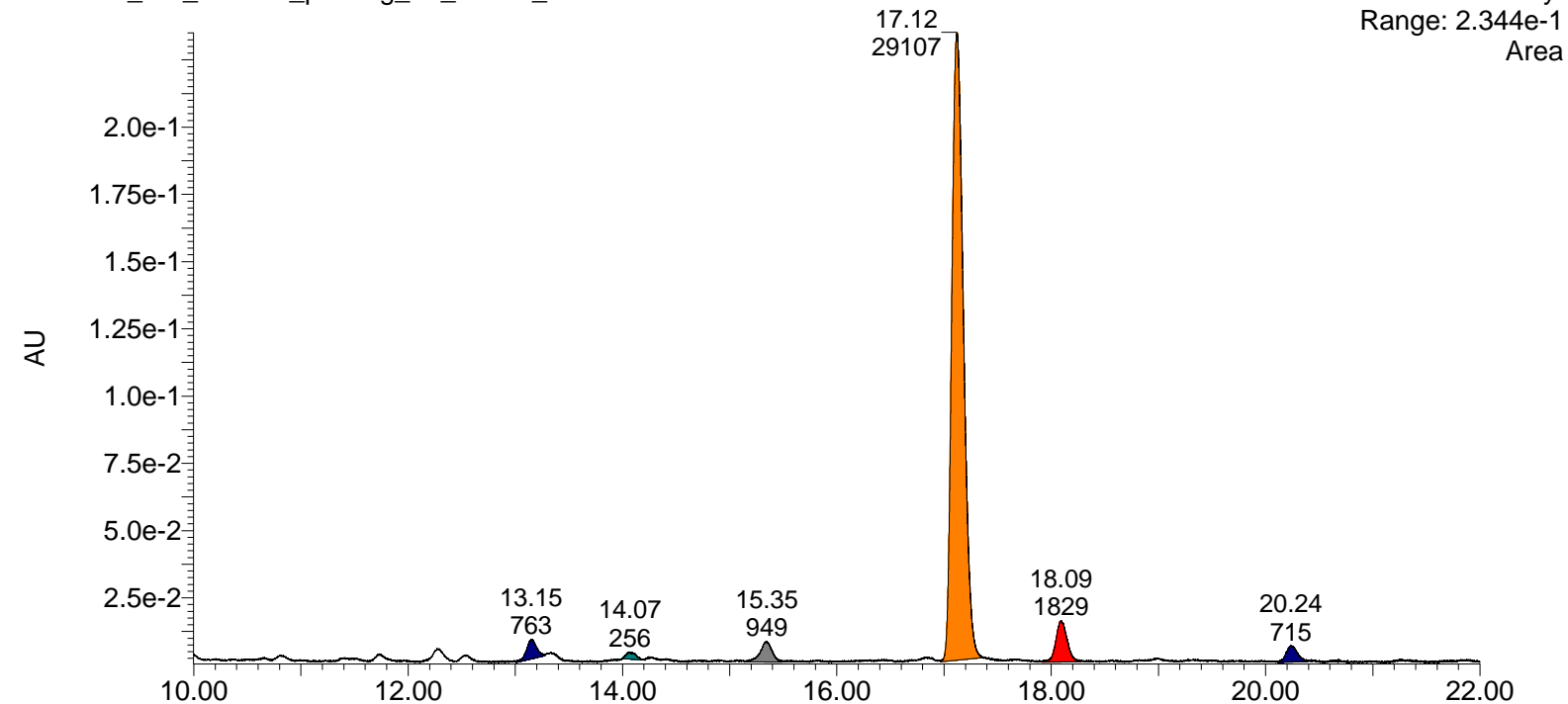

20240809\_SG\_CleanN3\_priming\_titr\_10mM\_2

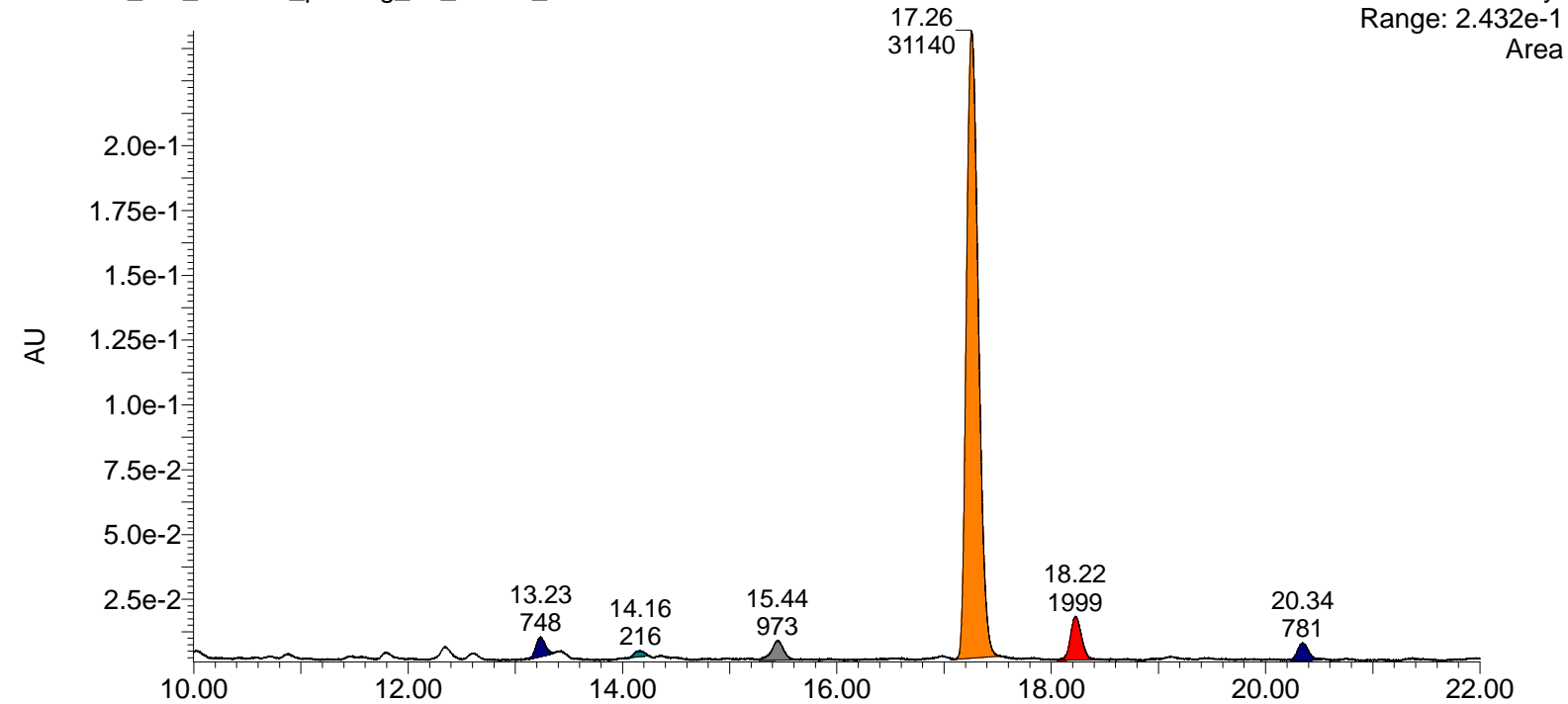

20240809\_SG\_CleanN3\_priming\_titr\_10mM\_3

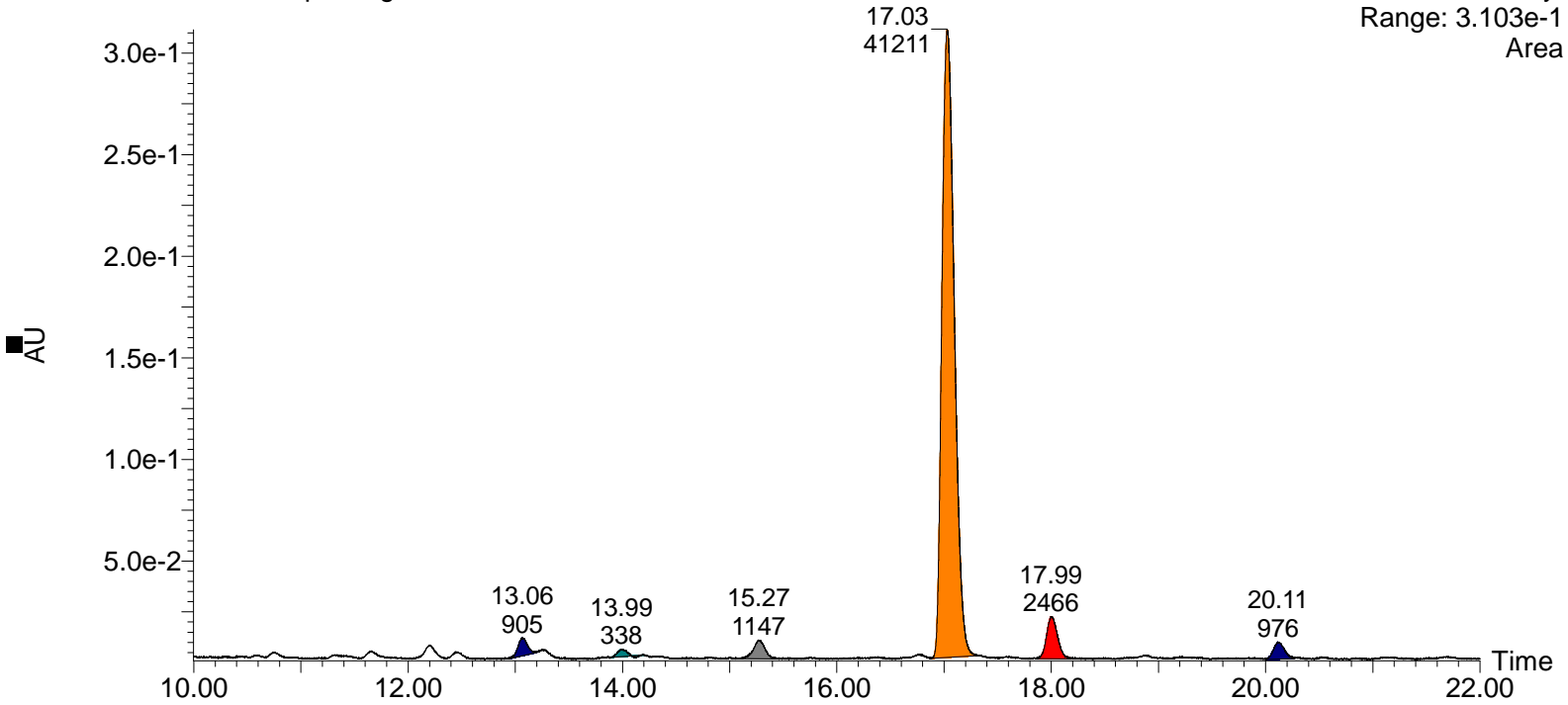

Supplement: Supplementary file 11 — Source Data [file 41467_2025_64257_MOESM11_ESM.zip › Source Data/Supplementary Figure 2/CleaN3 priming optimisation integration report 10 mM.pdf]

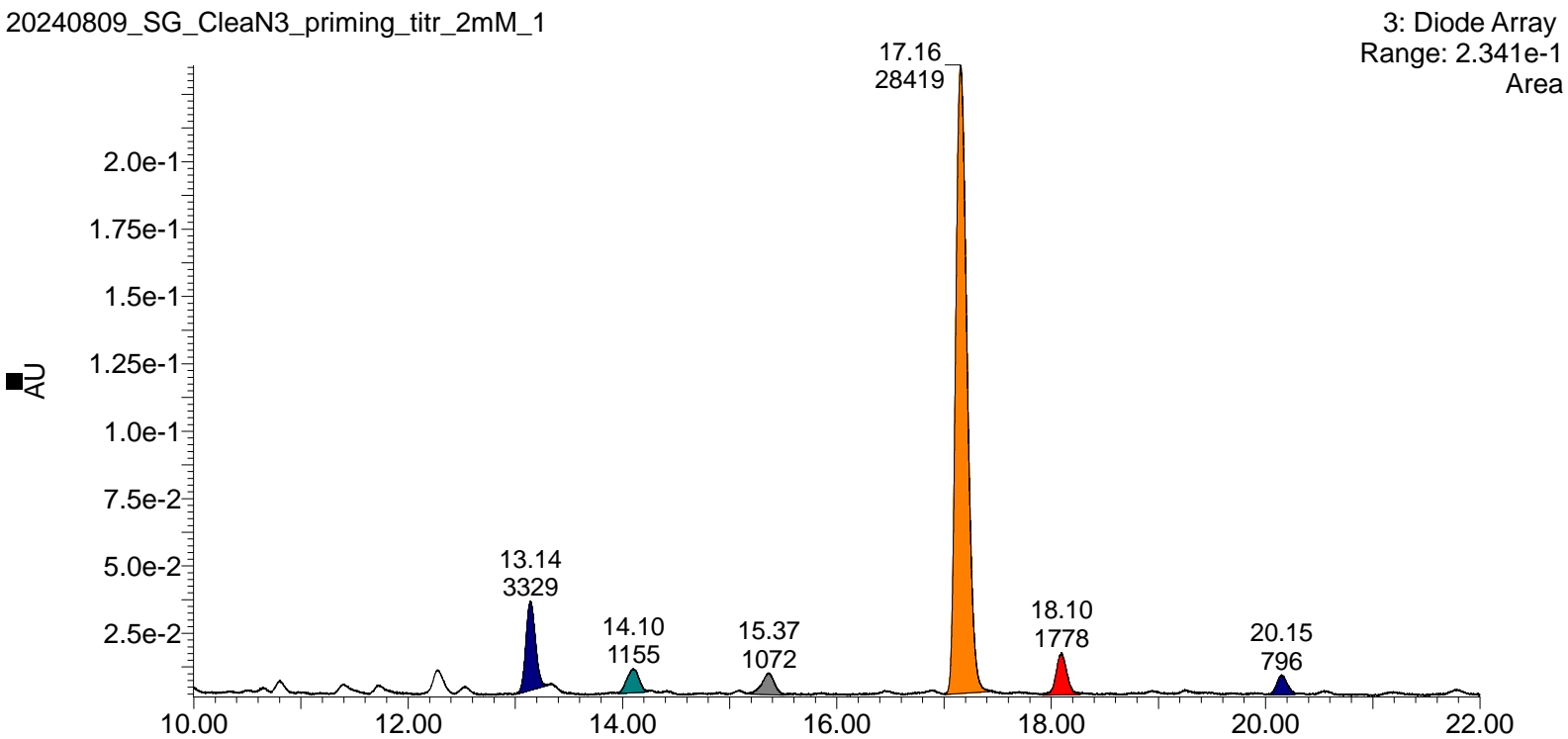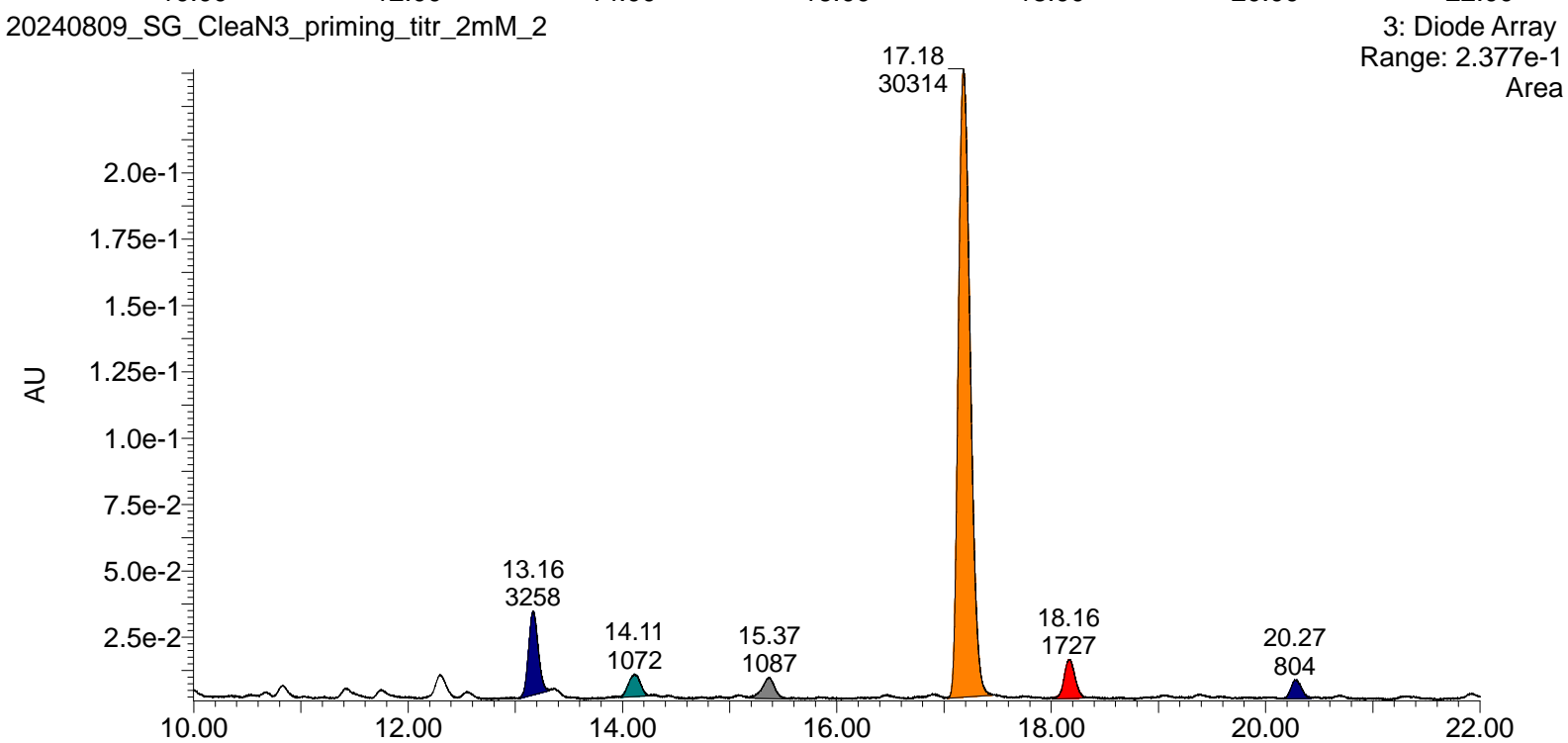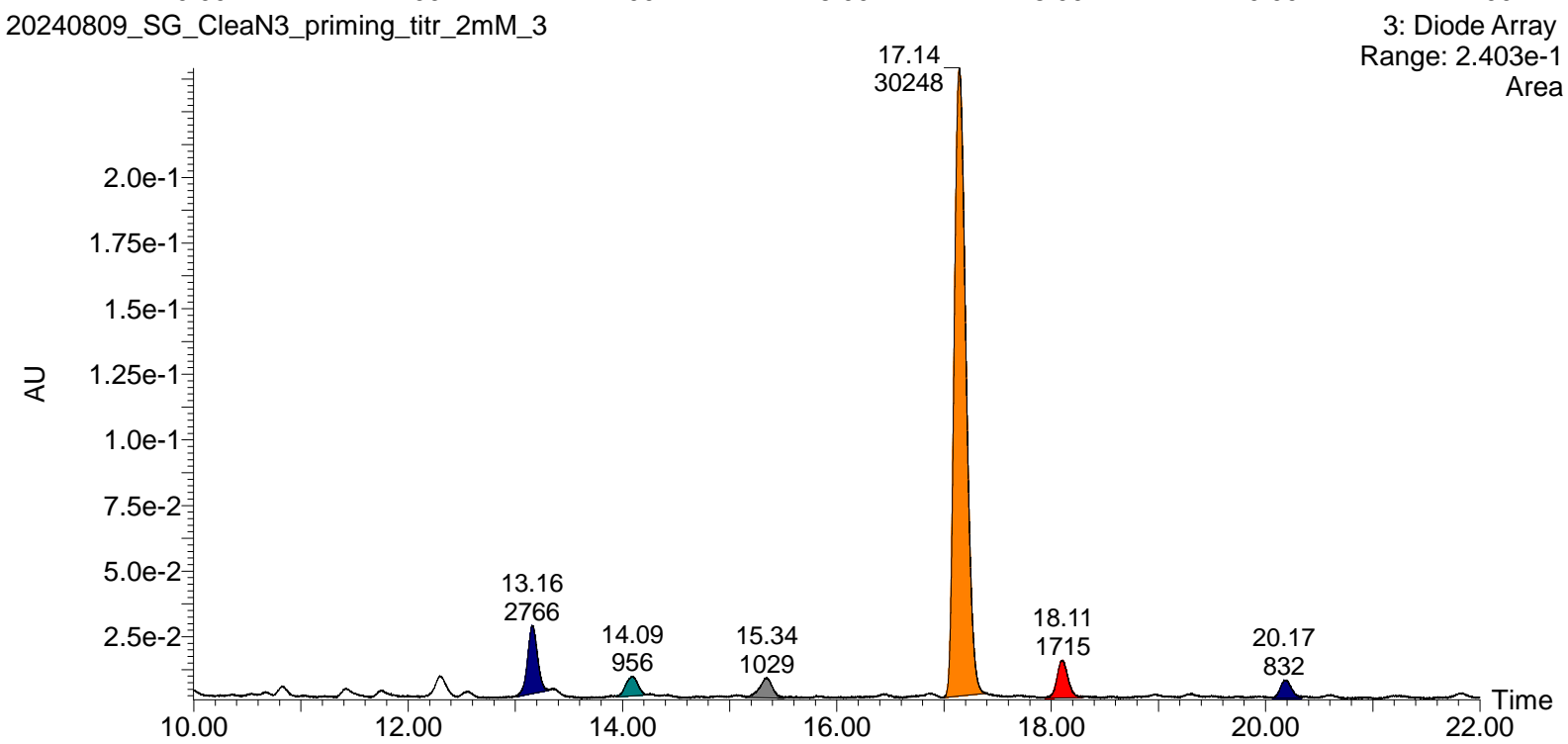

Supplement: Supplementary file 11 — Source Data [file 41467_2025_64257_MOESM11_ESM.zip › Source Data/Supplementary Figure 2/CleaN3 priming optimisation integration report 2 mM.pdf]

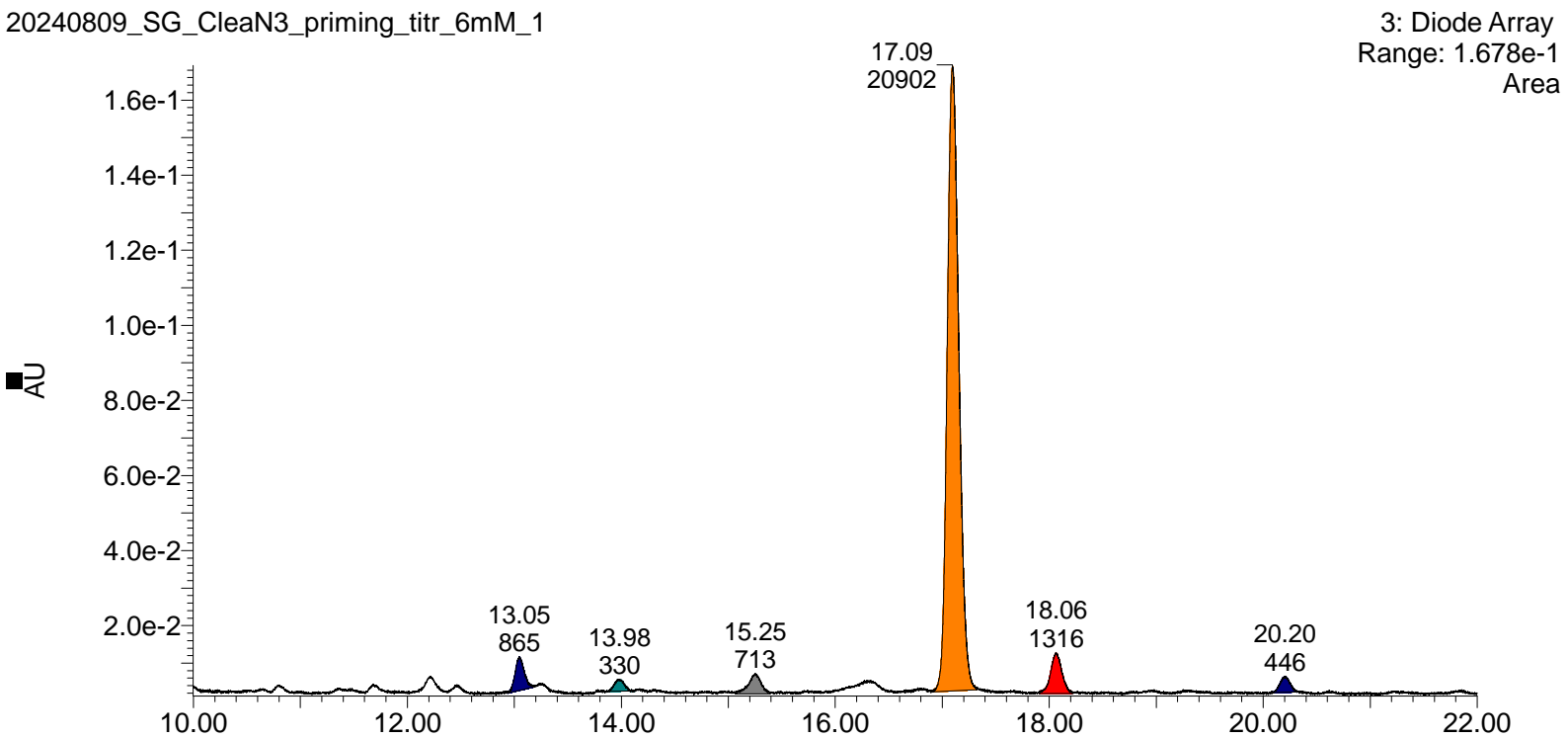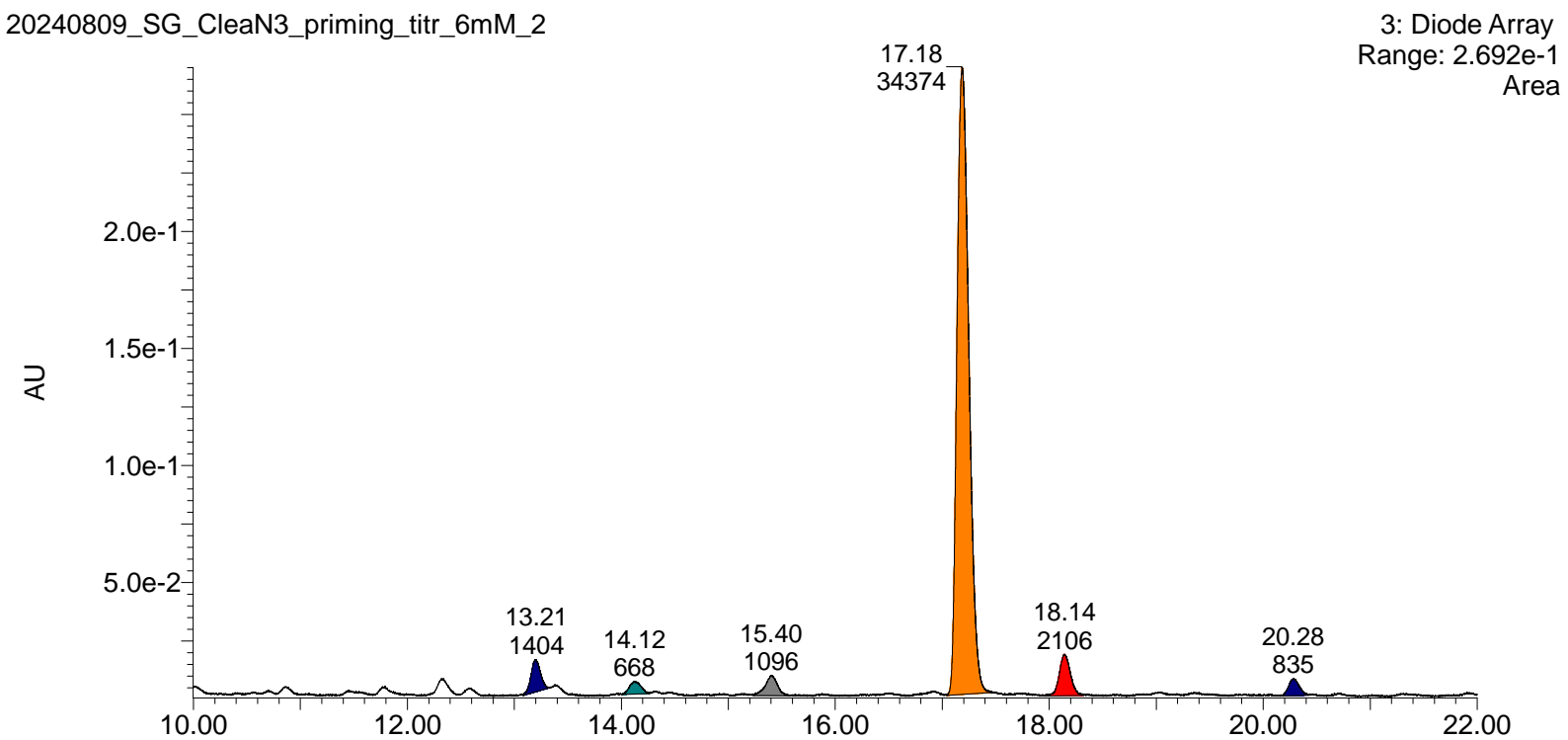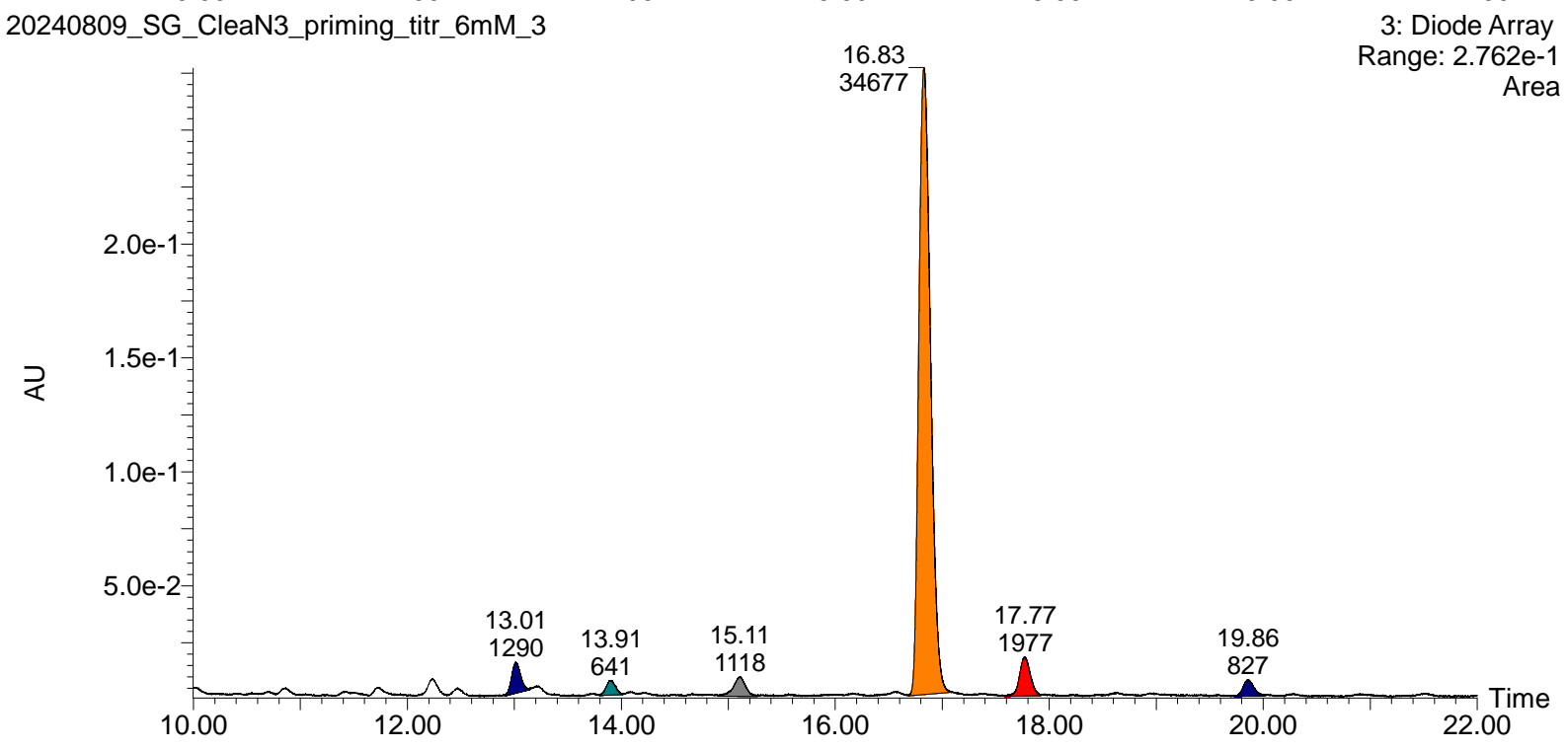

Supplement: Supplementary file 11 — Source Data [file 41467_2025_64257_MOESM11_ESM.zip › Source Data/Supplementary Figure 2/CleaN3 priming optimisation integration report 6 mM.pdf]

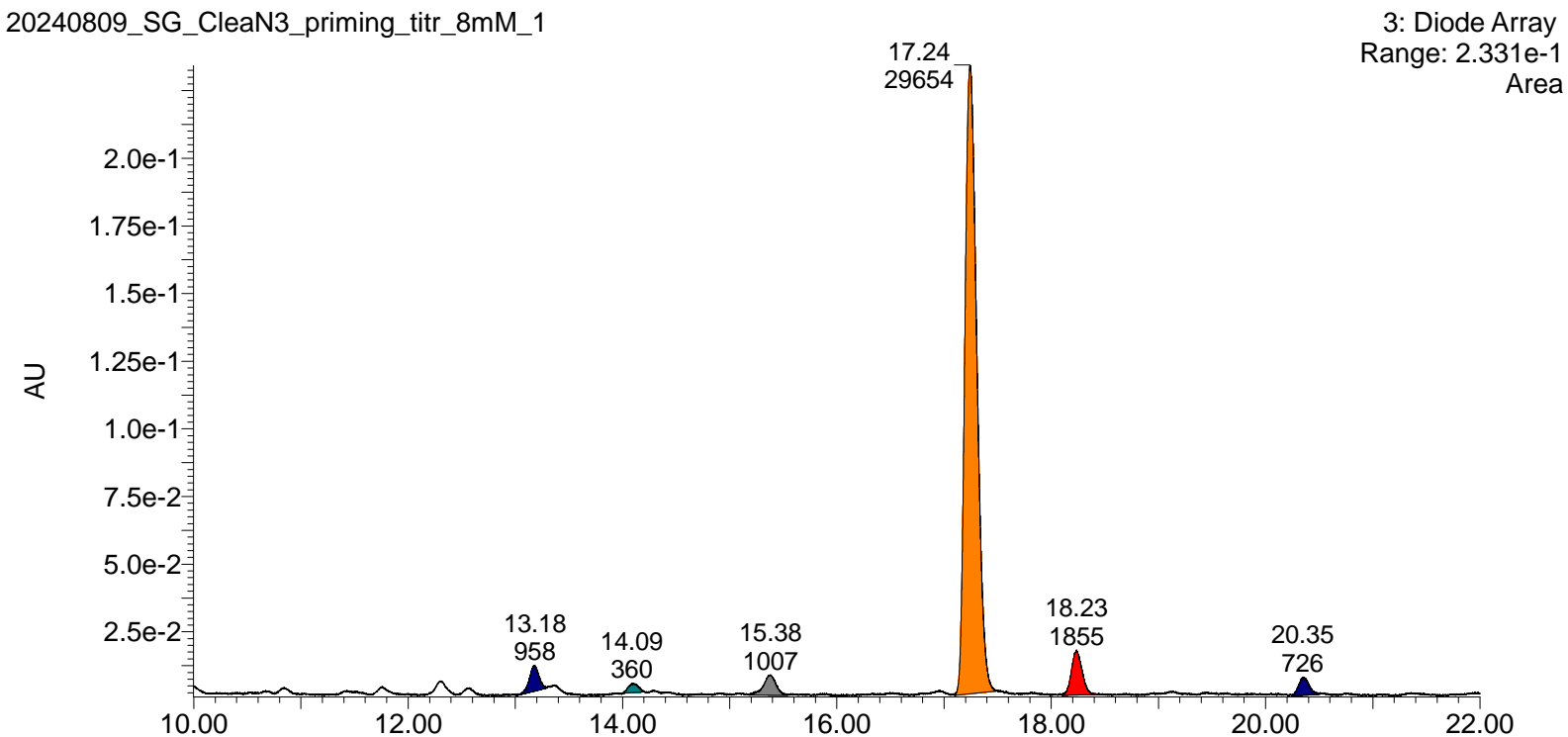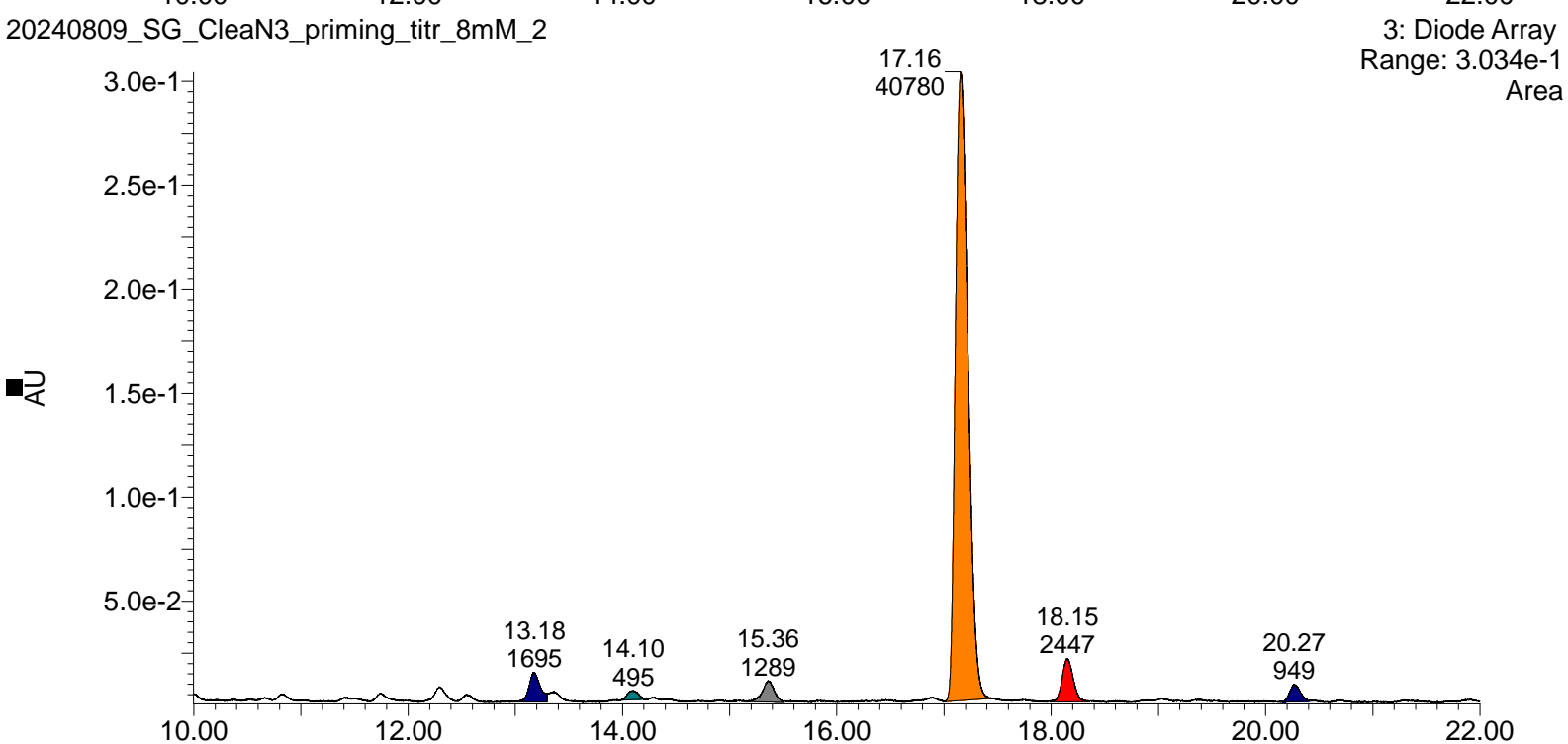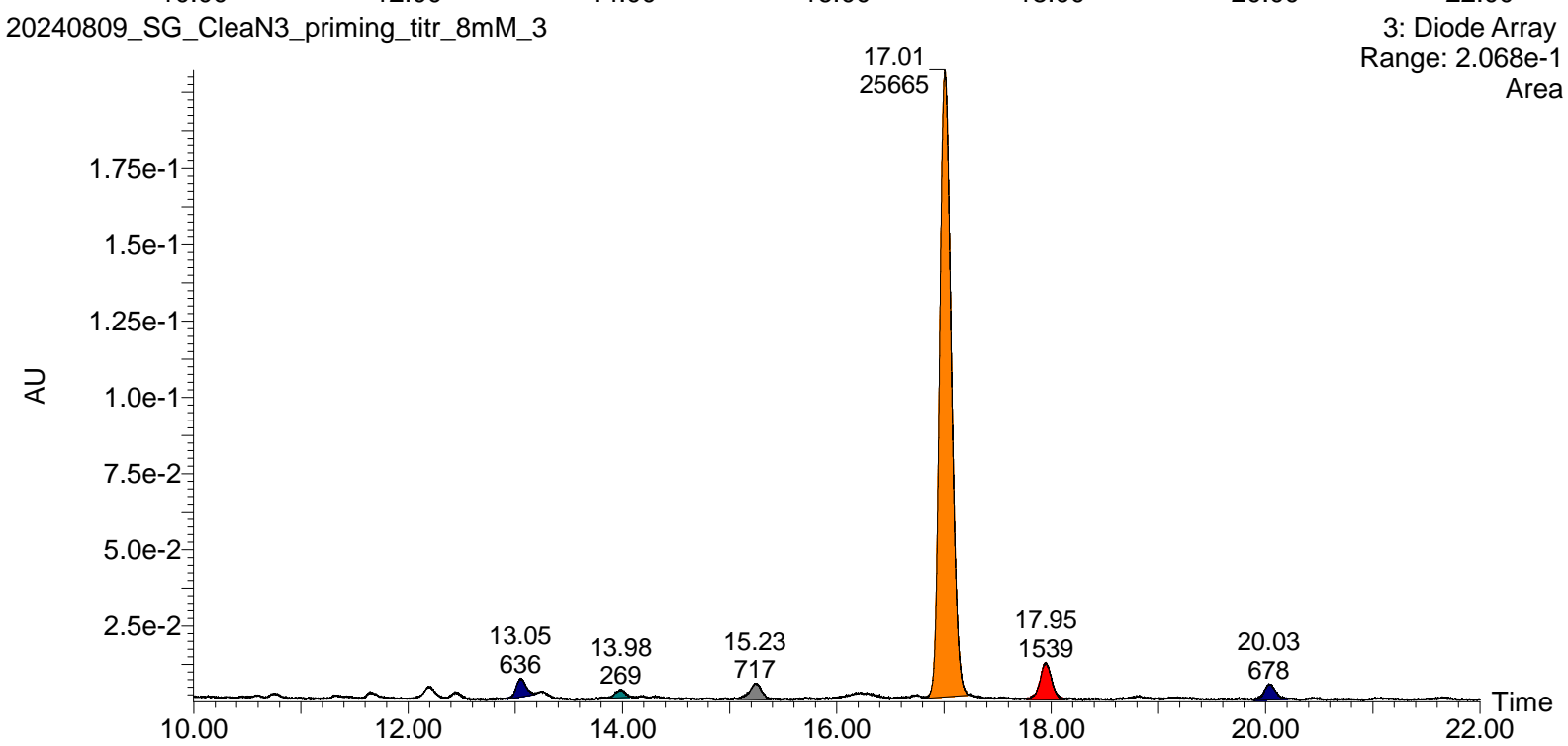

Supplement: Supplementary file 11 — Source Data [file 41467_2025_64257_MOESM11_ESM.zip › Source Data/Supplementary Figure 2/CleaN3 priming optimisation integration report 8 mM.pdf]

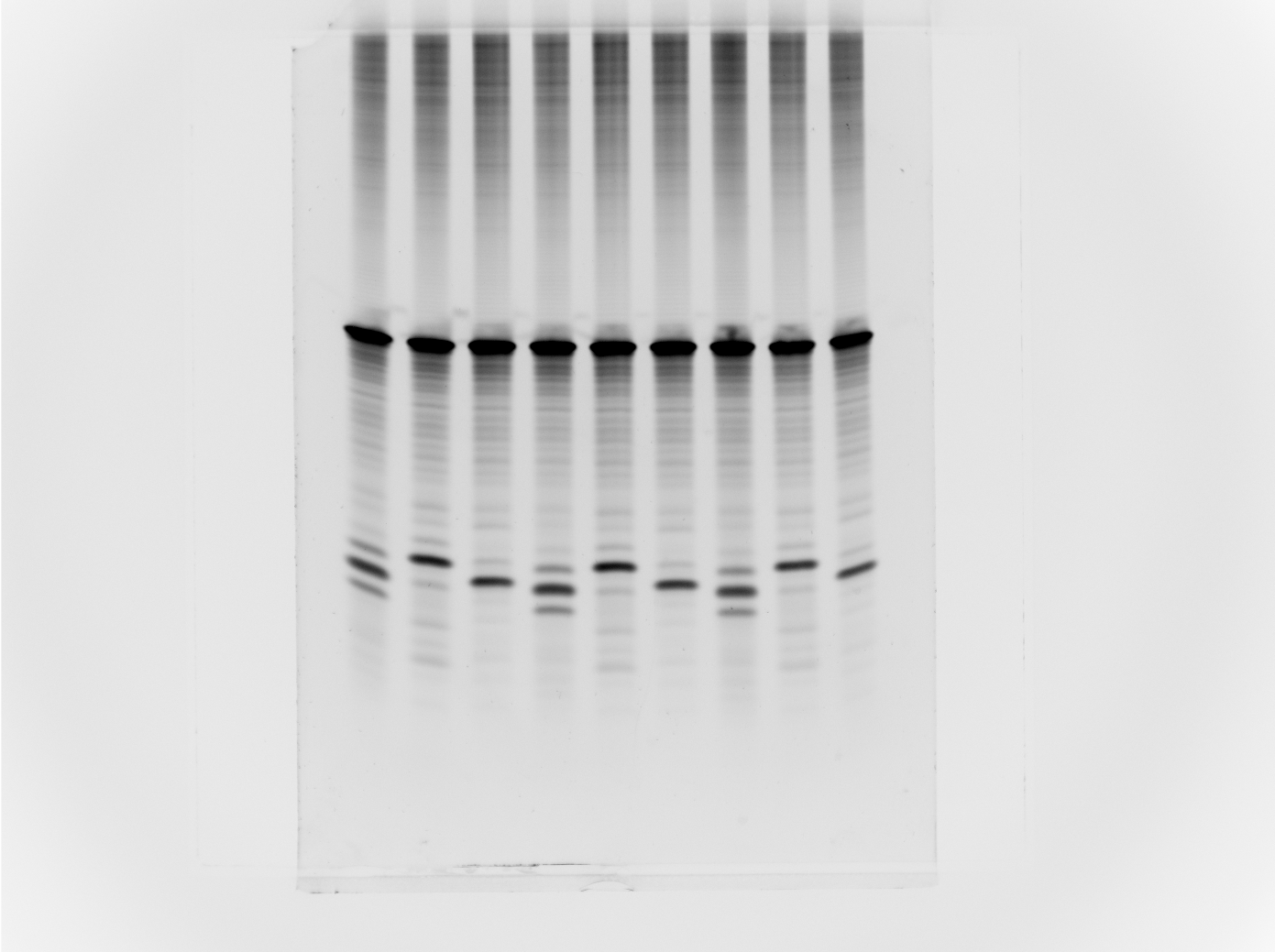

Supplement: Supplementary file 11 — Source Data [file 41467_2025_64257_MOESM11_ESM.zip › Source Data/Supplementary Figure 2/full gel.tif]

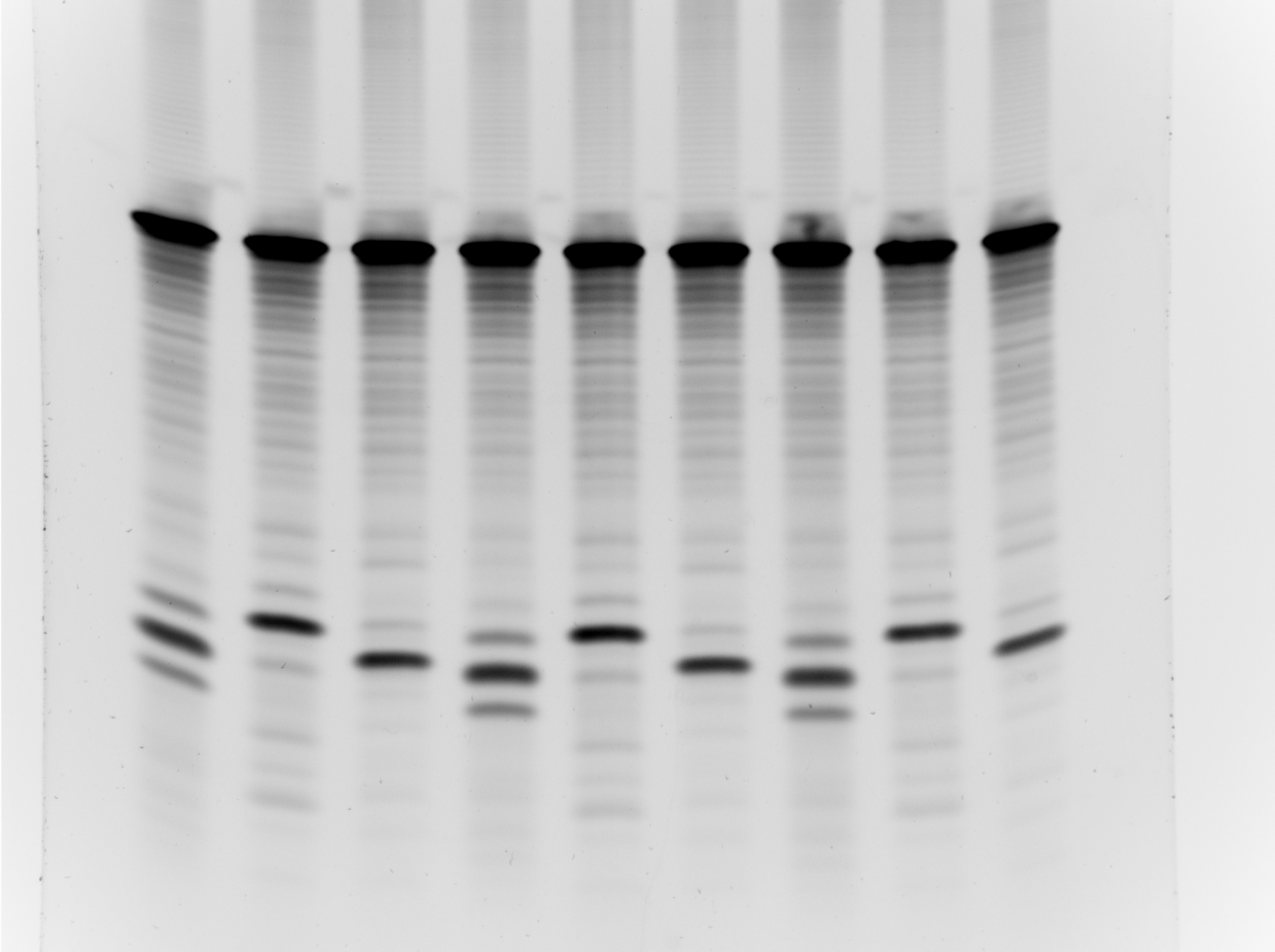

Supplement: Supplementary file 11 — Source Data [file 41467_2025_64257_MOESM11_ESM.zip › Source Data/Supplementary Figure 2/gel.tif]
